# Autonomic dysfunction and white matter microstructural changes in drug-naïve patients with Parkinson’s disease

**DOI:** 10.7717/peerj.5539

**Published:** 2018-09-13

**Authors:** Amir Ashraf-Ganjouei, Alireza Majd, Ali Javinani, Mohammad Hadi Aarabi

**Affiliations:** 1 Faculty of Medicine, Tehran University of Medical Sciences, Tehran, Iran; 2 Rheumatology Research Center, Tehran University of Medical Sciences, Tehran, Iran

**Keywords:** SCOPA-AUT, Parkinson Disease, Autonomic Dysfunction, Connectometry, Hoehn & Yahr stage, Diffusion MRI

## Abstract

**Background:**

Autonomic dysfunction (AD) is one of the non-motor features of Parkinson’s disease (PD). Some symptoms tend to occur in the early stages of PD. AD also has a great impact on patient’s quality of life. In this study, we aimed to discover the association between AD (Scales for Outcomes in Parkinson’s disease-Autonomic, SCOPA-AUT) and microstructural changes in white matter tracts in drug-naïve early PD patients to elucidate the central effects of autonomic nervous system impairments.

**Method:**

In total, this study included 85 subjects with PD recruited from the Parkinson’s Progression Markers Initiative (PPMI) database. Among the 85 PD patients, 38 were in Hoehn & Yahr stage 1 (HY1PD) and 47 were in stage 2 (HY2PD). Diffusion magnetic resonance imaging (DMRI) data were reconstructed in the MNI space using q-space diffeomorphic reconstruction to obtain the spin distribution function. The spin distribution function (SDF) values were used in DMRI connectometry analysis. We investigated through diffusion MRI connectometry the structural correlates of white matter tracts with SCOPA-AUT subscores and total score.

**Results:**

Connectometry analysis also revealed positive association with white matter density in bilateral corticospinal tract in HY1PD patients and negative association in genu of corpus callosum (CC) and, bilateral cingulum in both groups. In addition, there were associations between gastrointestinal, sexual, thermoregulatory and urinary items and structural brain connectivity in PD.

**Conclusion:**

Our study reveals positive correlation, suggesting neural compensations in early PD. Cingulum and CC tracts have well-known roles in PD pathology, compatible with our findings that bring new insights to specific areas of AD and its role in central nervous system (CNS) neurodegeneration, paving the way for using prodromal makers in the diagnosis and treatment of PD.

## Introduction

Parkinson’s disease (PD) is a common progressive neurodegenerative disorder that is characterized by loss of dopaminergic neurons mainly in the substantia nigra pars compacta (SNpc), diagnosed clinically on the basis of cardinal motor features such as resting tremor, bradykinesia, postural instability and muscular rigidity (Alexander, 2004). On the other hand, there are non-motor symptoms including sleep-related disturbances, behavioral impairments and autonomic dysfunction (AD), which may be due to the involvement of regions other than SNpc (Chaudhuri & Schapira, 2009; Verbaan et al., 2007). These symptoms have a huge effect on patients’ quality of life and might be potential clinical markers of PD’s premotor phase (Todorova, Jenner & Chaudhuri, 2014).

AD including deficits in gastrointestinal, urinary, thermoregulatory, cardiovascular, pupillomotor systems and sexual function has been considered a part of advanced PD (Asahina et al., 2013). However, recent evidence suggests that it might appear in the early stages of the disease, even years before a patient presents with classical motor features of PD (Hawkes, Tredici & Braak, 2010). With a prevalence up to 80%, numerous studies have proven that AD has a great impact on quality of life in PD individuals (Bostantjopoulou et al., 2016; Merola et al., 2017). Therefore, early diagnosis and effective treatment might reduce long-term morbidity and improve the overall outcome (Goldstein, 2014). Questionnaires are convenient tools for subjective assessment of autonomic symptoms and can be used as screening methods for AD in PD (Mantarova et al., 2013). Scales for Outcomes in Parkinson’s disease-Autonomic (SCOPA-AUT) is the most widely used questionnaire, created by Visser et al. (2004) in 2004 for assessment of dysfunctions associated with the autonomic nervous system in PD patients. SCOPA-AUT contains 25 questions assessing autonomic clinical symptoms in six categories including gastrointestinal (7 items), urinary (6 items), cardiovascular (3 items), thermoregulatory (4 items), pupillomotor (1 item) and sexual function (3 items for men and 2 items for women) (Visser et al., 2004).

To improve the usage of autonomic symptoms as markers for diagnosing PD or starting adjacent life-style changes and treatment, we need to put together different modalities such as subjective questionnaires, imaging techniques, etc. to assess these deficits. Diffusion magnetic resonance imaging (DMRI) is a non-invasive tool for evaluating white matter changes, especially in neurodegenerative disorders including PD (Basser, Mattiello & LeBihan, 1994). Since conventional DTI measures have some limitations, connectometry is used as a novel analytical approach with prominent advancements, using the concept of local connectome (Wen et al., 2016; Yeh, Badre & Verstynen, 2016). The local connectome is defined by the degree of connectivity (that is defined by the density of the diffusing spins) between voxels within a fascicle (Yeh, Badre & Verstynen, 2016). Thus, relevant variables can be inspected for significant associations with local connectomes.

In the current study, we aimed to assess the correlation between SCOPA-AUT score (alongside six subscores) and microstructural changes in white matter tracts in drug-naïve patients with Parkinson’s disease with Hoehn and Yahr (H&Y) stage I and II, to shed light on central effects of AD.

## Materials and Methods

### Participants

Participants involved in this study were recruited from the Parkinson’s Progression Markers Initiative (PPMI, http://www.ppmi-info.org/). The study was approved by the institutional review board of all participating sites in Europe, including Attikon University Hospital (Greece), Hospital Clinic de Barcelona and Hospital Universitario Donostia (Spain), Innsbruck University (Austria), Paracelsus-Elena Clinic Kassel/University of Marburg (Germany), Imperial College London (UK), Pitié-Salpêtrière Hospital (France), University of Salerno (Italy), and in the USA, including Emory University, Johns Hopkins University, University of Alabama at Birmingham, PD and Movement Disorders Center of Boca Raton, Boston University, Northwestern University, University of Cincinnati, Cleveland Clinic Foundation, Baylor College of Medicine, Institute for Neurodegenerative Disorders, Columbia University Medical Center, Beth Israel Medical Center, University of Pennsylvania, Oregon Health & Science University, University of Rochester, University of California at San Diego, University of California, San Francisco. Written informed consent was obtained from all participants before study enrollment. The study was performed in accordance with relevant guidelines and regulations. These participants were tested and confirmed negative for any neurological disorders apart from PD. The participants’ PD status was confirmed by Movement Disorder Society-Unified Parkinson’s Disease Rating Scale (MDS-UPDRS), and the loss of dopaminergic neurons was observed on DAT scans. Only drug-naïve PD patients in early stages of the disease were investigated in this study. Subjects were only excluded if imaging failed specific quality control criteria. Motor severity was evaluated using UDPRS-III. Montreal Cognitive Assessment (MoCA) was used to investigate global cognitive function. 15-item geriatric depression scale (GDS) and The University of Pennsylvania Smell Identification Test (UPSIT) were used to evaluate neuropsychiatric and olfaction function status respectively.

### Data acquisition

Data used in the preparation of this article were obtained from the Parkinson’s Progression Markers Initiative (PPMI) database (http://www.ppmi-info.org/data) (Marek et al., 2011). This dataset was acquired on a 3 Tesla Siemens scanner, producing 64 Diffusion MRI (repetition time = 7,748 MS, echo time = 86 ms; voxel size: 2.0 × 2.0 × 2.0 mm3; field of view = 224 × 224 mm) at b = 1,000 s/mm2 and one b0 image along with a 3D T1-weighted structural scan (repetition time = 8.2 ms, echo time = 3.7 ms; flip angle = 8° , voxel size: 1.0 × 1.0 × 1.0 mm3; field of view = 240 mm, acquisition matrix =240 × 240).

### Diffusion MRI processing

The Diffusion MRI data were corrected for subject motion, eddy current distortions, and susceptibility artifacts due to the magnetic field inhomogeneity using Explore DTI toolbox (Leemans et al., 2009). We performed quality control analysis on the subjects’s signals based on the goodness-of-fit value given in QSDR reconstruction of fibers. Each QSDR reconstruction file has a goodness-of-fit value quantified by R2. For example, an R82 indicates a goodness-of-fit between the subject and template of 0.82 total. We excluded cases in which the R2 value did not reach a threshold of 0.6 otherwise.

### Diffusion MRI connectometry

The diffusion data were reconstructed in the MNI space using q-space diffeomorphic reconstruction to obtain the spin distribution function (SDF) (Yeh & Tseng, 2011).

Connectometry is a novel approach in the analysis of diffusion MRI signals that simply tracks the correlation of white matter fibers with a variable of interest. Connectometry approach extracts the SDF in a given fiber orientation, as a measure of water density along that direction. There are a multitude of diffusion indices derived from spin density i.e., SDF, quantitative anisotropy (QA) being one of them (Yeh, Tang & Tseng, 2013). QA of each fiber orientation gives the peak value of water density in that direction or tracts with significant correlation to a variable of interest.

Diffusion MRI connectometry was used to study the effect of SCOPA-AUT and its subscores. A multiple regression model was used to investigate correlation of SCOPA-AUT score, and gastrointestinal, urinary, cardiovascular, thermoregulatory, pupillomotor, and sexual subscores with white matter QA, in a total of 85 PD patients, from each of the two groups, considering age, sex, GDS, MoCA, handedness, number of relatives with PD and UPDRS-III as covariates in the model. The SDF was normalized. A T-score threshold of 2.5 was assigned to select local connectomes, and the local connectomes were tracked using a deterministic fiber tracking algorithm. A length threshold of 40 mm was used to select tracks. The seeding density was 50 seeds per mm^3^. To estimate the false discovery rate, a total of 2,000 randomized permutation was applied to the group label to obtain the null distribution of the track length. The analysis was conducted using publicly available software DSI Studio (http://dsi-studio.labsolver.org).

### Statistical analysis

Demographic data were analyzed using IBM SPSS Statistics for Windows, version 22 (IBM Corp., Armonk, N.Y., USA). Compliance of variables with normal distribution was tested with probability graphics and Shapiro–Wilk test. Pearson’s chi-square was used to assess nominal variables across PD subgroups. Independent-sample t test (for variables with normal distribution) and Mann–Whitney *U* test (for variables without normal distribution) were used to assess differences between groups. *P* values less than 0.05 were considered statistically significant.

## Results

### Demographic and Clinical Findings

In total, this study included 85 subjects with PD recruited from the PPMI database with good quality baseline DWI data. Among the 85 PD patients, 38 patients were in Hoehn & Yahr stage 1 (HY1PD) and 47 patients were in stage 2 (HY2PD). None of the participants scored below the cut-off score for MoCA to be marked as demented. The mean disease duration was comparable between HY1PD and HY2PD patients (*p* = , 0.117). Demographic and clinical data are presented in Table 1. There were no significant group differences in age (*p* = 0.09), gender distribution (*p* = 0.264), handedness (*p* = 0.67), or years of education (*p* = 0.189). There were also no significant differences when comparing general cognitive function (*p* = 0.571), depressive disorder (*p* = 0.518) and olfaction dysfunction (*p* = 0.055). As expected, the HY2PD patients had greatest motor severity based on UPDRS-III score (*p* < 0.001).

**Table 1:** Demographic information of patients with Parkinson’s disease.

**Characteristic**	**HY1PD** (*n* = 38)	**HY2PD** (*n* = 47)	*P* value
Age, mean (SD) [95% CI], y	55.7 (8.2) [53.0–58.4]	59.0 (9.3) [56.2–61.8]	0.090^a^
Female/male, No. (%male)	17/21 (52.3)	15/32 (68.1)	0.264^b^
Left-handed/right-handed, No.(%right-handed)	2/35 (92.1)	4/40 (85.1)	0.678^b^
Education, mean (SD) [95% CI], y	14.7 (2.8) [13.8–15.6]	15.5 (2.7) [14.7–16.3]	0.189^a^
Disease duration, mean (SD) [95% CI], y	5.2 (5.2) [3.4–6.9]	7.9 (7.8) [5.6–10.2]	0.117^c^
UPRDS-III, mean (SD) [95% CI]	15.3 (6.0) [13.3–17.3]	24.9 (8.2) [22.5–27.3]	<0.001^a^
MOCA, mean (SD) [95% CI]	27.6 (2.0) [27.0–28.2]	27.9 (1.8) [27.3–28.4]	0.571^c^
GDS, mean (SD) [95% CI]	4.5 (1.0) [4.2–4.9]	4.3 (1.4) [3.9–4.7]	0.518^c^
UPSIT, mean (SD) [95% CI]	26.2 (6.3) [24.2–28.3]	22.9 (9.0) [20.2–25.5]	0.055^a^
SCOPA-AUT score, mean (SD) [95% CI]	7.4 (5.4) [5.6–9.2]	9.7 (5.8) [8.0–11.4]	0.039^c^

**Notes.**

Abbreviations HY1PD Hoehn and Yahr stage 1 Parkinson disease HY2PD Hoehn and Yahr stage 2 Parkinson disease UPDRS-III MDS-Unified Parkinson’s Disease Rating Scale-Motor Subscale MOCA Montreal cognitive assessment GDS Geriatric depression scale UPSIT University of Pennsylvania Smell Identification Test SCOPA-AUT Scales for Outcomes in Parkinson’s disease

a Based on independent-sample *t* test.

b Based on *χ*^2^ test or Fisher’s exact test.

c Based on Mann–Whitney *U* test.

### Multiple regression analysis

**Figure 1:**
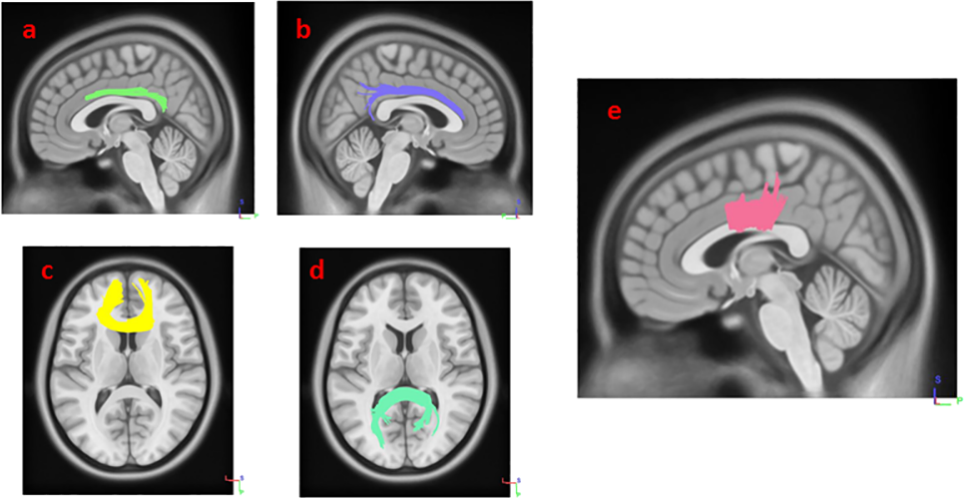
White matter pathways with significantly negative association with SCOPA-AUT in HY1PD patients (FDR = 0.0168067). (A) left cingulum, (B) right cingulum, (C) genu, (D) splenium, and (E) body of corpus callosum.

**Figure 2:**
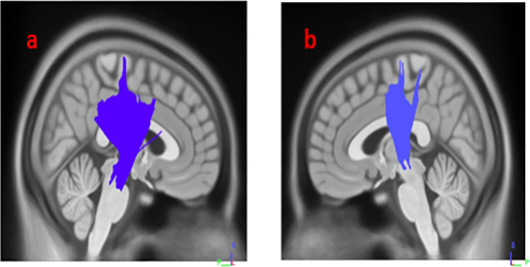
White matter pathways with significantly positive association with SCOPA-AUT in HY1PD patients (FDR = 0.0404073). (A) right corticospinal tract, (B) left corticospinal tract.

**Figure 3:**
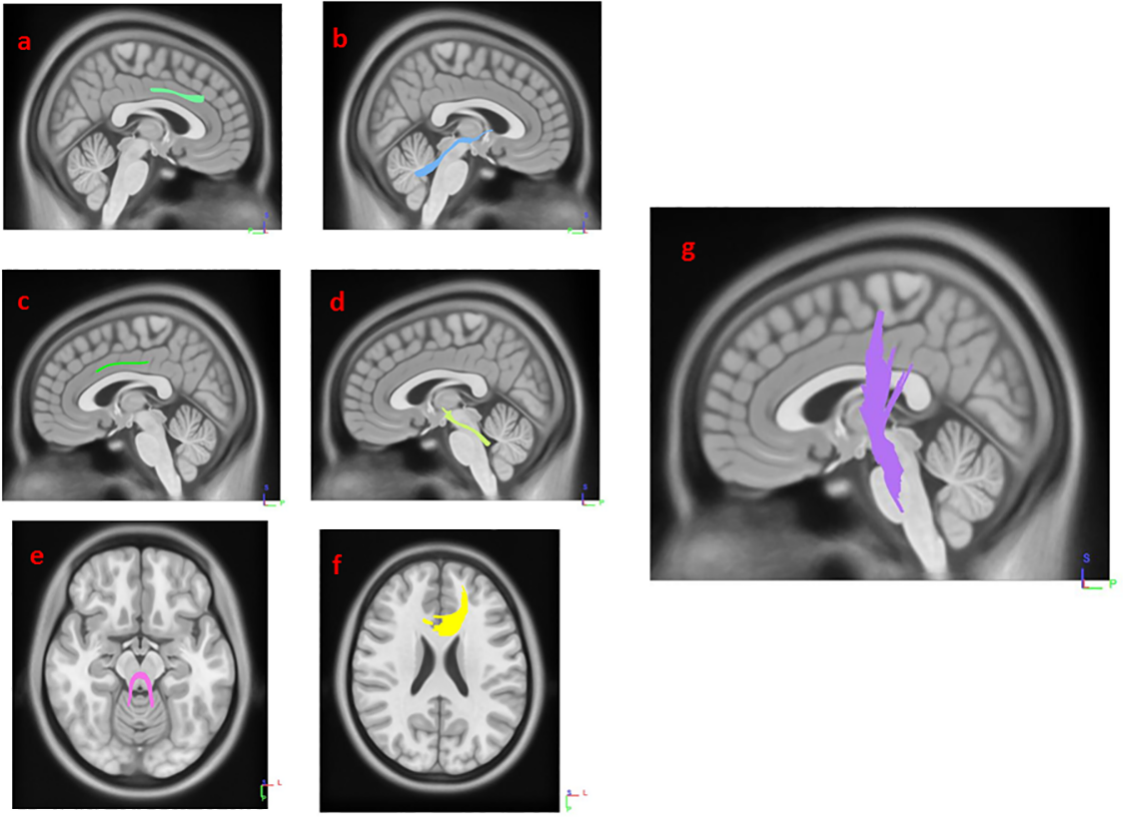
White matter pathways with significantly negative association with SCOPA-AUT in HY2PD patients (FDR = 0.0355872). (A) right cingulum, (B) right supra cerebellar peduncle, (C) left cingulum, (D) left supra cerebellar peduncle, (E) middle cerebellar peduncle, (F) genu, and (G) left corticospinal tract.

Results from multiple regression models in Diffusion MRI connectometry revealed areas where white matter QA was correlated with SCOPA-AUT score in each group after adjustment for age, sex, GDS and MoCA scores, number of relatives with PD and UPDRS-III. The SCOPA-AUT score could predict the highest number of fibers with significant positive/negative correlation in the QA, in the multiple regression models in HY1PD. The body, genu and splenium of corpus callosum (CC) and bilateral cingulum had significant negative correlation (FDR = 0.0168067), and bilateral corticospinal tract had significant positive correlation with SCOPA-AUT scores in HY1PD (FDR = 0.0404073) (Figs. 1 and 2). Genu of CC, bilateral cingulum, middle cerebellar peduncle, bilateral superior cerebellar peduncle and left corticospinal tract showed significant negative correlation in QA with SCOPA-AUT score in HY2PD (FDR = 0.0355872) (Fig. 3). Finally, we have used a similar analysis for each subscore of SCOPA-AUT and the results are presented in Table 2.

**Table 2:** White matter tracts showing significant correlation with each autonomic function in Parkinson’s disease patients.

Autonomic function^*^	±	**Significant tracts**
Gastrointestinal	−	Middle cerebellar peduncle, Bilateral Cingulum, Body and Genu and Splenium CC, Bilateral Corticospinal Tract
Pupillomotor	−	Bilateral Cingulum, Body and Genu CC, Fornix, Left ILF
Sexual	−	Middle cerebellar peduncle, Bilateral Cingulum, Body and Genu CC, Bilateral Frontopontine Tract, Bilateral IFOF
Thermoregulatory	−	Middle cerebellar peduncle, Bilateral Cingulum, Body and Genu and Splenium CC, Bilateral UF, Bilateral ILF, Right AF
Urinary	+	Bilateral Cingulum, Body and Genu CC, Fornix, Left ILF

**Notes.**

Abbreviations + positive correlation − negative correlation CC Corpus Callosum IFOF inferior fronto-occipital fasciculus ILF Inferior Longitudinal Fasciculus UF Uncinate Fasciculus AF Arcuate Fasciculus

* There was not any significant correlation between investigated tracts and cardiovascular score.

## Discussion

In the current study, we aimed to investigate the association between SCOPA-AUT scores and microstructural changes of the central nervous system, trying to find AD footprints in early stages of PD. The connectometry analysis revealed that SCOPA-AUT score has a negative association with the connectivity in the CC and cingulum bilaterally, in both HY1PD and HY2PD groups. Moreover, there was negative association between SCOPA-AUT and structural brain connectivity in middle cerebellar peduncle, superior cerebellar peduncle bilaterally and left corticospinal tract in HY2PD patients. These results suggest that in early PD, autonomic dysfunction might be associated with deficits in the mentioned tracts, preceding the involvement SNpc regarding motor symptoms. Interestingly, a positive association was observed between bilateral corticospinal tract connectivity and SCOPA-AUT in HY1PD patients. It is hypothesized that the positive association with this tract might be due to the compensation mechanism following the reduced function of involved parts of the brain.

Parkinson’s disease brings too much burden on patients’ lives. This burden results from both motor and non-motor impairments. Although recent studies have demonstrated that non-motor features are responsible for a considerable proportion of the disabilities, our knowledge about their properties and development is still inadequate (Chaudhuri, Healy & Schapira, 2006; Merola et al., 2017; Muller et al., 2013). Additionally, since a proportion of non-motor symptoms are treatable, besides having more knowledge of their pathophysiology and the possibility of early diagnosis, they could play an important role in PD treatment (Verbaan et al., 2007). SCOPA-AUT is a valuable tool for assessing autonomic symptoms in PD. However, like any other marker, it cannot accomplish the four key properties of a prodromal diagnostic marker alone: certainty, specificity, appropriate lead-time and cost (Postuma & Berg, 2016). Our results suggest that SCOPA-AUT score is associated with altered connectivity in certain white matter tracts. Hence the subjective assessment and DMRI findings could be put together to support AD as a potential diagnostic prodromal marker.

As discussed previously, AD can present with numerous symptoms attributable to the involvement of various systems. For instance, several studies have investigated the prevalence and relative risk of having constipation, which is the most common autonomic-related symptom in PD patients (Gao et al., 2011; Lin et al., 2014; Savica et al., 2009). These studies suggest that with a prevalence of 15–20% among healthy individuals and a relative risk of 2–3, constipation might occur 20 or more years before developing PD, but lacks acceptable specificity or predictive value for PD (Postuma & Berg, 2016; Savica et al., 2009). It is hypothesized that since the influence of the vagus nerve decreases in the rostrocaudal direction, gastrointestinal symptoms such as constipation more likely reflects the involvement of the enteric nervous system (Coon, Cutsforth-Gregory & Benarroch, 2018). In fact, few studies have investigated central neuropathology of gastrointestinal dysfunction in PD from an imaging aspect (Abbott et al., 2001; Kaufmann & Goldstein, 2013). However, for the first time we have reported significant negative correlations between gastrointestinal subscore of SCOPA-AUT and certain tracts (including middle cerebellar peduncle, bilateral cingulum, body and genu and splenium CC and bilateral corticospinal tract). Our results suggest that white matter alterations might also contribute to AD in PD patients.

Moreover, studies by Plouvier et al. (2014) and Schrag et al. (2015) showed that patients with urinary dysfunction are at higher risk for developing PD, with a relative risk of 2.3 and odd ratio of 1.9, respectively. Other studies have demonstrated that bladder dysfunction is associated with disease severity and progresses as PD become more advanced (Araki et al., 2000). Bladder hyperactivity in PD is also associated with cell loss in substantia nigra (Yoshimura et al., 2003). We have shown that the urinary subscore of SCOPA-AUT is positively correlated with certain white matter tracts, proposing a role for altered connectivity in autonomic dysfunction. These findings suggest that similar to gastrointestinal symptoms, urinary dysfunction probably has a low specificity, but being easy to monitor, might be used in combination with other symptoms and markers to make a suitable screening test.

On the other hand, few studies have investigated sexual dysfunction as a symptom of autonomic nervous system impairment (Gao et al., 2007; Yang et al., 2017). For example, Gao et al. (2007) showed that compared to men with no erectile dysfunction, those with erectile dysfunction have a relative risk of 3.8 for developing PD. Moreover, impaired sexual behavior, arousal and orgasm but not sexual fantasy, are observed in PD (Yu et al., 2004). Dopaminergic mechanisms are probably involved in sexual function (Kaufmann & Goldstein, 2013). However, the exact pathogenesis of sexual dysfunction in PD is still unclear. We have demonstrated that changes in white matter tracts including the middle cerebellar peduncle, bilateral cingulum, body and genu of corpus callosum might be involved in this regard.

Cardiovascular symptoms are also prevalent in patients with PD. For instance, several studies have shown that individuals with orthostatic hypertension have increased risk of developing PD (Gibbons & Freeman, 2015; Schrag et al., 2015). Another study by Postuma et al. (2015) showed that in patients with idiopathic rapid eye movement (REM) sleep behavior disorder (RBD), higher baseline cardiovascular SCOPA-AUT is associated with increased risk of developing PD. Moreover, Pyatigorskaya et al. (2016) used DTI to investigate cardiac-related AD correlation with medulla oblongata damage. Their results suggested a strong correlation between brain stem damage and AD in patients with PD. Interestingly, unlike other categories we did not find any significant correlation between the cardiovascular subscore of SCOPA-AUT and white matter alterations. This might be the result of a more prominent role of cardiac sympathetic denervation and brain stem damage in cardiovascular dysfunction in PD (Kamagata et al., 2012).

The cingulum has a number of white matter tracts projecting from the cingulate gyrus (and one of the important parts of limbic system) and has been proposed to be involved in PD. Previous investigations have revealed significant cingulum atrophy in early PD, besides its role in visuospatial processing, memory access and REM sleep dysfunction (Hall et al., 2016; Lim et al., 2016; Tessa et al., 2014). Kamagata et al. (2012) reported decreased fractional anisotropy (FA) in both the anterior and posterior cingulate fibers in PD patients with dementia compared to controls. Moreover, microstructural changes in cingulum and olfaction dysfunction in early PD patients and REM sleep behavior disorder are reported (Ansari et al., 2017; Rahmani & Aarabi, 2017). In 2016, Duncan et al. (2016) reported the involvement of cingulate gyrus and its white matter connections in PD-related cognitive dysfunction, even before gray matter loss became evident. In the current study, we have found significant correlations between cingulum connectivity and SCOPA-AUT score in both H&Y 1 and 2 stages, besides all other subscores except the cardiovascular. Putting together the critical role of the cingulate in regulation of autonomic function and the results of our study, it suggests the cingulum as a vulnerable area that is involved in early PD, especially in those patients with autonomic symptoms.

The fornix is a collection of fibers acting as a major connection for the hippocampus. Although not all axons in the fornix originate from hippocampus, its integrity is linked to the integrity of hippocampus and reduced fornix FA is observed in subjects with hippocampal atrophy (Kantarci, 2014). Our results show that there are significant correlations between urinary subscores and connectivity fornix. Previous studies have also demonstrated the role of hippocampus in autonomic actions (Goswami, Frances & Shoemaker, 2011; Norton, Luchyshyn & Shoemaker, 2013).

The cerebellum, with its key role in vestibular action and somatomotor coordination, has been known to have major influences on autonomic function, such as controlling blood pressure (Macey et al., 2015; Rector, Richard & Harper, 2006). We have also provided evidence for white matter alternations in cerebellar peduncles, which were significantly correlated with total SCOPA-AUT score besides gastrointestinal, sexual and thermoregulatory subscores in PD patients. These results suggest that cerebellum-related changes in PD might be significantly correlated with comorbidities such as AD.

A direct correlation between mini-mental status (MMSE) score of PD patients and changes in DTI tractography in corpus callosum is reported (Wiltshire et al., 2010). In 2016, Zhang et al. (2016) reported greater increase of radial diffusivity of corpus callosum in PD patients during PD progression comparing with normal healthy subjects. The CC also plays a role in motor features of PD, and microstructural CC changes are related to freezing of gait and bradykinesia (Hall et al., 2016). As cingulum, CC and other mentioned tracts have prominent roles in the pathogenesis of PD, the association of reduced connectivity and increased SCOPA-AUT score that was suggested by this study can pave the way for future use of prodromal factors in the diagnosis of PD.

Regarding the strength of the conclusion, several limiting factors should be considered including the limited number of cases and lack of follow-up. Furthermore, SCOPA-AUT score consists of diverse autonomic symptoms. Although the correlation between specific categories of symptoms and imaging findings was assessed in this study, further evaluations using other tools and clinical finding would be of great value.

## Conclusion

Assessment of microstructural changes in PD is becoming a powerful method and will hopefully shed light on the footprints of the disease initiation and course. To the best of our knowledge, this is the first study investigating the correlation of AD and white matter connectivity in PD patients. This study revealed a negative association between SCOPA-AUT score and the connectivity in cingulum bilaterally, genu of corpus callosum, middle cerebellar peduncle, superior cerebellar peduncle bilaterally and left corticospinal tract. Further studies are needed in order to put together these markers and reduce the huge burden of PD on patients and society.

##  Supplemental Information

10.7717/peerj.5539/supp-1 Supplemental Information 1 Dataset of demographic cases used in this study

10.7717/peerj.5539/supp-2 Supplemental Information 2 PPMI protocol study

10.7717/peerj.5539/supp-3 Supplemental Information 3 PPMI operation manual

## Additional Information and Declarations

### Data Availability

The following information was supplied regarding data availability: The raw data are provided in the Supplemental Files.
