# Supplementary material for: Autonomic dysfunction and white matter microstructural changes in drug-naïve patients with Parkinson’s disease
Source: PeerJ. 2018 Sep 13;6:e5539. doi: 10.7717/peerj.5539 (PMC6139241; doi:10.7717/peerj.5539)
Supplement: Supplemental Information 2 [file peerj-06-5539-s002.pdf]


**Trial No.:**	001
**Title:**	The Parkinson's Progression Markers Initiative (PPMI)
**Sponsor:**	Michael J. Fox Foundation
**Principal Investigator:**	Kenneth Marek, MD
**Date of Protocol:**	November 20, 2017
**Final Version:**	14.0
**Planned Dates Of Trial:**	April 1, 2010 – Sept 30, 2023

CONFIDENTIAL
This document is a confidential communication of the Michael J. Fox Foundation. The recipient agrees that no information contained herein will be published or disclosed without written approval. This document may be disclosed to appropriate institutional review boards, independent ethics committees, or duly authorized representatives of the U.S. Food & Drug Administration, European Medicines Agency, or other regulatory authorities as appropriate; under the condition that confidentiality is requested.

Investigator and Core Facility Contact Information

Principal Investigator

Ken Marek, MD
President and Senior Scientist
Institute of Neurodegenerative Disorders
60 Temple St., Suite 8B, New Haven, CT 06510
Ph : 203-401-4353
kmarek@indd.org

PPMI Cores

Bioanalytics Core

University of Pennsylvania
3600 Spruce Street, 3rd Fl, Maloney Bldg, Philadelphia, PA 19104
Principal Investigators: John Q. Trojanowski, MD, PhD
Ph: 215-662-6399
trojanow@mail.med.upenn.edu
Les Shaw, PhD
Ph: 215-662-6575
shawlmj@mail.med.upenn.edu

Bioinformatics Core

Laboratory of Neuro Imaging (LONI)
The Institute for Neuroimaging and Informatics
Keck School of Medicine of USC
University of Southern California
2001 North Soto Street – Room 102
Los Angeles, CA 90032
Principal Investigator: Arthur W. Toga, PhD
Ph: (323) 442-7246
toga@loni.ucla.edu

Biorepository Core

Indiana University
Indiana University School of Medicine
410 W. 10th Street (HS4021), Indianapolis, IN 46202
Principal Investigator: Tatiana Foroud, PhD
Ph: 317-278-1291
tforoud@iu.edu

Clinical Core
Clinical Trials Coordination Center (CTCC)
University of Rochester
265 Crittenden Blvd, Rochester, NY 14642
Principal Investigator: Karl Kieburtz, MD, MPH
Ph: 585-275-1557
Karl.kieburtz@chet.rochester.edu
Directors: Cynthia J. Casaceli, MBA
Ph: 585-276-3795
Cindy.casaceli@chet.rochester.edu

Ray Dorsey, MD, MBA
Ph: 585-275-7311
Ray.Dorsey@chet.rochester.edu

Sr. Project Manager: Renee Wilson, MA
Ph: 585-273-4242
Renee.wilson@chet.rochester.edu

Genetics Core:
Laboratory of Neurogenetics
National Institute on Aging/NIH
Building 35, Room 1A-1000, 35 Lincoln Drive Bethesda, MD 20892
Principal Investigator: Andrew Singleton, PhD
Ph: 301-451-6079
singleta@mail.nih.gov

Genetics Coordinating Core
Department of Medical and Molecular Genetics
Indiana University School of Medicine
410 W. 10th Street (HS4021), Indianapolis, IN 46202
Principal Investigator: Tatiana Foroud, PhD
Ph: 317-278-1291
tforoud@iu.edu

Imaging Core
Institute of Neurodegenerative Disorders
60 Temple St., Suite 8B, New Haven, CT 06510
Principal Investigator: John Seibyl, MD
Ph: 203-401-4300
jseibyl@indd.org

Olfactory Core	Institute of Neurodegenerative Disorders<br>60 Temple St., Suite 8B, New Haven, CT 06510<br>Principal Investigator: Ken Marek, MD<br>Ph : 203-401-4353<br>kmarek@indd.org
Participant Coordination Core	Institute of Neurodegenerative Disorders<br>60 Temple St., Suite 8B, New Haven, CT 06510<br>Principal Investigator: Ken Marek, MD<br>Ph : 203-401-4353<br>kmarek@indd.org
Pathology Core	Department of Medical and Molecular Genetics<br>Indiana University School of Medicine<br>410 W. 10th Street (HS4021), Indianapolis, IN 46202<br>Principal Investigator: Tatiana Foroud, PhD<br>Ph: 317-278-1291<br>tforoud@iu.edu
RBD Core	Hephata Hessisches Diakoniezentrum e. V.<br>Chefarzt der Hephata-Klinik<br>Schimmelpfengstr. 6<br>34613 Schwalmstadt Germany<br>Principal Investigator: Geert Mayer, MD<br>Ph: 49-6691/18-2001<br>geert.mayer@hephata.com
Statistics Core	Clinical Trials Statistical and Data Management Center<br>University of Iowa<br>2450 University Capitol Center, Iowa City, Iowa 52242<br>Principal Investigator: Christopher Coffey, PhD<br>Ph: 319-384-4197<br>Christopher.coffey@uiowa.edu

PROTOCOL APPROVAL

Amendment 13

The Parkinson's Progression Markers Initiative (PPMI)

Kenneth Marek, MD Date
Principal Investigator

Ray Dorsey, MD, MBA Date
CTCC Director

Todd Sherer, PhD Date
Michael J Fox Foundation (Sponsor)

INVESTIGATOR AGREEMENT

Protocol Amendment 13
The Parkinson's Progression Markers Initiative (PPMI)

I have carefully read this protocol including all appendices and agree that it contains all the necessary information for conducting the study safely.

I will conduct this study in strict accordance with this protocol and according to the current Good Clinical Practice (GCP) regulations and International Conference on Harmonization (ICH) guidelines, and local regulatory requirements. Any changes in procedure will only be made if necessary to eliminate immediate hazards and/or to protect the safety, rights or welfare of subjects.

I will provide copies of the protocol and all other information relating to this project, which were furnished to me, to all physicians and other study personnel responsible to me who participate in this study. I will discuss this information with them to assure that they are adequately informed regarding the conduct of the study.

I agree to keep records on all subject information (case report forms, informed consent statements and all other information collected during the study) in accordance with the current GCP, ICH, local, national and European regulations.

__________ ____

Site Number Printed Site Name

____

Printed Site Investigator Name

_______________________________________ ______________________

Site Investigator Signature Date

List of Abbreviations and Definitions

AD	Alzheimer disease
ADL	activities of daily living
ADNI	Alzheimer Disease Neuroimaging Initiative
AMADEUS	American and European Union SPECT Imaging Consortium
18-F-AV-133	18F-(1)fluoropropyldihydrotetrabenazine
β-CIT	2β-carboxymethoxy-3β-(4-iodophenyl) tropane
CFR	Code of Federal Regulations
β-CFT	2β-carboxymethoxy-3β-(4-fluorophenyl) tropane
Beta-HCG	beta-human chorionic gonadotropin
CFR	Code of Federal Regulations
CRF	Case Report Form
CSF	cerebral spinal fluid
CSOC	Clinical Study Oversight Committee
CTCC	Clinical Trials Coordination Center
DAT	dopamine transporter
DaTSCAN	Ioflupane I-123 injection
DNA	Deoxyribonucleic acid
DTI	diffusion tensor imaging
ECG	Electrocardiogram
eCRF	electronic Case Report Form
EDC	electronic data capture
ELLDOPA	Earlier versus Later Levodopa Therapy in Parkinson Disease
ESS	Epworth Sleepiness Scale
F-Dopa	Fluorodopa
GBA	Glucocerebrosidase (official name: glucosidase, beta, acid)
GCP	Good Clinical Practice
GDS	Geriatric Depression Scale
HC	Healthy Control
HIPAA	Health Insurance Portability and Accountability Act
HSPP	Human Subject Protection Program
HVLT-R	Hopkins Verbal Learning Test – Revised
ICH	International Conference on Harmonization
IEC	Independent Ethics Committee
iPSC	Induced pluripotent stem cell
IRB	Institutional Review Board
LONI	Laboratory of Neuro Imaging
LRRK2	Leucine-rich repeat kinase 2
MAO-B	Monoamine Oxidase-B
MDS-UPDRS	Movement Disorder Society Unified Parkinson Disease Rating Scale
MJFF	Michael J. Fox Foundation
MoCA	Montreal Cognitive Assessment
MRI	magnetic resonance imaging
OPDM	Objective Parkinson's Disease Measurement
OTC	over-the-counter
PARS	Parkinson Associated Risk Study
PBMC	Peripheral blood mononuclear cell

List of Abbreviations and Definitions (continued)

PD	Parkinson disease
PD RFQ-U	Parkinson's Disease Risk Factor Questionnaire
PET	positron emission tomography
PPMI	Parkinson's Progression Markers Initiative
PSG	Polysomnography
PW	premature withdrawal
QA	quality assurance
QC	quality control
QUIP-S	Questionnaire for Impulsive-Compulsive Disorders
RBDSQ	REM Sleep Behavior Disorder Screening Questionnaire
REM	rapid eye movement
SAB	Scientific Advisory Board
SAE	serious adverse event
SC	Steering Committee
SCOPA-AUT	Scales for Outcomes in Parkinson's Disease
SDMT	Symbol Digit Modalities Test
SNCA	synuclein, alpha Gene
SPECT	single-photon emission computed tomography
ST	symptomatic therapy
STAI	State-Trait Anxiety Inventory
SWEDD	Scans Without Evidence of Dopaminergic Degeneration
UPSIT	University of Pennsylvania Smell Identification Test
VMAT2	Vesicular Monoamine Transporter 2

PPMI
PROTOCOL SYNOPSIS

**Protocol Number**	Study 001
**Protocol Title**	**THE PARKINSON'S PROGRESSION MARKERS INITIATIVE (PPMI)**
**Sponsor**	Michael J Fox Foundation
**Investigators**	Multi-center trial
**Study Centers**	About 35 centers in North America, Europe, Israel, and Australia
**Study Period**	Up to 13 years
**Study Objective and Specific Aims**	The primary objective of this study is to identify clinical, imaging and biologic markers of PD progression for use in clinical trials of disease-modifying therapies.<br><br>The specific aims to accomplish the primary objective are:<br>a. Establish standardized protocols for acquisition, transfer and analysis of clinical, imaging and biologic data that can be used by the PD research community.<br>Develop a comprehensive and uniformly acquired clinical and imaging dataset and biological samples that can be used to estimate the mean rates of change and the variability around the mean of clinical, imaging and biomic outcomes in early PD patients, prodromal PD subjects, and PD subjects with a LRKK2, GBA or SNCA mutation.<br>Investigate existing and identify novel clinical, imaging, and biomic Parkinson disease progression markers to identify quantitative individual measures or combination of measures that demonstrate optimum interval change in PD patients in comparison to healthy controls, SWEDD subjects, Prodromal subjects, PD subjects with a LRRK2, GBA or SNCA mutation, unaffected LRRK2, GBA or SNCA mutation carriers, or in sub-sets of PD patients defined by baseline assessments, progression milestones and/or rate of clinical, imaging, genetic mutations, or biomic change.<br>Conduct preliminary verification studies on promising biological markers using stored collected samples.
**Study Design**	Longitudinal, multi-center study to assess progression of clinical features, imaging and biologic markers in Parkinson disease patients (de novo PD, Prodromal and subjects with specific genetic mutations) and healthy controls.
**Number of Subjects**	Approx 2000 Subjects Enrolled<br>400 Parkinson disease (PD)<br>70 SWEDD (subjects with PD symptoms but without evidence of dopaminergic deficit)<br>200 Healthy controls (HC)<br>100 Prodromal<br>600 Genetic Cohort<br>300 PD patients with a mutation (LRRK2, GBA or SNCA)<br>300 unaffected individuals with a mutation (LRRK2, GBA or SNCA) and/or a first degree relative with a mutation (LRRK2, GBA or SNCA)<br>600 Genetic registry<br>300 PD subjects with a mutation (LRRK2, GBA, or SNCA)

	300 unaffected subjects with a mutation (LRRK2, GBA, or SNCA) or with a first degree relative with a mutation (LRRK2, GBA, or SNCA)
**Main Eligibility Criteria**	**Parkinson Disease (PD) Subjects:**<br>Inclusion:<br>Patients must have at least two of the following: resting tremor, bradykinesia, rigidity (must have either resting tremor or bradykinesia); OR either asymmetric resting tremor or asymmetric bradykinesia.<br>A diagnosis of Parkinson disease for 2 years or less at Screening.<br>Hoehn and Yahr stage I or II at Baseline.<br>Confirmation from imaging core that screening dopamine transporter SPECT scan is consistent with dopamine transporter deficit (or for sites where DaTSCAN™ is not available, that VMAT-2 PET scan is consistent with VMAT deficit).<br>Not expected to require PD medication within at least 6 months from Baseline.<br>Male or female age 30 years or older at time of PD diagnosis.<br><br>Exclusion:<br>Currently taking levodopa, dopamine agonists, MAO-B inhibitors, amantadine or other PD medication.<br>Has taken levodopa, dopamine agonists, MAO-B inhibitors or amantadine within 60 days of Baseline.<br>Has taken levodopa or dopamine agonists prior to Baseline for more than a total of 60 days.<br>Received any of the following drugs that might interfere with dopamine transporter SPECT imaging: Neuroleptics, metoclopramide, alpha methyldopa, methylphenidate, reserpine, or amphetamine derivative, within 6 months of Screening.<br>Current treatment with anticoagulants (e.g., coumadin, heparin) that might preclude safe completion of the lumbar puncture.<br>Condition that precludes the safe performance of routine lumbar puncture, such as prohibitive lumbar spinal disease, bleeding diathesis, or clinically significant coagulopathy or thrombocytopenia.<br>Use of investigational drugs or devices within 60 days prior to Baseline (dietary supplements taken outside of a clinical trial are not exclusionary, e.g., coenzyme Q10).<br><br>**Healthy Control (HC) Subjects:**<br>Inclusion:<br>Male or female age 30 years or older at Screening.<br><br>Exclusion:<br>Current or active clinically significant neurological disorder (in the opinion of the Investigator).<br>First degree relative with idiopathic PD (parent, sibling, child).<br>MoCA score $\leq$ 26.<br>Received any of the following drugs that might interfere with dopamine transporter SPECT imaging: Neuroleptics, metoclopramide, alpha methyldopa,

	methylphenidate, reserpine, or amphetamine derivative, within 6 months of Screening.<br>Current treatment with anticoagulants (e.g. coumadin, heparin) that might preclude safe completion of the lumbar puncture.<br>Condition that precludes the safe performance of routine lumbar puncture, such as prohibitive lumbar spinal disease, bleeding diathesis, or clinically significant coagulopathy or thrombocytopenia.<br>Use of investigational drugs or devices within 60 days prior to baseline (dietary supplements taken outside of a clinical trial are not exclusionary, e.g., coenzyme Q10).<br><br>**SWEDD Subjects:**<br>Inclusion:<br>See Section 4.2.1 for Inclusion Criteria for PD subjects. All criteria apply except a SWEDD subject must have confirmation from imaging core that screening dopamine transporter SPECT scan shows no evidence of dopamine transporter deficit (or for sites where DaTSCAN™ is not available, that VMAT-2 PET scan shows no evidence of VMAT deficit).<br><br>Exclusion:<br>See Section 4.2.2 for Exclusion Criteria for PD subjects which also apply to SWEDD subjects.<br><br>**Prodromal Subjects:**<br>Inclusion:<br>See Section 4.2.7 for Inclusion Criteria. All subjects must demonstrate hyposmia, and/or RBD, plus must be eligible based on DaTSCAN assessment by imaging core.<br><br>Exclusion:<br>See Section 4.2.8 for Exclusion Criteria.<br><br>**Genetic Cohort: Parkinson Disease (PD) subjects**<br>Inclusion:<br>Patients must have at least two of the following: resting tremor, bradykinesia, rigidity (must have either resting tremor or bradykinesia); OR either asymmetric resting tremor or asymmetric bradykinesia.<br>A diagnosis of Parkinson disease for 7 years or less at Screening.<br>Hoehn and Yahr stage < 4 at Baseline.<br>Male or female age 18 years or older.<br>Willingness to undergo genetic testing and to be informed of genetic testing results<br>Confirmation of mutation in LRRK2, GBA or SNCA<br>For subjects taking any drugs that might interfere with dopamine transporter SPECT imaging (Neuroleptics, metoclopramide, alpha methyldopa, methylphenidate, reserpine, or amphetamine derivative) must be willing and able from a medical standpoint to hold the medication for at least 5 half-lives prior to screening DatSCAN™ imaging.

	Exclusion:<br>Current treatment with anticoagulants (e.g. coumadin, heparin) that might preclude safe completion of the lumbar puncture.<br>Condition that precludes the safe performance of routine lumbar puncture, such as prohibitive lumbar spinal disease, bleeding diathesis, or clinically significant coagulopathy or thrombocytopenia.<br><br>**Genetic Cohort: Unaffected Individuals**<br>Inclusion:<br>Male or female age 45 years or older at baseline with a LRRK2 or GBA mutation and/or a first degree relative with a LRRK2 or GBA mutation<br>OR<br>Male or female age 30 years or older at baseline with a SNCA mutation and/or a first degree relative with a SNCA mutation<br>Unaffected subjects at high risk of LRRK2, GBA or SNCA mutation due to first degree relative with a LRRK2, GBA or SNCA mutation may choose either to be informed of the results or remain unaware of the results.<br>Unaffected subjects from an ethnic or geographic group known to have relatively high risk of LRRK2, GBA or SNCA mutation (such as people of Ashkenazi Jewish or Basques descent) **and** who have a family member (either alive or deceased) who has/had PD must be willing to be informed of their own testing results.<br>Willingness to undergo genetic testing<br>For subjects taking any of the following drugs that might interfere with dopamine transporter SPECT imaging (Neuroleptics, metoclopramide, alpha methyldopa, methylphenidate, reserpine, or amphetamine derivative) must be willing and able from a medical standpoint to hold the medication for at least 5 half-lives prior to DatSCAN imaging.<br><br>Exclusion:<br>A clinical diagnosis of PD<br>Current treatment with anticoagulants (e.g. coumadin, heparin) that might preclude safe completion of the lumbar puncture.<br>Condition that precludes the safe performance of routine lumbar puncture, such as prohibitive lumbar spinal disease, bleeding diathesis, or clinically significant coagulopathy or thrombocytopenia.<br><br>**Genetic Registry:**<br>Inclusion:<br>Individual with a LRRK2, GBA or SNCA mutation and/or a first degree relative with a LRRK2, GBA or SNCA mutation<br>Male or female age 18 years or older<br>Willingness to undergo genetic testing, but may choose either to be informed of the results or remain unaware of the results

**Primary Outcome**	The primary study outcome is:<br>The mean rates of change and the variability around the mean of clinical, imaging and biomic outcomes in early PD patients, and where appropriate the comparison of these rates between PD patient subsets and between various subsets (including, but not limited to: PD vs. healthy subjects, PD vs. SWEDD, PD vs. prodromal, PD with and without LRRK2, GBA or SNCA mutation, unaffected LRRK2, GBA or SNCA mutation carriers vs. healthy controls) at study intervals ranging from 3 months to 36 months. Specific examples of outcomes include MDS-UPDRS, dopamine transporter imaging striatal uptake, vesicular monoamine transporter type-2 uptake, and serum and CSF alpha-synuclein**.** PD patient subsets may be defined by baseline assessments, genetic mutations, progression milestones and/or rate of clinical, imaging, or biomic change.
**Secondary Outcomes**	The secondary outcomes are:<br>Comparison between the rates of change in the mean of clinical, imaging and biomic outcomes in various subsets (including, but not limited to: early PD vs. healthy subjects, PD vs. SWEDD, PD vs. prodromal, PD with and without LRRK2, GBA or SNCA mutation, unaffected LRRK2, GBA or SNCA mutation vs. healthy controls) at study intervals ranging from 3 months to 156 months.<br>Prevalence of measures of clinical, imaging and biomic outcomes in various subsets (including, but not limited to: early PD patients, healthy subjects, PD vs SWEDDs, PD vs prodromal, PD with and without LRRK2, GBA or SNCA mutation, unaffected LRRK2, GBA or SNCA mutation carriers vs. healthy subjects)) at study intervals ranging from baseline to 156 months.<br>To establish the predictive value of early clinical non-motor features, baseline imaging and biomic outcomes for future course of disease.<br>To examine the proportion of SWEDD subjects that have a change in their clinical management at 24 months (SWEDD Clinical Diagnosis and Management Questionnaire).<br>Exploratory analysis to estimate the percentage of Prodromal subjects with one or more risk characteristics [hyposmia (<10th percentile for age and gender), RBD, or LRRK2, GBA or SNCA genetic mutation, and baseline DaTSCAN binding showing minimal to moderate DAT deficit] that phenoconvert to PD within 2 years, and an exploratory analysis to examine whether the baseline DaTSCAN binding or progression of clinical, imaging, or biospecimen markers may predict those subjects likely to phenoconvert.
**Safety Assessments**	Incidence of adverse events, proportion of withdrawals due to adverse events, vital signs and clinical laboratory assessment changes from baseline.
**Statistical Methods**	Changes from baseline to the one year, two year, three year, four year and five years evaluations will be calculated and summarized descriptively. We will calculate 95% confidence intervals for the mean rate of change and between subject variability, For this purpose the between subject variability will be estimated by fitting mixed models to all available data. Correlations will be calculated between the different measures, for example between change in total MDS-UPDRS and change in DAT uptake or alpha-synuclein levels.

**Data Access**	Data will be securely stored at central data coordinating facilities and will have all personally identifiable information removed before it is shared outside the study. All organizations responsible for data storage will observe the highest precautions to ensure data integrity and security. It is the goal of PPMI to enable timely access to the data by the PD research community.

Schedule of Activities- Parkinson's Disease (PD) Subjects [Please see modified Schedule of Activities on page 28 for visits post January 1, 2019 or post V15 (whichever is later)]

	Visit Number	SC	BL	V01	V02	V03	V04b	V05b	V06b	V07b	V08b	V09b	V10b	V11b	V12	V13	V14	V15	PW	ST	Unsch Visit
**Visit Description**	**Months (±30 days)**	**-45 day**	**0**	**3**	**6**	**9**	**12**	**18**	**24**	**30**	**36**	**42**	**48**	**54**	**60**	**72**	**84**	**96**	**---**	**---**	**---**
Written Informed Consent		X																			
Consent/Withdrawal of Consent for Future Procedures#		X	X	X	X	X	X	X	X	X	X	X	X	X	X	X	X	X	X	X	
Advance Directive/Review Continuing Ability to Consent #		X	X	X	X	X	X	X	X	X	X	X	X	X	X	X	X	X	X		X
Consent to Share Contact Information (FOUND in PPMI companion protocol)#		X					X	X	X	X	X	X	X	X	X	X	X	X	X	X	X
Consent to Share Contact Information with PPMI Brain and Tissue Bank/Advance Directive for PPMI Brain and Tissue Bank#†							X	X	X	X	X	X	X	X	X	X	X	X	X	X	X
Review Inclusion/Exclusion Criteria		X	X																		
Medical & Family History/Demographics		X																			
Physical Examination		X																			
Neurological Examination		X					X		X		X		X		X	X	X	X	X	Xk	Xg
Vital Signs		X	X[c]	X	X	X	X[c]	X	X[c]	X	X[c]	X	X[c]	X	X[c]	X[c]	X[c]	X[c]	X[c]	X	X
Blood Sample for DNA		X																			
Clinical Laboratory Assessments		X					X		X		X		X		X	X	X	X	X	Xk	Xg
Biomic blood sample			X[f]	X	X[f]	X	X[f]	X	X[f]	X	X[f]	X	X[f]	X	X[f]	X[f]	X[f]	X[f]	X[f]	X[f]	
PBMC blood sample			X		X			X		X		X		X							
MDS-UPDRS (including Hoehn & Yahr)h		X	X	X	X	X	X	X	X	X	X	X	X	X	X	X	X	X	X	X	
Modified Schwab & England ADL		X	X	X	X	X	X	X	X	X	X	X	X	X	X	X	X	X	X	X	
Clinical Diagnosis Assessment(s)		X					X		X		X		X		X	X	X	X	X	X	
MDS-UPDRS Repeat Part III/Hoehn & Yahr j							X		X		X		X		X	X	X	X	X		
Physical Activity Scale for the Elderly (PASE)							X		X		X		X		X	X	X	X	X		
Hopkins Verbal Learning Test – Revised			X				X		X		X		X		X	X	X	X	X	X[k]	
Benton Judgment of Line Orientation			X				X		X		X		X		X	X	X	X	X	X[k]	
Semantic Fluency			X				X		X		X		X		X	X	X	X	X	X[k]	
Letter Number Sequencing			X				X		X		X		X		X	X	X	X	X	X[k]	
Symbol Digit Modalities Test			X				X		X		X		X		X	X	X	X	X	X[k]	
Montreal Cognitive Assessment (MoCA)		X					X		X		X		X		X	X	X	X	X	X[k]	
Epworth Sleepiness Scale			X		X		X		X		X		X		X	X	X	X	X	X	
REM Sleep Behavior Questionnaire			X		X		X		X		X		X		X	X	X	X	X	X	
Geriatric Depression Scale (GDS-15)			X		X		X		X		X		X		X	X	X	X	X	X	
State-Trait Anxiety Inventory for Adults			X		X		X		X		X		X		X	X	X	X	X	X	
QUIP			X		X		X		X		X		X		X	X	X	X	X	X	
SCOPA-AUT			X		X		X		X		X		X		X	X	X	X	X	X	
Cognitive Categorization			X		X		X		X		X		X		X	X	X	X	X	X[k]	

Olfactory Testing (UPSIT)		X																		
MRI (structural)		X																		
MRI (DTI)e		X				X		X				X						^X		
DAT imaging[m,o]	X					X		X				X						^X	X[k]	
[18F] florbetaben PET imaging (see companion protocol)s								X		(X)		(X)		(X)	(X)	(X)	(X)	(X) +		
VMAT-2 imaging[m,o, r] (see companion protocol)	X[p]					X		X				X						^X	X[k]	
Lumbar puncture (CSF collection)		X		X		X		X		X		X		X	X	X	X	*X	X[k]	
Adverse Eventsa	X	X		X		X		X		X		X		X	X	X	X	X	X	
Current Medical Conditions Review		X	X	X	X	X	X	X	X	X	X	X	X	X	X	X	X	X	X	X
Concomitant Medication Review	X	X	X	X	X	X	X	X	X	X	X	X	X	X	X	X	X	X	X	X
Skin Biopsy t (see companion protocol)		X (+ follow up call within 7-10 days of biopsy)																		X
Blood draw[t]																				X
Sensor-PPMI activities (see companion protocol)	X																			

[a] Adverse events assessed at the visit and by phone 7 to 10 days following LP and/or DaTSCAN injection and following skin biopsy and/or [^{18}F] florbetaben PET imaging (for participating sites).
[b] Telephone consent will occur at Months 15, 21, 27, 33, 39, 45, 51 and 57.
[c] Height and weight also collected.
[e] Diffusion tensor MRI scan and resting state sequences conducted at selected sites.
[f] Biomic urine sample also collected.
[g] Conduct as clinically indicated – see protocol Sect. 5.3.18.
[h] Part IV once subject has started PD medication.
[j] Repeat assessment 1hr post treatment for subjects on levodopa or dopamine agonist.
[k] Not conducted depending on when ST visit completed – see protocol Sect. 5.3.20.
[m] DAT completed at all sites except Australia; VMAT completed in Australia and selected U.S. sites.

[†] Intent to Donate documentation completed with PPMI Brain and Tissue Bank study team or local PPMI site based on accessibility, local regulations and availability of infrastructure. For subjects who have not yet been approached concerning the Brain and Tissue Bank, please review subject interest in providing permission to forward their contact information to the PPMI Brain and Tissue Bank study team at their next visit (including interim visits). For subjects who may have already been approached concerning the Brain and Tissue Bank, but were undecided concerning participation or who have not yet signed an Intent to Donate form- please review Brain and Tissue Bank with subject at next annual visit (sites may also review this at interim visits if site deems appropriate).

[o] Urine (or serum) pregnancy test prior to injection for women of childbearing potential.
[p] Serum pregnancy test prior to injection day for women of childbearing potential; ECG for all subjects.
[r] Subjects enrolled in U.S. may agree to initial VMAT imaging scan at YR01 rather than Screening.[s] [^{18}F]
[s]florbetaben PET imaging at selected sites (see companion protocol) at either Month 24, 36, 48, 60, 72, 84 or 96[t] Skin biopsy for participating sites completed at any routine or unscheduled visit post amendment approval; for sites collecting blood samples as part of companion study, blood draw completed at unscheduled visit post amendment approval.
[t] Skin biopsy for participating sites completed at any routine or unscheduled visit post amendment approval; for sites collecting blood samples as part of companion study, blood draw completed at unscheduled visit or interim visit post amendment approval. (sites completing collection of blood sample at interim visit must forego collection of PBMCs and biomic blood samples at interim visit)

ST = Symptomatic Therapy
PW = Premature Withdrawal (*if not done in last 3 mths; ^if not done in last 12 mths; [+]if subject has not completed scan and is willing to complete at PW)
[#] For active subjects, begin at next visit post amendment approval and consent

Schedule of Activities – Healthy Control (HC) Subjects [Please see modified Schedule of Activities on page 28 for visits post January 1, 2019 or post V15 (whichever is later)**]**

	**Visit Number**	**SC**	**BL**	**V01**	**V02**	**V03**	**V04[b]**	**V05[b]**	**V06[b]**	**V07[b]**	**V08[b]**	**V09[b]**	**V10[b]**	**V11[b]**	**V12**	**V13**	**V14**	**V15**	**PW**	**Unsch. Visit**
**Visit Description**	**Months (±30 days)**	**-45 days**	**0**	**3**	**6**	**9**	**12**	**18**	**24**	**30**	**36**	**42**	**48**	**54**	**60**	**72**	**84**	**96**	**---**	**---**
Written Informed Consent		X																		
Consent/Withdrawal of Consent for Future Procedures[#]		X	X	X	X	X	X	X	X	X	X	X	X	X	X	X	X	X	X	
Advance Directive/Review Continuing Ability to Consent[#]		X	X	X	X	X	X	X	X	X	X	X	X	X	X	X	X	X	X	X
Consent to Share Contact Information (FOUND in PPMI companion protocol)[#]		X					X	X	X	X	X	X	X	X	X	X	X	X	X	X
Consent to Share Contact Information with PPMI Brain and Tissue Bank/Advance Directive for PPMI Brain and Tissue Bank[# †]							X	X	X	X	X	X	X	X	X	X	X	X	X	X
Review Inclusion/Exclusion Criteria		X	X																	
Medical & Family History/Demographics		X																		
Physical Examination		X																		
Neurological Examination		X					X		X		X		X		X	X	X	X	X	X[g]
Vital Signs		X	X[c]	X	X	X	X[c]	X	X[c]	X	X[c]	X	X[c]	X	X[c]	X	X[c]	X	X[c]	X
Blood Sample for DNA		X																		
Clinical Laboratory Assessments		X					X		X		X		X		X	X	X	X	X	X[g]
Biomic blood sample			X[f]	X	X[f]	X	X[f]	X	X[f]	X	X[f]	X	X[f]	X	X[f]	X[f]	X[f]	X[f]	*X[f]	
PBMC blood sample			X		X			X		X		X		X						
MDS-UPDRS (including Hoehn & Yahr)			X				X		X		X		X		X	X	X	X	X	
Clinical Diagnosis Assessment(s)		X					X		X		X		X		X	X	X	X	X	
Physical Activity Scale for the Elderly (PASE)							X		X		X		X		X	X	X	X	X	
Hopkins Verbal Learning Test – Revised			X				X		X		X		X		X	X	X	X	X	
Benton Judgment of Line Orientation			X				X		X		X		X		X	X	X	X	X	
Semantic Fluency			X				X		X		X		X		X	X	X	X	X	
Letter Number Sequencing			X				X		X		X		X		X	X	X	X	X	
Symbol Digit Modalities Test			X				X		X		X		X		X	X	X	X	X	
Montreal Cognitive Assessment (MoCA)		X					X		X		X		X		X	X	X	X	X	
Epworth Sleepiness Scale			X				X		X		X		X		X	X	X	X	X	
REM Sleep Behavior Questionnaire			X				X		X		X		X		X	X	X	X	X	
Geriatric Depression Scale (GDS-15)			X				X		X		X		X		X	X	X	X	X	
State-Trait Anxiety Inventory for Adults			X				X		X		X		X		X	X	X	X	X	
QUIP			X				X		X		X		X		X	X	X	X	X	
SCOPA-AUT			X				X		X		X		X		X	X	X	X	X	
Cognitive Categorization			X				X		X		X		X		X	X	X	X	X	
Olfactory Testing (UPSIT)			X																	

MRI (structural)		X																	
MRI (DTI)[e]		X				X		(X[e])		(X[e])		(X[e])		(X[e])				^X	
DAT imaging[m,o]	X																		
[18F] florbetaben PET imaging (see companion protocol)[s]								X		(X)		(X)		(X)		(X)		(X)[+]	
VMAT-2 imaging[m,o, r]	X[p]																		
Lumbar puncture (CSF collection)		X		X		X		X		X		X		X	X	X	X	*X	
Adverse Events[a]	X	X		X		X		X		X		X		X	X	X	X	X	
Current Medical Conditions Review		X	X	X	X	X	X	X	X	X	X	X	X	X	X	X	X	X	X
Concomitant Medication Review	X	X	X	X	X	X	X	X	X	X	X	X	X	X	X	X	X	X	X
Skin biopsy[t] (see companion protocol)		X (+ follow up call within 7-10 days of biopsy)																	X
Blood draw[t]																			X
Sensor-PPMI activities (see companion protocol)	X																		

[a] Adverse events assessed at the visit and by phone 7 to 10 days following LP and/or DaTSCAN injection and following skin biopsy and/or [18F] florbetaben PET imaging (for participating sites).
[b] Telephone consent will occur at Months 15, 21, 27, 33, 39, 45, 51 and 57.
[c] Height and weight also collected.
[e] Diffusion tensor MRI scan and resting state sequences conducted at selected sites.
[f] Biomic urine sample also collected.
[g] Conduct as clinically indicated – see protocol Sect. 5.3.18.
[h] Part IV once subject has started PD medication.
[j] Repeat assessment 1hr post treatment for subjects on levodopa or dopamine agonist.
[k] Not conducted depending on when ST visit completed – see protocol Sect. 5.3.20.
[m] DAT completed at all sites except Australia; VMAT completed in Australia and selected U.S. sites.

[o] Urine (or serum) pregnancy test prior to injection for women of childbearing potential.
[p] Serum pregnancy test prior to injection day for women of childbearing potential.
[r] Subjects enrolled in U.S. may agree to initial VMAT imaging scan at YR01 rather than Screening.
[s] [18F] florbetaben PET imaging at selected sites (see companion protocol) at either Month 24, 36, 48, or 60 (what about month 84?)
[t] Skin biopsy for participating sites completed at any routine or unscheduled visit post amendment approval; for sites collecting blood samples as part of companion study, blood draw completed at unscheduled visit or interim visit post amendment approval. (sites completing collection of blood sample at interim visit must forego collection of PBMCs and biomic blood samples at interim visit)
PV= Phone Visit
PW = Premature Withdrawal (*if not done in last 3 mths; ^ only if withdrawal within first 12 mths and MRI DTI not done in last 6 mths)

[#] For active subjects, begin at next visit post amendment approval and consent
[†] Intent to Donate documentation completed with PPMI Brain and Tissue Bank study team or local PPMI site based on accessibility, local regulations and availability of infrastructure.

Schedule of Activities – SWEDD Subjects

									For SWEDDS continuing or returning[s]											
**Visit Number**	**Re-SC**	**BL**	**V01**	**V02**	**V03**	**V04[b]**	**V05[b]**	**V06**	**[b]V07[b]**	**V08[b]**	**V09[b]**	**V10[b]**	**V11[b]**	**V12**	**V13**	**V14**	**V15**	**PW**	**ST**	**Unsch. Visit**
**Visit Description** / **Months (±30 days)**	**-45 day**	**0**	**3**	**6**	**9**	**12**	**18**	**24**	**30**	**36**	**42**	**48**	**54**	**60**	**72**	**84**	**96**	**---**	**---**	**---**
Written Informed Consent	X								X[u]											
Consent/Withdrawal of Consent for Future Procedures[#]	X	X	X	X	X	X	X	X	X	X	X	X	X	X	X	X	X	X	X	
Advance Directive/Review Continuing Ability to Consent[#]	X	X	X	X	X	X	X	X	X	X	X	X	X	X	X	X	X	X	X	X
Consent to Share Contact Information (FOUND in PPMI companion protocol)[#]	X					X		X	X	X	X	X	X	X	X	X	X	X	X	X
Consent to Share Contact Information with PPMI Brain and Tissue Bank /Advance Directive for PPMI Brain and Tissue Bank [#†]						X	X	X	X	X	X	X	X	X	X	X	X	X	X	X
Review Inclusion/Exclusion Criteria	X	X																		
Medical and Family History/Demographics	X																			
Physical Examination	X																			
Neurological Examination	X					X		X		X		X		X	X	X	X	X	X[k]	X[g]
Vital Signs	X	X[c]	X	X	X	X[c]	X	X[c]	X	X[c]	X	X[c]	X	X[c]	X[c]	X[c]	X[c]	X[c]	X	X
Clinical Laboratory Assessments	X					X		X		X		X		X	X	X	X	X	X[k]	X[g]
Biomic blood sample		X[f]	X	X[f]	X	X[f]	X	X[f]	X	X[f]	X	X[f]	X	X[f]	X[f]	X[f]	X[f]	*X[f]	X[f]	
Lumbar puncture (CSF collection)		X		X		X		X		X		X		X	X[v]	X[v]	X[v]	*X	X[k]	
MDS-UPDRS (including Hoehn & Yahr) [h]	X	X	X	X	X	X	X	X	X	X	X	X	X	X	X	X	X	X	X	
Modified Schwab & England ADL	X	X	X	X	X	X	X	X	X	X	X	X	X	X	X	X	X	X	X	
MDS-UPDRS Repeat Part III / Hoehn & Yahr[j]						X		X		X		X		X	X	X	X	X		
Clinical Diagnosis and Management Questionnaire		X	X	X	X	X	X	X										X	X	
Clinical Diagnosis Assessment(s)										X		X		X	X	X	X	X	X	
Physical Activity Scale for the Elderly (PASE)						X		X		X		X		X	X	X	X	X		
Hopkins Verbal Learning Test – Revised		X				X		X		X		X		X	X	X	X	X	X	
Benton Judgment of Line Orientation		X				X		X		X		X		X	X	X	X	X	X[k]	
Semantic Fluency		X				X		X		X		X		X	X	X	X	X	X[k]	
Letter Number Sequencing		X				X		X		X		X		X	X	X	X	X	X[k]	
Symbol Digit Modalities Test		X				X		X		X		X		X	X	X	X	X	X[k]	
Montreal Cognitive Assessment (MoCA)	X					X		X		X		X		X	X	X	X	X	X[k]	
Epworth Sleepiness Scale		X		X		X		X		X		X		X	X	X	X	X	X	
REM Sleep Behavior Questionnaire		X		X		X		X		X		X		X	X	X	X	X	X	
Geriatric Depression Scale (GDS-15)		X		X		X		X		X		X		X	X	X	x	X	X	
State-Trait Anxiety Inventory for Adults		X		X		X		X		X		X		X	X	X	X	X	X	
QUIP		X		X		X		X		X		X		X	X	X	X	X	X	
SCOPA-AUT		X		X		X		X		X		X		X	X	X	x	X	X	
Cognitive Categorization		X		X		X		X		X		X		X	X	X	X	X	X[k]	
Olfactory Testing (UPSIT)		X																		
MRI (structural)		X																		
MRI (DTI)[e]		X						X				X						^X		

DAT imaging[m,o]								X				X						^X	X[k]	
VMAT-2 imaging[m,o, r]								X				X						^X	X[k]	
Adverse Events[a]		X		X		X		X		X		X		X	X	X	X	X	X	
Current Medical Conditions and Concomitant Medication Review	X	X	X	X	X	X	X	X	X	X	X	X	X	X	X	X	X	X	X	X

[a] Adverse events assessed at the visit and by phone 7 to 10 days following LP and/or DaTSCAN injection.
[b] Telephone contact will occur at Months 15 and 21 (for SWEDDS post V06, also at Months 27 , 33, 39, 45, 51 and 57).
[c] Height and weight also collected.
[e] Diffusion tensor MRI scan and resting state sequences conducted at selected sites.
[f] Biomic urine sample also collected.
[g] Conduct as clinically indicated – see protocol Sect. 5.3.18.
[h] Part IV once subject has started PD medication.
[j] Repeat assessment 1hr post treatment for subjects on levodopa or dopamine agonist.

[†] For subjects who have not yet been approached concerning the Brain and Tissue Bank, please review subject interest in providing permission to forward their contact information to the PPMI Brain and Tissue Bank study team at their next visit (including interim visits). For subjects who may have already been approached concerning the Brain and Tissue Bank, but were undecided concerning participation or who have not yet signed an Intent to Donate form- please review Brain and Tissue Bank with subject at next annual visit (sites may also review this at interim visits if site deems appropriate).

[k] Not conducted depending on when ST visit completed – see protocol Sect. 5.3.20.
[m] DAT completed in all sites except Australia; VMAT completed in Australia and selected U.S. sites.
[o] Urine (or serum) pregnancy test prior to injection for women of childbearing potential.
[r] Subjects enrolled in U.S. may agree to initial VMAT imaging scan at YR01 rather than Screening.
[s] For SWEDDs who will continue (or return to) follow up after Month 24.
[u] Subjects should be re-consented before continuing

ST = Symptomatic Therapy
PW = Premature Withdrawal (*if not done in last 3 mths; ^ if not done in last 12 mths)
# For active subjects, begin at next visit post amendment approval and consent

Schedule of Activities – PRODROMAL Subjects [Please see modified Schedule of Activities on page 30 for post month 60 visits]

**Visit Number**		**Pre SC**	**SC**	**BL**	**V01**	**V02**	**V03**	**V04[b]**	**V05[b]**	**V06[b]**	**V07[b]**	**V08[b]**	**V09[b]**	**V10[b]**	**V11**	**V12**	**PW**	**ST**	**Unsch. Visit**
**Visit Description**	**Months (±30 days)**	**-6 mths**	**-45 days**	**0**	**3**	**6**	**9**	**12**	**18**	**24**	**30**	**36**	**42**	**48**	**54**	**60**	**---**	**---**	**---**
Written Informed Consent		X	X																
Consent/Withdrawal of Consent for Future Procedures[#]		X	X	X	X	X	X	X	X	X	X	X	X	X	X	X	X	X	
Advance Directive/Review Continuing Ability to Consent[#]			X	X	X	X	X	X	X	X	X	X	X	X	X	X	X	X	X
Consent to Share Contact Information (FOUND in PPMI companion protocol)[#]			X			X	X	X	X	X	X	X	X	X	X	X	X	X	X
Consent to Share Contact Information with PPMI Brain and Tissue Bank /Advance Directive for PPMI Brain and Tissue Bank [#] [†]								X	X	X	X	X	X	X	X	X	X	X	X
Review of Inclusion/Exclusion Criteria		X	X	X															
Review of PSG [p]		X																	
Self Reporting Questionnaire*		X																	
Olfactory Testing (UPSIT)		X		X								X[>]							
Medical & Family History/Demographics			X																
Physical Examination			X																
Neurological Examination			X					X		X		X		X		X	X	X[k]	X[g]
Vital Signs			X	X[c]	X	X	X	X[c]	X	X[c]	X	X[c]	X	X[c]	X	X[c]	X[c]	X	X
Blood Sample for DNA				X															
Clinical Laboratory Assessments			X					X		X		X		X		X	X	X[k]	X[g]
Biomic blood sample				X[f]	X	X[f]	X	X[f]	X	X[f]	X	X[f]	X	X[f]	X	X[f]	*X[f]	X[f]	
PBMC blood sample				X		X			X		X		X		X				
MDS-UPDRS (including Hoehn & Yahr)[h]			X	X	X	X	X	X	X	X	X	X	X	X	X	X	X	X	
Modified Schwab & England ADL			X	X	X	X	X	X	X	X	X	X	X	X	X	X	X	X	
MDS-UPDRS Repeat Part III/Hoehn & Yahr[j]								X		X		X		X			X		
Clinical Diagnosis Assessments			X	X	X	X	X	X	X	X	X	X	X	X			X	X	
Physical Activity Scale for the Elderly (PASE)				X				X		X		X		X		X	X		
Hopkins Verbal Learning Test – Revised				X				X		X		X		X		X	X	X[k]	
Benton Judgment of Line Orientation				X				X		X		X		X		X	X	X[k]	
Semantic Fluency				X				X		X		X		X		X	X	X[k]	
Letter Number Sequencing				X				X		X		X		X		X	X	X[k]	
Symbol Digit Modalities Test				X				X		X		X		X		X	X	X[k]	
Montreal Cognitive Assessment (MoCA)			X					X		X		X		X		X	X	X[k]	
Epworth Sleepiness Scale				X		X		X		X		X		X		X	X	X	
REM Sleep Behavior Questionnaire				X		X		X		X		X		X		X	X	X	
Geriatric Depression Scale (GDS-15)			X			X		X		X		X		X		X	X	X	
State-Trait Anxiety Inventory for Adults			X			X		X		X		X		X		X	X	X	
QUIP				X		X		X		X		X		X		X	X	X	
SCOPA-AUT				X		X		X		X		X		X		X	X	X	
Cognitive Categorization				X		X		X		X		X		X		X	X	X[k]	
Lumbar puncture (CSF collection)				X		X		X		X		X		X		X	*X	X[k]	
MRI (structural)				X															
MRI (DTI)[e]				X				X		X							^X		

DAT imaging[o]		X					X		X		X		X			^X	X[k]	
VMAT-2 imaging[m,o,] (see companion protocol)		X[q]					X		X							^X	X[k]	
[^{18}F] florbetaben PET imaging (see companion protocol)[s]							X		(X)		(X)		(X)			(X)+		
Adverse Events[a]		X	X		X		X		X		X		X		X	X	X	
Current Medical Conditions Review			X	X	X	X	X	X	X	X	X	X	X	X	X	X	X	X
Concomitant Medication Review		X	X	X	X	X	X	X	X	X	X	X	X	X	X	X	X	X
Skin Biopsy[t] (see companion protocol)		X (+ follow up call within 7-10 days of biopsy)																X
Blood draw[t]																		X
Sensor-PPMI activities (see companion protocol)			X															

Schedule of Activities – Prodromal Subjects

[a] Adverse events assessed at the visit and by phone 7 to 10 days following LP and/or DaTSCAN injection and following [^{18}F] florbetaben PET imaging (for participating sites).
[b] Telephone contact will occur at Months 15, 21, 27, 33, 39, 45, 51 and 57.
[c] Height and weight also collected.
[e] Diffusion tensor MRI scan and resting state sequences conducted at selected sites.
[f] Biomic urine sample also collected.
[g] Conduct as clinically indicated – see protocol Sect. 5.3.18.
[h] Part IV once subject has started PD medication.
[j] Repeat assessment 1hr post treatment for subjects on levodopa or dopamine agonist.
[k] Not conducted depending on when ST visit completed – see protocol Sect. 5.3.20.

[†] Intent to Donate documentation completed with PPMI Brain and Tissue Bank study team or local PPMI site based on accessibility, local regulations and availability of infrastructure. For subjects who have not yet been approached concerning the Brain and Tissue Bank, please review subject interest in providing permission to forward their contact information to the PPMI Brain and Tissue Bank study team at their next visit (including interim visits). For subjects who may have already been approached concerning the Brain and Tissue Bank, but were undecided concerning participation or who have not yet signed an Intent to Donate form- please review Brain and Tissue Bank with subject at next annual visit (sites may also review this at interim visits if site deems appropriate).

[m] DAT completed in all sites except Australia; VMAT completed in Australia and selected U.S. sites.
[o] Urine (or serum) pregnancy test prior to injection for women of childbearing potential.
[p] Completed based on recruitment method
[q] Serum pregnancy test prior to injection day for women of childbearing potential.
[s] [^{18}F] florbetaben PET imaging at selected sites (see companion protocol) at either Months 12, 24, 36, or 48
[t] Skin biopsy for participating sites completed at any routine or unscheduled visit post amendment approval; for sites collecting blood samples as part of companion study, blood draw completed at unscheduled visit or interim visit post amendment approval. (sites completing collection of blood sample at interim visit must forego collection of PBMCs and biomic blood samples at interim visit)

ST = Symptomatic Therapy;
PW = Premature Withdrawal (*if not done in last 3 mths; ^if not done in last 12 mths; +if subject has not completed scan and is willing to complete at PW visit)

[#] For active subjects, begin at next visit post amendment approval and consent
- ➢ Perform at month 36 visit or later visit if subject has already completed month 36 visit.

Schedule of Activities – Genetic Cohort: PD subjects [Please see modified Schedule of Activities on page 28 for post month 60 visits]

Visit Number	SC	BL	V02	V04[b]	V05[b]	V06[b]	V07[b]	V08[b]	V09[b]	V10[b]	V11[b]	V12	PW	ST	Unsch. Visit
**Visit Description** / **Months (±30 days)**	**-45 days**	**0**	**6**	**12**	**18**	**24**	**30**	**36**	**42**	**48**	**54**	**60**	**---**	**---**	**---**
Written Informed Consent	X														
Consent/Withdrawal of Consent for Future Procedures[#]	X	X	X	X	X	X	X	X	X	X	X	X	X	X	
Advance Directive/Review Continuing Ability to Consent[#]	X	X	X	X	X	X	X	X	X	X	X	X	X		X
Consent to Share Contact Information (FOUND in PPMI companion protocol)[#]	X	X	X	X	X	X	X	X	X	X	X	X	X	X	X
Consent to Share Contact Information with PPMI Brain and Tissue Bank/Advance Directive for PPMI Brain and Tissue Bank [#†]	X		X	X	X	X	X	X	X	X	X	X	X	X	X
Consent/Withdrawal of Consent for Future Contact	X	X	X	X	X	X	X	X	X	X	X	X	X	X	X
Review Inclusion/Exclusion Criteria	X	X													
Medical History/Demographics	X														
Family History of PD Review	X	X	X	X	X	X	X	X	X	X	X	X	X	X	
Family History Substudy (see companion protocol)[#]		X													
Physical Examination	X														
Neurological Examination	X			X		X		X		X		X	X	X[k]	X[g]
Vital Signs	X	X[c]	X	X[c]	X	X[c]	X	X[c]	X	X[c]	X	X[c]	X[c]	X	X
Blood Sample for DNA[@]	X														
Clinical Laboratory Assessments	X			X		X		X		X		X	X	X[k]	X[g]
Biomic blood sample		X[f]	X[f]	X[f]	X	X[f]	X	X[f]	X	X[f]	X	X[f]	*X[f]	X[f]	
PBMC blood sample		X	X		X		X		X		X				
MDS-UPDRS (including Hoehn & Yahr)[h]	X	X	X	X	X	X	X	X	X	X	X	X	X	X	
Modified Schwab & England ADL	X	X	X	X	X	X	X	X	X	X	X	X	X	X	
Clinical Diagnosis Assessment(s)	X		X	X	X	X	X	X	X	X	X	X	X	X	
MDS-UPDRS Repeat Part III/Hoehn & Yahr[j]		X		X		X		X		X		X	X		
Physical Activity Scale for the Elderly (PASE)		X		X		X		X		X		X	X		
Hopkins Verbal Learning Test – Revised		X		X		X		X		X		X	X	X[k]	
Benton Judgment of Line Orientation		X		X		X		X		X		X	X	X[k]	
Semantic Fluency		X		X		X		X		X		X	X	X[k]	
Letter Number Sequencing		X		X		X		X		X		X	X	X[k]	
Symbol Digit Modalities Test		X		X		X		X		X		X	X	X[k]	
Montreal Cognitive Assessment (MoCA)	X			X		X		X		X		X	X	X[k]	
Epworth Sleepiness Scale		X	X	X		X		X		X		X	X	X	
REM Sleep Behavior Questionnaire		X	X	X		X		X		X		X	X	X	
Geriatric Depression Scale (GDS-15)		X	X	X		X		X		X		X	X	X	
State-Trait Anxiety Inventory for Adults		X	X	X		X		X		X		X	X	X	
QUIP		X	X	X		X		X		X		X	X	X	
SCOPA-AUT		X	X	X		X		X		X		X	X	X	
Cognitive Categorization		X	X	X		X		X		X		X	X	X[k]	
Olfactory Testing (UPSIT)		X													
MRI (structural)		X													
MRI (DTI)[e]		X				X				X			^X		
DAT imaging[m,o]	X					X				X			^X	X[k]	
VMAT-2 imaging[m,o, r] (see companion protocol)	X[p]					X				X			^X	X[k]	

Lumbar puncture (CSF collection)		X		X		X		X		X		X	*X	X[k]	
[^{18}F] florbetaben PET imaging (see companion protocol)[s]				X		(X)		(X)		(X)		(X)	(X)+		
Adverse Events[a]	X	X		X		X		X		X		X	X	X	
Current Medical Conditions Review		X	X	X	X	X	X	X	X	X	X	X	X	X	X
Concomitant Medication Review	X	X	X	X	X	X	X	X	X	X	X	X	X	X	X
Skin biopsy [t] (see companion protocol)		X (+ follow up call within 7-10 days of biopsy)													X
Blood draw[t]															X
Gait assessments (see companion protocol)[x]		(X)		(X)		(X)		(X)		(X)		(X)			
Sensor-PPMI activities (see companion protocol)	X														

Schedule of Activities – Genetic Cohort (PD)

[a] Adverse events assessed at the visit and by phone 7 to 10 days following LP and/or DaTSCAN injection and following skin biopsy and/or [^{18}F] florbetaben PET imaging (for participating sites).
[b] Telephone contact will occur at Months 15, 21, 27, 33, 39, 45, 51 and 57.
[c] Height and weight also collected.
[e] Diffusion tensor MRI scan and resting state sequences conducted at selected sites.
[f] Biomic urine sample also collected.
[g] Conduct as clinically indicated – see protocol Sect. 5.3.18.
[h] Part IV once subject has started PD medication.
[j] Repeat assessment 1hr post treatment for subjects on levodopa or dopamine agonist.
[k] Not conducted depending on when ST visit completed – see protocol Sect. 5.3.20.
[m] DAT completed at all sites except Australia; VMAT completed in Australia and selected U.S. sites.
[o] Urine (or serum) pregnancy test prior to injection for women of childbearing potential.
[p] Serum pregnancy test prior to injection day for women of childbearing potential; ECG for all subjects.
[r] Subjects enrolled in U.S. may agree to initial VMAT imaging scan at YR01 rather than Screening.

[†] Intent to Donate documentation completed with PPMI Brain and Tissue Bank study team or local PPMI site based on accessibility, local regulations and availability of infrastructure. For subjects who have not yet been approached concerning the Brain and Tissue Bank, please review subject interest in providing permission to forward their contact information to the PPMI Brain and Tissue Bank study team at their next visit (including interim visits). For subjects who may have already been approached concerning the Brain and Tissue Bank, but were undecided concerning participation or who have not yet signed an Intent to Donate form- please review Brain and Tissue Bank with subject at next annual visit (sites may also review this at interim visits if site deems appropriate).

[s] [^{18}F] florbetaben PET imaging at selected sites (see companion protocol) at either Months 12, 24,or 36, 48, or 60
[t] Skin biopsy for participating sites completed at any routine or unscheduled visit post-Amendment 8 approval. Genetic cohort subjects must be positive for LRRK2, GBA or SNCA mutation. For sites collecting blood samples as part of companion study, blood draw completed at unscheduled visit or interim visit post amendment approval. (sites completing collection of blood sample at interim visit must forego collection of PBMCs and biomic blood samples at interim visit)
x Gait assessments at selected sites (see companion protocol); two gait assessments completed- first gait assessment completed at annual visit during which subject consents to gait assessment protocol; second gait assessment completed during subsequent regularly scheduled annual visit
ST = Symptomatic Therapy
PW = Premature Withdrawal (*if not done in last 3 mths; ^if not done in last 12 mths; +if subject has not completed scan and is willing to complete at PW)
[@] May be done at BL if not at SC

For active subjects, begin at next visit post amendment approval and consent

Schedule of Activities – Genetic Cohort: Unaffected Subjects [Please see modified Schedule of Activities on page 30 for post month 60 visits]

**Visit Number**	**SC**	**BL**	**V02**	**V04[b]**	**V05[b]**	**V06[b]**	**V07[b]**	**V08[b]**	**V09[b]**	**V10[b]**	**V11[b]**	**V12**	**PW**	**Unsch. Vist**
**Visit Description** — **Months (±30 days)**	**-45 days**	**0**	**6**	**12**	**18**	**24**	**30**	**36**	**42**	**48**	**54**	**60**	**---**	**---**
Written Informed Consent	X													
Consent/Withdrawal of Consent for Future Contact#	X	X	X	X	X	X	X	X	X	X	X	X	X	
Advance Directive/ Review Continuing Ability to Consent[#]	X	X	X	X	X	X	X	X	X	X	X	X	X	X
Consent to Share Contact Information (FOUND in PPMI companion protocol)[#]	X	X	X`	X	X	X	X	X	X	X	X	X	X	X
Consent to Share Contact Information with PPMI Brain and Tissue Bank/Advance Directive for PPMI Brain and Tissue Bank [#†]	X		X	X	X	X	X	X	X	X	X	X	X	X
Review Inclusion/Exclusion Criteria	X	X												
Medical History/Demographics	X													
Family History of PD Review	X	X	X	X	X	X	X	X	X	X	X	X	X	
Family History Substudy (see companion protocol)[#]		X												
Physical Examination	X													
Neurological Examination	X			X		X		X		X		X	X	X[g]
Vital Signs	X	X[c]	X	X[c]	X	X[c]	X	X[c]	X	X[c]	X	X[c]	X[c]	X
Blood Sample for DNA[@]	X													
Clinical Laboratory Assessments	X			X		X		X		X		X	X	X[g]
Biomic blood sample		X[f]	X[f]	X[f]	X	X[f]	X	X[f]	X	X[f]	X	X[f]	*X[f]	
PBMC blood sample		X	X		X		X		X		X			
MDS-UPDRS (including Hoehn & Yahr) [h]	X	X	X	X	X	X	X	X	X	X	X	X	X	
Modified Schwab & England ADL	X	X	X	X	X	X	X	X	X	X	X	X	X	
Clinical Diagnosis Assessment(s)	X		X	X	X	X	X	X	X	X	X	X	X	
MDS-UPDRS Repeat Part III/Hoehn & Yahr[j]		X		X		X		X		X		X	X	
Physical Activity Scale for the Elderly (PASE)		X		X		X		X		X		X	X	
Hopkins Verbal Learning Test – Revised		X		X		X		X		X		X	X	
Benton Judgment of Line Orientation		X		X		X		X		X		X	X	
Semantic Fluency		X		X		X		X		X		X	X	
Letter Number Sequencing		X		X		X		X		X		X	X	
Symbol Digit Modalities Test		X		X		X		X		X		X	X	
Montreal Cognitive Assessment (MoCA)	X			X		X		X		X		X	X	
Epworth Sleepiness Scale		X	X	X		X		X		X		X	X	
REM Sleep Behavior Questionnaire		X	X	X		X		X		X		X	X	
Geriatric Depression Scale (GDS-15)		X	X	X		X		X		X		X	X	
State-Trait Anxiety Inventory for Adults		X	X	X		X		X		X		X	X	
QUIP		X	X	X		X		X		X		X	X	
SCOPA-AUT		X	X	X		X		X		X		X	X	
Cognitive Categorization		X	X	X		X		X		X		X	X	
Olfactory Testing (UPSIT)		X						X[>]				X		
MRI (structural)		X												
MRI (DTI)[e]		X				X				X			^X	

DAT imaging[m,o]	X					X				X			^X	
VMAT-2 imaging[m,o, r] (see companion protocol)	X[p]					X				X			^X	
Lumbar puncture (CSF collection)		X		X		X		X		X		X	*X	
[18F] florbetaben PET imaging (see companion protocol)[s]				X		(X)		(X)		(X)		(X)	(X)+	
Adverse Events[a]	X	X		X		X		X		X		X	X	
Current Medical Conditions Review		X	X	X	X	X	X	X	X	X	X	X	X	X
Concomitant Medication Review	X	X	X	X	X	X	X	X	X	X	X	X	X	X
Skin Biopsy [t] (see companion protocol)		X (+ follow up call within 7-10 days of biopsy)												X
Blood draw[t]														X
Gait assessments (see companion protocol)[x]		(X)		(X)		(X)		(X)		(X)		(X)		

<u>Schedule of Activities –Genetic Cohort: Unaffected Subjects</u>

[a] Adverse events assessed at the visit and by phone 7 to 10 days following LP and/or DaTSCAN injection and following skin biopsy and/or [18F] florbetaben PET imaging (for participating sites).
[b] Telephone contact will occur at Months 15, 21, 27, 33, 39, 45, 51 and 57.
[c] Height and weight also collected.
[e] Diffusion tensor MRI scan and resting state sequences conducted at selected sites.
[f] Biomic urine sample also collected.
[g] Conduct as clinically indicated – see protocol Sect. 5.3.18.
[h] Part IV once subject has started PD medication
[j] Repeat assessment 1hr post treatment for subjects on levodopa or dopamine agonist.
[k] Not conducted depending on when ST visit completed – see protocol Sect. 5.3.20.
[m] DAT completed at all sites except Australia; VMAT completed in Australia and selected U.S. sites.
[o] Urine (or serum) pregnancy test prior to injection for women of childbearing potential.
[p] Serum pregnancy test prior to injection day for women of childbearing potential; ECG for all subjects.
[r] Subjects enrolled in U.S. may agree to initial VMAT imaging scan at YR01 rather than Screening.
[@] May be done at BL if not at SC
[s] [18F] florbetaben PET imaging at selected sites (see companion protocol) at either Months 12, 24, 36, 48, or 60
[t] Skin biopsy for participating sites completed at any routine or unscheduled visit post-amendment approval; for sites collecting blood samples as part of companion study, blood draw completed at unscheduled visit or interim visit post amendment approval. (sites completing collection of blood sample at interim visit must forego collection of PBMCs and biomic blood samples at interim visit)
[x] Gait assessments at selected sites (see companion protocol); two gait assessments completed- first gait assessment completed at annual visit during which subject consents to gait assessment protocol; second gait assessment completed during subsequent regularly scheduled annual visit

PW = Premature Withdrawal (*if not done in last 3 mths; ^if not done in last 12 mth; +if subject has not completed scan and is willing to complete at PW)

For active subjects, begin at next visit post amendment approval and consent
➢ Perform at month 36 visit or later visit if subject has already completed month 36 visit.

[†] Intent to Donate documentation completed with PPMI Brain and Tissue Bank study team or local PPMI site based on accessibility, local regulations and availability of infrastructure. For subjects who have not yet been approached concerning the Brain and Tissue Bank, please review subject interest in providing permission to forward their contact information to the PPMI Brain and Tissue Bank study team at their next visit (including interim visits). For subjects who may have already been approached concerning the Brain and Tissue Bank, but were undecided concerning participation or who have not yet signed an Intent to Donate form- please review Brain and Tissue Bank with subject at next annual visit (sites may also review this at interim visits if site deems appropriate).

Schedule of Activities – Genetic Registry Subjects*

Visit Number	SC/ BSL	PV02	PV04	PV05	PV06	PV07	PV08	PV09	PV10	PV11	PV12	PW	Unsch. Visit
**Visit Description** — **Months (±30 days)**	**0**	**6**	**12**	**18**	**24**	**30**	**36**	**42**	**48**	**54**	**60**	**---**	**---**
Written Informed Consent	X												
Consent/Withdrawal of Consent for Future Contact[#]	X	X	X	X	X	X	X	X	X	X	X	X	X
Advance Directive/Continuing Ability to Consent[#]	X												X
Consent to Share Contact Information (FOUND in PPMI companion protocol)[#]	X	X	X	X	X	X	X	X	X	X	X	X	X
Consent to Share Contact Information with PPMI Brain and Tissue Bank /Advance Directive for PPMI Brain and Tissue Bank s)[# †]	X	X	X	X	X	X	X	X	X	X	X	X	X
Review Inclusion/Exclusion Criteria	X												
Medical History/Demographics	X												
Family History of PD Review	X	X	X	X	X	X	X	X	X	X	X	X	
Family History Substudy (see companion protocol)[#]	X												
Physical Examination	X												
Neurological Examination	X												X[g]
Vital Signs	X[c]												X
Blood Sample for DNA	X												
Clinical Laboratory Assessments	X												X[g]
Biomic blood sample	X[f]												
Olfactory Testing (UPSIT)	X												
MDS-UPDRS (including Hoehn & Yahr)[h]	X												
Modified Schwab & England ADL	X												
Clinical Diagnosis Assessment(s)	X												X[g]
Clinical Diagnosis Review		X	X	X	X	X	X	X	X	X	X	X	
MDS-UPDRS Repeat Part III/Hoehn & Yahr[j]													
Montreal Cognitive Assessment (MoCA)	X												
REM Sleep Behavior Questionnaire	X												
Current Medical Conditions Review		X	X	X	X	X	X	X	X	X	X	X	X
Concomitant Medication Review	X	X	X	X	X	X	X	X	X	X	X	X	X

[c] Height and weight also collected.
[f] Biomic urine sample also collected.
[g] Conduct as clinically indicated – see protocol Sect. 5.3.18.
[h] Part IV once subject has started PD medication.
For active subjects, begin at next visit post amendment approval and consent

PV= Phone Visit
PW = Premature Withdrawal
* With amendment 13 approval, Genetic Registry subjects will complete one final phone visit, and then discontinue visits
j Repeat assessment 1hr post treatment for subjects on levodopa or dopamine agonist.

[†] Intent to Donate documentation completed with PPMI Brain and Tissue Bank study team or local PPMI site based on accessibility, local regulations and availability of infrastructure. For subjects who have not yet been approached concerning the Brain and Tissue Bank, please review subject interest in providing permission to forward their contact information to the PPMI Brain and Tissue Bank study team at their next visit (including interim visits). For subjects who may have already been approached concerning the Brain and Tissue Bank, but were undecided concerning participation or who have not yet signed an Intent to Donate form- please review Brain and Tissue Bank with subject at next annual visit (sites may also review this at interim visits if site deems appropriate).

Modified Schedule of Activities- Parkinson's Disease, Healthy Controls and PD Genetic Cohort Subjects- Post Month 60 or Post January 1, 2019 visits

Visit number	V13	P13	V14	P14	V15	P15	V16	P16	V17	P17	V18	P18	V19	P19	V20	PW visit
**Visit Description** / **Months (+30 days)**	**72**	**78**	**84**	**90**	**96**	**102**	**108**	**114**	**120**	**126**	**132**	**138**	**144**	**150**	**156**	
Consent/Withdrawal of Consent for Future Procedures	X		X		X		X		X		X		X		X	X
Advance Directive/Review Continuing Ability to Consent	X		X		X		X		X		X		X		X	X
Consent to Share Contact Information (FOUND in PPMI companion protocol)	X[+]	X[+]	X[+]	X[+]	X[+]	X[+]	X[+]	X[+]	X[+]	X[+]	X[+]	X[+]	X[+]	X[+]	X[+]	X[+]
Consent to Share Contact Information with PPMI Brain and Tissue Bank /Advance Directive for PPMI Brain and Tissue Bank	X[+]	X[+]	X[+]	X[+]	X[+]	X[+]	X[+]	X[+]	X[+]	X[+]	X[+]	X[+]	X[+]	X[+]	X[+]	X[+]
Vital Status		X		X		X		X		X		X		X		
Confirm/Update Subject Contact Information		X		X		X		X		X		X		X		
Medical & Family History/Demographics	X		X		X		X		X		X		X		X	X
Vital Signs	X		X		X		X		X		X		X		X	X
Biomic blood sample (no urine)	X		X		X		X		X		X		X		X	X
PBMC (assuming volume acceptable)	X		X		X		X		X		X		X		X	X
MDS-UPDRS (including Hoehn & Yahr)^	X		X		X		X		X		X		X		X	X
Modified Schwab & England ADL [a]	X		X		X		X		X		X		X		X	X
Clinical Diagnosis Assessment(s)	X	X	X	X	X	X	X	X	X	X	X	X	X	X	X	X
MDS-UPDRS Repeat Part III/Hoehn & Yahr [#]	X		X		X		X		X		X		X		X	X
Physical Activity Scale for the Elderly (PASE)	X		X		X		X		X		X		X		X	X
Hopkins Verbal Learning Test – Revised	X		X		X		X		X		X		X		X	X
Benton Judgment of Line Orientation	X		X		X		X		X		X		X		X	X
Semantic Fluency (Animals only)	X		X		X		X		X		X		X		X	X
Letter Number Sequencing	X		X		X		X		X		x		X		X	X
Symbol Digit Modalities Test	X		X		X		X		X		X		X		X	X
Lexical Fluency (FAS)	X		X		X		X		X		X		X		X	X
Boston Naming Test	X		X		X		X		X		X		X		X	X
Montreal Cognitive Assessment (MoCA)	X		X		X		X		X		X		X		X	X
Epworth Sleepiness Scale	X		X		X		X		X		X		X		X	X
REM Sleep Behavior Questionnaire	X		X		X		X		X		X		X		X	X
Geriatric Depression Scale (GDS-15)	X		X		X		X		X		X		X		X	X
State-Trait Anxiety Inventory for Adults	X		X		X		X		X		X		X		X	X

QUIP	X		X		X		X		X		X		X		X	X
SCOPA-AUT	X		X		X		X		X		X		X		X	X
Cognitive Categorization	X		X		X		X		X		X		X		X	X
Trails A & B	X		X		X		X		X		X		X		X	X
Determination of Falls	X		X		X		X		X		X		X		X	X
NeuroQOL	X		X		X		X		X		X		X		X	X
Olfactory Testing (UPSIT)	X		X		X		X		X		X		X		X	X
Lumbar puncture (CSF collection)			X				X				X				X	X*
Adverse Events ~			X~				X~				X~				X~	X~
Current Medical Conditions Review	X		X		X		X		X		X		X		X	X
Concomitant Medication Review	X		X		X		X		X		X		X		X	X

* If not done in last 3 months

~ Adverse events assessed at visit and 7 to 10 days following LP (when LP completed as part of a visit)

^ Part IV once subject has started PD medication

Repeat assessment 1hr post treatment for subjects on levodopa or dopamine agonist

+ If subject has not previously consented to contact with PPMI Pathology Core or FOUND in PPMI team

[a] Healthy Control subjects will not complete the Modified Schwab and England ADL

P= Phone visit

PW = Premature Withdrawal

Modified Schedule of Activities- Prodromal and Unaffected Genetic Cohort Subjects- Post Month 60 or Post January 1, 2019 visits

**Visit number**	**V13**	**P13**	**V14**	**P14**	**V15**	**P15**	**V16**	**P16**	**V17**	**P17**	**V18**	**P18**	**V19**	**P19**	**V20**	**PW visit**	**PV visit**
**Visit Description Months (+30 days)**	**72**	**78**	**84**	**90**	**96**	**102**	**108**	**114**	**120**	**126**	**132**	**138**	**144**	**150**	**156**		
Consent/Withdrawal of Consent for Future Procedures	X		X		X		X		X		X		X		X	X	X
Advance Directive/Review Continuing Ability to Consent	X		X		X		X		X		X		X		X	X	
Consent to Share Contact Information (FOUND in PPMI companion protocol)	X$^+$	X$^+$	X$^+$	X$^+$	X$^+$	X$^+$	X$^+$	X$^+$	X$^+$	X$^+$	X$^+$	X$^+$	X$^+$	X$^+$	X$^+$	X$^+$	X$^+$
Consent to Share Contact Information with PPMI Pathology Core	X$^+$	X$^+$	X$^+$	X$^+$	X$^+$	X$^+$	X$^+$	X$^+$	X$^+$	X$^+$	X$^+$	X$^+$	X$^+$	X$^+$	X$^+$	X$^+$	X$^+$
Vital Status		X		X		X		X		X		X		X			
Confirm/Update Subject Contact Information		X		X		X		X		X		X		X			
Participant Questionnaire/Self-Report	X	X	X	X	X	X	X	X	X	X	X	X	X	X	X	X	X
UPSIT			X				X				X				X	X	X
Medical & Family History/Demographics	X		X		X		X		X		X		X		X	X	X
Self-reporting questionnaire	X		X		X		X		X		X		X		X	X	X
Neurological Examination	X		X		X		X		X				X		X	X	X
Vital Signs	X		X		X		X		X		X		X		X	X	X
Biomic blood sample (no urine)	X		X		X		X		X		X		X		X	X	X
PBMC Blood sample	X		X		X		X		X		X		X		X	X	X
MDS-UPDRS (including Hoehn & Yahr) ^	X		X		X		X		X		X		X		X	X	X
Modified Schwab & England ADL	X		X		X		X		X		X		X		X	X	X
MDS-UPDRS Repeat Part III/Hoehn & Yahr #	X		X		X		X		X		X		X		X	X	X
Clinical Diagnosis Assessments	X	X	X	X	X	X	X	X	X	X	X	X	X	X	X	X	X
Physical Activity Scale for the Elderly (PASE)	X		X		X		X		X		X		X		X	X	X
Hopkins Verbal Learning Test – Revised	X		X		X		X		X		X		X		X	X	X
Benton Judgment of Line Orientation	X		X		X		X		X		X		X		X	X	X
Semantic Fluency-Animals only	X		X		X		X		X		X		X		X	X	X
Letter Number Sequencing	X		X		X		X		X		X		X		X	X	X
Symbol Digit Modalities Test	X		X		X		X		X		X		X		X	X	X
Lexical Fluency (FAS)	X		X		X		X		X		X		X		X	X	X
Boston Naming Test (15 item)	X		X		X		X		X		X		X		X	X	X
Montreal Cognitive Assessment	X		X		X		X		X		X		X		X	X	X
Epworth Sleepiness Scale	X		X		X		X		X		X		X		X	X	X
REM Sleep Behavior Questionnaire	X		X		X		X		X		X		X		X	X	X
Geriatric Depression Scale (GDS-15)	X		X		X		X		X		X		X		X	X	X

State-Trait Anxiety Inventory for Adults	X		X		X		X		X		X		X		X	X	X
QUIP	X		X		X		X		X		X		X		X	X	X
SCOPA-AUT	X		X		X		X		X		X		X		X	X	X
Cognitive Categorization	X		X		X		X		X		X		X		X	X	X
Trails A & B	X		X		X		X		X		X		X		X	X	X
Determination of Falls	X		X		X		X		X		X		X		X	X	X
NeuroQOL	X		X		X		X		X		X		X		X	X	X
DAT imaging [a]																	X
Lumbar puncture (CSF collection)			X				X				X				X	X*	X*
Adverse Events ~			X~				X~				X~				X~	X~	X~
Current Medical Conditions Review	X		X		X		X		X		X		X		X	X	X
Concomitant Medication Review	X		X		X		X		X		X		X		X	X	X

* If not done in last 3 months
~ Adverse events assessed at visit and 7 to 10 days following LP or DAT imaging (when LP or DAT imaging completed as part of a visit)
^ Part IV once subject has started PD medication
# Repeat assessment 1hr post treatment for subjects on levodopa or dopamine agonist
[a] Urine (or serum) pregnancy test prior to injection for women of childbearing potential.
+ If subject has not previously consented to contact with PPMI Pathology Core or FOUND in PPMI team

P= Phone visit
PW = Premature Withdrawal
PV=Phenoconversion visit

Table of Content

INVESTIGATOR AND CORE FACILITY CONTACT INFORMATION 2

PROTOCOL APPROVAL 5

INVESTIGATOR AGREEMENT 6

LIST OF ABBREVIATIONS AND DEFINITIONS 7

PROTOCOL SYNOPSIS 9

SCHEDULE OF ACTIVITIES – HEALTHY CONTROL (HC) SUBJECTS [PLEASE SEE MODIFIED SCHEDULE OF ACTIVITIES ON PAGE 28 FOR VISITS POST JANUARY 1 2019] 17

SCHEDULE OF ACTIVITIES – SWEDD SUBJECTS 19

SCHEDULE OF ACTIVITIES – PRODROMAL SUBJECTS [PLEASE SEE MODIFIED SCHEDULE OF ACTIVITIES ON PAGE 30 FOR POST MONTH 60 VISITS] 21

SCHEDULE OF ACTIVITIES – GENETIC COHORT: PD SUBJECTS [PLEASE SEE MODIFIED SCHEDULE OF ACTIVITIES ON PAGE 28 FOR POST MONTH 60 VISITS] 23

SCHEDULE OF ACTIVITIES – GENETIC COHORT: UNAFFECTED SUBJECTS [PLEASE SEE MODIFIED SCHEDULE OF ACTIVITIES ON PAGE 30 FOR POST MONTH 60 VISITS] 25

SCHEDULE OF ACTIVITIES – GENETIC REGISTRY SUBJECTS* 27

1. INTRODUCTION 34

1.1. BACKGROUND 34
1.2. RATIONALE FOR PPMI 38

2. STUDY OBJECTIVES 40

2.1. PRIMARY OBJECTIVE 40
2.2. SECONDARY OBJECTIVES 40

3. STUDY OUTCOMES 41

3.1 PRIMARY OUTCOMES 41
3.2 SECONDARY OUTCOMES 41

4. STUDY DESIGN AND POPULATIONS 42

4.1 OVERALL STUDY DESIGN 42
4.2. SELECTION OF STUDY POPULATION 52
4.3 AGE AND GENDER MATCHING 61

5. INVESTIGATIONAL PLAN 61

5.1. SUBJECT IDENTIFICATION NUMBERS 61
5.2 SCHEDULE OF ACTIVITIES 62
5.3 STUDY PROCEDURES AT EACH VISIT (PD, HEALTHY, SWEDD, PRODROMAL, GENETIC COHORT AND GENETIC REGISTRY) 63

6. STUDY ASSESSMENTS 91
6.1. CLINICAL ASSESSMENTS 91
6.2. SAFETY ASSESSMENTS 96
6.3. OTHER ASSESSMENTS 98
7. CONCOMITANT MEDICATIONS 102
8. SUBJECT WITHDRAWALS 103
9. SUBJECT RETENTION 103
9.1 ADVANCE DIRECTIVE FOR CLINICAL RESEARCH PARTICIPATION 103
9.2. FOLLOW UP OF PERSONS WITH NEUROLOGIC DISEASE (FOUND) IN PPMI 104
10. PPMI BRAIN AND TISSUE BANK 105
11. SAFETY/ADVERSE EVENTS 106
10.1 ADVERSE EVENT DEFINITIONS 106
10.2 RELATIONSHIP TO INVESTIGATIONAL PRODUCT 108
10.3 INTENSITY/SEVERITY OF AN ADVERSE EVENT 108
10.4 RECORDING OF ADVERSE EXPERIENCES 109
10.5 RESPONSIBILITIES FOR REPORTING SERIOUS ADVERSE EXPERIENCES 109
11. REPORTABLE EVENTS 110
12. REFERRALS 110
13. POTENTIAL RISKS 110
13.1 BLOOD SAMPLING 110
13.2. LUMBAR PUNCTURE 110
13.3. MRI / DTI 111
13.4. IMAGING 111
13.5 . RISK OF DISCLOSURE OF GENETIC INFORMATION 111
13.6 UNKNOWN RISKS 112
14. STATISTICAL METHODS 112
14.1 STATISTICAL DESIGN 112
14.2 PRIMARY OBJECTIVE 112
14.3 SECONDARY OBJECTIVES 113
14.4. PLANNED ANALYSES 113
14.5. DETERMINATION OF SAMPLE SIZE 115
15. REGULATORY / ETHICS 116
15.1. COMPLIANCE STATEMENT 116
15.2. INFORMED CONSENT 117
15.3. INSTITUTIONAL REVIEW BOARD/INDEPENDENT ETHICS COMMITTEE 117
15.4. PROTOCOL AMENDMENTS 118
15.5. SUBJECT CONFIDENTIALITY 118
16. DOCUMENTATION 118
16.1. STUDY FILE AND SITE DOCUMENTS 118
16.2. MAINTENANCE AND RETENTION OF RECORDS 119
16.3. CASE REPORT FORMS 119
16.4. PRIMARY SOURCE DOCUMENTS 120
17. DATA MANAGEMENT 120

18. STUDY MONITORING ... 121
10. PUBLICATION OF RESEARCH FINDINGS ... 122
REFERENCES ... 124

1. Introduction

1.1. Background

The defining motor features of Parkinson disease (PD) are characterized by their insidious onset and inexorable but variable progression. Reliable and well-validated biomarkers to monitor PD progression would dramatically improve patient care and accelerate research into both PD etiology and therapeutics. During the past two decades much progress has been made in identifying and assessing PD biomarkers, but as yet no fully validated biomarker for PD is currently available. Nonetheless there is increasing evidence that assessment of blood and CSF, and advanced *in vivo* brain imaging will provide critical tools for PD drug development and ultimately to assist in the medical management of PD patients.

While it is important to acknowledge that a true surrogate marker for PD does not exist, it is even more critical to note that existing and developing biomarkers for PD are and may be extremely useful for disease monitoring and for drug development. In considering biomarkers for PD three crucial issues must be addressed: 1) to identify whether the marker is meaningful or relevant to the disease process; 2) to identify the performance characteristics of the marker in the relevant subject population (e.g., examination of markers for PD progression in early, untreated PD patients); and 3) to determine the generalizability of the biomarker to the larger disease population (e.g., carefully assess how the biomarker is affected by PD stage, subject age or gender, medications, or environment).

<u>Use of biomarkers to complement clinical assessments during a research study</u>
Biomarkers offer the potential to complement the clinical assessments used as a primary study outcome in clinical studies of PD. Although biomarkers are generally objective measures of disease and are more easily standardized and reproducible, standardization of biomarker collection and analysis requires clear and detailed procedures to enable objective data to be pooled at multiple study sites. As a result, biomarkers are most often assessed at a core lab with expertise in analytical methodology. Specific procedures for transfer of biological samples and/or imaging data must be in place. The AMADEUS imaging network, a SPECT imaging consortium for PD, and the Alzheimer Disease Neuroimaging Initiative (ADNI) for AD have demonstrated that both biological and imaging samples can be collected and analyzed in studies of disease progression[1]. Given the multiple genetic etiologies for PD already identified, the marked variability in the loss of dopaminergic markers measured by imaging at motor symptom onset, and the clear heterogeneity of clinical symptoms in PD onset and clinical progression, it is clear that many biomarkers with a focus ranging from clinical symptoms to PD pathobiology to molecular genetic mechanisms will be necessary to fully map PD progression[2-4].

<u>Assessment of multiple biomarkers in a single subject</u>
In clinical studies, multiple biomarkers may be assessed in the same subjects. This strategy may enable comparison and correlation of biomarkers from imaging markers, biomic assays,

and/or markers that target multiple neuronal systems. When multiple biomarkers are evaluated concurrently, the power of the study may be substantially increased. For example in studies of PD relatives tested for olfactory function and then undergoing dopamine transporter imaging, combining the loss of olfaction and dopamine transporter imaging density identifies a sub-group with increased risk of developing PD[5]. Imaging studies comparing dopaminergic ligands and metabolic tracers have provided complementary data enhancing the utility of both tracers[6]. The potential of combining in vivo radiotracer imaging and/or nigral ultrasound with proteomic, metabolomic and transcriptomic analyses is currently under evaluation in several ongoing studies such as the PARS study and other risk marker assessment studies[7,8].

Use of biomarkers to define a study cohort

Biomarkers may be also used in clinical studies to better define or enhance the study cohort. While pre-defining the cohort may reduce generalizability of the study outcome, using biomarkers to define eligibility may ensure a more accurate diagnosis of the study subjects and therefore reduce variance in the outcome. In several studies of newly diagnosed PD in vivo dopaminergic imaging has identified about 10-15% with scans in the normal range termed scans without evidence of dopaminergic degeneration (SWEDD)[2,9,10]. Subsequent follow-up has indicated that those study participants with SWEDD are unlikely to have PD[11]. In the ELLDOPA study and REAL PET study data analysis using the imaging biomarker to define the study cohort changed the statistical significance of study outcomes[9,10]. In other studies, biomarkers are used as an *a priori* definition of the study cohort as in studies of subjects with a LRRK2 mutation. These studies utilize biomarkers to explore a specific etiology or sub-type of PD.

Furthermore, clinical, genetic and DAT imaging biomarkers identified from existing and ongoing proof of concept studies may be used to identify a cohort of subjects who are at high risk of developing PD (referred to as the ‘Prodromal’ cohort). These studies suggest that subjects with risk due to hyposmia, RBD or LRRK2 mutation, with DAT imaging in the PD range, who do not have motor symptoms consistent with PD, are at risk to develop motor PD within approximately two years of the imaging assessment. Successful identification of this Prodromal cohort will provide evidence for the utility of biomarkers in identification of prodromal PD, which would improve knowledge of early etiology and present an opportunity to target interventions earlier in the disease process.

Utility of biomarkers in clinical study design

Perhaps the most important rationale for using biomarkers in clinical studies is the potential for longitudinal biomarker outcomes to provide critical data with a shorter duration of follow-up and a reduced sample size compared to that required of typical clinical outcomes. The sample size requirements for a progression study depend on the effect size and the variance of the outcome measure. For example, in vivo dopamine neuroimaging requires a similar sample size and observation interval to that of common clinical outcomes (change in UPDRS or need for dopaminergic therapy)[2,12]. Identifying biomarkers that could provide a more rapid assessment of drug effect would substantially accelerate development of putative disease modifying drugs.

In the PPMI study, a striatal dopamine transporter deficit on dopamine transporter imaging, or (for sites where DaTSCAN™ is not available) a striatal monoamine transporter deficit on VMAT2 imaging, is a requirement for inclusion into the PD cohort. The purpose of providing

these inclusion criteria was to increase the probability that the PD subjects (screened with at least an asymmetric motor sign) enrolled in PPMI have a diagnosis of PD or a Parkinsonian Syndrome (PS) with the characteristic signs of degeneration of dopaminergic neurons in the substantia nigra pars compacta. Developing this relatively 'pure' PD cohort is especially important for the PPMI study in which the development and evaluation of biomarkers for PD is the main purpose. While the subjects screened as PD without evidence of dopaminergic (or VMAT) deficit (SWEDD) are not enrolled as PD subjects in the PPMI protocol, there is important information to be gained by following these subjects and better understanding their clinical progression, biomic characterization and ultimately their clinical diagnosis.

Finally, given evidence that pre-motor subjects with hyposmia, RBD or LRRK2 mutation who also have a DAT deficit (compared to healthy subjects) have a high risk of conversion to motor PD, a Prodromal cohort will be enrolled with hyposmia, RBD and/or LRRK2 mutation with DAT binding in the PD range. Since we are uncertain about the exact distribution of DAT binding in this group, and while we anticipate that the majority of subjects will have a range of DAT deficit similar to subjects with early PD, we will also select no DAT deficit or minimal DAT deficit subjects similar in age, gender, and risk profile to those with mild to moderate DAT deficit. Phenoconversion of these at risk subjects to motor PD will be determined according to research based diagnostic criteria as assessed by the Investigator.

<u>Current landscape of biomarkers for PD progression</u>
Progression markers for PD are both a critical and as yet an unmet need. Validated biomarkers of disease progression are necessary to monitor the natural history of PD and to assess therapies that may modify disease progression.

Although no fully validated progression biomarker has been developed, several biomarkers have been tested in PD progression studies. In some studies, biomarkers such as nigral ultrasound hyperechogenicity and microglial activation as a measure of neuroinflammation may be markers of disease risk, but do not appear to change with disease progression[13,14]. However, other studies have identified biomarkers such as alpha-synuclein, elevated plasma urate and imaging measures such as DAT density that may predict or track disease. While there is little direct data that alpha-synuclein is a progression marker, the age related changes in alpha-synuclein and association with PD severity suggest that it may be possible to measure change as PD progresses[15,16]. Recent assessment of two large PD clinical trials has demonstrated that elevated urate may be associated with slowed disease progression, possibly predicting PD progression, but not tracking progression[17].

Imaging tracers targeting presynaptic nigrostriatal function have been the most widely used biomarker to track PD progression. Most of these studies have used either F-Dopa and/or DAT tracers to monitor dopaminergic degeneration[18-25]. Dopamine ligands are useful to assess PD in so far as they reflect the ongoing dopaminergic degeneration in PD. In the study most directly correlating changes in dopamine pathology and imaging outcomes there is good correlation between dopamine neuron loss and F-Dopa uptake, although conclusions are limited by a very small sample size of only five subjects[26]. Numerous other studies have shown that the dopamine transporter density is reduced in striatum in postmortem brain from PD patients[27-29]. In turn numerous clinical imaging studies have shown reductions in F-Dopa, VMAT2 and DAT ligands uptake in PD patients and aging healthy subjects consistent with the expected pathology of PD and of normal aging. Specifically these imaging studies demonstrate

asymmetric, putamen>caudate loss of dopaminergic uptake and the imaging loss correlates with worsening clinical symptoms in cross-sectional evaluation[22,30-34]. In addition, DAT ligands demonstrate reductions in activity with normal aging[35-37].

In longitudinal studies of PD progression, F-Dopa, VMAT2, DAT (ß-CIT and CFT), using both PET and SPECT have demonstrated an annualized striatal rate of reduction of about 4% to 13% in PD patients compared with 0% to 2.5% change in healthy controls[6,9,12,38-43]. Evidence from studies of hemi-PD subjects provides further insight into the rate of progression of disease. In early hemi-PD there is a reduction in F-Dopa, VMAT2 and DAT of about 50% in the effected putamen and of 25-35% in the unaffected putamen. Since most patients will progress clinically from unilateral to bilateral in 3-6 years it is therefore likely that the loss of these in vivo imaging markers of dopaminergic degeneration in the previously unaffected putamen will progress at about 4-10% per annum[19,33,44,45].

DAT and F-Dopa imaging have been used to assess the effects of possible disease modifying drugs in several clinical trials. However, several caveats limit the interpretation of these imaging data[46,47]. There has been concern that the drug under testing or concomitant symptomatic medications might directly regulate the imaging outcome so that it would not be a true measure of disease progression. Given that recent studies demonstrate that the most common symptomatic medications (levodopa and dopamine agonists) do not have a short-term regulatory effect on DAT imaging, there is increased confidence in DAT imaging as a measure of progression[48]. Nonetheless, future imaging studies must include an assessment of the short-term effect of the test drug on the imaging outcome. A second caveat for imaging studies of disease progression has been the inconsistent correlation of changes in imaging outcomes and clinical outcomes in these clinical trials. The lack of clinical–imaging correlation may be explained since these outcomes reflect very different aspects of the disease (imaging – a physiological measure of dopamine presynaptic function, clinical – a functional measure of disability). Therefore imaging and clinical outcomes may best be considered complementary rather than correlative. Many clinical outcomes may be also confounded by symptomatic medications further complicating the correlation of clinical and imaging outcomes once symptomatic treatment has begun.

In summary, the studies of dopaminergic imaging as a tool for disease progression have both provided useful and important data but have also highlighted the difficulties in validating a progression marker and the as yet unmet need for additional tools to more fully and more rapidly assess disease progression.

<u>Current landscape for prodromal PD biomarkers</u>
Parkinson disease has a prolonged pre-diagnostic period during which nigral dopamine degeneration and likely brainstem and olfactory bulb pathology has occurred several years in advance of typical motor symptoms. Accurately defining a pre-diagnostic or prodromal phase for PD requires the ability to reliably identify individuals who are at high risk to phenoconvert to PD, but presents an opportunity to ultimately provide early intervention to treat these individuals prior to the onset of motor symptoms or delay the development of manifest PD.

Numerous studies have demonstrated that at clinical diagnosis of PD, a 40-60% loss of dopaminergic neurons has already occurred during a prodromal period estimated to be 5-15 years in duration[2,9,40,48-54]. Several lines of evidence demonstrate that dopaminergic imaging

can reliably identify subjects with a reduction in dopamine transporter density or F-Dopa during this prodromal phase of their disease. Early non-motor symptoms like olfactory loss, autonomic dysfunction, sleep disturbance, cognitive and behavioral impairment may also occur prior to typical PD motor symptoms, reflecting early brainstem or extra-nigral pathology. These symptoms may serve as sensitive, but non-specific clinical markers that may enrich a population to be further assessed with dopaminergic imaging markers to more specifically identify PD subjects with early evidence of neurodegeneration[55-58]. Recent understanding of both these early non-motor manifestations and the molecular genetics of PD have provided several specific strategies to identify those individuals without typical PD motor symptoms, but at increased risk to develop PD[59-62]. Several proof of concept studies have demonstrated that enriching a population for PD risk by combining non-motor symptoms (olfaction or RBD) or genetic risk with dopamine transporter imaging (DAT) may successfully identify individuals at high risk to phenoconvert to manifest PD.

The PPMI study presents an opportunity to use clinical, genetic and DAT imaging biomarkers tested in these existing and ongoing proof-of-concept studies to develop and follow a cohort at high risk of developing PD within two years of the imaging assessment.

1.2. Rationale for PPMI

Given that lack of validated biomarkers for PD and prodromal PD have impeded therapeutic development, there is increasing consensus that a major initiative to develop biomarkers for disease progression and early detection is both necessary and feasible. Recent advances in molecular genetics, neurobiology, imaging technology and radiochemistry have provided new tools that may be useful in identification of such biomarkers for further studies of disease modifying therapies. The goal of this biomarker initiative is to create a consortium of academic centers, government agencies, PD foundations, and pharmaceutical and biotech companies to collectively design, fund, and implement a comprehensive program to establish markers of PD progression. This strategy has been successfully employed by a consortium of Alzheimer disease researchers to create the ADNI, a group that has now organized infrastructure and has developed a research cohort to examine progression biomarkers in AD.

In PPMI, technologies including neuroimaging modalities, biochemical markers in the CSF and plasma, genetic markers, and early clinical disease markers will be investigated. A major initial focus of this biomarker consortium will be to standardize biomarker acquisition and assessment and to establish well-defined quantitative biomarker outcomes that are consistent among many research sites and laboratories. Core laboratories for biomarker analysis will be used for uniformity of analyses and quality control.

Genetic approaches to understanding disease are complex and rapidly changing; however, we anticipate several approaches that may be applied to this study population. First, for subjects enrolled with idiopathic PD, prodromal subjects identified by olfactory loss or RBD, and healthy subjects focused genotyping that aims to interrogate genetic variability that is implicated in the disease process, either by previous studies or as a plausible biological candidate. This would include, for example, the more common LRRK2 and GBA mutations, in addition to previously validated risk variants in SNCA and MAPT. Second, more broad application of genome wide analyses, both because such approaches may become a more cost effective alternative to focused genotyping and because at some point in the future these patient

samples may be included in larger population based studies on the genetics of PD. Such methods would include genome wide association studies, whole exome sequencing and whole genome sequencing. Third, given the increasing opportunity to identify and enroll specific genetic cohorts, PPMI can add a genetic cohort to investigate the relationship between idiopathic PD and PD with known genetic mutation and further explore the prodromal PD period in unaffected family members of individuals with PD with known genetic mutation in LRRK2, GBA or SNCA.

Mutations in LRRK2 result in autosomal dominant PD that presents at approximately 60 years of age, but which can also occur more than a decade earlier or later. Individuals who carry a mutation in one of the 2 LRRK2 alleles are at significantly greater risk of developing PD. However, it has been shown that not all individuals who inherit a LRRK2 mutation will develop PD. Rather, there is age-dependent penetrance of the mutation. Precise estimates of the penetrance of the mutation are poor and PPMI provides an opportunity to better estimate these rates. There is one predominant disease-producing mutation in LRRK2, G2019S which has been found at significantly greater frequencies among PD patients of Ashkenazi Jewish and northern African ancestry. Other pathologic mutations include the R1441C and R1441G (most prevalent in Northern Spain).

Mutations in GBA result in autosomal dominant PD that also generally presents at approximately after 60 years of age, but also can occur decades earlier or later. Similar to LRRK2, it has been shown that not all individuals who inherit a GBA mutation will develop PD. Precise estimates of the penetrance of a GBA mutation are uncertain and PPMI provides an opportunity to better estimate these rates. Individuals who are homozygous for GBA mutations may develop Gaucher disease and these individuals are at increased risk of PD compared to GBA+ heterozygotes. While there are several identified GBA mutations, the study will focus on the N370S as well as several other GBA mutations that have been found at higher frequency among Ashkenazi Jewish ancestry. The study may also test for other mutations of interest to PD.

Mutations in SNCA can also result in autosomal dominant PD. Individuals with a mutation in one of their two SNCA alleles typically develop PD in their 40's, although the age of onset can be variable. The penetrance of the SNCA mutation is uncertain and estimates are poor because so few families have been found with a SNCA mutation that results in PD.

Imaging tools for PD provide both an opportunity for ensuring accuracy of diagnosis and for assessing disease progression. Early diagnosis of PD in de novo subjects, like those being recruited for PPMI, is difficult because characteristic signs and symptoms have not yet fully emerged and patients may present atypical signs and symptoms. Diagnostic accuracy in a de novo PD population is a function of disease stage (the earlier the stage the more difficult the diagnosis) and examiner expertise, diagnoses by movement disorder specialists being more accurate than by general neurologists[12]. For example, in prior PD clinical trials enrolling newly diagnosed subjects, ten to fourteen percent of subjects have SWEDD[2,9,10]. Subsequent follow-up suggests that these subjects are unlikely to have idiopathic PD. Understanding the biomarker profiles in the subjects without dopamine transporter deficit provides a dataset that serves as a 'disease control' and offers biomarker information with which to compare with PD and healthy control subjects.

This approach to biomarker development is ambitious and requires cooperation among many in academics, industry, government, and the public sector. The lack of success of recent disease modifying therapeutic trials[2] coupled with the huge expense of these studies has highlighted the need for such an approach to identify and validate biomarkers of PD progression for future clinical studies of disease modifying drugs.

2. Study Objectives

2.1. Primary Objective

The overall objective of this study is to identify clinical, imaging and biologic markers of PD progression for use in clinical trials of disease-modifying therapies.

Specific aims to accomplish this objective are:

a. Establish standardized protocols for acquisition, transfer and analysis of clinical, imaging and biologic data that can be used by the PD research community.
b. Develop a comprehensive and uniformly acquired clinical and imaging dataset and biological samples that can be used to estimate the mean rates of change and the variability around the mean of clinical, imaging and biomic outcomes in early PD patients, prodromal PD subjects, and PD subjects with a LRKK2, GBA or SNCA mutation.
c. Investigate existing and identify novel clinical, imaging, and biomic Parkinson disease progression markers to identify quantitative individual measures or combination of measures that demonstrate optimum interval change in PD patients in comparison to healthy controls, SWEDD subjects, Prodromal subjects, or in sub-sets of PD patients, PD subjects with a LRRK2, GBA or SNCA mutation, unaffected LRRK2, GBA or SNCA mutation carriers, defined by baseline assessments, genetic mutations, progression milestones and/or rate of clinical, imaging, or biomic change.
d. Conduct preliminary verification studies on promising biological markers using stored collected samples.

2.2. Secondary Objectives

2.2.1. To evaluate the probability of a change over a 24-month period in the clinical diagnosis of PPMI PD subjects with a baseline dopamine transporter image that shows no evidence for striatal DAT or VMAT deficit (SWEDD).

2.2.2. Assess the influence of dopamine transporter or VMAT2 imaging status on the clinical diagnosis and management of SWEDD subjects.

2.2.3. To evaluate the probability of phenoconversion to PD for individuals enrolled in the Prodromal cohort and Unaffected Genetic Cohort.

2.2.4. Investigate existing and identify novel clinical, imaging, and biomic Parkinson disease progression markers to identify quantitative individual measures or combination of measures that demonstrate optimum interval change of SWEDD subjects in comparison to healthy controls, PD subjects, Prodromal subjects, Genetic Cohort or in sub-sets of PD patients defined by baseline assessments, genetic mutations, progression milestones and/or rate of clinical, imaging, or biomic change.

2.2.5. Investigate existing and identify novel clinical, imaging, and biomic Parkinson disease progression markers to identify quantitative individual measures or combination of measures that demonstrate optimum interval change of Prodromal subjects (including both prodromal cohort and Unaffected Genetic Cohort) in comparison to healthy controls, PD subjects, SWEDD subjects or sub-sets of PD patients defined by baseline assessments, progression milestones and/or rate of clinical, imaging, or biomic change.

3. Study Outcomes

3.1 Primary Outcomes

The mean rates of change and the variability around the mean of clinical, imaging and biomic outcomes in early PD patients, and where appropriate the comparison of these rates between PD patient subsets and between various subsets (including, but not limited to: PD vs. healthy subjects, PD vs. SWEDD, PD vs. prodromal, PD with and without LRKK2, GBA or SNCA mutation, unaffected LRRK2, GBA or SNCA mutation carriers vs. healthy subjects) at study intervals ranging from 3 months to 36 months. Specific examples of outcomes include MDS-UPDRS, dopamine transporter striatal uptake, vesicular monoamine transporter type-2 uptake, and serum and CSF alpha-synuclein**.** PD patient subsets may be defined by baseline assessments, genetic mutation, progression milestones and/or rate of clinical, imaging, or biomic change.

3.2 Secondary Outcomes

a) To compare the rates of change in the mean of clinical, imaging and biomic outcomes in various subsets (including, but not limited to: early PD patients vs. healthy subjects, PD vs. SWEDD, PD vs. prodromal, PD with and without LRRK2, GBA or SNCA mutation, unaffected LRRK2, GBA or SNCA mutation carriers vs. healthy subjects) at study intervals ranging from 3 months to 60 months.
b) To determine the prevalence of measures of clinical, imaging and biomic outcomes various subsets (including, but not limited to: in early PD patients and healthy subjects, PD vs. SWEDD, PD vs. prodromal, PD with and without LRRK2, GBA or SNCA mutation, unaffected LRRK2, GBA or SNCA mutation carriers vs. healthy subjects) at study intervals from baseline to 156 months.
c) To establish the predictive value of early clinical non-motor features, baseline imaging and biomic outcomes for future course of disease.
d) To examine the proportion of SWEDD subjects having a change in their clinical management at 24 months (SWEDD Clinical Diagnosis and Management Questionnaire).
e) To estimate the percentage of Prodromal subjects with one or more risk characteristics [hyposmia (<10th percentile by age and gender), RBD, or genetic mutation (LRRK2, GBA or SNCA), and baseline DaTSCAN binding showing minimal to moderate DAT deficit] who phenoconvert to PD within two years, and an exploratory analysis to examine whether the baseline DaTSCAN binding or progression of clinical, imaging, or biospecimen markers may predict those subjects likely to phenoconvert.

4. Study Design and Populations

4.1 Overall Study Design

Longitudinal, multi-center study to assess progression of clinical features, imaging and biologic biomarkers in Parkinson disease (PD) patients compared to healthy controls (HC) and in PD patient subtypes. PPMI will be a thirteen-year natural history study (a minimum of 5-year involvement) of *de novo* idiopathic PD patients and healthy controls. Approximately 400 PD and 200 healthy controls and other cohorts detailed below will be recruited from about 33 clinical sites. All subjects will be comprehensively assessed at baseline and every three to six months thereafter. Subjects will undergo clinical (motor, neuropsychiatric and cognitive) and imaging assessments and will donate blood, urine, and cerebral spinal fluid (CSF). A blood sample for DNA will be collected. Data will be collected by each site under uniformly established protocols and data will be stored and analyzed at designated core facilities.

In addition, biomarkers will be assessed in approximately 60 – 100 SWEDD subjects. The SWEDD subjects will be followed over a 2 year (24 month) period at the same intervals and visit schedule as the PD and Control subjects participating in the PPMI study. Subjects will undergo a dopamine transporter imaging scan at screening and 24 months. The clinical diagnosis on the Clinical Diagnosis and Management Questionnaire at the 24 Month visit will serve as the final clinical diagnosis for SWEDD subjects, unless there is substantial evidence that the subject may have PD (e.g., per clinical assessment of the repeat imaging scan suggestive of a dopamine deficit). In this event the subject may be asked to extend or (if already completed their final visit at Month 24) return to participation in the study up to a total of thirteen years.

An additional cohort of Prodromal subjects will be included in the clinical, imaging and biologic assessments of PPMI. Approximately 100 Prodromal subjects will be enrolled and followed over a 5-10 year period. Prodromal subjects with RBD, and/or hyposmia with DAT binding ranging from no DAT deficit to moderate DAT deficit will be selected (most subjects with DAT binding <80% of age expected striatal DAT binding based on the existing PPMI healthy subject database). Since we are uncertain about the exact distribution of DAT binding in this group, and while we anticipate that the majority of subjects (approx. 80) will have a range of DAT deficit similar to subjects with early PD, we will also select no DAT deficit or minimal DAT deficit subjects (approx. 20) to be similar in age, gender, and risk profile to those with mild to moderate DAT deficit. Subjects will not be informed of their DAT binding. It is anticipated that approximately 400 screening SPECT scans will be completed in order to identify the 100 Prodromal subjects for participation and enrollment.

An additional cohort of subjects selected because of genetic risk will be included in the clinical, imaging and biologic assessments of PPMI. Approximately 1200 subjects will be enrolled and followed over a 5-10 year period. Approximately 600 of these 1200 subjects will be evaluated as part of the Genetic Cohort and approximately an additional 600 will be evaluated as part of the Genetic Registry. The Genetic Cohort participants will be evaluated similarly to the already enrolled PPMI PD and healthy control cohort. The Genetic Registry participants will be evaluated at less frequent intervals to augment and broaden the follow-up of PD subjects and family members with PD associated mutations and are in addition to the Genetic Cohort.

Genetic Cohort (n=600)

- 300 PD patients with a mutation (LRRK2, GBA or SNCA)
- 300 unaffected individuals with a mutation (LRRK2, GBA or SNCA) and/or a first degree relative with a mutation (LRRK2, GBA or SNCA)

Genetic Registry (n=600)

- 300 PD patients with a mutation (LRRK2, GBA, or SNCA 300 unaffected individuals with a mutation (LRRK2, GBA, or SNCA) or with a first degree relative with a mutation (LRRK2, GBA, or SNCA)

Subjects must have a causative LRRK2, GBA or SNCA mutation to be considered as having a positive LRRK2, GBA or SNCA mutation.

The subjects participating in the Genetic Cohort and Genetic Registry will be identified by pre-screening activities including genetic testing and counseling. In order to be recruited for genetic testing, an individual must be 18 years of age or older and be in at least one of the following categories:

- Has PD **and** 1) has a first degree relative with LRRK2, GBA or SNCA mutation **and** 2) is willing to be informed of their own testing results
- Has PD **and** 1) is from an ethnic or geographic group known to have high risk of LRRK2, GBA or SNCA mutation (such as people of Ashkenazi Jewish or Basques descent) **and** 2) is willing to be informed of their own testing results
- Is unaffected **and** at high risk of LRRK2, GBA or SNCA mutation due to first degree relative with a LRRK2, GBA or SNCA mutation. These subjects may choose to be informed or remain unaware of their own testing results
- Is unaffected **and** 1) is from an ethnic or geographic group known to have relatively high risk of LRRK2, GBA or SNCA mutation (such as people of Ashkenazi Jewish or Basques descent) **and** 2) has a family member (either alive or deceased) who has/had PD and 3) is willing to be informed of their own testing results.

PD and unaffected individuals verified through previous genetic testing to have LRRK2, GBA or SNCA mutation may also be recruited and do not require retesting.

Once genetic testing results are known, individuals will be informed that they are not eligible to participate in the Genetic Cohort or Genetic Registry or will be invited to participate in either the PD Genetic Cohort, Unaffected Genetic, or Genetic Registry. The genetic cohort or registry assignment will be based on the subject's genetic status, awareness of results, age, and duration of PD as indicated below and in the eligibility criteria. Individuals who are qualified for either arm of the Genetic Cohort but decline will be offered participation in the Genetic Registry. See Figures 1A and 1B. With approval of amendment 12, sites will no longer enroll subjects with a LRRK2 or GBA mutation into the Genetic Registry. Subjects who are not eligible for the Genetic Cohort (and were formerly eligible for the Genetic Registry) will become ineligible for enrollment into PPMI except for subjects with a SNCA mutation. Please refer to specific eligibility criteria below for details.

For individuals with PD

– The PD subjects must be willing to be informed of their genetic testing results

  - o Individuals with PD who are found to be gene negative will be made aware of their genetic status and will not be eligible for either the Genetic Cohort or Registry.
  - o Individuals with PD who are found to have a LRRK2, GBA or SNCA mutation will be informed of their genetic status.
    - Those who were diagnosed within the past 7 years will be invited to participate in the PD Genetic Cohort.
      - Those who were diagnosed more than 7 years ago will not be eligible for the Genetic Cohort.

For unaffected individuals at high risk of LRRK2, GBA or SNCA mutation due to first degree relative with a LRRK2, GBA or SNCA mutation

– These unaffected individuals at high risk of LRRK2, GBA or SNCA mutation due to first degree relative with a LRRK2, GBA or SNCA mutation may choose to be informed or unaware of their genetic testing result

  - o Unaffected individuals at high risk of LRRK2, GBA or SNCA mutation due to first degree relative with a LRRK2, GBA or SNCA mutation who choose to be made aware of their genetic status and are found to have a LRRK2 or GBA mutation and are 45 years or older (or SNCA mutation and 30 or older) will be invited to participate in the Genetic Cohort.

  - o Unaffected individuals at high risk of LRRK2, GBA or SNCA mutation due to first degree relative with a LRRK2, GBA or SNCA mutation who choose to be made aware of their genetic status and are found to have a LRRK2 or GBA mutation and are younger than 45 will not be eligible for enrollment in PPMI. Individuals with SNCA mutation and younger than 30 will be invited to participate in the Genetic Registry.
    - ▪ Unaffected individuals at high risk of LRRK2, GBA or SNCA mutation due to first degree relative with a LRRK2, GBA or SNCA mutation who choose to be made aware of their genetic status and are found to have a LRRK2 or GBA mutation and were enrolled in the Genetic Registry previously may transition into the Genetic Cohort provided that the current inclusion criteria is met.

  - o Unaffected individuals at high risk of LRRK2, GBA or SNCA mutation due to first degree relative with a LRRK2, GBA or SNCA mutation who choose to be made aware of their genetic status and are found to be gene negative will not be eligible for either the Genetic Cohort or Registry.

  - o Unaffected individuals at high risk of LRRK2, GBA or SNCA mutation due to first degree relative with a LRRK2, GBA or SNCA mutation who choose to remain unaware of their genetic status and are found to have a LRRK2 or GBA mutation

and are 45 years or older (or SNCA mutation and 30 years or older) will be invited to participate in the Unaffected Genetic Cohort.

- Unaffected individuals at high risk of LRRK2, GBA or SNCA mutation due to first degree relative with a LRRK2, GBA or SNCA mutation who choose to remain unaware of their genetic status and are found to have a LRRK2 or GBA mutation and are younger than 45 will not be eligible for enrollment in PPMI. Individuals with SNCA mutation and younger than 30 will be invited to participate in the Genetic Registry.
  - Unaffected individuals at high risk of LRRK2, GBA or SNCA mutation due to first degree relative with a LRRK2, GBA or SNCA mutation who choose to be made aware of their genetic status and are found to have a LRRK2 or GBA mutation and were enrolled in the Genetic Registry previously may transition into the Genetic Cohort provided that the current inclusion criteria is met.

- Unaffected individuals at high risk of LRRK2, GBA or SNCA mutation due to first degree relative with a LRRK2, GBA or SNCA mutation who choose to remain unaware of their genetic status, are found not to have a LRRK2 or GBA mutation and are 45 or older (or SNCA mutation and 30 or older), and have a first degree relative with a LRRK2 or GBA (or SNCA) mutation will be invited to participate in the Unaffected Genetic Cohort.

- Unaffected individuals at high risk of LRRK2, GBA or SNCA mutation due to first degree relative with a LRRK2, GBA or SNCA mutation who choose to remain unaware of their genetic status, are found not to have a LRRK2 or GBA mutation and are younger than 45 and have a first degree relative with a LRRK2 or GBA mutation will not be eligible for enrollment in PPMI. Individuals with SNCA mutation and younger than 30 will be invited to participate in the Genetic Registry
  - Unaffected individuals at high risk of LRRK2, GBA or SNCA mutation due to first degree relative with a LRRK2, GBA or SNCA mutation who choose to be made aware of their genetic status and are found to have a LRRK2 or GBA mutation and were enrolled in the Genetic Registry previously may transition into the Genetic Cohort provided that the current inclusion criteria is met.

- For unaffected individuals from an ethnic or geographic group known to have relatively high risk of LRRK2, GBA or SNCA mutation (such as people of Ashkenazi Jewish or Basques descent) **and** who have a family member (either alive or deceased) who has/had PD must be informed

– These unaffected Individuals must be willing to be informed of their genetic testing results

- Unaffected individuals from an ethnic or geographic group known to have relatively high risk of LRRK2, GBA or SNCA mutation (such as people of

Ashkenazi Jewish or Basques descent) **and** who have a family member (either alive or deceased) who has/had PD who are found to be gene negative will not be eligible for either the Genetic Cohort or Genetic Registry.

- Unaffected individuals from an ethnic or geographic group known to have relatively high risk of LRRK2, GBA or SNCA mutation (such as people of Ashkenazi Jewish or Basques descent) **and** who have a family member (either alive or deceased) who has/had PD who are found to have a LRRK2, GBA or SNCA mutation will be informed of their genetic status.

- Those who are 45 years or older (or SNCA mutation and 30 years or older) will be invited to participate in the Unaffected Genetic Cohort.

- Those who are younger than 45 (or SNCA mutation and younger than 30), will not be eligible for enrollment in PPMI.
  - Unaffected individuals at high risk of LRRK2, GBA or SNCA mutation due to first degree relative with a LRRK2, GBA or SNCA mutation who choose to be made aware of their genetic status and are found to have a LRRK2 or GBA mutation and were enrolled in the Genetic Registry previously may transition into the Genetic Cohort provided that the current inclusion criteria is met.

Note that the LRRK2 Unaffected Genetic Cohort will replace the LRRK2 component of the prodromal cohort. The prodromal cohort will now consist of 100 subjects with RBD, and/or hyposmia.. Note that the GBA cohort will replace 250 LRRK2 subjects. Both the LRRK2 cohort and the GBA cohort now consist of 250 subjects. Note subject with both LRRK2 and GBA mutations will be enrolled using the criteria of a LRRK2 subject.

Please see next page for a schematic of the assignment of PD and unaffected subjects to the Genetic Cohort or Genetic Registry.

Figure 1: Schematic of PD (A) or Unaffected (B) assignment to the Genetic Cohort or Genetic Registry#

Figure 1A: Schematic of PD Subject Assignment to Genetic Cohort and Genetic Registry#

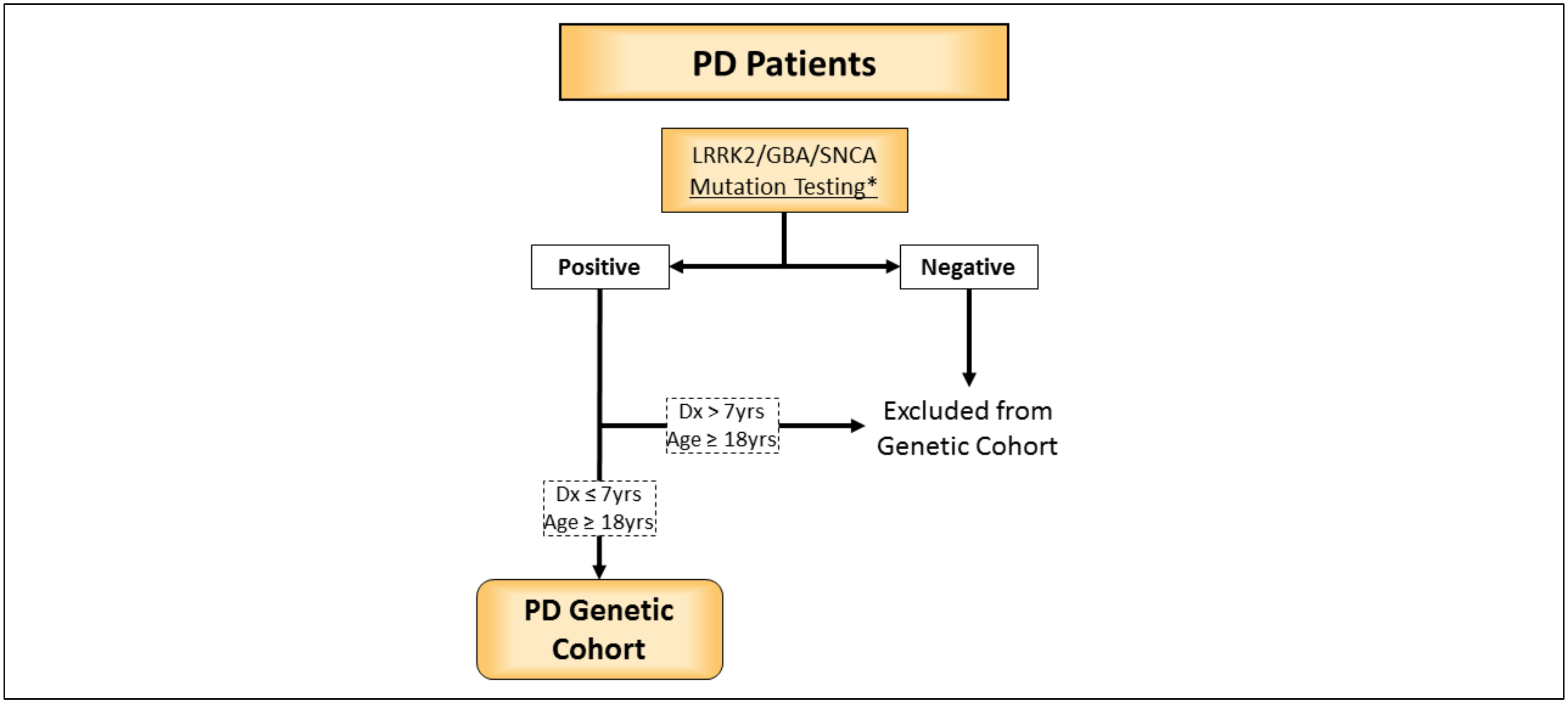

*Subjects are made aware of results. Subjects are excluded if they are unwilling to know results.
Genetic Registry enrollment ending for LRRK2 and GBA subjects with Amendment 12

Figure 1B: Schematic of Unaffected subject assignment to Genetic Cohort or Registry#

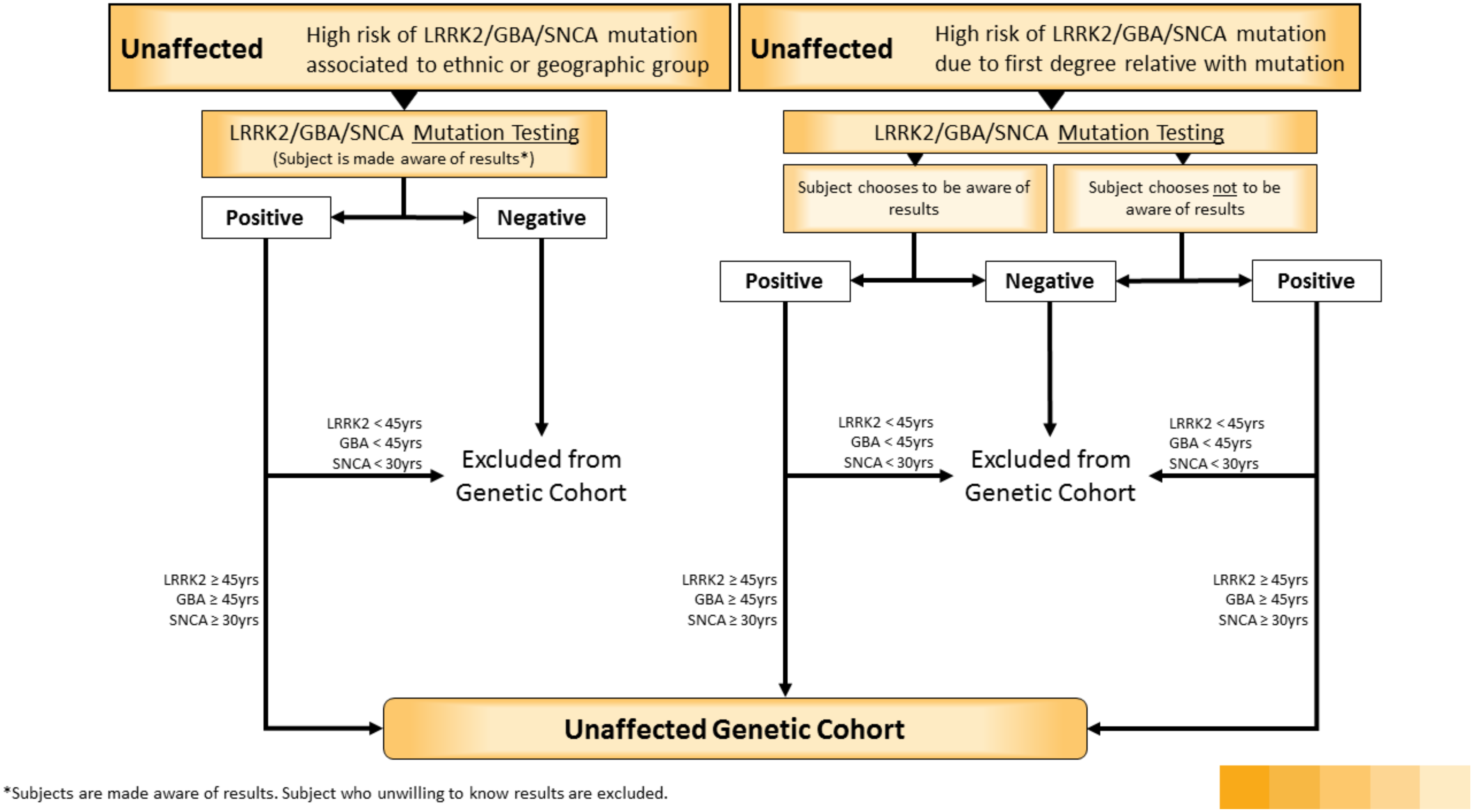

*Subjects are made aware of results. Subjects are excluded if they are unwilling to know results.
Genetic Registry closing with Amendment 13

4.1.1. PPMI Companion Studies

4.1.1.1 **^{18}F-AV-133 VMAT-2 PET imaging** will occur in Australia in place of the dopamine transporter imaging for PD, Control, SWEDD, Prodromal and Genetic Cohort subjects. At selected U.S. sites, participating subjects (PD, HC, SWEDD, Prodromal and Genetic Cohort) may undergo ^{18}F-AV-133 VMAT-2 PET imaging in addition to the dopamine transporter imaging per the schedule of activities. Refer to the ^{18}F-AV-133-PPMI companion protocol.

4.1.1.2 **Evaluation of [^{18}F] florbetaben Positron Emission Tomography (PET) imaging** will occur in approximately 200 PPMI subjects with PD (idiopathic and genetic cohorts), HC, and Prodromal subjects (including RBD, olfaction and genetic subjects). Refer to the *Evaluation of [^{18}F] florbetaben in Subjects Participating in the Parkinson's Progression Markers Initiative* companion protocol.

4.1.1.3 **Skin biopsies** or **blood draws** will be obtained from approximately 200 PPMI subjects with PD, affected and unaffected subjects with a LRRK2, GBA or SNCA mutation, prodromal and healthy control subjects at PPMI sites. The primary objective of this study is to develop a bank of fibroblasts and induced pluripotent stem (iPS) cell lines from skin biopsies and blood samples obtained from PPMI study subjects in order to create a cellular disease model in PD with the goals of 1) furthering understanding of disease origin and underlying causes of PD and 2) enabling future studies of disease-modifying therapies. Sites collecting blood samples will perform associated blood draws at an unscheduled visit separate from routine PPMI study visits or at an interim visit (subjects completing blood draw at an interim visit must forego collection of PBMCs and biomic blood samples at interim visit). Refer to the *Skin Biopsies for the Generation of Induced Pluripotent Stem Cells to Model Parkinson's Disease from the Parkinson's Progression Markers Initiative (PPMI)* companion protocol for further details on procedures for skin biopsies. Refer to *Appendix M (Blood Collection for Induced Pluripotent Stem Cells (iPSCs))* of the PPMI Biologics Manual for further details on procedures related to collection of blood samples.

4.1.1.4 **PPMI Family History Substudy,** a companion protocol, will permit collection of family history from approximately 1200 Genetic Cohort subjects. The aim of the Family History Substudy is to identify individuals participating within the PPMI study who are related to each other so as to create family trees (pedigrees) which delineate the relationship among participating PPMI subjects. Subjects will be given a packet of study

materials approved by the Indiana University School of Medicine IRB. These materials contain a cover letter of introduction, a Consent Reply form with the PPMI subject ID inserted by the PPMI site coordinator, an Informed Consent form, an Authorization to Release Health Information for Research form (HIPAA) and a prepaid return envelope. The PPMI site will inform Indiana University by fax which PPMI subjects (by subject ID only) received the Family History packet. The Indiana University study team will contact subjects who provide informed consent to initiate the completion of the Family History Questionnaire. Refer to *The Parkinson's Progression Markers Initiative (PPMI) Family History Substudy* companion protocol.

4.1.1.5 **Prospective, longitudinal follow-up** of Parkinson's disease, vital status and assessment of life-long environmental exposure histories of PPMI subjects will be accomplished by subject participation in a registry entitled, *Follow-Up of Persons with Neurologic Disease (FOUND)-PPMI.* The registry will be managed by the University of California-San Francisco (UCSF) under their IRB approved protocol. PPMI subjects will be consented to allow the PPMI site to provide the subject's contact information to the UCSF study team, who will then contact subjects to invite them to undergo the informed consent process to join the registry (see section 9.2).

4.1.1.6 **PPMI Brain and Tissue Bank.** An important aspect of Parkinson's Disease research is post-mortem analysis of brain tissue and other types of tissue. Through this analysis, changes in the brain can be examined and correlated with motor, cognitive, and behavioral changes. Brain tissue will help Parkinson's Disease researchers further their understanding of the disease and its progression.

The PPMI Brain and Tissue Bank will be managed by Indiana University under their IRB approved protocol. Enrollment into the PPMI Brain and Tissue Bank may occur in one of two ways. At PPMI sites where donation is able to be implemented through a centralized process, the local site will provide subjects with an information packet explaining the opportunity and process for donating brain and other tissues, upon their death. Subjects will be asked to provide permission to allow the PPMI site to provide the subject's contact information to the Pathology Core study team, who will then contact subjects to discuss tissue donation. Subjects will also be provided contact information for the Pathology Core study team as part of the initial information packet, so subjects may decide to contact the Pathology Core study team on their own. These subjects will be asked to sign an Intent to Donate form approved by the Pathology Core's IRB to document their desire to donate brain tissue and other relevant tissues upon their death.

At PPMI sites where donation is not able to be implemented through a centralized process, the local sites will provide subjects with an information

packet explaining the opportunity and process for donating brain and other tissues and discuss donation directly with subjects. If a subject agrees to donation, the site will ask the subject to sign an Intent to Donate form approved by their local Ethics Committee or IRB to document their desire to donate brain tissue and other relevant tissues upon their death.
PPMI subjects who have previously consented to donate to a brain or tissue bank that is not part of PPMI will be given the option to consent to have any tissue donated to this bank shared with the PPMI study. Subjects may pursue the option of withdrawing their consent with the previous brain bank program.

PPMI subjects who may have withdrawn consent from the PPMI study or completed study activities can donate their tissue and brain to the PPMI study. Inactive PPMI subjects will need to sign a separate advanced directive with the Pathology Core or local site as needed. This data will be linked to their data previously collected as part of the PPMI study.

4.1.1.7 **Characterization of Motor Function and Potential Transition States to Parkinson's Disease in LRRK2 mutation Carriers and Controls.**

Gait disturbances play a major role in the motor manifestation of PD. Traditional assessments aimed at measuring motor deficits, including the UPDRS, do not identify subtle differences in individuals at risk for PD, while quantitative measures have shown promise in this. Thus, some investigators reason that more sensitive tests of motor function are needed to increase the likelihood of identifying pre-diagnosis motor changes. Quantitative measures of gait and mobility should provide a means for assessing pre-diagnosis changes.

Researchers at Tel Aviv Medical Center have completed preliminary work supporting the potential of using quantitative measures of gait and mobility as a means for assessing pre-diagnosis changes. The current sub-study will build on this preliminary work and involve a protocol to obtain longitudinal motor function data at a subset of PPMI sites. A short motor assessment will be administered using a gait system that includes 3 lightweight wireless wearable sensors. The system measures acceleration of movement in 3 orthogonal axes as a function of time. It is anticipated that longitudinal data from this protocol will provide insight into phenoconversion and pre-clinical motor symptoms and inform on dynamic changes of function throughout disease and potential modifiers and mediators of motor symptoms. Refer to the PPMI Characterization of Motor Function and Potential Transition States to Parkinson's Disease in LRRK2 mutation Carriers and Controls companion protocol.

4.1.1.8 **Passive monitoring of individuals through wearable sensors or non-invasive objective measure (Sensor-PPMI).**

This observational study will determine the feasibility and value of using multiple wearable sensors (e.g., watch, wearable sensors or other non-

invasive measures) to identify clinical and sub-clinical characteristics (e.g., changes in activity, movement, sleep, socialization) that are present in participants with PD across the spectrum of disease severity as well as among individuals with possible prodromal Parkinson disease based on either symptoms (e.g., hyposmia and/or RBD) or genetic status (e.g., LRRK2 and GBA mutation carriers). Participants at centers with individuals with prodromal PD, PD and healthy controls will undergo their standard assessments as part of the PPMI study. In addition, they may be provided a watch or other non-invasive device for use for the duration of the study and asked to wear sensors or another type of non-invasive device on their limbs and trunk. The watch, sensor or device will be worn as frequently as desired by participants but ideally most of the time (including while sleeping). Participants will wear the watch and/or sensors or device during their in-person PPMI assessments and may have at least the examination portion of such visits video recorded.

A group of age matched healthy control subjects from the PPMI cohort may also participate in order to evaluate differences between these groups. At the study's conclusion, participants will complete a survey about their experiences in the study. Passive monitoring in PPMI may include more than one sub-study to evaluate devices. Refer to the **Passive monitoring of individuals through wearable sensors or non-invasive methods (Sensor-PPMI sub-study)** companion protocol for further details.

4.2. Selection of Study Population

4.2.1. Inclusion Criteria (Parkinson Disease Subjects)

4.2.1.1. Patients must have at least two of the following: resting tremor, bradykinesia, rigidity (must have either resting tremor or bradykinesia); OR either asymmetric resting tremor or asymmetric bradykinesia.
4.2.1.2. A diagnosis of Parkinson disease for 2 years or less at Screening.
4.2.1.3. Hoehn and Yahr stage I or II at Baseline.
4.2.1.4. Not expected to require PD medication within at least 6 months from Baseline.
4.2.1.5. Male or female age 30 years or older at time of PD diagnosis.
4.2.1.6. Confirmation from imaging core that screening dopamine transporter SPECT scan is consistent with dopamine transporter deficit (or for sites where DaTSCAN™ is not available that VMAT-2 PET scan is consistent with VMAT deficit).
4.2.1.7. Ability to provide written informed consent in accordance with Good Clinical Practice (GCP), International Conference on Harmonization (ICH), and local regulations.
4.2.1.8. Willing and able to comply with scheduled visits, required study procedures and laboratory tests.
4.2.1.9. Women may not be pregnant, lactating or planning pregnancy during the course of the study.

- Includes a negative urine pregnancy test on day of Screening scan prior to injection (DaTSCAN^{TM} and/or ^{18}F-AV-133).
- Includes a negative serum pregnancy test prior to Screening scan injection (^{18}F-AV-133 only).

4.2.1.10. Women participating in VMAT-2 PET imaging must be of non-childbearing potential **or** be using a highly effective method of birth control 14 days prior to until at least 24 hours after injection of ^{18}F-AV-133).

- Non-child bearing potential is defined as a female that must be either postmenopausal (no menses for at least 12 months prior to Screening) or surgically sterile (bilateral tubal ligation, bilateral oophorectomy or hysterectomy).
- Highly effective method of birth control is defined as practicing at least one of the following: A birth control method that results in a less than 1% per year failure rate when used consistently and correctly, such as oral contraceptives for at least 3 months prior to injection, an intrauterine device (IUD) for at least 2 months prior to injection, or barrier methods, e.g., diaphragm or combination condom and spermicide. Periodic abstinence (e.g. calendar, ovulation, symptothermal, post-ovulation methods) is not acceptable.

4.2.2. Exclusion Criteria (Parkinson Disease Subjects)

4.2.2.1. Atypical PD syndromes due to either drugs (e.g., metoclopramide, flunarizine, neuroleptics) or metabolic disorders (e.g., Wilson’s disease), encephalitis, or degenerative diseases (e.g., progressive supranuclear palsy).

4.2.2.2. Currently taking levodopa, dopamine agonists, MAO-B inhibitors (e.g., selegiline, rasagiline), amantadine or other PD medication.

4.2.2.3. Has taken levodopa, dopamine agonists, MAO-B inhibitors or amantadine within 60 days of Baseline.

4.2.2.4. Has taken levodopa or dopamine agonists prior to Baseline for more than a total of 60 days.

4.2.2.5. A clinical diagnosis of dementia[63] as determined by the investigator (Appendix 1).

4.2.2.6. Received any of the following drugs that might interfere with dopamine transporter SPECT imaging: Neuroleptics, metoclopramide, alpha methyldopa, methylphenidate, reserpine, or amphetamine derivative, within 6 months of Screening.

4.2.2.7. Subjects participating in VMAT-2 PET imaging have received any of the following medications that might interfere with ^{18}F-AV-133 PET imaging: neuroleptics, metoclopramide, alpha methyldopa, methylphenidate, reserpine, or amphetamine derivative, within 2 weeks prior to the Screening ^{18}F-AV-133 injection.

4.2.2.8. Current treatment with anticoagulants (e.g., coumadin, heparin) that might preclude safe completion of the lumbar puncture.

4.2.2.9. Condition that precludes the safe performance of routine lumbar puncture, such as prohibitive lumbar spinal disease, bleeding diathesis, or clinically significant coagulopathy or thrombocytopenia.

4.2.2.10. Any other medical or psychiatric condition or lab abnormality, which in the opinion of the investigator might preclude participation.

4.2.2.11. Use of investigational drugs or devices within 60 days prior to Baseline (dietary supplements taken outside of a clinical trial are not exclusionary, e.g., coenzyme Q10).

4.2.2.12. Previously obtained MRI scan with evidence of clinically significant neurological disorder (in the opinion of the Investigator).

4.2.3. Inclusion Criteria (Healthy Control Subjects)

4.2.3.1. Male or female age 30 years or older at Screening.

4.2.3.2. Ability to provide written informed consent in accordance with Good Clinical Practice (GCP), International Conference on Harmonization (ICH), and local regulations.

4.2.3.3. Willing and able to comply with scheduled visits, required study procedures and laboratory tests.

4.2.3.4. Women may not be pregnant, lactating or planning pregnancy during the course of the study.

- Includes a negative urine pregnancy test on day of Screening scan prior to injection (DaTSCAN™ and/or ^{18}F-AV-133).
- Includes a negative serum pregnancy test prior to Screening scan injection (^{18}F-AV-133 only).

4.2.3.5. Women participating in VMAT-2 PET imaging must be of non-childbearing potential **or** be using a highly effective method of birth control 14 days prior to until at least 24 hours after injection of ^{18}F-AV-133).

- Non-child bearing potential is defined as a female that must be either postmenopausal (no menses for at least 12 months prior to Screening) or surgically sterile (bilateral tubal ligation, bilateral oophorectomy or hysterectomy).
- Highly effective method of birth control is defined as practicing at least one of the following: A birth control method that results in a less than 1% per year failure rate when used consistently and correctly, such as oral contraceptives for at least 3 months prior to injection, an intrauterine device (IUD) for at least 2 months prior to injection, or barrier methods, e.g., diaphragm or combination condom and spermicide. Periodic abstinence (e.g. calendar, ovulation, symptothermal, post-ovulation methods) is not acceptable.

4.2.4. Exclusion Criteria (Healthy Control Subjects)

4.2.4.1. Current or active clinically significant neurological disorder (in the opinion of the Investigator).
4.2.4.2. First degree relative with idiopathic PD (parent, sibling, child).
4.2.4.3. MoCA score of 26 or less (i.e., eligible if score is 27 to 30).
4.2.4.4. Received any of the following drugs that might interfere with dopamine transporter SPECT imaging: Neuroleptics, metoclopramide, alpha methyldopa, methylphenidate, reserpine, or amphetamine derivative, within 6 months of Screening.
4.2.4.5. Subjects participating in VMAT-2 PET imaging have received any of the following medications that might interfere with ^{18}F-AV-133 PET imaging: neuroleptics, metoclopramide, alpha methyldopa, methylphenidate, reserpine, or amphetamine derivative, within 2 weeks prior to the Screening ^{18}F-AV-133 injection.
4.2.4.6. Current treatment with anticoagulants (e.g., coumadin, heparin) that might preclude safe completion of the lumbar puncture.
4.2.4.7. Condition that precludes the safe performance of routine lumbar puncture, such as prohibitive lumbar spinal disease, bleeding diathesis, or clinically significant coagulopathy or thrombocytopenia.
4.2.4.8. Any other medical or psychiatric condition or lab abnormality, which in the opinion of the investigator might preclude participation.
4.2.4.9. Use of investigational drugs or devices within 60 days prior to baseline (dietary supplements taken outside of a clinical trial are not exclusionary, e.g., coenzyme Q10).
4.2.4.10. Previously obtained MRI scan with evidence of clinically significant neurological disorder (in the opinion of the Investigator).

4.2.5. Inclusion Criteria (SWEDD Subjects)

See Section 4.2.1 for Inclusion Criteria for PD subjects. All criteria apply except a SWEDD subject must have confirmation from imaging core that screening dopamine transporter SPECT scan shows no evidence of dopamine transporter deficit (or for sites where DaTSCAN™ is not available that VMAT-2 PET scan shows no evidence of VMAT deficit).

4.2.6. Exclusion Criteria (SWEDD Subjects)

See Section 4.2.2 for Exclusion Criteria for PD subjects which also apply to SWEDD subjects.

4.2.7. Inclusion Criteria (Prodromal Subjects)

4.2.7.1. Subjects must have at least one of the following characteristics:

Hyposmia:

a) Male or female age 60 years or older
b) Confirmation from olfactory core that olfaction as determined by UPSIT is at or below the 10th percentile by age and gender

REM Behavior Disorder (RBD):

a) Male or female age 60 years or older
b) Confirmation from sleep core that subject's Polysomnography (PSG) meets criteria for RBD and/or clinical diagnosis of RBD by site investigator including existing PSG.

4.2.7.2. Confirmation from imaging core that screening dopamine transporter SPECT scan (or V-MAT-2 PET scan for sites where DaTSCAN^{TM} is not available) is read as eligible (see below)

- About 80 subjects will have a range of DAT deficit similar to subjects with early PD (mild to moderate DAT deficit). About 20 subjects will be selected with no DAT deficit or minimal DAT deficit similar in age, gender, and risk profile to those with mild to moderate DAT deficit.

4.2.7.3. Ability to provide written informed consent in accordance with Good Clinical Practice (GCP), International Conference on Harmonization (ICH), and local regulations.

4.2.7.4. Willing and able to comply with scheduled visits, required study procedures and laboratory tests.

4.2.7.5. Women may not be pregnant, lactating or planning pregnancy during the course of the study.

- Includes a negative urine pregnancy test on day of screening scan prior to injection (DaTSCAN^{TM}).
- Includes a negative serum pregnancy test prior to Screening scan injection (^{18}F-AV-133 only).

4.2.7.6. Women participating in VMAT-2 PET imaging must be of non-childbearing potential **or** be using a highly effective method of birth control 14 days prior to until at least 24 hours after injection of ^{18}F-AV-133).

- Non-child bearing potential is defined as a female that must be either postmenopausal (no menses for at least 12 months prior to Screening) or surgically sterile (bilateral tubal ligation, bilateral oophorectomy or hysterectomy).
- Highly effective method of birth control is defined as practicing at least one of the following: A birth control method that results in a less than 1% per year failure rate when used consistently and correctly, such as oral contraceptives for at least 3 months prior to injection, an intrauterine device (IUD) for at least 2 months prior to injection, or barrier methods, e.g., diaphragm or combination condom and spermicide. Periodic abstinence (e.g. calendar, ovulation, symptothermal, post-ovulation methods) is not acceptable.

4.2.8. Exclusion Criteria (Prodromal Subjects)

4.2.8.1. Current or active clinically significant neurological disorder or psychiatric disorder (in the opinion of the Investigator).

4.2.8.2. GDS score greater than or equal to 10 (GDS score of 5 – 9 requires Investigator discretion to enter study).

4.2.8.3. STAI Form Y-1 greater than or equal to 54 requires Investigator discretion to enter study.
4.2.8.4. A clinical diagnosis of dementia[63] as determined by the investigator (Appendix 1).
4.2.8.5. A clinical diagnosis of Parkinson disease at the Screening visit as determined by the Investigator.
4.2.8.6. Received any of the following drugs that might interfere with dopamine transporter SPECT imaging: Neuroleptics, metoclopramide, alpha methyldopa, methylphenidate, reserpine, or amphetamine derivative, within 6 months of Screening.
4.2.8.7. Current treatment with anticoagulants (e.g., coumadin, heparin) that might preclude safe completion of the lumbar puncture.
4.2.8.8. Condition that precludes the safe performance of routine lumbar puncture, such as prohibitive lumbar spinal disease, bleeding diathesis, or clinically significant coagulopathy or thrombocytopenia.
4.2.8.9. Any other medical or psychiatric condition or lab abnormality, which in the opinion of the investigator might preclude participation.
4.2.8.10. Use of investigational drugs or devices within 60 days prior to Baseline (dietary supplements taken outside of a clinical trial are not exclusionary, e.g., coenzyme Q10).
4.2.8.11. Previously obtained MRI scan with evidence of clinically significant neurological disorder (in the opinion of the Investigator).

4.2.9. Inclusion criteria (Genetic Cohort – PD Subjects)

4.2.9.1. Patients must have at least two of the following: resting tremor, bradykinesia, rigidity (must have either resting tremor or bradykinesia); OR either asymmetric resting tremor or asymmetric bradykinesia
4.2.9.2. Parkinson disease diagnosis for 7 years or less at Screening
4.2.9.3. Hoehn and Yahr stage less than 4 at Baseline.
4.2.9.4. Male or female age 18 years or older at baseline.
4.2.9.5. Confirmation of causative LRRK2, GBA or SNCA mutation (willingness to undergo genetic testing as part of the prescreening or documentation of prior genetic testing results).
4.2.9.6. Ability to provide written informed consent in accordance with Good Clinical Practice (GCP), International Conference on Harmonization (ICH), and local regulations.
4.2.9.7. Willing and able to comply with scheduled visits, required study procedures and laboratory tests.
4.2.9.8. For subjects taking any of the following drugs (Neuroleptics, metoclopramide, alpha methyldopa, methylphenidate, reserpine, or amphetamine derivative) must be willing and medically able to hold the medication for at least 5 half-lives before DaTSCAN™ imaging.
4.2.9.9. Women who are pregnant or lactating will not undergo DaTSCAN™ and/or ^{18}F-AV-133 imaging assessments. (The imaging assessments that would have occurred during this time will be waived)

- Includes a negative urine (or serum if required by site) pregnancy test on day of Screening scan prior to injection (DaTSCAN™ and/or ^{18}F-AV-133).
- Includes a negative serum pregnancy test prior to Screening scan injection (^{18}F-AV-133 only).

4.2.9.10. Women participating in VMAT-2 PET imaging must be of non-childbearing potential **or** be using a highly effective method of birth control 14 days prior to until at least 24 hours after injection of ^{18}F-AV-133).

- Non-child bearing potential is defined as a female that must be either postmenopausal (no menses for at least 12 months prior to Screening) or surgically sterile (bilateral tubal ligation, bilateral oophorectomy or hysterectomy).
- Highly effective method of birth control is defined as practicing at least one of the following: A birth control method that results in a less than 1% per year failure rate when used consistently and correctly, such as oral contraceptives for at least 3 months prior to injection, an intrauterine device (IUD) for at least 2 months prior to injection, or barrier methods, e.g., diaphragm or combination condom and spermicide. Periodic abstinence (e.g. calendar, ovulation, symptothermal, post-ovulation methods) is not acceptable.

4.2.10. Exclusion Criteria (Genetic Cohort- PD Subjects)

4.2.10.1. A clinical diagnosis of dementia[63] as determined by the investigator (Appendix 1).

4.2.10.2. Subjects participating in VMAT-2 PET imaging have received any of the following medications that might interfere with ^{18}F-AV-133 PET imaging: neuroleptics, metoclopramide, alpha methyldopa, methylphenidate, reserpine, or amphetamine derivative, within 2 weeks prior to the Screening ^{18}F-AV-133 injection.

4.2.10.3. Current treatment with anticoagulants (e.g., coumadin, heparin) that might preclude safe completion of the lumbar puncture.

4.2.10.4. Condition that precludes the safe performance of routine lumbar puncture, such as prohibitive lumbar spinal disease, bleeding diathesis, or clinically significant coagulopathy or thrombocytopenia.

4.2.10.5. Any other medical or psychiatric condition or lab abnormality, which in the opinion of the investigator might preclude participation.

4.2.10.6. Previously obtained MRI scan with evidence of clinically significant neurological disorder (in the opinion of the Investigator).

4.2.11. Inclusion Criteria (Genetic Cohort- Unaffected Subjects)

4.2.11.1. Male or female age 45 years or older at baseline with a LRRK2 or GBA mutation and/or a first degree relative with a LRRK2 or GBA mutation

OR

4.2.11.2. Male or female age 30 years or older at baseline with a SNCA mutation and/or a first degree relative with a SNCA mutation

4.2.11.3. Willingness to undergo genetic testing

- unaffected subjects at high risk of LRRK2, GBA or SNCA mutation due to first degree relative with a LRRK2, GBA or SNCA mutation may choose either to be informed of the results or remain unaware of the results
- unaffected subjects from an ethnic or geographic group known to have relatively high risk of LRRK2, GBA or SNCA mutation (such as people of Ashkenazi Jewish or Basques descent) **and** who have a family member (either alive or deceased) who has/had PD must be willing to be informed of their own testing results

4.2.11.4. Ability to provide written informed consent in accordance with Good Clinical Practice (GCP), International Conference on Harmonization (ICH), and local regulations.

4.2.11.5. Willin**g a**nd able to comply with scheduled visits, required study procedures and laboratory tests.

4.2.11.6. For subjects taking any of the following drugs (Neuroleptics, metoclopramide, alpha methyldopa, methylphenidate, reserpine, or amphetamine derivative) must be willing and medically able to hold the medication for at least 5 half-lives before DaTSCAN™ imaging.

4.2.11.7. Women who are pregnant or lactating will not undergo DaTSCAN™ and/or ^{18}F-AV-133 imaging assessments. (The imaging assessments that would have occurred during this time will be waived)

- Includes a negative urine (or serum if required by site) pregnancy **test on day of Screening scan prior to injection (DaTSCAN™ and/or ^{18}F-AV-133).**
- Includes a negative serum pregnancy test prior to Screening scan injection (^{18}F-AV-133 only).

4.2.11.8 Women participating in VMAT-2 PET imaging must be of non-childbearing potential **or** be using a highly effective method of birth control 14 days prior to until at least 24 hours after injection of ^{18}F-AV-133).

- Highly effective method of birth control is defined as practicing at least one of the following: A birth control method that results in a less than 1% per year failure rate when used consistently and correctly, such as oral contraceptives for at least 3 months prior to injection, an intrauterine device (IUD) for at least 2 months prior to injection, or barrier methods, e.g., diaphragm or combination condom and spermicide. Periodic abstinence (e.g. calendar, ovulation, symptothermal, post-ovulation methods) is not acceptable.
- Non-child bearing potential is defined as a female that must be either postmenopausal (no menses for at least 12 months prior to Screening) or surgically sterile (bilateral tubal ligation, bilateral oophorectomy or hysterectomy).

4.2.12. Exclusion Criteria (Genetic Cohort- Unaffected Subjects)

4.2.12.1. A clinical diagnosis of PD
4.2.12.2. A clinical diagnosis of dementia[63] as determined by the investigator (Appendix 1).
4.2.12.3. GDS score greater than or equal to 10 (GDS score of 5 – 9 requires Investigator discretion to enter study).
4.2.12.4. STAI Form Y-1 greater than or equal to 54 requires Investigator discretion to enter study.
4.2.12.5. Subjects participating in VMAT-2 PET imaging have received any of the following medications that might interfere with ^{18}F-AV-133 PET imaging: neuroleptics, metoclopramide, alpha methyldopa, methylphenidate, reserpine, or amphetamine derivative, within 2 weeks prior to the Screening ^{18}F-AV-133 injection.
4.2.12.6. Current treatment with anticoagulants (e.g., coumadin, heparin) that might preclude safe completion of the lumbar puncture.
4.2.12.7. Condition that precludes the safe performance of routine lumbar puncture, such as prohibitive lumbar spinal disease, bleeding diathesis, or clinically significant coagulopathy or thrombocytopenia.
4.2.12.8. Any other medical or psychiatric condition or lab abnormality, which in the opinion of the investigator might preclude participation.
4.2.12.9. Previously obtained MRI scan with evidence of clinically significant neurological disorder (in the opinion of the Investigator).

Note: New enrollments of LRRK2 or GBA mutation carriers into the Genetic Registry are being ceased with amendment 12. Subjects who are not eligible for enrollment into the Genetic Cohort will not be eligible for participation in PPMI.

4.2.13. Inclusion Criteria (Genetic Registry)

4.2.13.1. Male or female age 18 years or older.
4.2.13.2. Individual with a SNCA mutation or with a first degree relative having a SNCA mutation
4.2.13.3. Willingness to undergo genetic testing, but may choose either to be informed of the results or remain unaware of the results
4.2.13.4. Ability to provide written informed consent in accordance with Good Clinical Practice (GCP), International Conference on Harmonization (ICH), and local regulations.
4.2.13.5. Willing and able to comply with scheduled visits, required study procedures and laboratory tests.

4.2.14 Exclusion Criteria (Genetic Registry)

4.2.14.1 A clinical diagnosis of dementia[63] as determined by the investigator (Appendix 1).
4.2.14.2. Any other medical or psychiatric condition or lab abnormality, which in the opinion of the investigator might preclude participation.

4.2.14.3. Previously obtained MRI scan with evidence of clinically significant neurological disorder (in the opinion of the Investigator).

4.3 Age and Gender Matching

Enrollments will be monitored centrally by the Steering Committee with the goal of achieving age and gender balance across the PD and Healthy Control subjects. Individual sites should generally attempt to match healthy control subjects as closely as possible in age (target within 5 years) and gender to the PD subjects enrolled at the site. Sites will be instructed if recruitment restrictions need to be implemented as the study progresses in order to maintain a balanced population.

5. Investigational Plan

5.1. Subject Identification Numbers

5.1.1 Subject Identification (ID) Number

A Subject ID Number will be assigned in sequential order by the site from a list provided to the site by the CTCC. This 4-digit number for PD, HC and SWEDD subjects will be used to identify the subject on all study forms and lab specimens.

The Prodromal subjects will be assigned a 5-digit subject ID number assigned in sequential order from a list provided to the site and appropriate cores by the CTCC. This 5-digit number for Prodromal subjects will be used to identify the subject on all study forms and lab specimens.

Subjects who participate in the Genetic Cohort (PD and unaffected) and the Genetic Registry will receive a Subject ID Number which will be assigned in sequential order by the site from a list provided to the site by the CTCC. This 5-digit number will be used to identify the subject on all study forms and lab specimens.

5.1.2 CTCC Unique ID Number

Subjects will be instructed how to obtain a 9-digit Unique Identification Number at the Screening Visit. This ID system has the ability to track individual subjects across multiple CTCC studies without storing any personally identifiable information. The protected system uses an algorithm of nine data element inputs (last name at birth, first name at birth, gender at birth, day, month and year of birth, city and country of birth, and mother's maiden name), and produces an electronic "fingerprint" output. The system stores only the "fingerprint" and clears the individual's inputted data elements from memory. The subject is then assigned a 9-digit CTCC Unique ID Number that is associated with their electronic "fingerprint."

Once a subject signs the informed consent, the subject and the study coordinator or designated study staff will go to a secure website on a computer at the research

clinic and enter the subject's nine data elements. The CTCC Unique ID Number will be printed and provided to the subject. The study coordinator will record this number on the CTCC Unique ID CRF.

If a subject has participated in previous CTCC studies and already has an existing CTCC Unique ID Number, that number should be used for this study. The site can regenerate a subject's CTCC Unique ID Number by returning to the secure website, enter the same nine data elements in the exact same way they were entered the first time to receive the same CTCC Unique ID Number.

5.2 Schedule of Activities

Refer to the protocol synopsis Schedule of Activities that summarizes the assessments to be conducted at each visit.

Sites participating in ^{18}F-AV-133 PET imaging refer to the ^{18}F-AV-133-PPMI companion protocol Trial Flow Charts for the schedule of activities and assessments to be conducted at each visit.

Sites participating in [^{18}F] florbetaben PET imaging will refer to the companion protocol, entitled, ***Evaluation of [^{18}F] florbetaben in Subjects Participating in the Parkinson's Progression Markers Initiative***, for the schedule of activities and assessments to be conducted at an applicable study visit.

PPMI sites participating in the collection of skin biopsies refer to the companion protocol entitled, ***Skin Biopsies for the Generation of Induced Pluripotent Stem Cells to Model Parkinson's disease from the Parkinson's Progression Markers Initiative (PPMI)***, for the schedule of activities and assessments to be conducted at an applicable study visit. Refer to the main PPMI protocol synopsis Schedule of Activities for subjects participating in the collection of a blood sample for the generation of induced pluripotent stem cells study.

Refer to the schedules of activities (all cohorts) for initiating subject referral for the ***Follow Up of Persons with Neurologic Disease (FOUND) in PPMI*** and the PPMI Brain and Tissue Bank companion protocols, and to the FOUND and Genetic Cohort schedules of activities for those pertaining to the Family History Study companion protocol.

Sites participating in the ***Characterization of Motor Function and Potential Transition States to Parkinson's Disease in LRRK2 mutation Carriers and Controls*** study will refer to that companion protocol for the schedule of activities and assessments to be conducted at applicable study visits.

Sites participating in the ***Passive monitoring of individuals through wearable sensors or non-invasive methods (Sensor-PPMI sub-study)*** study will refer to that companion protocol for the schedule of activities and assessments to be conducted at applicable study visits.

SWEDD Subjects
SWEDD subjects who completed a PPMI Screening visit 12 months ago or longer will be re-screened with the SWEDD Screening visit activities. SWEDD subjects who completed a PPMI Screening visit less than 12 months ago will proceed with the SWEDD Baseline visit activities. Subjects who were enrolled in PPMI and were then determined to be a SWEDD based on their delayed initial SPECT scan, will proceed with the next follow up study visit as scheduled (e.g., subject completed SPECT scan at PPMI V01 and was deemed a SWEDD, the subject will resume participation at V02 using the SWEDD schedule of activities). SWEDD subjects may be asked to continue follow up through up to V15/Month 96 at the investigator's discretion, if by clinical assessment (e.g., if subject had a positive scan at or before Month 24), the investigator determines there is substantial evidence that the subject may have PD.

Prodromal Subjects
Subjects being considered for participation in the Prodromal cohort will undergo pre-screening activities including self-administered olfactory testing using the UPSIT (University of Pennsylvania Smell Identification Test), and/or review of already acquired polysomnography (PSG) for signs of REM behavior disorder (RBD). Subjects who meet the criteria for hyposmia, and/or RBD will be invited to a PPMI clinical research site to review the PPMI consent. Subjects consenting to participate in PPMI will complete DAT imaging as part of the Screening visit.

Genetic Cohort
Subjects being considered for participation in the Genetic Cohort will undergo pre-screening activities including genetic testing and genetic counseling. Subjects who meet the criteria will be invited to participate in the Genetic Cohort for up to 10 years.

5.3 Study Procedures at Each Visit (PD, Healthy, SWEDD, Prodromal, Genetic Cohort and Genetic Registry)

Subjects will undergo all procedures as outlined in the sections below for each cohort. Assessments that require completion by the Site Investigator (unless otherwise approved and delegated) include: Neurological Exam, MDS-UPDRS Part Ia (coordinator may conduct if requested in advance, as long as the assessment is completed consistently for all subjects/all visits), Part III, Part IV, Hoehn & Yahr Stage, Modified Schwab & England ADL, Cognitive Categorization, Clinical Diagnosis and Management Questionnaire, Primary Diagnosis, Diagnostic Questionnaire and Diagnostic Features.

Specific procedures for the clinical labs, biomic labs, imaging, neuropsychological testing and lumbar puncture are indicated in section 6 and corresponding operation manuals.

Sites participating in ^{18}F-AV-133 PET imaging refer to ^{18}F-AV-133-PPMI companion protocol for study procedures required in addition to those detailed below.

5.3.1. Pre-Screening Prodromal:

Pre-screening activities discussed in this section apply only to subjects that have been recruited to participate in the Prodromal cohort. The pre-screening activities are completed to determine if subjects may be eligible to participate in the PPMI Screening visit. There are two types of pre-screening activities through which subjects may become eligible and include: hyposmia based on olfactory testing, and polysomnographic evidence of RBD . The recruitment methods and pre-screening activities are detailed below.

- **Hyposmia:** Recruitment and pre-screening activities for hyposmic subjects will occur centrally through the Olfactory Core based at the Institute for Neurodegenerative Disorders (IND). Subjects will be recruited through mailings, newsletters, Fox Trial Finder, and the PPMI website. Interested subjects will be asked to complete a survey and answer questions related to conditions that may impact their sense of smell. Subjects returning the survey who do not have a condition that could account for the hyposmia will be sent an Informed Consent Form (ICF), an UPSIT to evaluate smell identification, and a brief Self Reporting Questionnaire (SRQ). Once completed, subjects will return the ICF, SRQ and UPSIT by mail to IND. The UPSIT will be scored and compared to a normative database based on age and gender. Subjects who score at or below the 10th percentile for age and gender may be eligible for participation in the PPMI Screening visit. The hyposmic subjects will be notified by phone or mail that they will be contacted by a PPMI clinical site to further discuss possible study participation. Subjects who are not eligible based on their UPSIT score will be contacted by IND and informed of their ineligibility.
- **RBD:** Recruitment and pre-screening activities for subjects with a diagnosis of RBD will occur at selected PPMI sites that have a collaborative relationship with a local sleep center. Sleep Center/Clinic Investigators and staff will review polysomnograms (PSG) obtained in patients who have completed recent sleep studies. Patients that have evidence for RBD based on their PSG will be approached by the Sleep physician or staff who will provide information to the patient about PPMI and ask permission to provide their contact information to the PPMI Investigator or Coordinator. PPMI Investigators and Coordinators will contact patients to obtain initial consent and permission to obtain their recently acquired PSG to determine if they might be eligible for participation in PPMI. Once consent is obtained (oral or written), the PSG may be sent to a central reading lab to determine if the PSG meets criteria for RBD as established for PPMI or may be read at the clinical site. If the PSG meets RBD criteria the subject is re-contacted and asked to come into the clinic and further discuss possible study participation. Subjects that are not eligible based on PSG are contacted and informed of their ineligibility.

Note that LRRK2 pre-screening will now be conducted as part of pre-screening for the Genetic Cohort or Genetic Registry (5.3.2)

5.3.2. Pre-Screening Genetic Cohort:

Prescreening and genetic testing for the Genetic Cohort and Genetic Registry: Recruitment and pre-screening activities for subjects with LRRK2, GBA or SNCA mutations will occur at all PPMI sites. Pre-screening activities will involve genetic testing and counseling. Refer to section 4.1 for a listing of individuals who may be recruited.

After informed consent, subjects will undergo genetic testing and have the opportunity for genetic counseling at Pre-screening visit 1. Up to two samples (blood or saliva) will be collected from each subject for DNA analysis. Either one or both samples may be used in the testing process. Genetic testing results will be available to the PPMI Genetic Coordination Core. Subjects eligible to participate in the Genetic Cohort will return to the PPMI site following genetic testing for Pre-screening visit 2. PPMI sites will be instructed by the Genetic Coordination Core to request that subjects participate in the Genetic Cohort based on their eligibility. At Pre-screening visit 2, all PD subjects and those unaffected subjects who are willing to be informed will learn and have an opportunity to discuss the results of their genetic testing and may receive additional genetic counseling. Those subjects already tested at an approved laboratory can provide documentation of their test results; if approved, these subjects would not require genetic testing at pre-screening visit 1. Subjects agreeing to participate in PPMI will undergo PPMI screening and study visits as detailed below.

Study visits for PD, Healthy, SWEDD, Prodromal, and Genetic Cohort

5.3.3. Screening Visit:

All subjects will undergo a screening evaluation prior to the Baseline visit. This evaluation will include the following activities and will take about 8 hours to complete:

- An explanation of the purpose, procedures, potential risks and benefits of this study and informed consent will be obtained
- As part of the consenting process, an explanation of the purpose and procedures for the Advance Directive for Clinical Research Participation will be given; subjects may take time to review and complete at a subsequent visit (may be done at a later visit for active subjects if screening visit has already occurred); the investigator will review subjects' continuing ability to give informed consent at each in-person visit
- As part of the consenting process, an explanation of FOUND in PPMI will be given; subjects will be asked permission to have their contact information sent to the FOUND coordinating site at UCSF so that UCSF can contact them about their interest in participation (complete at next visit for active subjects if screening visit has already occurred)
- As part of the consenting process, an explanation of the PPMI Brain and Tissue Bank substudy will be discussed and subjects will be provided with an information packet. At some sites, subjects will be asked permission to have their contact information sent to the PPMI Brain and Tissue Bank (complete at

next visit for active subjects if screening visit has already occurred). At other sites, following an explanation of the PPMI Brain and Tissue Bank substudy, subjects will be provided with an information packet and Intent to Donate form. Subjects wishing to sign the Intent to Donate form may do so at the time of screening. If subject is undecided, site should reapproach subject at next visit (or at site's discretion).

- Review of the subject's medical and family history
- Review of concomitant medications
- Vital signs (blood pressure, heart rate and temperature)
- General physical examination
- General neurological examination
- Movement Disorder Society Unified Parkinson Disease Rating Scale (MDS-UPDRS) Parts I- III (PD, Prodromal and Genetic Cohort subjects only) (Part IV also conducted for subjects who have started PD medication)
- Hoehn and Yahr (PD, Prodromal and Genetic Cohort subjects only)
- Modified Schwab & England (PD, Prodromal, and Genetic Cohort subjects only)
- Clinical diagnosis assessment
- Montreal Cognitive Assessment (MoCA)
- Geriatric Depression Scale (GDS-15) (Prodromal Subjects only)
- State-Trait Anxiety Inventory (Prodromal Subjects only)
- Collect blood sample for DNA (may be collected at Baseline if not done at Screening)
- Clinical laboratory assessments
    - Includes serum pregnancy test for women of childbearing potential participating in VMAT-2 PET imaging (see ^{18}F-AV-133-PPMI companion protocol)
- Dopamine transporter SPECT imaging scan (see Section 6.3.1), includes urine (or serum if required by site) pregnancy test for women of childbearing potential prior to injection
- ECG for subjects participating in VMAT-2 PET imaging (see ^{18}F-AV-133-PPMI companion protocol)
- VMAT-2 PET imaging scan, at selected sites (see Section 6.3.2)
- Review of adverse events in follow up to SPECT (and/or VMAT-2 PET) imaging
- A review of the inclusion/exclusion criteria to confirm that the subject is eligible to continue to the Baseline visit
- Consent/withdrawal of consent for contact regarding future studies related or unrelated to PPMI

<u>SWEDD Re-Screen Visit:</u> Conducted for a SWEDD subject who completed a PPMI Screening visit 12 months ago or longer.

- An explanation of the purpose, procedures, potential risks and benefits of this study and informed consent will be obtained
- As part of the consenting process, an explanation of the purpose and procedures for the Advance Directive for Clinical Research Participation will be given;

subjects may take time to review and complete at a subsequent visit (may be done at a later visit for active subjects if screening visit has already occurred); the investigator will review subjects' continuing ability to give informed consent at each in-person visit

- As part of the consenting process, an explanation of FOUND in PPMI will be given; subjects will be asked permission to have their contact information sent to the FOUND coordinating site at UCSF so that UCSF can contact them about their interest in participation (complete at a later visit for active subjects if screening visit has already occurred)
- As part of the consenting process, an explanation of the PPMI Brain and Tissue Bank substudy will be discussed and subjects will be provided with an information packet. At some sites, subjects will be asked permission to have their contact information sent to the PPMI Brain and Tissue Bank (complete at next visit for active subjects if screening visit has already occurred). At other sites, following an explanation of the PPMI Brain and Tissue Bank substudy, subjects will be provided with an information packet and Intent to Donate form. Subjects wishing to sign the Intent to Donate form may do so at the time of screening. If subject is undecided, site should reapproach subject at next annual visit (or at site's discretion).
- Clinical laboratory assessments
- Vital signs (blood pressure, heart rate and temperature)
- Review of the subject's medical and family history
- Review of concomitant medications
- Review of current medical conditions
- General physical examination
- General neurological examination
- Movement Disorder Society Unified Parkinson Disease Rating Scale (MDS-UPDRS) Parts I- III
- Hoehn and Yahr
- Modified Schwab & England
- Montreal Cognitive Assessment (MoCA)
- A review of the inclusion/exclusion criteria to confirm that the subject is eligible to continue to the Baseline visit

5.3.4. Baseline Visit (Day 0):

The activities at the baseline visit will be completed within 45 days of completing the Screening visit. The Baseline visit will include the following activities and will take about 6 hours to complete. All assessments and activities listed below must be completed prior to enrollment of the subject into the study.

- Review of continuing ability to consent/Advance Directive
- Review willingness to share contact information with FOUND or, if enrolled in FOUND, review individual status report
- Height and weight
- Vital signs (blood pressure, heart rate and temperature)
- Blood draws for research samples (including blood draw for PBMCs)

- Urine collection for research samples
- Olfactory testing using the University of Pennsylvania Smell Identification Test (UPSIT)
- Clinical diagnosis assessment (SWEDD and Prodromal subjects only)
- Review of family history of PD (Genetic Cohort subjects only)
- Provide Family History substudy packet to subject (may be completed at a later visit for subjects enrolled prior to Amendment 8 approval); Record PPMI subject ID on Family Packet distribution sheet and fax to Indiana University (Genetic Cohort subjects only)
- PASE Exercise Questionnaire (Prodromal and Genetic Cohort subjects only)
- Epworth Sleepiness Scale
- REM Sleep Behavior Disorder Questionnaire
- Geriatric Depression Scale (GDS-15) (not applicable for Prodromal Subjects)
- State-Trait Anxiety Inventory (not applicable for Prodromal Subjects)
- Questionnaire for Impulsive-Compulsive Disorders
- Scales for Outcomes in Parkinson’s Disease (SCOPA-AUT)
- Letter Number Sequencing
- Hopkins Verbal Learning Test – Revised (Form 1)
- Symbol Digit Modalities Test (Form 1)
- Benton Judgment of Line Orientation (Odd items)
- Semantic Fluency
- Cognitive Categorization Assessment
- MDS-UPDRS Parts I-III (Part IV also conducted at each visit for subjects who have started PD medication)
- Hoehn and Yahr
- Modified Schwab & England (PD, Prodromal, SWEDD, and Genetic Cohort subjects only)
- MDS-UPDRS Part III and Hoehn & Yahr will be repeated one hour following dosing in clinic for Genetic Cohort subjects (PD and Unaffected) who have started levodopa or dopamine agonist
- Brain magnetic resonance imaging (MRI), (MRI will include DTI and resting state sequences at selected sites only)
- Lumbar puncture for collection of cerebral spinal fluid (CSF)
- Gait assessments, at selected sites (Genetic Cohort subjects only) (see section 6.3.9 for details)
- Review of concomitant medications
- Review of current medical conditions
- Review of adverse events related to lumbar puncture
- Repeat review of the inclusion/exclusion criteria
- Consent/withdrawal of consent for contact regarding future studies related or unrelated to PPMI

Once all Baseline activities have been completed and the Investigator determines that all eligibility criteria have been met, the subject may be enrolled into the study.

5.3.5. Visit 01 (3 months ±30 days)

- Review of continuing ability to consent/Advance Directive
- Review willingness to share contact information with FOUND or, if enrolled in FOUND, review individual status report
- Vital signs (blood pressure, heart rate and temperature)
- Blood draw for research samples
- MDS-UPDRS Parts I-III (PD, Prodromal and SWEDD subjects only) (Part IV also conducted at each visit for subjects who have started PD medication)
- Hoehn and Yahr scale (PD, Prodromal and SWEDD subjects only)
- Modified Schwab & England (PD, Prodromal and SWEDD subjects only)
- Clinical diagnosis assessment (Prodromal and SWEDD subjects only)
- Review of current medical conditions
- Review of concomitant medications
- Consent/withdrawal of consent for contact regarding future studies related or unrelated to PPMI

Note Genetic Cohort will not undergo Visit 01

5.3.6. Visit 02 (6 months ±30 days)

- Review of continuing ability to consent/Advance Directive
- Review willingness to share contact information with FOUND or, if enrolled in FOUND, review individual status report
- PPMI Brain and Tissue Bank (if subject has not yet been approached regarding Brain and Tissue Bank, review subject interest in sharing contact information with PPMI Brain and Tissue Bank study team or discuss Brain and Tissue Bank and present Intent to Donate form to subject)
- Vital signs (blood pressure, heart rate and temperature)
- Blood draws for research samples (including blood draw for PBMCs)
- Urine collection for research samples
- Lumbar puncture for collection of CSF
- *Epworth Sleepiness Scale
- *REM Sleep Behavior Disorder Questionnaire
- *GDS-15
- *State-Trait Anxiety Inventory
- *Questionnaire for Impulsive-Compulsive Disorders
- *SCOPA-AUT
- *MDS-UPDRS Parts I-III (Part IV also conducted at each visit for subjects who have started PD medication)
- *Hoehn and Yahr
- *Modified Schwab & England
- *Cognitive Categorization Assessment
- Clinical diagnosis assessment (Prodromal, SWEDD and Genetic Cohort subjects only)
- Review of family history of PD (Genetic Cohort subjects only)

- Review of current medical conditions
- Review of concomitant medications
- Review of adverse events related to lumbar puncture
- Consent/withdrawal of consent for contact regarding future studies related or unrelated to PPMI

*Completed for PD, SWEDD, Prodromal and Genetic Cohort subjects only

Note Genetic Cohort will not undergo Lumbar Puncture at Visit 02

5.3.7. Visit 03 (9 months ±30 days)

- Review of continuing ability to consent/Advance Directive
- Review willingness to share contact information with FOUND or, if enrolled in FOUND, review individual status report
- PPMI Brain and Tissue Bank (if subject has not yet been approached regarding Brain and Tissue Bank, review subject interest in sharing contact information with PPMI Brain and Tissue Bank study team or discuss Brain and Tissue Bank and present Intent to Donate form to subject)
- Vital signs (blood pressure, heart rate and temperature)
- Blood draw for research samples
- MDS-UPDRS Parts I-III (PD, Prodromal and SWEDD subjects only) (Part IV also conducted at each visit for subjects who have started PD medication)
- Hoehn and Yahr scale (PD, Prodromal and SWEDD subjects only)
- Modified Schwab & England (PD, Prodromal and SWEDD subjects only)
- Clinical diagnosis assessment (Prodromal and SWEDD subjects only)
- Review of current medical conditions
- Review of concomitant medications
- Consent/withdrawal of consent for contact regarding future studies related or unrelated to PPMI

Note Genetic Cohort will not undergo Visit 03

5.3.8. Visit 04 (12 months ±30 days)

- Review of continuing ability to consent/Advance Directive
- Review willingness to share contact information with FOUND or, if enrolled in FOUND, review individual status report
- PPMI Brain and Tissue Bank (for subjects who did not yet sign a form providing permission to be contacted by the Brain and Tissue Bank study team or have not yet signed an Intent to Donate form, review subject interest in PPMI Brain and Tissue Bank and remind interested subjects to follow up with the Brain and Tissue Bank study team or have subject sign an Intent to Donate form)
- General neurological examination
- Height and weight
- Vital signs (blood pressure, heart rate and temperature)

- Blood draw for research samples
- Urine collection for research samples
- Clinical laboratory assessments
- PASE Exercise Questionnaire
- Epworth Sleepiness Scale
- REM Sleep Behavior Disorder Questionnaire
- GDS-15
- State-Trait Anxiety Inventory
- Questionnaire for Impulsive-Compulsive Disorders
- SCOPA-AUT
- Montreal Cognitive Assessment (MoCA)
- Letter Number Sequencing
- Hopkins Verbal Learning Test – Revised (Form 2)
- Symbol Digit Modalities Test (Form 2)
- Benton Judgment of Line Orientation (Even items)
- Semantic Fluency
- Cognitive Categorization Assessment
- MDS-UPDRS Parts I-III (Part IV also conducted at each visit for subjects who have started PD medication)
- Hoehn and Yahr
- Modified Schwab & England (PD, Prodromal, SWEDD, and Genetic Cohort subjects only)
- MDS-UPDRS Part III and Hoehn & Yahr will be repeated one hour following dosing in clinic for subjects who have started levodopa or dopamine agonist
- Review of clinical diagnosis
- Review of family history of PD (Genetic Cohort subjects only)
- Lumbar puncture for collection of CSF
- SPECT imaging (PD and Prodromal subjects only – see Section 6.3.1), includes urine (or serum if required by site) pregnancy test for women of childbearing potential prior to injection
- VMAT-2 PET imaging, at selected sites, PD and Prodromal subjects (see Section 6.3.2)
- MRI-DTI and resting state (PD, Prodromal and HC subjects at selected sites)
- [^{18}F] florbetaben PET imaging at selected sites, PD (idiopathic and genetic cohorts), Prodromal (including RBD, olfactory and genetic cohort) subjects; or may be done at either V06 (24 months), V08 (36 months), V10 (48 months) or V12 (month 60); (or at V13 (month 72), V14 (month 84) or V15 (month 96) for PD and HC cohorts); see companion protocol.
- Gait assessments, at selected sites (Genetic Cohort subjects only) (see section 6.3.9 for details)
- Review of current medical conditions
- Review of concomitant medications
- Review of adverse events in follow up to SPECT (and/or VMAT-2 PET) imaging and/or lumbar puncture

- Consent/withdrawal of consent for contact regarding future studies related or unrelated to PPMI

Note Genetic Cohort will not undergo SPECT imaging, VMAT-2 imaging or DTI at Visit 04

5.3.9. Visit 05 (18 months ±30 days)

- Review of continuing ability to consent/Advance Directive
- Review willingness to share contact information with FOUND or, if enrolled in FOUND, review individual status report
- PPMI Brain and Tissue Bank (if subject has not yet been approached regarding Brain and Tissue Bank, review subject interest in sharing contact information with PPMI Brain and Tissue Bank study team or discuss Brain and Tissue Bank and present Intent to Donate form to subject)
- Vital signs (blood pressure, heart rate and temperature)
- Blood draws for research samples (including blood draw for PBMCs)
- MDS-UPDRS Parts I-III (PD, Prodromal SWEDD, and Genetic Cohort subjects only) (Part IV also conducted for subjects who have started PD medication)
- Hoehn and Yahr scale (PD, Prodromal, SWEDD and Genetic Cohort subjects only)
- Modified Schwab & England (PD, Prodromal, SWEDD and Genetic Cohort subjects only)
- Clinical diagnosis assessment (Prodromal, SWEDD and Genetic Cohort subjects only)
- Review of family history of PD (Genetic Cohort subjects only)
- Review of current medical conditions
- Review of concomitant medications
- Consent/withdrawal of consent for contact regarding future studies related or unrelated to PPMI

5.3.10. Visit 06 (24 months ±30 days)

- Review of continuing ability to consent/Advance Directive
- Review willingness to share contact information with FOUND or, if enrolled in FOUND, review individual status report
- PPMI Brain and Tissue Bank (for subjects who did not yet sign a form providing permission to be contacted by the Brain and Tissue Bank study team or have not yet signed an Intent to Donate form, review subject interest in PPMI Brain and Tissue Bank and remind interested subjects to follow up with the Brain and Tissue Bank study team or have subject sign an Intent to Donate form)
- General neurological examination
- Height and weight
- Vital signs (blood pressure, heart rate and temperature)
- Clinical laboratory assessments

- Blood draw for research samples
- Urine collection for research samples
- PASE Exercise Questionnaire
- Epworth Sleepiness Scale
- REM Sleep Behavior Disorder Questionnaire
- GDS-15
- State-Trait Anxiety Inventory
- Questionnaire for Impulsive-Compulsive Disorders
- SCOPA-AUT
- Montreal Cognitive Assessment (MoCA)
- Letter Number Sequencing
- Hopkins Verbal Learning Test – Revised (Form 3)
- Symbol Digit Modalities Test (Form 1)
- Benton Judgment of Line Orientation (Odd items)
- Semantic Fluency
- Cognitive Categorization Assessment
- MDS-UPDRS Parts I-III (Part IV also conducted at each visit for subjects who have started PD medication)
- Hoehn and Yahr
- Modified Schwab & England (PD, Prodromal, SWEDD and Genetic Cohort subjects only)
- MDS-UPDRS Part III and Hoehn & Yahr will be repeated one hour following dosing in clinic for subjects who have started levodopa or dopamine agonist
- Review of clinical diagnosis
- Review of family history of PD (Genetic Cohort subjects only)
- Lumbar puncture for collection of CSF
- SPECT imaging (PD, Prodromal, SWEDD, and Genetic Cohort subjects only – see Section 6.3.1), includes urine (or serum if required by site) pregnancy test for women of childbearing potential prior to injection
- VMAT-2 PET imaging, at selected sites, PD, SWEDD, Prodromal and Genetic Cohort subjects only (see Section 6.3.2)
- [^{18}F] florbetaben PET imaging at selected sites, PD (idiopathic and genetic cohorts), HC, and Prodromal (including RBD, olfactory and genetic cohort) subjects if not done at V04 (12 months); or may be done at V08 (36 months), V10 (48 months) or V12 (60 months); (or at V13 (month 72), V14 (month 84) or V15 (month 96) for PD and HC cohorts); see companion protocol.
- MRI DTI and resting state (PD, Prodromal, SWEDD and Genetic Cohort subjects at selected sites; HC at selected sites if not obtained at a previous visit
- Gait assessments, at selected sites (Genetic Cohort subjects only) (see section 6.3.9 for details)
- Review of current medical conditions
- Review of concomitant medications
- Review of adverse events in follow up to SPECT (and/or VMAT-2 PET) imaging and/or lumbar puncture

- Consent/withdrawal of consent for contact regarding future studies related or unrelated to PPMI
- Conclusion of study participation (SWEDD subjects only, unless continuing participation). Note SWEDD subjects may be asked to continue follow up through up to V15/Month 96 at the investigator's discretion, if by clinical assessment (e.g., subject had a positive scan at or before Month 24), the investigator determines there is substantial evidence that the subject may have PD.

5.3.11. Visit 07 (30 months +30 days)

- Review of continuing ability to consent/Advance Directive
- Review willingness to share contact information with FOUND or, if enrolled in FOUND, review individual status report
- PPMI Brain and Tissue Bank (if subject has not yet been approached regarding Brain and Tissue Bank, review subject interest in sharing contact information with PPMI Brain and Tissue Bank study team or discuss Brain and Tissue Bank and present Intent to Donate form to subject)
- Reconsent (For SWEDDS returning to the study)
- Vital signs (blood pressure, heart rate and temperature)
- Blood draws for research samples (including blood draw for PBMCs)
- MDS-UPDRS Parts I-III (PD, Prodromal and Genetic Cohort subjects only) (Part IV also conducted at each visit for subjects who have started PD medication)
- Hoehn and Yahr scale (PD, Prodromal and Genetic Cohort subjects only)
- Modified Schwab & England (PD, Prodromal and Genetic Cohort subjects only)
- Clinical diagnosis assessment (Prodromal and Genetic Cohort subjects only)
- Review of family history of PD (Genetic Cohort subjects only)
- Review of current medical conditions
- Review of concomitant medications
- Consent/withdrawal of consent for contact regarding future studies related or unrelated to PPMI

5.3.12. Visit 08 (36 months +30 days)

- Review of continuing ability to consent/Advance Directive
- Review willingness to share contact information with FOUND or, if enrolled in FOUND, review individual status report
- PPMI Brain and Tissue Bank (for subjects who did not yet sign a form providing permission to be contacted by the Brain and Tissue Bank study team or have not yet signed an Intent to Donate form, review subject interest in PPMI Brain and Tissue Bank and remind interested subjects to follow up with the Brain and Tissue Bank study team or have subject sign an Intent to Donate form)
- General neurological examination

- Height and weight
- Vital signs (blood pressure, heart rate and temperature)
- Clinical laboratory assessments
- Blood draw for research samples
- Urine collection for research samples
- PASE Exercise Questionnaire
- Epworth Sleepiness Scale
- REM Sleep Behavior Disorder Questionnaire
- GDS-15
- State-Trait Anxiety Inventory
- Questionnaire for Impulsive-Compulsive Disorders
- SCOPA-AUT
- Montreal Cognitive Assessment (MoCA)
- Letter Number Sequencing
- Hopkins Verbal Learning Test – Revised (Form 4)
- Symbol Digit Modalities Test (Form 2)
- Benton Judgment of Line Orientation (Even items)
- Semantic Fluency
- Cognitive Categorization Assessment
- UPSIT (Prodromal and Unaffected Genetic Cohort subjects only; if subject has already completed their month 36 visit at time of amendment 12 approval, this may be completed at a later visit)
- MDS-UPDRS Parts I-III (Part IV also conducted at each visit for subjects who have started PD medication)
- Hoehn and Yahr
- Modified Schwab & England (PD, Prodromal, and Genetic Cohort subjects only)
- MDS-UPDRS Part III and Hoehn & Yahr will be repeated one hour following dosing in clinic for subjects who have started levodopa or dopamine agonist
- SPECT imaging (Prodromal subjects only), includes urine (or serum if required by site) pregnancy test for women of childbearing potential prior to injection
- [^{18}F] florbetaben PET imaging at selected sites, PD (idiopathic and genetic cohorts), HC, and Prodromal (including RBD, olfactory and genetic cohort) subjects if not done at V04 or V06; or may be done at V10 (48 months) or V12 (60 months); (or at V13 (month 72), V4 (month 84) or V15 (month 96) for PD and HC cohorts); see companion protocol.
- MRI-DTI and resting state, HC at selected sites if not obtained at a previous visit
- Gait assessments, at selected sites (Genetic Cohort subjects only) (see section 6.3.9 for details)
- Review of clinical diagnosis
- Review of family history of PD (Genetic Cohort subjects only)
- Lumbar puncture for collection of CSF
- Review of current medical conditions
- Review of concomitant medications

- Review of adverse events relatedto SPECT imaging and/or lumbar puncture
- Consent/withdrawal of consent for contact regarding future studies related or unrelated to PPMI

5.3.13. Visit 09 (42 months ±30 days)

- Review of continuing ability to consent/Advance Directive
- Review willingness to share contact information with FOUND or, if enrolled in FOUND, review individual status report
- PPMI Brain and Tissue Bank (if subject has not yet been approached regarding Brain and Tissue Bank, review subject interest in sharing contact information with PPMI Brain and Tissue Bank study team or discuss Brain and Tissue Bank and present Intent to Donate form to subject)
- Vital signs (blood pressure, heart rate and temperature)
- Blood draws for research samples (including blood draw for PBMCs)
- MDS-UPDRS Parts I-III (PD, Prodromal, and Genetic Cohort subjects only) (Part IV also conducted at each visit for subjects who have started PD medication)
- Hoehn and Yahr scale (PD, Prodromal, and Genetic Cohort subjects only)
- Modified Schwab & England (PD, Prodromal, and Genetic Cohort subjects only)
- Clinical diagnosis assessment (Prodromal, and Genetic Cohort subjects only)
- Review of family history of PD (Genetic Cohort subjects only)
- Review of current medical conditions
- Review of concomitant medications
- Consent/withdrawal of consent for contact regarding future studies related or unrelated to PPMI

5.3.14. Visit 10 (48 months ±30 days)

- Review of continuing ability to consent/Advance Directive
- Review willingness to share contact information with FOUND or, if enrolled in FOUND, review individual status report
- PPMI Brain and Tissue Bank (for subjects who did not yet sign a form providing permission to be contacted by the Brain and Tissue Bank study team or have not yet signed an Intent to Donate form, review subject interest in PPMI Brain and Tissue Bank and remind interested subjects to follow up with the Brain and Tissue Bank study team or have subject sign an Intent to Donate form)
- General neurological examination
- Height and weight
- Vital signs (blood pressure, heart rate and temperature)
- Clinical laboratory assessments
- Blood draw for research samples
- Urine collection for research samples
- PASE Exercise Questionnaire

- Epworth Sleepiness Scale
- REM Sleep Behavior Disorder Questionnaire
- GDS-15
- State-Trait Anxiety Inventory
- Questionnaire for Impulsive-Compulsive Disorders
- SCOPA-AUT
- Montreal Cognitive Assessment (MoCA)
- Letter Number Sequencing
- Hopkins Verbal Learning Test – Revised (Form 5)
- Symbol Digit Modalities Test (Form 1)
- Benton Judgment of Line Orientation (Odd items)
- Semantic Fluency
- Cognitive Categorization Assessment
- MDS-UPDRS Parts I-III (Part IV also conducted at each visit for subjects who have started PD medication)
- Hoehn and Yahr
- Modified Schwab & England (PD, Prodromal, and Genetic Cohort subjects only)
- MDS-UPDRS Part III and Hoehn & Yahr will be repeated one hour following dosing in clinic for subjects who have started levodopa or dopamine agonist
- Review of clinical diagnosis
- Review of family history of PD (Genetic Cohort subjects only)
- Lumbar puncture for collection of CSF
- SPECT imaging (PD, prodromal and Genetic Cohort subjects only – see Section 6.3.1), includes urine (or serum if required by site) pregnancy test for women of childbearing potential prior to injection
- VMAT-2 PET imaging, at selected sites, PD, and Genetic Cohort subjects only (see Section 6.3.2)
- MRI DTI and resting state (PD, and Genetic Cohort subjects at selected sites; HC at selected sites if resting state sequences were not obtained at a previous visit)
- [^{18}F] florbetaben PET imaging at selected sites, PD (idiopathic and genetic cohorts), HC, and Prodromal (including RBD, olfactory and genetic cohort) subjects if not done at V04, V06 or V08; or may be done at V12 (60 months); (or at V13 (month 72), V14 (month 84) or V15 (month 96) for PD and HC cohorts); see companion protocol.
- Gait assessments, at selected sites (Genetic Cohort subjects only) (see section 6.3.9 for details)
- Review of current medical conditions
- Review of concomitant medications
- Review of adverse events in follow up to SPECT imaging (and/or VMAT-2 PET) and/or lumbar puncture
- Consent/withdrawal of consent for contact regarding future studies related or unrelated to PPMI

5.3.15. Visit 11 (54 months +30 days)

- Review of continuing ability to consent/Advance Directive
- Review willingness to share contact information with FOUND or, if enrolled in FOUND, review individual status report
- PPMI Brain and Tissue Bank (if subject has not yet been approached regarding Brain and Tissue Bank, review subject interest in sharing contact information with PPMI Brain and Tissue Bank study team or discuss Brain and Tissue Bank and present Intent to Donate form to subject)
- Vital signs (blood pressure, heart rate and temperature)
- Blood draws for research samples (including blood draw for PBMCs)
- MDS-UPDRS Parts I-III (PD and Genetic Cohort subjects only) (Part IV also conducted at each visit for subjects who have started PD medication)
- Hoehn and Yahr scale (PD and Genetic Cohort subjects only)
- Modified Schwab & England (PD and Genetic Cohort subjects only)
- Clinical diagnosis assessment (Genetic Cohort subjects only)
- Review of family history of PD (Genetic Cohort subjects only)
- Review of current medical conditions
- Review of concomitant medications
- Consent/withdrawal of consent for contact regarding future studies related or unrelated to PPMI

5.3.16. Visit 12 (60 months +30 days)

- Review of continuing ability to consent/Advance Directive
- Review willingness to share contact information with FOUND or, if enrolled in FOUND, review individual status report
- PPMI Brain and Tissue Bank (for subjects who did not yet sign a form providing permission to be contacted by the Brain and Tissue Bank study team or have not yet signed an Intent to Donate form, review subject interest in PPMI Brain and Tissue Bank and remind interested subjects to follow up with the Brain and Tissue Bank study team or have subject sign an Intent to Donate form)
- General neurological examination
- Height and weight
- Vital signs (blood pressure, heart rate and temperature)
- Clinical laboratory assessments
- Blood draw for research samples
- Urine collection for research samples
- PASE Exercise Questionnaire
- Epworth Sleepiness Scale
- REM Sleep Behavior Disorder Questionnaire
- GDS-15
- State-Trait Anxiety Inventory
- Questionnaire for Impulsive-Compulsive Disorders
- SCOPA-AUT

- Montreal Cognitive Assessment (MoCA)
- Letter Number Sequencing
- Hopkins Verbal Learning Test – Revised (Form 6)
- Symbol Digit Modalities Test (Form 2)
- Benton Judgment of Line Orientation (Even items)
- Semantic Fluency
- Cognitive Categorization Assessment
- MDS-UPDRS Parts I-III (Part IV also conducted at each visit for subjects who have started PD medication)
- Hoehn and Yahr
- Modified Schwab & England (PD, prodromal, PD and Unaffected Genetic Cohort subjects only)
- MDS-UPDRS Part III and Hoehn & Yahr will be repeated one hour following dosing in clinic for subjects who have started levodopa or dopamine agonist
- Review of clinical diagnosis
- Review of family history of PD (Genetic Cohort subjects only)
- Lumbar puncture for collection of CSF
- MRI DTI and resting state (PD and Genetic Cohort subjects at selected sites; HC at selected sites if resting state sequences were not obtained at a previous visit).
- [^{18}F] florbetaben PET imaging at selected sites, PD (idiopathic and genetic cohorts), HC and Prodromal (genetic cohort) subjects if not done at V04 or V06 or V10; (or may be done at V13 (month 72), V14 (month 84) or V15 (month 96) for PD and HC cohorts); see companion protocol.
- Gait assessments, at selected sites (Genetic Cohort subjects only) (see section 6.3.9 for details)
- Review of current medical conditions
- Review of concomitant medications
- Review of adverse events related to lumbar puncture
- Consent/withdrawal of consent for contact regarding future studies related or unrelated to PPMI

After January 1 2019, all subjects completing visits post month 60/visit 12 will follow a modified schedule of activities. This applies to all cohorts. Please see the section below titled "Modified Schedule of Activities" for details on the assessments and procedures to be completed at post V12 visits after January 1, 2019.

5.3.17. Visit 13 (72 months ±30 days)- (Parkinson Disease and Healthy Control Subjects only prior to January 1, 2019)

- Review of continuing ability to consent/Advance Directive
- Review willingness to share contact information with FOUND or, if enrolled in FOUND, review individual status report
- PPMI Brain and Tissue Bank (for subjects who did not yet sign a form providing permission to be contacted by the Brain and Tissue Bank study team or have not yet signed an Intent to Donate form, review subject interest in PPMI Brain and Tissue Bank and remind interested subjects to follow up with the

Brain and Tissue Bank study team or have subject sign an Intent to Donate form)

- General neurological examination
- Height and weight
- Vital signs (blood pressure, heart rate and temperature)
- Clinical laboratory assessments
- Blood draw for research samples
- Urine collection for research samples
- PASE Exercise Questionnaire
- Epworth Sleepiness Scale
- REM Sleep Behavior Disorder Questionnaire
- GDS-15
- State-Trait Anxiety Inventory
- Questionnaire for Impulsive-Compulsive Disorders
- SCOPA-AUT
- Montreal Cognitive Assessment (MoCA)
- Letter Number Sequencing
- Hopkins Verbal Learning Test – Revised (Form 1)
- Symbol Digit Modalities Test (Form 1)
- Benton Judgment of Line Orientation (odd items)
- Semantic Fluency
- Cognitive Categorization Assessment
- MDS-UPDRS Parts I-III (Part IV also conducted at each visit for subjects who have started PD medication)
- Hoehn and Yahr
- Modified Schwab & England (PD only)
- MDS-UPDRS Part III and Hoehn & Yahr will be repeated one hour following dosing in clinic for subjects who have started levodopa or dopamine agonist
- Review of clinical diagnosis
- Lumbar puncture for collection of CSF (optional)
- [^{18}F] florbetaben PET imaging at selected sites, PD (idiopathic cohort) subjects if not done at V04, V06, V10 or V12; (or may be done at V14 (month 84) or V15 (month 96) for PD and HC cohorts); see companion protocol.
- Review of current medical conditions
- Review of concomitant medications
- Review of adverse events related to lumbar puncture (if lumbar puncture performed)
- Consent/withdrawal of consent for contact regarding future studies related or unrelated to PPMI

5.3.18. Visit 14 (84 months ±30 days) (Parkinson Disease and Healthy Control Subjects only prior to January 1, 2019)

- Review of continuing ability to consent/Advance Directive
- Review willingness to share contact information with FOUND or, if enrolled in FOUND, review individual status report
- PPMI Brain and Tissue Bank (for subjects who did not yet sign a form providing permission to be contacted by the Brain and Tissue Bank study team or have not yet signed an Intent to Donate form, review subject interest in PPMI Brain and Tissue Bank and remind interested subjects to follow up with the Brain and Tissue Bank study team or have subject sign an Intent to Donate form)
- General neurological examination
- Height and weight
- Vital signs (blood pressure, heart rate and temperature)
- Clinical laboratory assessments
- Blood draw for research samples
- Urine collection for research samples
- PASE Exercise Questionnaire
- Epworth Sleepiness Scale
- REM Sleep Behavior Disorder Questionnaire
- GDS-15
- State-Trait Anxiety Inventory
- Questionnaire for Impulsive-Compulsive Disorders
- SCOPA-AUT
- Montreal Cognitive Assessment (MoCA)
- Letter Number Sequencing
- Hopkins Verbal Learning Test – Revised (Form 2)
- Symbol Digit Modalities Test (Form 2)
- Benton Judgment of Line Orientation (even items)
- Semantic Fluency
- Cognitive Categorization Assessment
- MDS-UPDRS Parts I-III (Part IV also conducted at each visit for subjects who have started PD medication)
- Hoehn and Yahr
- Modified Schwab & England (PD only)
- MDS-UPDRS Part III and Hoehn & Yahr will be repeated one hour following dosing in clinic for subjects who have started levodopa or dopamine agonist
- Review of clinical diagnosis
- Lumbar puncture for collection of CSF (optional)
- [^{18}F] florbetaben PET imaging at selected sites, PD (idiopathic cohort) or HC subjects if not done at V04, V06, V10, V12 or V13; (or may be done V15 (month 96) for PD cohort); see companion protocol.
- Review of current medical conditions
- Review of concomitant medications

- Review of adverse events related to lumbar puncture (if lumbar puncture performed)
- Consent/withdrawal of consent for contact regarding future studies related or unrelated to PPMI

5.3.19 Visit 15 (96 months ±30 days) (Parkinson Disease and Healthy Control Subjects prior to January 1, 2019)

- Review of continuing ability to consent/Advance Directive
- Review willingness to share contact information with FOUND or, if enrolled in FOUND, review individual status report
- PPMI Brain and Tissue Bank (for subjects who did not yet sign a form providing permission to be contacted by the Brain and Tissue Bank study team or have not yet signed an Intent to Donate form, review subject interest in PPMI Brain and Tissue Bank and remind interested subjects to follow up with the Brain and Tissue Bank study team or have subject sign an Intent to Donate form) General neurological examination
- Height and weight
- Vital signs (blood pressure, heart rate and temperature)
- Clinical laboratory assessments
- Blood draw for research samples
- Urine collection for research samples
- PASE Exercise Questionnaire
- Epworth Sleepiness Scale
- REM Sleep Behavior Disorder Questionnaire
- GDS-15
- State-Trait Anxiety Inventory
- Questionnaire for Impulsive-Compulsive Disorders
- SCOPA-AUT
- Montreal Cognitive Assessment (MoCA)
- Letter Number Sequencing
- Hopkins Verbal Learning Test – Revised (Form 3)
- Symbol Digit Modalities Test (Form 1)
- Benton Judgment of Line Orientation (odd items)
- Semantic Fluency
- Cognitive Categorization Assessment
- MDS-UPDRS Parts I-III (Part IV also conducted at each visit for subjects who have started PD medication)
- Hoehn and Yahr
- Modified Schwab & England (PD only)
- MDS-UPDRS Part III and Hoehn & Yahr will be repeated one hour following dosing in clinic for subjects who have started levodopa or dopamine agonist
- Review of clinical diagnosis
- Lumbar puncture for collection of CSF (optional)

- [^{18}F] florbetaben PET imaging at selected sites, PD (idiopathic cohort) subjects if not done at V04, V06, V10, V12, V13 or V14; see companion protocol.
- Review of current medical conditions
- Review of concomitant medications
- Review of adverse events related to lumbar puncture (if lumbar puncture performed)
- Consent/withdrawal of consent for contact regarding future studies related or unrelated to PPMI

Modified Schedule of Activities

The schedule of activities below should be followed for all post month 60 visits after January 1, 2019 for all cohorts. For the PD and Healthy Control cohorts, this may include month 72, 84, 96, 108, 120, 132, 144 and 156 visits; for the Prodromal and Genetic cohorts, this may include month 72, 84, 96, 108 and 120 visits.

5.3.20 Modified annual in clinic visit

- Review of continuing ability to consent/Advance Directive
- Consent/withdrawal of consent for contact regarding future studies related or unrelated to PPMI
- Review willingness to share contact information with FOUND or, if enrolled in FOUND, review individual status report
- PPMI Brain and Tissue Bank (for subjects who did not yet sign a form providing permission to be contacted by the Brain and Tissue Bank study team or have not yet signed an Intent to Donate form, review subject interest in PPMI Brain and Tissue Bank and remind interested subjects to follow up with the Brain and Tissue Bank study team or have subject sign an Intent to Donate form)
- Participant Questionnaire (Prodromal and Unaffected Genetic cohort subjects only; questionnaire to be completed prior to subject interacting with investigator)
- General neurological examination (Prodromal and Unaffected Genetic Cohort subjects only)
- Vital signs
- Blood draw for research samples
- Blood draw for PBMCs
- PASE Exercise Questionnaire
- Epworth Sleepiness Scale
- REM Sleep Behavior Disorder Questionnaire
- GDS-15
- State-Trait Anxiety Inventory for Adults
- Questionnaire for Impulsive-Compulsive Disorders
- SCOPA-AUT
- Montreal Cognitive Assessment (MoCA)
- Letter Number Sequencing
- Hopkins Verbal Learning Test – Revised (use next subsequent form; see PPMI Operations manual for schedule of forms)

- Symbol Digit Modalities Test (alternate form 1 and 2; see PPMI Operations manual for schedule of forms)
- Benton Judgment of Line Orientation (alternate odd and even items; see PPMI Operations manual for schedule)
- Semantic Fluency (animals only)
- Trails A and B
- Lexical Fluency (FAS)
- Boston Naming Test
- Cognitive Categorization Assessment
- Determination of Falls
- NeuroQOL
- UPSIT (at every annual visit for PD, PD Genetic Cohort and Healthy Control subjects; at V12, V14, V16, V18 and V20 for Prodromal and Unaffected Genetic Cohort subjects)
- MDS-UPDRS Parts I-III (Part IV also conducted at each visit for subjects who have started PD medication)
- Hoehn and Yahr
- Modified Schwab & England (except Healthy Control subjects)
- MDS-UPDRS Part III and Hoehn & Yahr will be repeated one hour following dosing in clinic for subjects who have started levodopa or dopamine agonist
- Review of clinical diagnosis
- Lumbar puncture for collection of CSF (V12, V14, V16, V18 and V20 only)
- DatSCAN (at one time point only and only for selected subjects in prodromal and genetic cohorts)
- Review of current medical conditions
- Review of concomitant medications
- Review of adverse events related to lumbar puncture (if lumbar puncture performed)

5.3.21 Telephone Contacts

Telephone call visits will be conducted 7 to 10 days following a visit when lumbar puncture and/or dopamine transporter SPECT imaging has occurred to assess for adverse events. In addition, after Month 12, telephone call visits will take place 3 months through month 60 following each in-person visit to discuss any questions, verify whether or not PD medications have been started and to confirm the date of the next scheduled visit.

After month 60 during the Modified Schedule of Activities, telephone call visits will take place 6 months after each annual in person visit. During those visits the following activities will occur:

- Consent to share contact information for FOUND
- Consent to share contact information for PPMI Brain and Tissue Bank
- Confirmation of vital status
- Confirm/update subject contact information
- Clinical diagnosis review

- Participant Questionnaire (Prodromal and Unaffected Genetic cohort subjects only)

Refer to ^{18}F-AV-133-PPMI companion protocol for telephone follow up requirements for subjects participating in VMAT-2 PET imaging and refer to [18F] florbetaben companion protocol for telephone follow up requirements related to that study.

5.3.22 Unscheduled Visits (Visit U01, U02, etc.), if required

Unscheduled visits may be performed at any time during the study whenever necessary to assess for or to follow up on adverse events or as deemed necessary by the Site Investigator or Coordinator. Subjects undergoing imaging multiple assessments (e.g., subjects participating in companion imaging protocols) that require a delay between scans may complete their imaging at an unscheduled visit. Subjects consenting to skin biopsies (at participating sites) may have their biopsy completed at an unscheduled visit. Subjects consenting to an additional blood draw (at participating sites) may have their blood draw completed at an unscheduled visit. The following activities will be completed at an Unscheduled Visit:

- Review of continuing ability to consent/Advance Directive
- Review willingness to share contact information with FOUND or, if enrolled in FOUND, review individual status report
- PPMI Brain and Tissue Bank (if subject has not yet been approached regarding Brain and Tissue Bank, review subject interest in sharing contact information with PPMI Brain and Tissue Bank study team or discuss Brain and Tissue Bank and present Intent to Donate form to subject)
- Vital signs
- *General neurological examination
- *Collect blood for clinical laboratory assessments
- Review of current medical conditions
- Review of concomitant medications

*Conducted only if clinically indicated

5.3.23 Premature Withdrawal of Participation

If the subject agrees, the following procedures should be performed when a subject withdraws early from the study:

- Review of continuing ability to consent/Advance Directive
- Review willingness to share contact information with FOUND or, if enrolled in FOUND, review individual status report
- PPMI Brain and Tissue Bank (for subjects who did not yet sign a form providing permission to be contacted by the Brain and Tissue Bank study team or have not yet signed an Intent to Donate form, review subject interest in PPMI Brain and Tissue Bank and remind interested subjects to follow up with the Brain and Tissue Bank study team or have subject sign an Intent to Donate form)
- General neurological examination

- Height and weight
- Vital signs (blood pressure, heart rate and temperature)
- Clinical diagnosis assessment
- Review of family history of PD (Genetic Cohort only)
- Blood draw for research samples – only if not done in the last 3 months
- Urine collection for research samples – only if not done in the last 3 months
- Clinical laboratory assessments
- PASE Exercise Questionnaire
- Epworth Sleepiness Scale
- REM Sleep Behavior Disorder Questionnaire
- GDS-15
- State-Trait Anxiety Inventory
- Questionnaire for Impulsive-Compulsive Disorders
- SCOPA-AUT
- Montreal Cognitive Assessment (MoCA)
- Letter Number Sequencing
- Hopkins Verbal Learning Test – Revised (version not used at preceding visit)
- Symbol Digit Modalities Test (version not used at preceding visit)
- Benton Judgment of Line Orientation (items not used at preceding visit)
- Semantic Fluency
- Cognitive Categorization Assessment
- MDS-UPDRS Parts I-III (Part IV will also be conducted at each visit for subjects who have started PD medication)
- Hoehn and Yahr
- Modified Schwab & England (PD Prodromal, Genetic Cohort subjects only)
- MDS-UPDRS Part III and Hoehn & Yahr will be conducted again one hour following dosing in clinic for subjects who have started levodopa or dopamine agonist
- Lumbar puncture for collection of CSF – only if not done in the last 3 months
- SPECT imaging – only if not done in the last 12 months (PD, Prodromal SWEDD, and Genetic Cohort subjects only – see Section 6.3.1,) includes urine (or serum if required by site) pregnancy test for women of childbearing potential prior to injection
- VMAT-2 PET imaging – only if not done in the last 12 months for PD SWEDD, Prodromal and Genetic Cohort subjects only (refer to ^{18}F-AV-133-PPMI companion protocol)
- MRI DTI and resting state (**PD**, Prodromal, SWEDD and Genetic Cohort subjects at selected sites if MRI not done in the last 12 months)
- MRI DTI and resting state (**HC** subjects at selected sites if PW visit conducted within first 12 months and MRI not done in the last 6 months)
- [^{18}F] florbetaben PET imaging if subject has not completed this imaging at earlier visit and is willing to complete at PW visit.
- Review of current medical conditions
- Review of concomitant medications

- Review of adverse events in follow up to SPECT imaging and/or lumbar puncture
- Consent/withdrawal of consent for contact regarding future studies related or unrelated to PPMI
- Lexical Fluency (FAS) [During modified schedule of activities only]
- Boston Naming Test [During modified schedule of activities only]
- Determination of falls [During modified schedule of activities only]
- NeuroQOL [During modified schedule of activities only]

5.3. 24 PD, SWEDD, or Prodromal Subjects Starting PD Medication:

If the site becomes aware that a subject will begin PD medication in advance of or at the time of a scheduled visit, the site should determine if the subject is willing to conduct a Symptomatic Therapy (ST) visit prior to starting medications. If a subject is not willing to return for an ST visit prior to starting PD medication, conduct the next study visit per the regular visit schedule. If the subject agrees, an ST visit should be conducted and the appropriate visit schedule below should be followed (see Section 5.3.26 for list of ST visit assessments):

- Before Visit 01: Complete ST visit assessments. Visit 01 will be missed and the subject will return to the regular visit schedule for Visit 02.
- At Visit 01 or prior to Visit 02: Complete ST visit assessments. Visit 02 will be missed and the subject will return to the regular visit schedule for Visit 03.
- At Visit 02 or prior to Visit 03: Complete ST visit assessments. Visit 03 will be missed and the subject will return to the regular visit schedule for Visit 04.
- At Visit 03 or prior to Visit 04: Complete ST visit assessments. Visit 04 will be missed and the subject will return to the regular visit schedule for Visit 05.
- At Visit 04: Complete ST visit assessments. The subject will return to the regular visit schedule for Visit 05 assessments.
- After Visit 04: If an ST visit is conducted within 3 months prior to the next visit's Target Date, or as part of that next regularly scheduled study visit, the ST visit will replace that visit. The subject will then continue following the Visit Window Schedule. If the subject returns outside of the 3 month window prior to the next visit, conduct the ST visit. The subject will return again for the next regular study visit.

The start of PD medication should be reported to the CTCC and documented on the appropriate source worksheet.

5.3.25 Genetic Cohort Subjects Starting PD Medication:

If the site becomes aware that a subject will begin PD medication in advance of or at the time of a scheduled visit, the site should determine if the subject is willing to conduct a Symptomatic Therapy (ST) visit prior to starting medications. If a subject is not willing to

return for an ST visit prior to starting PD medication, conduct the next study visit per the regular visit schedule. If the subject agrees, an ST visit should be conducted and the appropriate visit schedule below should be followed (see Section 5.3.26 for list of ST visit assessments):

- At or prior to Visit 02: Complete ST visit assessments. Visit 02 will be missed and the subject will return to the regular visit schedule for Visit 04.
- After Visit 2 but Prior to Visit 04: Complete ST visit assessments. Visit 04 will be missed and the subject will return to the regular visit schedule for Visit 05.
- At Visit 04: Complete ST visit assessments. The subject will return to the regular visit schedule for Visit 05 assessments.
- After Visit 04: If an ST visit is conducted within 3 months prior to the next visit's Target Date, or as part of that next regularly scheduled study visit, the ST visit will replace that visit. The subject will then continue following the Visit Window Schedule. If the subject returns outside of the 3 month window prior to the next visit, conduct the ST visit. The subject will return again for the next regular study visit.

The initiation of PD medication should be reported to the CTCC and documented on the appropriate source worksheet.

5.3.26. Symptomatic Therapy Visit (PD, SWEDD, Prodromal and PD Genetic Cohort subjects only)*

- Review of continuing ability to consent/Advance Directive
- Review willingness to share contact information with FOUND or, if enrolled in FOUND, review individual status report
- PPMI Brain and Tissue Bank (for subjects who did not yet sign a form providing permission to be contacted by the Brain and Tissue Bank study team or have not yet signed an Intent to Donate form, review subject interest in PPMI Brain and Tissue Bank and remind interested subjects to follow up with the Brain and Tissue Bank study team or have subject sign an Intent to Donate form) Vital signs (blood pressure, heart rate and temperature)
- Blood draw for research samples
- Urine collection for research samples
- Epworth Sleepiness Scale
- REM Sleep Behavior Disorder Questionnaire
- GDS-15
- State-Trait Anxiety Inventory
- Questionnaire for Impulsive-Compulsive Disorders
- SCOPA-AUT
- MDS-UPDRS Parts I-III
- Hoehn and Yahr
- Modified Schwab and England
- Clinical diagnosis assessment

- Review of family history of PD (PD Genetic Cohort subjects only)
- Lumbar puncture for collection of CSF (not repeated if CSF was collected less than 3 months prior to the ST visit)
- SPECT Imaging
  - ❖ Conduct for PD and Prodromal subjects when ST visit replaces a Month 12, Month 24, or Month 48 visit (as applicable)
  - ❖ Conduct for SWEDD subject when ST visit replaces Month 24 visit
  - ❖ Conduct for PD Genetic Cohort subjects when ST visit replaces a Month 12, Month 24, Month 36, or Month 48 visit (as applicable)
- VMAT-2 PET Imaging (selected sites only; refer to ^{18}F-AV-133-PPMI companion protocol)
  - ❖ Conduct for PD subject when ST visit replaces Month 12, Month 24, or Month 48 visit
  - ❖ Conduct for SWEDD subject when ST visit replaces Month 24 visit
  - ❖ Conduct for PD Genetic Cohort subjects when ST visit replaces a Month 12, Month 24, Month 36, or Month 48 visit (as applicable)
- Review of current medical conditions
- Review of concomitant medications
- Review of adverse events day of visit and in follow up to lumbar puncture and/or SPECT (or VMAT-2) imaging (as applicable)
- Consent/withdrawal of consent for contact regarding future studies related or unrelated to PPMI

Additional assessments to be completed when ST visit is conducted in place of an annual visit:

- General neurological examination
- Clinical laboratory assessments
- MoCA
- Letter Number Sequencing
- Hopkins Verbal Learning Test – Revised (refer to section 6 of Operations Manual to determine appropriate form to administer)
- Symbol Digit Modalities Test
- Benton Judgment of Line Orientation
- Semantic Fluency
- Cognitive Categorization

* Symptomatic therapy visits will not be completed as part of the modified schedule of activities.

5.3.27. Phenoconversion Visit (Prodromal and Unaffected Genetic Cohort subjects only)
If a Prodromal or Unaffected Genetic cohort subject is diagnosed with PD or other neurodegenerative disorder in the course of clinical care, then a phenoconversion visit should be completed. The clinical diagnosis of PD or a neurodegenerative disorder can be made by the site investigator if the investigator is also providing clinical care to the participant or by a non-study physician with appropriate training (e.g. non-study neurologist, primary care provider). The

schedule of activities for a phenoconversion visit is below. The phenoconversion visit should occur within 45 days of the site being aware of clinical phenoconversion.

Following the phenoconversion visit, please resume the schedule of activities, starting with the subject's next regularly scheduled visit. However, if the phenoconversion visit occurs within the 2 months prior to an expected annual visit, then the phenoconversion visit will supersede the annual visit and the annual visit will not be conducted.

All assessments and activities listed below should be completed as part of a phenoconversion visit:

- Review of clinical diagnosis*
- Review of continuing ability to consent/Advance Directive
- Consent/withdrawal of consent for contact regarding future studies related or unrelated to PPMI
- Review willingness to share contact information with FOUND or, if enrolled in FOUND, review individual status report
- PPMI Brain and Tissue Bank (for subjects who did not yet sign a form providing permission to be contacted by the Brain and Tissue Bank study team or have not yet signed an Intent to Donate form, review subject interest in PPMI Brain and Tissue Bank and remind interested subjects to follow up with the Brain and Tissue Bank study team or have subject sign an Intent to Donate form)
- General neurological examination
- Vital signs
- Blood draw for research samples
- Blood draw for PBMCs
- PASE Exercise Questionnaire
- Epworth Sleepiness Scale
- REM Sleep Behavior Disorder Questionnaire
- GDS-15
- State-Trait Anxiety Inventory for Adults
- Questionnaire for Impulsive-Compulsive Disorders
- SCOPA-AUT
- Montreal Cognitive Assessment (MoCA)
- Letter Number Sequencing
- Hopkins Verbal Learning Test – Revised (use next subsequent form; see PPMI Operations manual for schedule of forms)
- Symbol Digit Modalities Test (alternate form 1 and 2; see PPMI Operations manual for schedule of forms)
- Benton Judgment of Line Orientation (alternate odd and even items; see PPMI Operations manual for schedule)
- Semantic Fluency (animals only)
- Cognitive Categorization Assessment
- Trails A and B
- Lexical Fluency (FAS)
- Boston Naming Test

- Determination of Falls
- NeuroQOL
- UPSIT
- MDS-UPDRS Parts I-III (Part IV also conducted at each visit for subjects who have started PD medication)
- Hoehn and Yahr
- Modified Schwab & England
- MDS-UPDRS Part III and Hoehn & Yahr will be repeated one hour following dosing in clinic for subjects who have started levodopa or dopamine agonist
- Participant Questionnaire
- DAT imaging
- Lumbar puncture for collection of CSF
- Review of current medical conditions
- Review of concomitant medications
- Review of adverse events related to lumbar puncture or DAT imaging (if lumbar puncture or DAT imaging performed)

5.3.28 Study visits for Genetic Registry

Once sites receive amendment 13 approval, all visits will cease for Genetic Registry subjects. Genetic Registry subjects who are enrolled in FOUND in PPMI will have the opportunity to continue to be followed through FOUND in PPMI. Genetic Registry subjects will complete one final phone visit following site's approval of amendment 13. (At the site's discretion, the final Genetic Registry visit may be done in person if deemed appropriate.)

This final phone visit will include the following activities:

- Verbal consent
- Review of clinical diagnosis
- Review of current medical conditions
- Review willingness to share contact information with FOUND or, if enrolled in FOUND, review individual status report.
- Review subject status regarding enrollment in PPMI Brain and Tissue Bank

6. Study Assessments

6.1. Clinical Assessments

6.1.1. MDS-UPDRS

The MDS-UPDRS is a multimodal scale assessing both impairment and disability and is separated into 4 subscales (Parts I-IV). The MDS-UPDRS includes components assessed by the study investigator as well as sections completed by the subject. Every effort should be made to have the same investigator perform the ratings for an individual subject throughout the course of the study.

- Part I: This assesses non-motor experiences of daily living and is comprised of two components:
    - ❖ Part IA contains 6 questions that are assessed by the Investigator and focuses on complex behaviors.
    - ❖ Part IB contains 7 questions that are part of the Patient Questionnaire completed by the subject.
- Part II: This assesses motor experiences of daily living. There are an additional 13 questions that are also part of the Patient Questionnaire completed by the subject.
- Part III: This assesses the motor signs of PD and is administered by the Investigator.
- Part IV: This assesses motor complications, dyskinesias and motor fluctuations using historical and objective information. The Investigator will complete this assessment at each visit once a subject has started PD medication.

Subjects (except Registry-PD subjects) who have started PD medication (levodopa or dopamine agonist) will have an annual assessment of the motor exam (Part III) and Hoehn and Yahr in a practically defined off state and then these assessments will be repeated one hour after receiving medication in clinic. These subjects will need to be reminded not to take PD medication on the day of each annual study visit. If possible, subjects on PD medication who withdraw prematurely from the study should also have the Part III assessment one hour after receiving medication during the Premature Withdrawal Visit.

6.1.2. Hoehn and Yahr Stage

The Hoehn and Yahr is a commonly used system for describing how the symptoms of Parkinson disease progress. The scale allocates stages from 0 to 5 to indicate the relative level of disability. This scale is included within the MDS-UPDRS and will be completed for all subjects.

- Stage zero: No symptoms.
- Stage one: Symptoms on one side of the body only.
- Stage two: Symptoms on both sides of the body. No impairment of balance.
- Stage three: Balance impairment. Mild to moderate disease. Physically independent.
- Stage four: Severe disability, but still able to walk or stand unassisted.
- Stage five: Wheelchair-bound or bedridden unless assisted.

6.1.3. Modified Schwab & England Activities of Daily Living

The Modified Schwab & England Activities of Daily Living (ADL) scale reflects the speed, ease, and independence with which an individual performs daily activities, or personal chores, such as eating, toileting, and dressing. This scale uses a rating scale from 0% to 100%, with 100% representing complete independence in performing daily activities and 0% representing a vegetative, bedridden state.

6.1.4. The University of Pennsylvania Smell Test

The University of Pennsylvania Smell Identification TEST (UPSIT) is a 40-item, multiple choice, scratch and sniff test used to evaluate odor identification. It is a forced-choice test in which subjects must identify an odor among four response alternatives. There are four booklets containing ten odorants each. The instructions will be explained to the subjects by the coordinator at the clinical site. Subjects may complete the UPSIT independently. It will be reviewed for completion prior to the end of the visit. The UPSIT will be scored by the coordinator reflecting the number of correct responses out of 40 items.

6.1.5. Clinical Diagnosis and Management Questionnaire

The Clinical Diagnosis and Management Questionnaire is being used in the SWEDD cohort of subjects to allow site Investigators to provide a more detailed assessment regarding the subject's clinical signs and symptoms of disease and to document any changes in diagnosis or management of the clinical diagnosis.

6.1.6. Diagnostic Questionnaire

The Diagnostic Questionnaire is being used for the Prodromal and Genetic Cohort and Registry subjects to allow Investigators to provide a detailed assessment regarding any clinical signs and symptoms and to document any changes in diagnosis. This assessment and the Diagnostic Features assessment will be used as research based criteria for determining phenoconversion to motor PD as assessed by the Investigator.

6.1.7 Determination of Falls

Questions assess the incidence of falls and freezing of gait over the previous week and 12 months. The questionnaire also documents whether any injuries resulted from reported falls and potential resource utilization.

Neuropsychological and Cognitive Assessments

6.1.8. Montreal Cognitive Assessment

The Montreal Cognitive Assessment (MoCA): In early Parkinson disease, when cognitive deficits occur, they are subtle and mild and the patients usually perform in the normal range on the widely used Mini Mental State Examination. The Montreal Cognitive Assessment (MoCA) is a rapid screening instrument like the MMSE but was developed to be more sensitive to patients presenting with mild cognitive complaints. It assesses short term and working memory, visuospatial abilities, executive function, attention, concentration, language and orientation. The total score ranges from 0 to 30.

6.1.9. Epworth Sleepiness Scale (ESS)[64],

The Epworth Sleepiness Scale (ESS)[64], used extensively in PD related studies, is a self-administered questionnaire collecting information on the propensity to fall asleep in eight different situations encountered commonly in daily life. Each situation is rated from 0 (no chance of dozing) to 3 (high chance of dozing), and the total score ranges from 0 to 24. Total scores of zero to 10 are normal, scores from 10 to 12 are borderline, and scores from 12 to 24 are abnormal.

6.1.10. Geriatric Depression Scale (GDS-15)

The Geriatric Depression Scale (GDS-15) is a self-report scale shown to be a useful measure of depressive symptoms in patients with Parkinson disease[65]. It is particularly easy for patients to use given its "yes/no" format. The GDS-15 is a validated shortened version of the original scale.

6.1.11. WMS-III Letter-Number Sequencing Test[66]

The WMS-III Letter-Number Sequencing Test[66] is a measure of verbal working memory. In this test, subjects are read a combination of random letters and numbers and are asked to repeat the string back to the experimenter organized so that numbers are first in ascending order and letters next in alphabetical order. The length of the string is increased at each trial. The total score is the number of trials correctly repeated.

6.1.12. Hopkins Verbal Learning Test-Revised (HVLT-R)

The Hopkins Verbal Learning Test-Revised (HVLT-R) is a test of verbal, short-term memory/new learning requiring rapid encoding of information[67]. Subjects must learn a list of 12 words which are grouped into three semantically-related categories each consisting of four words (e.g., dwellings, precious gems, animals). Subjects are given three repeated learning trials followed by a 20-25 minute delayed recall and recognition phase.

6.1.13. Benton Judgment of Line Orientation Test

The Benton Judgment of Line Orientation T**est** is a measure of spatial perception and orientation. It is recognized that disturbances of these functions result from brain disease. The booklet consists of 5 practice items in addition to the test stimuli, consisting of line segments appearing at various angled intervals in the top half of the booklet and multiple-choice response cards in the lower half. The test will be conducted such that 15 of the 30 items are completed at each administration.

6.1.14. Symbol Digit Modalities Test (SDMT)

The Symbol Digit Modalities Test (SDMT) screens cognitive impairment by using a simple substitution task that adults with normal functioning can easily perform. Using a reference key, the examinee has 90 seconds to match specific numbers with geometric figures. Responses may be oral or written, allowing the test to be used with a wide variety of people, including those with motor disabilities or speech disorders. The SDMT is relatively culture free since it uses only geometric figures and numbers. Norms for adults are separated by age group and educational level.

6.1.15. The Trail Making Test (TMT)

The TMT provides information on visual search, scanning, speed of processing, mental flexibility, and executive functions. The TMT consists of two parts. TMT-part A requires an individual to draw lines sequentially connecting 25 encircled numbers distributed on a sheet of paper. Task requirements are similar for TMT-part B except the person must alternate between numbers and letters (e.g., 1, A, 2, B, 3, C, etc.). The score on each part represents the amount of time required to complete the tasks.

6.1.16. Boston Naming Test (15- item)

The Boston Naming Test assesses subjects' visual confrontation naming abilities and can assist in detecting mild word retrieval deficits.

6.1.17. Lexical and Semantic fluency

The FAS lexical fluency test is a subtest of the Neurosensory Center Comprehensive Examination for Aphasia[74]. It assesses phonemic fluency by requesting an individual to orally produce as many words as possible that begin with the letters F, A, and S within a prescribed time frame. Semantic fluency will be measured in a similar manner using the category of animals. Subjects will be asked to name as many animals as they can in a one minute trial.

6.1.18. State-Trait Anxiety Inventory (STAI-Y)

The State-Trait Anxiety Inventory (S**TAI-Y)** is a self rated assessment to measure emotional state anxiety in adults[68]. The 40-item state-trait anxiety questions will be administered. Responses resulting in a higher score indicate greater anxiety.

6.1.19. REM Sleep Behavior Disorder Screening Questionnaire (RBDSQ)

The REM Sleep Behavior Disorder Screening Questionnaire (RBDSQ) is a 10-item self rated questionnaire to assess sleep-wake disturbances. Patients with clinical characterizations of sleep behavior disorder may represent early manifestations of progressive neurodegenerative disorders, including Parkinson disease[69], thus making this an important tool for longitudinal prospective studies.

6.1.20. Questionnaire for Impulsive-Compulsive Disorders (QUIP-S)

The abbreviated version of the Questionnaire for Impulsive-Compulsive Disorders **(QUIP-S)** is a 13-item self administered assessment. This questionnaire will measure impulse control disorders and other compulsive behaviors in subjects with Parkinson disease[70] as compared to a SWEDD and healthy control population.

6.1.21. Scales for Outcomes in Parkinson's Disease

The Scales for Outcomes in Parkinson's Disease assessment of autonomic dysfunction **(SCOPA-AUT)]**[71] is a 26-item self administered test developed to evaluate autonomic symptoms, such as gastrointestinal and urinary problems, in subjects with PD. The assessment will measure whether subject's experience an increase in autonomic dysfunction as the disease severity progresses.

6.1.22. Determination of Dementia/Mild Cognitive Impairment

The **Cognitive Categorization** assessment will be completed to make a determination of Parkinson Disease Dementia (PDD) and PD with mild cognitive impairment (PD-MCI). The assessment will be made on the basis of criteria developed by the Movement Disorders Society[63,72,73]. Information provided by the subject or other informant, the Investigator's judgment, and results from the cognitive testing covering four cognitive domains will be used for this assessment.

The determination of PDD will be made on the following factors:

1. History of cognitive decline determined by the investigator based on information from the patient, other informant (spouse, family member or friend) and the investigator's judgment.
2. Cognitive impairment defined as at least 1 test score (out of 6 scores) from at least 2 domains (out of 4 domain) >1.5 SD below the standardized mean.
3. Functional limitation as a result of cognitive impairment.

The determination of PD-MCI will be made based on the following factors:

1. Cognitive complaint by either the patient or informant (spouse, family member or friend).
2. Cognitive impairment defined as at least 2 test scores (out of 6 scores) from at least 1 domain (out of 4) >1.0 SD below the standardized mean.
3. No functional impairment as a result of cognitive impairment.

Cognitive categorization will be performed annually. For subjects who have had visits prior to the implementation of this assessment, the site investigator may be asked to make the determination retrospectively based on his/her best clinical judgment. Retrospective determinations will be noted in the PPMI database.

6.1.23. Physical Activity Scale for the Elderly (PASE) Assessment

The PASE assessment is a subject completed questionnaire to evaluate physical activity levels in the PD subjects as compared to the Controls, SWEDD, Prodromal, and Genetic Cohort subjects. The assessment will be used to measure the impact of exercise frequency and intensity on the rate of progression of PD disability.

6.1.21 Quality of Life in Neurological Disorders (Neuro-QOL)

Neuro-QoL is a set of self-report measures that assesses the health-related quality of life of adults with neurological disorders. The measure is comprised of scales that evaluate symptoms, concerns, and issues that are relevant to people with neurological disorders, including PD. The self-report measures can be completed by a proxy responder when necessary. Scales assessing the domains of lower extremity function/mobility, upper extremity function/fine motor, cognitive function and communication will be administered.

6.1.25 Participant Questionnaire

The Participant Questionnaire assesses whether subjects have experienced specified symptoms (e.g., poor balance, difficulty rising from chair, shuffling gait, etc.) over the prior week. Subjects are also asked whether they have received a diagnosis of PD or another neurological condition since their last study visit.

6.2. Safety Assessments

6.2.1. Medical History and Physical/Neurological Examination

Medical and family history, as well as a complete physical and neurological exam will be captured on all subjects at Screening. A neurological exam will also be conducted annually and at the last completed visit. Healthy control subjects will have a complete neurological

examination conducted at screening and every 12 months to ensure no changes have occurred since entry into the study. Genetic Cohort subjects will have a complete neurological examination at their annual visits. (A neurological examination will not be performed as part of the modified schedule of activities for PD, healthy control and PD Genetic cohort subject visits post month 60 or post January 1, 2019.)

6.2.2 Vital Signs/Weight/Height

Pulse rate (supine and standing), blood pressure (supine and standing), and oral temperature will be determined at every visit. The supine blood pressure and pulse rate will be determined after 1-3 minutes of quiet rest and the standing pressure and rate will be determined after 1-3 minutes in the standing position. Weight and height will be collected at the Baseline visit and annually or bi-annually according to the visit schedule.

6.2.3. Clinical Laboratory Tests

Routine clinical laboratory tests indicated in the table below will be performed at screening and follow up visits according to the visit schedule. (Clinical laboratory tests will not be performed as part of the modified schedule of activities.) A central laboratory will be implemented in order to guarantee identical analysis methods, consistent normal ranges and thus common interpretation of laboratory changes. If not stated otherwise, venous whole blood will be collected in blood collection tubes (vacutainers). All samples for laboratory analysis must be collected, prepared, labelled, and shipped according to the laboratory's requirement as detailed in the lab manual. The total amount of blood needed for the clinical lab tests will be no more than 10 ml.

The coagulation panel (PT/PTT) will be collected and shipped by all sites to the central lab for analysis at the Screening visit only. Sites have the option, per clinical practice, to collect an additional blood sample to evaluate coagulation results prior to the conduct of additional lumbar puncture assessments. Sites will be provided with supplies from the central lab; however, the sample should be sent to a local lab facility for analysis (if a site does not have a local lab facility, the coagulation panel will be shipped to the central lab). Results will be evaluated to determine, in the opinion of the Investigator, whether there are any issues that may preclude conduct of the follow up lumbar puncture. Results should be maintained as part of the subject's study documents; however, will not be included in the study database.

No more than 45 ml will be drawn at any visit, including both clinical and research blood samples.

CENTRAL LAB TESTS	
**METABOLIC PANEL**	**COMPLETE BLOOD COUNT**
Sodium (Na)<br>Potassium (K)<br>Chloride (Cl)<br>Carbon Dioxide (CO2)<br>Blood Urea Nitrogen (BUN)<br>Glucose<br>Calcium (Ca)<br>Creatinine (Crn)<br>Bilirubin Total<br>Albumin	White Blood Cell Count (WBC)<br>Red Blood Cell Count (RBC)<br>Hemoglobin (Hb)<br>Hematocrit (HCT)<br>Platelet Count (PLT)

Total Protein<br>Aspartate aminotransferase (AST)<br>Alanine aminotransferase (ALT)<br>Alkaline Phosphatase (ALKP)<br>Uric Acid<br>Prothrombin time (PT) – Screening Only<br>Partial Thromboplastin Time (PTT) – Screening Only	

6.3. Other Assessments

6.3.1. Dopamine Transporter Scan and SPECT Imaging

Subjects will have dopamine transporter imaging procedure to measure the amount of dopamine in the brain using single photon emission computed tomography (SPECT). All subjects (except Genetic Registry subjects) will undergo SPECT imaging scan at Screening. Parkinson disease, SWEDD, Prodromal and Genetic Cohort (PD and Unaffected) subjects will also undergo follow up SPECT scans as indicated in the visit schedule.

The SPECT imaging procedure will be performed at the individual sites using DaTSCAN™ as the dopamine transporter. Should there be any interruption in the availability of DaTSCAN™ during the conduct of the protocol subjects will be asked to travel to the Institute for Neurodegenerative Disorders (IND) in New Haven, CT to complete the SPECT scan. Should DaTSCAN™ be unavailable at IND, subjects will be asked to undergo [^{123}I]β-CIT injection and SPECT scan. [^{123}I]β-CIT is another dopamine transporter ligand that produces an outcome comparable to that of DaTSCAN™. Any subject who travels to IND for the SPECT imaging scan will be given a separate informed consent for signature prior to completion of any SPECT imaging scan activities. Travel to New Haven, CT will be provided for the subject and a companion through study funds.

Upon completion of the initial (Screening) SPECT scan, the imaging core will complete a Visual Interpretation Report.

- If the Visual Interpretation read for a PD subject indicates that the scan does not show evidence of dopamine transporter deficit, the subject may be enrolled as a SWEDD subject (or continue follow up visits as a SWEDD subject if the SPECT scan is completed after the Baseline visit).
- If the Visual Interpretation read for a Control subject indicates that the scan shows evidence of dopamine transporter deficit, the subject may be enrolled (or will remain in the study if the SPECT scan is completed following the baseline visit).
- For subjects undergoing a SPECT scan as a screening procedure for the Prodromal cohort, a quantitative analysis will be completed to determine whether the subject is eligible for enrollment into the Prodromal cohort (see Section 4.1 and 4.2.7.2).

Since this imaging information and the products used to complete the dopamine transporter SPECT scans are investigational as used in the PPMI study, it cannot provide definite information about a clinical diagnosis.

Subjects will be monitored by study personnel for adverse events on the day that a dopamine transporter SPECT scan is obtained. Subjects will also be contacted by phone 7 to 10 days following the injection/scan to assess adverse events. These events will be reported by the site investigator as required to the site's Institutional Review/Ethics Boards and to his/her Radiation Safety Committee.

The procedures that would take place for a DaTSCAN™ or [^{123}I]β-CIT injection are described below.

6.3.1.1. DaTSCAN™ Imaging Procedure

Women of childbearing potential must have a urine (or serum if required by the site) pregnancy test prior to injection of DaTSCAN™. The result must be confirmed as negative prior to proceeding with the injection. Before the DaTSCAN™ injection, subjects will be pre-treated with stable iodine (10 drops of a saturated solution of potassium iodide) to reduce the uptake of DaTSCAN™ by the thyroid. Subjects will be injected with 3-5 mCi of dopamine transporter. Within a 4 hour (+/- 30 minute) window following the injection, subjects will undergo SPECT imaging on the camera. The data and quality assurance procedures to be employed in this study are described in the operations manual.

6.3.1.2. [^{123}I]β-CIT Imaging Procedure

Subjects will be injected with up to 6 mCi of [^{123}I]β-CIT the day before the SPECT scan. Clinical laboratory tests (chem 20 and CBC) may be completed at IND if screening labs are not available or were completed more than 60 days prior to the injection of [^{123}I]β-CIT. An ECG will be acquired at IND prior to injection. In addition, vital signs (blood pressure, pulse) will be completed prior to the injection and approximately 15 minutes post injection. The labs, ECG and vital signs are completed to check the general health of the subject before completing the imaging procedures. Women of childbearing potential will have a urine pregnancy test, as well as a serum pregnancy test prior to injection. The result of the urine pregnancy test must be confirmed as negative prior to proceeding with the injection. Before the [^{123}I]β-CIT injection, subjects will be pre-treated with stable iodine (10 drops of a saturated solution of potassium iodide) to reduce the uptake of [^{123}I]β-CIT by the thyroid. Subjects will return to IND about 20 hours post injection for the SPECT scan. Vital signs will be taken prior to the scan. Markers filled with ^{57}CO will be attached to both sides of the subject's head at the level of the canthomeatal line before imaging to facilitate post hoc computer reorientation of transaxial images. Projection data will be acquired for about 30 minutes.

6.3.2. VMAT-2 PET Imaging [Refer to the ^{18}F-AV-133-PPMI companion protocol]

Subjects participating in Australia and selected U.S. sites will undergo [^{18}F] AV-133 PET imaging to assess targeting the vesicular monoamine transporter. In Australia, [^{18}F] AV-133 PET imaging will be used as an inclusion criterion for PD subjects and is required of all subjects. In the U.S., [^{18}F] AV-133 PET imaging will be conducted in addition to DAT imaging and will be optional. Subjects in the U.S. may consent to participate in the companion study beginning with the Year 01 (month 12) scan. All

subjects in Australia and those participating in the selected U.S. sites will undergo an initial [^{18}F] AV-133 PET imaging scan. Parkinson disease SWEDD, prodromal and genetic cohort subjects will also undergo follow up [^{18}F] AV-133 PET scans as indicated in the visit schedule. For subjects completing both DAT and [^{18}F] AV-133 imaging, PET scans will be performed 1 to 8 weeks after DAT SPECT imaging (i.e., the initial Screening PET scan may be completed after the subject completes PPMI Baseline visit/enrollment). The imaging information and the products used to complete the VMAT-2 PET scans are investigational. [^{18}F] AV-133 data will not be provided to the study subjects other than to provide information regarding study eligibility.

In Australia, upon completion of the initial (Screening) [^{18}F] AV-133 PET scan, the Imaging core will complete a Visual Interpretation Report. If the Visual Interpretation read for a PD subject indicates that the scan does not show evidence of VMAT deficit, the subject will be excluded from the study as a PD subject. Subjects screened as PD subjects, but without evidence of VMAT deficit may be enrolled as a SWEDD subject.

In the U.S., for subjects receiving both DAT SPECT imaging and [^{18}F] AV-133 PET, if the Visual Interpretation read of the DAT SPECT for a patient screened as a PD subject indicates that the scan does not show evidence of dopamine transporter deficit (regardless of the [^{18}F] AV-133 PET imaging) the subject may be enrolled as a SWEDD subject.

6.3.2.1. [^{18}F] AV-133 Imaging Procedure

Refer to the ^{18}F-AV-133-PPMI companion protocol and the PPMI Technical Operations Manual for AV-133 PET imaging procedures.

6.3.3. [^{18}F] florbetaben PET imaging

[Refer to the *Evaluation of [^{18}F] florbetaben in Subjects Participating in the Parkinson's Progression Markers Initiative* companion protocol]
Subjects participating at selected PPMI sites will undergo [^{18}F] florbetaben PET imaging at either their Month 12, 24, 36, 48, 60, 72, 84 or 96 visit to evaluate an investigational imaging agent [^{18}F] florbetaben, as a tracer for the visual detection of beta-amyloid in the brain.

6.3.4. Magnetic Resonance Imaging (MRI) and Diffusion Tensor Imaging (DTI)

Subjects (except Genetic Registry subjects) will undergo a structural MRI brain scan at the Baseline visit. At the discretion of the investigator and imaging staff, subjects who have presence of pacemakers, aneurysm clips, artificial heart valves, ear implants, metal fragments or foreign objects in the eyes, skin or body or any other known contra-indication to MRI may be advised not to complete a baseline (or follow-up) MRI scan, but these subjects may still participate in the study. MRI is being conducted to assess the structure of the brain. At selected sites with DTI 3T scanner capabilities, subjects will undergo MRI (DTI) at Baseline and follow up visits as indicated in the visit schedules. The diffusion tensor imaging will be conducted to further assess the

pathways that connect parts of the brain and the function of cells in those pathways. In addition, at selected sites, subjects undergoing MRI (DTI) may also have resting state functional magnetic resonance imaging (rsfMRI) sequences collected. The rsfMRI sequences will provide another mechanism to assess the structural networks in the brain as related to PD.

6.3.5. Biologic Sampling (Blood and Urine)

Whole blood (about 6 ml), serum (about 10 ml) and plasma (about 10 ml) will be collected to conduct proteomic, metabolomic and other analyses. Blood will be obtained (about 8 ml) for the extraction of DNA to conduct sequencing and genomic analyses. Blood will also be obtained (about 5 ml) for the extraction of RNA to conduct biochemical analyses and for the collection of peripheral blood mononuclear cells (PBMC) (about 10 ml). PBMCs are an important biospecimen resource for PD research focused on inflammation, LRRK2 detection, and lysosomal enzyme activity. Urine (about 10 ml) will be collected to conduct analyte analyses. (Urine samples will not be collected as part of the modified schedule of activities.)

It is strongly advised that the research blood samples are collected in a fasted state (i.e., minimum of 8 hours since last meal/food intake) to ensure the quality of samples for future analyses. If fasting is not possible, then subjects should be strongly advised to eat a low lipid diet as provided. All research samples will be sent to a central repository to be stored indefinitely for research purposes. Samples will be made available to researchers to conduct analyses related to PD and other disorders. Subjects will not receive any individual results of analysis or testing conducted on the biologic samples.

6.3.6. Lumbar Puncture

The lumbar puncture (LP) is performed by the site investigator or another qualified clinician appointed by the investigator. A lumbar puncture for the collection of 15-20 ml of CSF will be conducted for all subjects (except Genetic Registry subjects) per the visit schedule unless there is evidence of clinically significant coagulopathy or thrombocytopenia that would interfere with the safe conduct of the procedure. While it is expected that all subjects will undergo an LP at Baseline, SWEDD subjects may be permitted to enroll without completion of the baseline LP. The first 2 ml of CSF will be processed at the site's local lab facility (unless the lab is not able to process the CSF within 4 hours) to conduct standard analyses on cell count, protein and glucose levels. Subjects will be closely monitored during the procedure and following the procedure. Subjects will be contacted by phone 7 to 10 days following an LP to assess for any adverse events. The CSF samples will be sent to a central repository to be stored indefinitely for research purposes. The CSF samples will be made available to researchers to conduct analyses related to PD and other disorders.

6.3.7 Skin Biopsy/Blood samples

Subjects participating at selected sites involved in collecting biological samples for research purposes including the production of iPS cells will either undergo a skin biopsy or blood draw.

Subjects who consent to participation in the companion protocol titled *"Skin Biopsies for the Generation of Induced Pluripotent Stem Cells to Model Parkinson Disease from the Parkinson's Progression Markers Initiative (PPMI)"* will undergo a skin biopsy procedure at a routine or unscheduled PPMI study visit, with a post-biopsy follow-up telephone contact within 7-10 days. (Refer to the companion protocol for the schedule of activities and operations manual for procedures to be followed.)

Subjects who consent to collection of a blood sample will complete blood draws at an unscheduled visit, separate from a routine PPMI study visit. The unscheduled visit should occur approximately 1 month apart from a routine PPMI study visit for subjects completing a blood draw. (This blood draw will be in addition to the blood samples described above as part of routine PPMI study visits.) Alternately, sites may forego the collection of PBMCs and the biomic blood sample at a single interim visit in order to perform an iPSC blood sample collection at the interim visit instead.

6.3.8 Gait Assessments

Subjects participating at selected sites involved in the study of gait and mobility in PPMI (*Characterization of Motor Function and Potential Transition States to Parkinson's Disease in LRRK2 mutation Carriers and Controls)* will complete a short series of motor assessments using wearable devices. The system includes 3 lightweight wireless wearable sensors containing 3 axial accelerometers, gyroscopes and magnetometers (Opal , APDM Ltd.). The system measures acceleration of movement in 3 orthogonal axes as a function of time. The recording unit is small, lightweight and housed in a custom made Velcro-belt. The sensors are worn on both wrists and on the lower back of the participants during all gait measurements to quantify temporal measures such as stride time, gait variability, step symmetry, axial angular movement in three planes, and magnitude and symmetry of arm swing during each of the walks. Subjects in the PD and unaffected genetic cohort who are LRRK2 carriers will be invited to participate in the gait assessment study. [Refer to the *Characterization of Motor Function and Potential Transition States to Parkinson's Disease in LRRK2 mutation Carriers and Controls* companion protocol]

7. Concomitant Medications

7.1.1. Initiation of PD Medications

It is anticipated that the majority of PD (and SWEDD) subjects will be able to remain off PD medications for at least 6 months after Baseline. However, PD medications may be initiated at any time after enrollment at the discretion of the patient or treating physician (inclusive of Prodromal and Genetic Cohort PD subjects). The medication used is at the discretion of the treating physician. It is anticipated that the majority of Genetic Cohort-PD and Registry-PD subjects may be on PD medications at baseline. If PD medications are to be initiated, subjects (except the Genetic Registry) will be asked to return for an ST

visit. The investigator will document any new medications or changes in medication at each study visit on the Concomitant Medication Log.

7.1.2. Use of Concomitant Medications

Concomitant medications, including over-the-counter (OTC), dietary supplements (e.g., herbal remedies) or prescriptions, are permitted during the study period, except for the following medications that might interfere with dopamine transporter SPECT imaging which are restricted 6 months prior to a DaTSCAN^{TM} injection: Neuroleptics, metoclopramide, alpha methyldopa, methylphenidate, reserpine, or amphetamine derivative. For subjects participating in VMAT-2 PET imaging, refer to the ^{18}F-AV-133-PPMI companion protocol Section 5.4 for restricted medications. All concomitant medications reported at the time of the screening visit and for the duration of the subject's participation should be recorded on the Concomitant Medication Log.

7.1.3. Participation in Clinical Trials

It is preferred that PPMI subjects do not participate in clinical trials of investigational intervention during the entire PPMI study. However, for subjects who choose to participate in clinical trials of investigational interventions, it is preferred that this not occur until after 12 months of participation in PPMI. The investigator will document the study drug dosage, if applicable, and, if unknown, will report on the identity of the study drug and dosage after it is unmasked. If a PD, SWEDD, Prodromal or Genetic Cohort subject chooses to participate in another clinical trial at any time during participation in PPMI, an ST visit may need to be conducted before the subject begins investigational intervention. Please contact the CTCC for further instruction.

8. Subject Withdrawals

Subjects will be advised in the written informed consent forms that they have the right to withdraw from the study at any time without prejudice, and may be withdrawn at the investigator's or sponsor's discretion at any time. A subject should be withdrawn from the study if the investigator considers it to be medically necessary, or if the subject withdraws consent. All reasons for subject withdrawals from the study will be recorded in the source documentation and appropriate eCRF.

9. Subject Retention

Recruitment and retention of research participants is paramount to the success of PPMI. Approaches to retention and long-term follow up are described as follows.

9.1 Advance Directive for Clinical Research Participation

The potential for development of cognitive impairment in PPMI subjects is recognized. In accordance with good clinical practices in ensuring each subject's ability to give ongoing informed consent over the course of study participation, the Advance Directive for Clinical Research Participation ("advance directive") is one way to enable continued participation in subjects whose ability to consent becomes compromised. The advance directive enables subjects to clarify their preferences, thus guiding the substitute decision maker and the

investigator. It is noted that the accepted term and/or required directive and/or consent for a designated substitute decision maker (also known as a research proxy) may vary on a country/state/provincial basis.

During the consenting process, subjects will be encouraged to identify a substitute decision maker who they will permit to carry out the subject's wishes regarding continued participation (or not) in PPMI should the subject lose the ability to make his own decision. If the subject agrees to complete the advance directive, the site investigator will exercise clinical judgment and ascertain the subject's ability to continue giving informed consent. This ascertainment may include a review of the study purpose, differences between research and clinical assessments and the risks of study participation. If deemed necessary by the investigator, the subject will be approached about contacting the person(s) named in the advance directive at that visit, while the subject is still capable of discussing the need for a proxy. The substitute decision maker will be contacted by telephone during the study visit if not already present to discuss the next steps for determining the subject's continuing participation or not. Completion of the advance directive is voluntary; thus, identification of a substitute decision maker (research proxy) is not required in order for the subject to participate in PPMI; however, in the absence of an advance directive the Investigator deems the subject no longer able to provide consent, the subject will be withdrawn from the study.

Documentation of completion of the advance directive, routine review of the subject's continuing ability to give informed consent at each visit, and any discussion with the subject's substitute decision maker, will be noted on in the subject source and eCRF.

For subjects already enrolled prior to implementation of this amendment, the advance directive should be introduced and obtained at the next possible in-person visit, if the subject is willing.

9.2. Follow Up of Persons with Neurologic Disease (FOUND) in PPMI

The Follow Up of Persons with Neurologic Disease (FOUND) study (Caroline Tanner MD, Principal Investigator, University of California-San Francisco (UCSF)) provides a parallel, centralized system to prospectively collect vital status and disease progression information from persons with parkinsonism, related disorders and healthy controls who are participating in clinical research studies. Participation in this study complements in-person assessment and enables continuity of follow up of individuals who complete or withdraw from a study. A PPMI subject's participation in FOUND via the companion protocol, Follow Up of Persons with Neurologic Disease in PPMI, will enable centralized contact both during and after completion of PPMI, using convenient methods for systematic data collection (e.g., regular mail, telephone, internet contacts). The companion study may aid PPMI subject retention by 1) minimizing lost-to-follow-up status for unanticipated dropouts, 2) providing continued assessment of disease status for those unable to complete the study protocol, 3) provide systematically, prospectively collected information on subjects after study completion, and 4) streamline processes for maintaining communications with participants through use of a single IRB (centralized IRB).

As part of FOUND, subjects will also complete an assessment of exposure to environmental factors, the Parkinson's Disease Risk Factor Questionnaire (PD-RFQ). The Risk Factor Questionnaire is a validated questionnaire developed to provide a standard assessment tool for documenting environment exposure factors in studies of Parkinson's disease. It has been validated for self-report and interview. The PD-RFQ assesses exposures in the following domains: caffeine, tobacco, alcohol, head injury, residential and occupational histories, NSAID and hormonal medications, body habitus, pesticide exposure, other chemical exposures.

Each PPMI site will ask its subjects at initial consent to PPMI and at each visit if their contact information may be shared with Dr. Tanner and her team at UCSF. The subject's decision will be documented in the PPMI informed consent and CRF. The PPMI site will notify UCSF, who will proceed with contacting the subject to invite their participation. UCSF will share with referring PPMI sites their subjects' participation status in FOUND at regular intervals via a report. Sites will then review this with participants at subject visits. For subjects who have incomplete enrollment in FOUND, sites will identify if there are any issues impeding enrollment and address any such issues. The PPMI study data will be maintained at UCSF under an IRB approved protocol. The data collected from the FOUND study will be uploaded into the PPMI data repository at the Laboratory of Neuro Imaging (LONI) The Institute for Neuroimaging and Informatics in Los Angeles, California, at regular intervals.

10. PPMI Brain and Tissue Bank

The PPMI Brain and Tissue Bank (Tatiana Foroud, PhD, Principal Investigator, Indiana University) provides a depository for brain and other relevant tissues donated for research purposes. Post-mortem analysis of brain tissue is an important aspect of Parkinson's Disease research. The donated tissue will allow researchers to examine changes noted in the post-mortem brain tissue and correlate it with changes in neuropsychological, imaging, and biomic data collected in the PPMI study.

Each PPMI site will discuss the PPMI Brain and Tissue Bank with subjects and provide them with an information packet at initial consent to PPMI and at periodic intervals. At some PPMI sites, subjects will be asked to provide permission to allow the PPMI site to provide the subject's contact information to the Brain and Tissue Bank study team, who will then contact subjects to discuss tissue donation further and answer questions. Those subjects agreeing to donation will be asked to sign an Intent to Donate form approved by the PPMI Brain and Tissue Bank's IRB to document their desire to donate brain tissue and other relevant tissues upon their death.

At other PPMI sites, the local sites will provide subjects with an information packet explaining the opportunity and process for donating brain and other tissues and discuss donation directly with subjects. If a subject agrees to donation, the site will ask the subject to sign an Intent to Donate form approved by their local Ethics Committee or IRB to document their desire to donate brain tissue and other relevant tissues upon their death.

Subjects will also be provided with contact information for the Pathology Core study team and may contact the Pathology Core directly. The Pathology Core study team will discuss tissue donation further with such subjects and answer questions. Those subjects agreeing to donation will be asked to sign an Intent to Donate form.

The data collected from the PPMI Brain and Tissue Bank will be collated at the central core and uploaded into the PPMI data repository at the Laboratory for Neuro Imaging (LONI) The Institute for Neuroimaging and Informatics in Los Angeles, California, at regular intervals.

11. Safety/Adverse Events

Site investigators and coordinators will be instructed to assess for adverse events at in-person study visits when an LP, skin biopsy and/or dopamine transporter SPECT imaging activity is conducted, as well as by telephone 7 to 10 days following such activity.

In addition, for select sites, adverse events will be assessed at the in-person visit when [^{18}F] AV-133 or [^{18}F] florbetaben PET imaging is conducted, by telephone 48 ($\pm$ 24) hours following such activity.Each subject must be carefully monitored for adverse events (AEs). An assessment must be made of the seriousness, intensity, and relationship to the study procedure (LP or skin biopsy) and/or administration of the study drug (DaTSCAN™ , [^{18}F] AV-133, and/or [^{18}F] florbetaben as applicable).

Table 1. Adverse event monitoring timelines:

Procedure	Assessed through visit	48 hours (+/-24 hrs) telephone contact	7 to 10 day telephone contact
Lumbar Puncture	X		X
Dopamine Transporter SPECT Imaging	X		X
AV-133 PET Imaging	X	X	
[^{18}F] florbetaben Imaging	X		X
Skin biopsy	X		X

Events occurring outside of the study drug/ study procedure AE reporting period do not have to be recorded for study purposes (i.e., they do not have to be captured on the AE log).

10.1 Adverse Event Definitions

<u>Adverse Events (AE)</u>

An AE is any undesirable experience occurring to a subject during a clinical trial, whether or not considered related to the investigational product.

Pre-existing conditions (i.e., undesirable experiences, signs or symptoms that begin prior to the Screening Visit) will be recorded on the medical history CRF page. These conditions will not be reported as an AE unless they worsen in intensity or frequency after the Screening Visit (per the reporting period defined in section 9 above).

Treatment-emergent AEs are undesirable experiences, signs, or symptoms that begin or worsen in intensity or frequency at the time of or after the administration of study drug (per the reporting period defined in section 9 above).

For reporting purposes, Avid (manufacturer of [^{18}F] AV-133) will distinguish among pre-existing conditions and treatment-emergent AEs.

Serious Adverse Event (SAE)

An SAE is an AE that is fatal or life-threatening, or results in hospitalization, prolongation of hospitalization, persistent or significant disability/incapacity, or a congenital anomaly/birth defect. A life-threatening AE is an AE that, in the view of the investigator, places the subject at immediate risk of death from the reaction, as it occurred. Important medical events that may not result in death, be life-threatening, or require hospitalization may be considered an SAE when, based upon appropriate medical judgment, they may jeopardize the subject and may require medical or surgical intervention to prevent one of the outcomes listed in this definition.

Inpatient admission in the absence of a precipitating, treatment-emergent, clinical adverse event is not subject to immediate reporting. For example:

- Admission for treatment of a pre-existing condition not associated with the development of a new adverse event
- Social admission (e.g., subject has no place to sleep)
- Protocol-specific admission during a clinical study (e.g., for a procedure required by another study protocol)
- Optional admission not associated with a precipitating clinical adverse event (e.g., for elective cosmetic surgery)

Inpatient admission does not include the following:

- Emergency Room/Accident and Emergency/Casualty Department visits
- Outpatient/same-day/ambulatory procedures
- Observation/short-stay units
- Rehabilitation facilities
- Hospice facilities
- Respite care (e.g., caregiver relief)
- Skilled nursing facilities
- Nursing homes
- Custodial care facilities

Unexpected Adverse Event

For FDA reporting purposes, an unexpected AE is an AE not previously reported or an AE that occurs with specificity, severity or frequency that is not consistent with the current investigator's brochure.

10.2 Relationship to Investigational Product

The assessment of the relationship of an AE to the administration of the study drug is a clinical decision based on all available information at the time the event is being documented. The following definitions of the relationship between the study drug and the AE (including SAEs) should be considered:

- Unrelated - No possible relationship
  The temporal relationship between drug exposure and the adverse event onset/course is unreasonable or incompatible, or a causal relationship to study drug is implausible.

- Unlikely - Not reasonably related, although a causal relationship cannot be ruled out
  While the temporal relationship between drug exposure and the adverse event onset/course does not preclude causality, there is a clear alternate cause that is more likely to have caused the adverse event than the study drug.

- Possible - Causal relationship is uncertain
  The temporal relationship between drug exposure and the adverse event onset/course is reasonable or unknown, dechallenge or rechallenge information is either unknown or equivocal, and while other potential causes may not exist, a causal relationship to the study drug does not appear probable.

- Probable - High degree of certainty for causal relationship
  The temporal relationship between drug exposure and the adverse event onset/course is reasonable. There is a clinically compatible response to dechallenge (rechallenge is not required), and other causes have been eliminated or are unlikely.

- Definite - Causal relationship is certain
  The temporal relationship between drug exposure and the adverse event onset/course is reasonable, there is a clinically compatible response to dechallenge, other causes have been eliminated, and the event must be definitive pharmacologically or phenomenologically, using a satisfactory rechallenge procedure if necessary.

10.3 Intensity/Severity of an Adverse Event

In addition to assessing the relationship of the administration of the investigational product to AEs, an assessment is required of the intensity (severity) of the event. The following classifications should be used:

- *Mild:*
  A mild AE is an AE, usually transient in nature and generally not interfering with normal activities.

- *Moderate*:
  A moderate AE is an AE that is sufficiently discomforting to interfere with normal activities.

- *Severe:*
  A severe AE is an AE that incapacitates the subject and prevents normal activities. Note that a severe event is not necessarily a serious event. Nor must a serious event necessarily be severe.

10.4 Recording of Adverse Experiences

Adverse experiences, whether observed by the investigator, elicited from or volunteered by the subject, should be recorded on the Adverse Event Log. This will include a brief description of the experience, the date of onset, the date of resolution, the severity, and seriousness and whether the event was related to participation in the research study.

If the imaging scan is completed at the Institute for Neurodegenerative Disorders, staff at IND will contact the subject by telephone 7 to 10 days following injection to assess adverse events. A copy of all relevant study documentation generated by IND will be sent to the appropriate site Investigator.

Any adverse event ongoing at the final 7 to 10 day reporting telephone visit, or the final 48 hour reporting telephone visit (whichever occurs last), should be followed until resolution or stabilization, but not more than 30 days from last injection of investigational product or lumbar puncture.

10.5 Responsibilities for Reporting Serious Adverse Experiences

- The Investigator should notify the CTCC Project Manager (PM) by telephone within 24 hours of his/her becoming aware of the occurrence of a serious adverse experience. The PM will in turn notify the CTCC Clinical Monitor. The site Coordinator will fill out the MedWatch form (Lilly form for AV-133) provided by the CTCC, and email it to the CTCC Project Manager.

- Upon completion of the telephone report, the CTCC Project Manager will enter the appropriate subject information into the Incident Module.

- The following information should be supplied if available at the time of the telephone call: study number, site number, subject number, subject age and gender, date of onset of event, event description, whether event required treatment, death and autopsy report, an identification of which criteria for a serious experience have been met, the Investigator’s current opinion of the relationship between the event and study participation.

- The Investigator will comply with his/her local Institutional Review Board (IRB) and Radiation Safety Committee regulations regarding the reporting of adverse experiences.

- Notwithstanding the estimated data availability timeframe, the CTCC/IND will report to sponsors of companion studies involving therapeutic agents per the respective protocol (e.g., Lilly within 24 hours of notification from the site of an occurrence of any SAEs occurring within 48 hours post ^{18}F-AV-133 injection; likewise to Piramal for [^{18}F] florbetaben).

11. Reportable Events

The following incidents will be considered reportable events and will be reported to the CTCC within 24 hours of the event, or the Site Investigator's knowledge of the event.

- Initiation of PD medication
- Change of clinical diagnosis (includes phenoconversion to PD for Prodromal, Genetic Cohort-unaffected and Registry-unaffected subjects)
- Participation in any other clinical trial or study
- Premature withdrawal (e.g. withdrawal of consent)
- Serious Adverse Event (occurring in the appropriate AE follow up window)
- Pregnancy (reported by female subject or female partner of a male subject)
- Death

12. Referrals

If a research assessment, lab or MRI result reveals a clinically significant abnormality (e.g., indication of suicidality, depression, or renal impairment on metabolic profile) the subject should be informed of this result and instructed to follow up with his or her primary care physician. Should there be a safety concern warranting a referral for medical or psychiatric follow-up, the Investigator should provide the subject with the appropriate referral as necessary.

13. Potential Risks

13.1 Blood Sampling

Risks associated with venous blood draw include pain and bruising at the site where the blood is taken. Sometimes people can feel lightheaded or even faint after having blood drawn.

13.2. Lumbar Puncture

The most common risks of a lumbar puncture are pain at the site and a temporary headache usually due to a small amount of CSF leakage around the needle insertion site. Lying down for a few hours after the test can make a headache less likely to occur. There is a slight risk of infection because the needle breaks the skin's surface, providing a possible portal of entry for bacteria. A temporary numbness to the legs or lower back pain may be experienced. There is a small risk of bleeding in the spinal canal. Subjects will have blood drawn at Screening to test for coagulopathies.

13.3. MRI / DTI

Subjects should notify the study doctor if they suffer from claustrophobia because they may become anxious while in the magnetic resonance scanner. There may be loud noises such as knocking or hammering that occur while the MRI is being conducted. Subjects should also inform the study doctor if they have a pacemaker or metal implants (screws, plates or clips) because this may preclude MR evaluation.

13.4. Imaging

Specific potential risks for dopamine transporter SPECT imaging and VMAT-2 PET imaging are as follows:

1) radiation exposure from DaTSCAN™, ^{18}F-AV-133, or [^{123}I]β-CIT, the ^{57}Co transmission source, and from ^{57}Co containing markers,
2) potential pharmacological effects of DaTSCAN™,
3) having an intravenous injection.

Risks of DaTSCAN™: DaTSCAN™ is administered at radiotracer doses and is not expected to have any pharmacological or toxicological effects. DaTSCAN™ binds to the dopamine and serotonin transporter. At pharmacologic doses DaTSCAN™ might be expected to have stimulant-like effects and affect cardiovascular responses. However, in the proposed study the estimated mass dose of DaTSCAN™ is very low - <30/pmol kg. More than 180,000 doses of the drug have been administered to human subjects.

Risks of [^{18}F] AV-133: Refer to ^{18}F-AV-133-PPMI companion protocol Section 6.

Risks of [^{18}F] florbetaben: Refer to [^{18}F] florbetaben-PPMI companion protocol Section 2.3.

Risks of [^{123}I]β-CIT: The U.S. Food and Drug Administration (FDA) has established guidelines for the radiation dose considered acceptable for determining the distribution of radiotracer compounds in normal adult research subjects. The radiation exposure from this study is within the limits specified by the FDA. [^{123}I]β-CIT has been given to over 2000 subjects. In a few subjects (less than 1%) minor headaches and metallic taste in the mouth have occurred. It is possible that unexpected side effects could develop.

Iodine: Prior to each injection subjects will be pretreated with Lugol's solution, 10 drops of a saturated solution of potassium iodide) to reduce thyroid uptake ofthe radioactive agent. Subjects may experience a metallic or bitter taste in their mouths from the iodine. Subjects with allergies to iodine might get itching, a rash, bloating, severe blood pressure changes (shock), and death if given iodine. Subjects who are allergic to iodine will be administered potassium perchlorate rather than Lugol's.

13.5 . Risk of Disclosure of Genetic Information

All genetic information will be maintained in a confidential research file. While every effort will be made to maintain confidentiality there is a small risk that information will be disclosed. A Federal law, called **the Genetic Information Nondiscrimination Act (GINA)** generally makes it illegal for health insurance companies, group health plans and most employers to discriminate based on genetic information. This law generally provides that

- Health insurance companies and group health plans may not request an individual's genetic information from this research.

- Health insurance companies and group health plans may not use genetic information when making decisions regarding eligibility or premiums.
- Employers with 15 or more employees may not use genetic information from this research when making a decision to hire, promote, or fire an individual or when setting the terms of an individual's employment.

However, this law does not protect the subject from genetic discrimination by companies that sell life insurance, disability insurance, or long-term care insurance.

13.6 Unknown Risks

In addition to the known risks listed above, the imaging procedures in this study may cause unknown risks to the participant, or a developing embryo or fetus or possible risks to the future offspring of male participants. Female subjects or a female partner of a male subject who report a pregnancy within 30 days of DaTSCAN™ injection will be asked to have a urine pregnancy test.

13.7 Genetic Testing

There is a small risk of laboratory error with the genetic tests performed as a part of the pre-screening for participation in the Genetic Cohort and Genetic Registry. There is also a risk that the test may not work and will need to be repeated. In some cases where testing needs to be repeated, a new sample may need to be acquired. Up to two samples (blood or saliva) for DNA analysis will be collected from each subject. Either one or both samples may be used for quality control/quality assurance of the testing process to guard against such a risk.

13.8 Risks of gait and mobility assessments: Refer to ***Characterization of Motor Function and Potential Transition States to Parkinson's Disease in LRRK2 mutation Carriers and Controls*** companion protocol.

13.9 Refer to **Passive monitoring of individuals through wearable sensors or non-invasive methods (Sensor-PPMI sub-study)** protocol for risks associated with that companion study.

14. Statistical Methods

14.1 Statistical Design

PPMI is a longitudinal, multi-center study to assess progression of clinical features and imaging and biomic biomarkers in Parkinson disease patients and healthy controls. PD, Prodromal (including the Prodromal and Unaffected Genetic Cohort) and control subjects will be evaluated longitudinally for at least 36 months.

14.2 Primary Objective

To estimate the mean rates of change and the variability around the mean of clinical, imaging and biomic outcomes in early PD patients, and where appropriate to compare these rates between various subsets (including, but not limited to: early PD subjects vs. healthy subjects, PD vs. SWEDD, PD vs. Prodromal, PD with and without LRRK2, GBA or SNCA mutation, unaffected LRRK2, GBA or SNCA mutation carriers vs. healthy subjects) at study intervals ranging from 3 months to 36 months.

14.3 Secondary Objectives

14.3.1 Comparison between the rates of change in the mean of clinical, imaging and biomic outcomes in various subsets (including, but not limited to: early PD patients and vs. healthy subjects, PD vs. SWEDD, PD vs. Prodromal, PD with and without LRRK2, GBA or SNCA mutation, unaffected LRRK2, GBA or SNCA mutation carriers vs. healthy subjects) at study intervals ranging from 3 months to 156 months.

14.3.1. To determine the prevalence of measures of clinical, imaging and biomic outcomes in various subsets (including, but not limited to: early PD patients vs. healthy subjects PD vs. SWEDD, PD vs. Prodromal, PD with and without LRRK2, GBA or SNCA mutation, unaffected LRRK2, GBA or SNCA mutation carriers vs. healthy subjects) at study intervals ranging from baseline to 156 months.

14.3.2. To establish the predictive value of early clinical non-motor features, baseline imaging and biomic outcomes for future course of disease.

14.3.3. To examine the proportion of SWEDD subjects that have a change in their clinical management at 24 months (SWEDD Clinical Diagnosis and Management Questionnaire).

14.3.4. To examine the proportion of Prodromal subjects with one or more risk characteristics [hyposmia (<10th percentile by age and gender), RBD, or LRRK2, GBA or SNCA mutation, and baseline DaTSCAN binding showing minimal to moderate DAT deficit who phenoconvert within two years. To correlate the baseline DaTSCAN binding with risk of phenoconversion

14.3.5. To conduct exploratory analyses to examine whether the baseline DaTSCAN binding or progression of clinical, imaging, or biospecimen biomarkers will predict those subjects likely to phenoconvert.

14.3.6. Exploratory analysis of SWEDD subjects with substantial evidence of PD followed for longer than 2 years compared to PD subjects

14.4. Planned Analyses

Information summarizing planned analyses is described below. Further details regarding the analyses and plans for additional analyses will be contained in the PPMI Data Analysis Plan.

14.4.1. **Comparison of baseline characteristics among various subsets.** The first set of analyses will involve a comparison of baseline characteristics among the various subsets enrolled into the study. Continuous variables will be examined using a t-test and dichotomous variables will be examined using a chi-square test. Appropriate assumptions will be assessed for each comparison and any necessary adjustments (i.e., transformations) will be made prior to analysis. All analyses will be conducted at the 0.05 level.

14.4.2. **Comparison of short-term change in progression endpoints.** The second set of analyses will examine the short-term change during the first six months

for each progression endpoint. For continuous progression endpoints, the change over time will be modeled using a mixed model approach. For dichotomous progression endpoints, a logistic regression model will be fit. For each 'full' model of interest, an initial model will consist of all baseline characteristics, an indicator variable for whether the subject is a PD patient or healthy control, and all possible two-way interactions. We will utilize backwards selection to build a model for each progression endpoint.

14.4.3. **Examination of whether short-term change in progression endpoints is predictive of change in long-term endpoints.** The third set of analyses will examine whether short-term changes in the progression endpoints are predictive of changes in long-term endpoints in the MDS-UPDRS score. This analysis will examine a subset of the progression endpoints. Only progression endpoints that show differences between subsets will be considered (since for example, a marker that doesn't distinguish PD patients from healthy subjects would not be thought to be a biomarker for long-term outcome in PD patients). All progression endpoints that meet these criteria will be modeled using a process similar to that described above. The primary focus will be on the long-term change in the UPDRS score. However, additional long-term endpoints may be considered as well. Because this modeling process involves longer term endpoints, which would be much harder to replicate, a ten-fold cross validation procedure will be used to test the predictive validity of each model. If successful, the final model will provide a subset of one or more short-term progression endpoints that are predictive of the change in one or more of the long-term endpoints. This would suggest that these short-term progression endpoints are valid biomarkers for future studies of interventions in PD patient populations.

14.4.4. **Examination of PD Subsets.** Each of the first three sets of analyses will be repeated comparing subsets of PD subjects, rather than PD subjects vs. healthy subjects. If successful, the final model from these subset comparisons will determine whether some of the short-term progression endpoints are more predictive of long-term change in the MDS-UPDRS score for some subsets of PD subjects and less predictive for other subsets of PD subjects.

14.4.5. **Calculation of the proportion of SWEDD subjects that have a change in diagnosis over the 24-month evaluation period.** The analysis will involve identifying all SWEDD subjects that have a change in diagnosis from PD to another diagnosis. The percentage and 95% confidence interval will be reported. The other possible diagnoses will be further divided into 2 categories: 1) Other parkinsonian syndrome with a dopamine transporter deficit; 2) Other condition without a dopamine transporter deficit. The same information will be reported as well by these two categories.

14.4.6. **Exploratory analysis of Prodromal subjects that phenoconvert.** The analysis will involve estimating the percentage of Prodromal subjects that

phenoconvert to PD within 2 years. The percentage and 95% confidence interval will be reported.

14.4.7. **Exploratory analyses of biomarkers for predicting phenoconversion among Prodromal Subjects.** This set of analyses will examine whether short-term changes in the progression endpoints are predictive of phenoconversion among Prodromal subjects. All progression endpoints will be considered. The examination will involve fitting a logistic regression model for phenoconversion, with each progression endpoint serving as a covariate.

14.5. Determination of Sample Size

As summarized above, much of the proposed analysis plan for the PPMI study is focused on a set of exploratory analyses with the goal of identifying short-term progression markers that can be used as biomarkers for future studies in PD patient population. Because of the exploratory nature of these analyses, it is very difficult to provide a formal sample size justification for the entire model building process. Furthermore, the PPMI study has a broad range of goals that reach beyond any single, pre-planned analysis. However, we can examine the ability of the proposed sample size to detect meaningful effects of interest for the preliminary comparisons of baseline characteristics and univariate assessments of progression markers across the groups of interest.

The table below provides generic information about the detectable effect sizes for three types of statistical analysis that may be performed on the PPMI data. For each analysis the two-sided alpha level is set to 0.05 and the beta level to 0.80. The first column gives the total sample size assumed to be available for the analysis, in the first two rows either 400 (total PD sample) or 300 (PD sample after allowance for 25% withdrawals). The third and fourth rows of the table correspond to the total sample size of 600 (400 PD, 200 HC) when, respectively 75% and 100% of the subjects are available for analysis. The second column gives the detectable correlation coefficient between two continuous measures (e.g., change in striatal β-CIT uptake vs. change in total MDS-UPDRS). The third column gives the detectable difference in prevalence rates of some characteristic (e.g. presence of dopaminergic side effects) between two “halves” of the sample (e.g. the younger patients vs. the older patients, with age dichotomized at the median for the entire group). For the first two rows the third column gives the detectable “effect size”, expressed as ratio of difference in means to standard deviation, for comparing two “halves” of the sample (e.g. younger vs. older patients as above) in relation to a continuous measure (e.g. change in total MDS-UPDRS). For the third and fourth row the comparisons are between PD patients and HC. The table suggests that the PPMI trial is adequately powered to detect effects that would generally be of clinical interest. While possible that smaller effects than those listed in the table might also be of clinical interest, it was determined that the added power for these comparisons did not offset any additional costs and logistical issues that would accompany a larger study in this population. Rather, the proposed study should prove to be effective for screening a large number of variables and identifying those that show the most promise for further exploration in follow-up studies.

**Total sample size**	**Detectable Correlation**	**Detectable Difference in Prevalence**	**Detectable Difference in Means (Standardized)**
**300**	**0.16**	**17%**	**0.33**
**400**	**0.14**	**14%**	**0.28**
**450**	**0.14**	**15%**	**0.28**
**600**	**0.11**	**13%**	**0.24**

The planned sample size of approximately 80 SWEDD subjects is based on the estimated percentage of SWEDD subjects that will be observed over the recruitment period for the PPMI study. This is based on preliminary data from the study suggesting that about 20% of subjects screened with parkinsonian symptoms have no evidence for dopamine transporter deficit. The clinical and biomarker data from the SWEDD subjects will serve as a disease control population and will allow for a number of important exploratory assessments comparing the SWEDD subjects to the PPMI PD subjects and Healthy Controls.

The planned sample size of approximately 100 for the prodromal cohort is based primarily on prior exploratory studies. Nevertheless, based on preliminary data from studies combining olfactory loss, RBD, or LRRK2 mutation with DAT imaging deficit to define an at-risk population, we estimate 30% phenoconversion during the 24 month evaluation post DAT imaging. Under this assumption, the sample size will allow estimating the phenoconversion rate with a 95% confidence interval width of approximately ± 10%. It is further anticipated that the change in clinical, imaging, and biomarker data from the Prodromal cohort will allow for a number of important exploratory assessments comparing Prodromal subjects who convert to Prodromal subjects who do not convert. Finally, additional exploratory assessments will compare the Prodromal subjects to the PPMI PD subjects, Healthy Controls, and SWEDD subjects.

The planned sample size of 600 subjects with either a LRRK2, GBA or SNCA mutation is based primarily on exploratory studies and considerations regarding the availability of subjects with PD and family members with these genetic mutations. The clinical and biomarker data from the Genetic Cohort will allow for a number of important exploratory assessments comparing the genetic PD and early PD subjects and the prodromal cohort with the unaffected Genetic Cohort. The Genetic Registry will allow exploratory assessments of genetic subjects with more advanced PD and of family members who are unable to participate in the Genetic Cohort.

15. Regulatory / Ethics

15.1. Compliance Statement

This study will be conducted in accordance with the Good Clinical Practice (GCP) and the International Conference on Harmonization (ICH) guidelines and any applicable national and local regulations.

All procedures not described in this protocol will be performed according to the study Operation Manuals unless otherwise stated. Laboratory tests/evaluations described in

this protocol will be conducted in accordance with quality laboratory standards as described in the central laboratory manual unless otherwise stated.

15.2. Informed Consent

In accordance with relevant regulations, an informed consent agreement explaining the procedures and requirements of the study, together with any potential hazards/risks must be read by and/or explained to each subject. Each subject will sign such an informed consent form. The subject must be assured of the freedom to withdraw from participation in the study at any time. In addition, a Certificate of Confidentiality has been obtained to provide additional confidentiality protections for subjects at participating PPMI sites in the United States.

It is the Investigator's responsibility to make sure that the subject understands what she/he is agreeing to and that written informed consent is obtained before the subject is involved in any protocol-defined procedures including screening procedures. It is also the Investigator's responsibility to retain the original signed consent form and provide each subject with a copy of the signed consent form.

The CTCC must be given an opportunity to review the consent forms prior to site IRB submission and before it is used in the study.

Subjects will be encouraged to complete an Advance Directive for Clinical Research Participation, if willing, to document their wishes about participation in PPMI should they no longer be able to give informed consent. The advance directive permits a Substitute Decision Maker to be named to help make decisions for the subject concerning his/her participation in the PPMI study in the event the subject is no longer able to decide for him/herself (see Section 9.1).

15.3. Institutional Review Board/Independent Ethics Committee

The CTCC will supply all necessary information to the Investigator for submission of the protocol and consent forms to the IRB/IEC for review and approval. The Investigator agrees to provide the IRB/IEC all appropriate material. The trial study will not begin until the Investigator has obtained appropriate IRB/IEC approval. A copy of the approval letter and approved consent form must be submitted to the CTCC.

The Investigator will request from the IRB/IEC a composition of the IRB members reviewing the protocol and informed consents. Appropriate reports on the progress of this study by the Investigator will be made to the IRB/IEC and the CTCC in accordance with institutional and government regulations. The CTCC will notify the site when the IRB/IEC may be notified of study completion. It is the Investigator's responsibility to notify the IRB when the study ends. This includes study discontinuation, whether it is permanent or temporary. A copy of the site IRB/IEC's acknowledgement of study completion must be submitted to the CTCC.

15.4. Protocol Amendments

Changes to the protocol should only be made via an approved protocol amendment. Protocol amendments must be approved by the Sponsor, the study's Steering Committee and each respective site's IRB/IEC prior to implementation, except when necessary to eliminate hazards and/or to protect the safety, rights or welfare of subjects.

15.5. Subject Confidentiality

The site Investigator must assure that the confidentiality of subjects, including their personal identity and personal medical information, will be maintained at all times. U.S. sites have additional confidentiality obligations to study subjects under the Health Insurance Portability and Accountability Act (HIPAA). Subjects will be identified by code numbers on case report forms and other study materials submitted to the CTCC, the central laboratory, and central biorepository.

After a subject signs an informed consent, it is required that the site Investigator permit the study monitor or regulatory agency personnel to review the signed informed consent(s) and that portion of the subject's medical record that is directly related to the study. This shall include all study relevant documentation including subject medical history to verify eligibility, laboratory test result reports, admission/discharge summaries for hospital admissions occurring while the subject is in the study, and autopsy reports for deaths occurring during the study (when available).

16. Documentation

16.1. Study File and Site Documents

The Investigator should have the following study documents accessible to the Monitor during the study.

- *Curriculum vitae* for investigator and coordinator
- The signed IRB/IEC form/letter stating IRB/IEC approval of protocol, consent forms, and advertisement notices, documentation of the IRB/IEC composition, and all IRB/IEC correspondence including notification/approval of protocol amendments, notification of serious adverse events to the IRB/IEC, and IRB/IEC notification of study termination
- IRB/IEC approved consent forms (sample) and advertisements as applicable
- Signed protocol (and amendments, where applicable)
- Signed subject consent forms
- Copies of the completed CRF worksheets
- Delegation Log with names, signatures, initials and functional role of all persons completing protocol assessments, providing back-up to the site Investigator and Coordinator, if applicable, as well as staff entering data to the eClinical system.
- Laboratory accreditation and relevant laboratory reference ranges
- Copies of laboratory reports/printouts
- Any source data/records not kept with the subject's hospital/medical records
- Signed and dated receipt of supplies
- Record of all monitoring visits

- Copies of correspondence to and from CTCC
- Investigator’s Brochure (where applicable)
- Certificate for Human Subject Protection Program (HSPP) or equivalent GCP program for each individual named on the Delegation log who has direct subject contact
- Copy of professional licensure/registration, as applicable, for each individual who has direct subject contact ensuring licensure is in the state/region in which the study will be conducted
- A Note to File indicating the assessments that will be considered source documents
- Any other documentation as required by the CTCC (e.g., Conflict-of-Interest/Financial Disclosure)

The Investigator must also retain all printouts/reports of tests/procedures, as specified in the protocol, for each subject. This documentation, together with the subject’s hospital/medical records, is the subject’s source information for the study.

16.2. Maintenance and Retention of Records

It is the responsibility of the Site Investigator to maintain a comprehensive and centralized filing system of all relevant documentation. Site Investigators must retain all study records required by the CTCC and regulatory authorities in a secure and safe facility with limited access. The Site Investigator will be instructed to consult with the CTCC before disposal of any study records and to notify the CTCC of any change in the location, disposition, or custody of the study files.

An electronic case report form (eCRF) utilizing an Electronic Data Capture (EDC) application will be used for this study. In the event of an audit or regulatory authority inspection, the eCRFs can be printed out.

16.3. Case Report Forms

Sites will enter subject information and data into the eCRF in the EDC application. The eCRFs are used to record study data and are an integral part of the study and subsequent reports. Therefore the eCRFs must be completed according to the subject’s source data on a per-visit basis for each subject screened or enrolled. Authorized study personnel will each be granted access to the electronic data capture tool via provision of a unique password-protected user-ID that will limit access to enter and view data specifically for subjects enrolled at their site. Timely data entry is considered to be data entered into the EDC system within 14 days of a subject’s visit.

Sites will have access to download worksheets that correspond to the electronic case report form (eCRF). The worksheets will serve as source documents as described in the Operations Manual and are to be used to enter data into the eCRFs. Sites will enter all data into the subject’s medical chart and/or onto source documentation worksheets prior to entering data into the eCRFs via computer stations connected remotely to the CTCC’s central server through an Internet browser.

<u>Electronic Signatures</u>:
An electronic signature from the site Investigator (or delegated Sub-investigator) is required on the following eCRFs:

- Signature Form
- Adverse Event Form

It is the site Investigator's responsibility to ensure that entries are proper and complete. During entry of data, error checks will be performed by the EDC system that will immediately flag problematic data (i.e., missing, out of range, inconsistent), allowing for sites to correct the data at that time. Error checks will be implemented in the EDC system based upon specifications defined in the data management plan.

16.4. Primary Source Documents

The Investigator must maintain primary source documents supporting data collected for each subject. This includes documentation of:

- General information supporting the subject's consent to participant in the study
- Demographic information
- Evidence supporting the diagnosis/condition for which the subject is being studied
- General history and physical findings
- Hospitalization or Emergency Room records (if applicable)
- Each study visit by date, including any relevant findings/notes by the Investigator(s), occurrence (or lack) of adverse events, and changes in medication usage including the date the medication commenced and completed
- Any additional visits during the study
- Any relevant telephone conversations with the subject regarding the study or possible adverse experiences
- Original, signed informed consent forms for study participation

During monitoring visits the study monitor will need to validate data in the eCRFs against these source data.

17. Data Management

An Internet accessible Electronic Data Capture (EDC) system for data management will be utilized for this study. This system is protected by 128-bit server certificates and utilizes authenticated, password-protected accounts for each site. The EDC system is designed to ensure timeliness and accuracy of data as well as the prompt reporting of data from the study on an ongoing basis to the study principal investigators. The system is compliant with relevant FDA regulatory requirements per 21 CFR Part 11.

Data review, coding and query processing will be done through interaction with the CTCC, site personnel and the Study Monitor. Queries will be generated in real-time as data are entered. Once the data are submitted to the EDC system, they are immediately stored in the central study database located at the CTCC and are accessible for review by data management staff. Any changes to the data will be fully captured in an electronic audit trail. As data recorded by sites in eCRFs are received, narrative text of adverse experiences and concomitant medications will be periodically coded using established coding mechanisms.

The cycle of electronic data entry, review, query identification/resolution, and correction occurs over the course of the study period until all subjects have completed the study.

Data will be securely transferred to the Statistics Core. Once the Statistics Core and the CTCC, in conjunction with the Sponsor and the principal investigator, agree that all queries have been adequately resolved and the database has been deemed "clean." The database will be officially signed off and deemed locked. All permissions to make changes (append, delete, modify or update) the database are removed at this time.

All data obtained during the conduct of the PPMI study, will be sent to the Laboratory of Neuro Imaging (LONI) in Los Angeles, California to be stored indefinitely for research purposes. Research data will be made available to researchers to conduct analyses related to PD and other disorders.

Data obtained from subjects participating in the finger tapping testing (Objective Parkinson's Disease Measurement) will be saved during the testing period onto a portable data drive specific to the OPDM device and transferred to the secure server of the Kinetics Foundation via a secured file transfer program (FTP) link. The transferred data will be encrypted and will contain the PPMI subject ID. When the data is uploaded at Kinetics, each data session will include the date, start and end time of the objective motor test battery and an identification number for the machine with which the test was administered. OPDM data will be transferred from Kinetics to the CTCC for verification and quality control. After completing quality checks, the data will be transferred as part of the PPMI data set to the Laboratory of Neuro Imaging (LONI) at the University of Southern California (UCSC) to be stored indefinitely for research purposes. Research data obtained from the OPDM will be made available to researchers to conduct analyses related to PD and other disorders.

Data obtained from subjects participating in the *Characterization of Motor Function and Potential Transition States to Parkinson's Disease in LRRK2 mutation Carriers and Controls* companion study will be collected using wearable devices. The raw data from these devices will be downloaded and transferred to the study team at Tel Aviv Medical Center. The study team will then process this data and transfer it to Laboratory of Neuro Imaging (LONI) at the University of Southern California (UCSC) to be stored indefinitely for research purposes.

Data from the ***Passive monitoring of individuals through wearable sensors or non-invasive methods (Sensor-PPMI sub-study)*** companion study will be collected through wearable sensors or other non-invasive devices. The data collected by each wearable sensor or device will be stored locally on non-volatile memory. Data will be uploaded to a secure platform, where it can be accessed for analysis by the PPMI team or collaborators. Data collected as part of a PPMI companion study will ultimately be transferred to and linked to the PPMI study data set, and be accessible to researchers through Laboratory of NeuroImaging (LONI).

18. Study Monitoring

In accordance with ICH Guidelines for Good Clinical Practice 5.18 the study will be monitored to verify that:

(a) The rights and well being of human subjects are protected.
(b) The reported trial data are accurate, complete, and verifiable from source documents.
(c) The conduct of the trial is in compliance with the currently approved protocol/amendment(s), with GCP, and with the applicable regulatory requirement(s).

The Steering Committee has the responsibility to monitor all procedures for safety and for GCP and regulatory compliance. The committee members have the expertise to monitor all aspects of this study.

Study data will also be provided to the Clinical Study Oversight Committee (CSOC). The CSOC will convene periodically to review safety data. Any recommendations for changes to the conduct of the study will be conveyed to the Steering Committee.

The study will have ongoing monitoring to ensure that the trial is conducted properly. The monitoring activities will include:

- Verifying that the site investigators and coordinators have adequate qualifications, that resources remain adequate throughout the trial period, and that facilities, equipment, and staff are adequate to safely and properly conduct the trial.
- Verifying that the site investigators follow the approved protocol and all approved amendment(s), if any.
- Verifying that written consent was obtained for each subject participating in the trial.
- Verifying that the investigators are enrolling only eligible subjects.
- Verifying that source documents and other trial records are accurate, complete, kept up-to-date and maintained.
- Verifying that the investigator provides all the required reports, notifications, applications, and submissions, and that these documents are accurate, complete, timely, legible, dated, and identify the trial.
- Monitoring adverse events, concomitant medications and intercurrent illnesses. Determining whether all adverse events (AEs) are appropriately reported within the time periods required by GCP, the protocol, the IRB, and other applicable regulatory requirement(s).
- Communicating deviations from the protocol, Standard Operating Procedures, GCP, and the applicable regulatory requirements to the investigator and taking appropriate action designed to prevent recurrence of the detected deviations.
- Federal regulations 21 CFR §56.109(f) and 45 CFR §46.109(e) state that an IRB shall conduct continuing review of research covered by these regulations at intervals appropriate to the degree of risk, but not less than once per year, and shall have authority to observe or have a third party observe the consent process and the research. Continuing review by the IRB routinely includes interim progress reports, as directed by the Board, review of proposed changes to research, adverse event reports, review of any protocol deviations, visits to the research site, and annual review of the research.

10. Publication of Research Findings

Publication of results of this study will be governed by the policies and procedures developed by the PPMI Steering Committee (PMI Publications Policy www.ppmi-info.org) and in accordance

with the International Committee of Medical Journal Editors (ICJME) Uniform Requirements for Manuscripts Submitted to Biomedical Journals.

References

1 Mueller, S. G. *et al.* Ways toward an early diagnosis in Alzheimer's disease: the Alzheimer's Disease Neuroimaging Initiative (ADNI). *Alzheimer's & dementia : the journal of the Alzheimer's Association* **1**, 55-66, doi:10.1016/j.jalz.2005.06.003 (2005).

2 Mixed lineage kinase inhibitor CEP-1347 fails to delay disability in early Parkinson disease. *Neurology* **69**, 1480-1490, doi:10.1212/01.wnl.0000277648.63931.c0 (2007).

3 Jankovic, J. *et al.* Variable expression of Parkinson's disease: a base-line analysis of the DATATOP cohort. The Parkinson Study Group. *Neurology* **40**, 1529-1534 (1990).

4 Klein, C. & Schlossmacher, M. G. Parkinson disease, 10 years after its genetic revolution: multiple clues to a complex disorder. *Neurology* **69**, 2093-2104, doi:10.1212/01.wnl.0000271880.27321.a7 (2007).

5 Ponsen, M. M. *et al.* Idiopathic hyposmia as a preclinical sign of Parkinson's disease. *Annals of neurology* **56**, 173-181, doi:10.1002/ana.20160 (2004).

6 Eckert, T. & Eidelberg, D. The role of functional neuroimaging in the differential diagnosis of idiopathic Parkinson's disease and multiple system atrophy. *Clinical autonomic research : official journal of the Clinical Autonomic Research Society* **14**, 84-91, doi:10.1007/s10286-004-0167-1 (2004).

7 Siderowf, A. & Stern, M. B. Preclinical diagnosis of Parkinson's disease: are we there yet? *Current neurology and neuroscience reports* **6**, 295-301 (2006).

8 Stern, M. B. The preclinical detection of Parkinson's disease: ready for prime time? *Annals of neurology* **56**, 169-171, doi:10.1002/ana.20180 (2004).

9 Fahn, S. *et al.* Levodopa and the progression of Parkinson's disease. *The New England journal of medicine* **351**, 2498-2508, doi:10.1056/NEJMoa033447 (2004).

10 Whone, A. L. *et al.* Slower progression of Parkinson's disease with ropinirole versus levodopa: The REAL-PET study. *Annals of neurology* **54**, 93-101, doi:10.1002/ana.10609 (2003).

11 Marek, K. Beta-CIT Scans without evidence of dopaminergic deficit (SWEED) in the ELLDOPA-CIT and CALM-CIT study: long-term imaging assessment. *Neurology* **20**, A298 (2003).

12 Dopamine transporter brain imaging to assess the effects of pramipexole vs levodopa on Parkinson disease progression. *JAMA : the journal of the American Medical Association* **287**, 1653-1661 (2002).

13 Berg, D. Transcranial sonography in the early and differential diagnosis of Parkinson's disease. *Journal of neural transmission. Supplementum*, 249-254 (2006).

14 Gerhard, A. *et al.* In vivo imaging of microglial activation with [11C](R)-PK11195 PET in idiopathic Parkinson's disease. *Neurobiology of disease* **21**, 404-412, doi:10.1016/j.nbd.2005.08.002 (2006).

15 Mollenhauer, B. & Trenkwalder, C. Neurochemical biomarkers in the differential diagnosis of movement disorders. *Movement disorders : official journal of the Movement Disorder Society* **24**, 1411-1426, doi:10.1002/mds.22510 (2009).

16 Tokuda, T. *et al.* Decreased alpha-synuclein in cerebrospinal fluid of aged individuals and subjects with Parkinson's disease. *Biochemical and biophysical research communications* **349**, 162-166, doi:10.1016/j.bbrc.2006.08.024 (2006).

17 Schwarzschild, M. A. *et al.* Serum urate as a predictor of clinical and radiographic progression in Parkinson disease. *Archives of neurology* **65**, 716-723, doi:10.1001/archneur.2008.65.6.nct70003 (2008).

18 Asenbaum, S. *et al.* Imaging of dopamine transporters with iodine-123-beta-CIT and SPECT in Parkinson's disease. *Journal of nuclear medicine : official publication, Society of Nuclear Medicine* **38**, 1-6 (1997).

19 Booij, J. *et al.* Imaging of dopamine transporters with iodine-123-FP-CIT SPECT in healthy controls and patients with Parkinson's disease. *Journal of nuclear medicine : official publication, Society of Nuclear Medicine* **39**, 1879-1884 (1998).

20 Brooks, D. J. *et al.* Differing patterns of striatal 18F-dopa uptake in Parkinson's disease, multiple system atrophy, and progressive supranuclear palsy. *Annals of neurology* **28**, 547-555, doi:10.1002/ana.410280412 (1990).

21 Eidelberg, D. *et al.* Early differential diagnosis of Parkinson's disease with 18F-fluorodeoxyglucose and positron emission tomography. *Neurology* **45**, 1995-2004 (1995).

22 Frey, K. A. *et al.* Presynaptic monoaminergic vesicles in Parkinson's disease and normal aging. *Annals of neurology* **40**, 873-884, doi:10.1002/ana.410400609 (1996).

23 Innis, R. B. *et al.* Single photon emission computed tomographic imaging demonstrates loss of striatal dopamine transporters in Parkinson disease. *Proceedings of the National Academy of Sciences of the United States of America* **90**, 11965-11969 (1993).

24 Mozley, P. D. *et al.* Binding of [99mTc]TRODAT-1 to dopamine transporters in patients with Parkinson's disease and in healthy volunteers. *Journal of nuclear medicine : official publication, Society of Nuclear Medicine* **41**, 584-589 (2000).

25 Vingerhoets, F. J. *et al.* Positron emission tomographic evidence for progression of human MPTP-induced dopaminergic lesions. *Annals of neurology* **36**, 765-770, doi:10.1002/ana.410360513 (1994).

26 Snow, B. J. *et al.* Human positron emission tomographic [18F]fluorodopa studies correlate with dopamine cell counts and levels. *Annals of neurology* **34**, 324-330, doi:10.1002/ana.410340304 (1993).

27 Kaufman, M. J. & Madras, B. K. Severe depletion of cocaine recognition sites associated with the dopamine transporter in Parkinson's-diseased striatum. *Synapse* **9**, 43-49, doi:10.1002/syn.890090107 (1991).

28 Niznik, H. B., Fogel, E. F., Fassos, F. F. & Seeman, P. The dopamine transporter is absent in parkinsonian putamen and reduced in the caudate nucleus. *Journal of neurochemistry* **56**, 192-198 (1991).

29 Wilson, J. M. *et al.* Differential changes in neurochemical markers of striatal dopamine nerve terminals in idiopathic Parkinson's disease. *Neurology* **47**, 718-726 (1996).

30 Booij, J. *et al.* [123I]FP-CIT SPECT shows a pronounced decline of striatal dopamine transporter labelling in early and advanced Parkinson's disease. *Journal of neurology, neurosurgery, and psychiatry* **62**, 133-140 (1997).

31 Fischman, A. J. *et al.* Rapid detection of Parkinson's disease by SPECT with altropane: a selective ligand for dopamine transporters. *Synapse* **29**, 128-141, doi:10.1002/(SICI)1098-2396(199806)29:2<128::AID-SYN4>3.0.CO;2-9 (1998).

32 Huang, W. S. *et al.* Evaluation of early-stage Parkinson's disease with 99mTc-TRODAT-1 imaging. *Journal of nuclear medicine : official publication, Society of Nuclear Medicine* **42**, 1303-1308 (2001).

33 Sawle, G. V., Playford, E. D., Burn, D. J., Cunningham, V. J. & Brooks, D. J. Separating Parkinson's disease from normality. Discriminant function analysis of fluorodopa F 18 positron emission tomography data. *Archives of neurology* **51**, 237-243 (1994).

34 Seibyl, J. P. *et al.* Decreased single-photon emission computed tomographic [123I]beta-CIT striatal uptake correlates with symptom severity in Parkinson's disease. *Annals of neurology* **38**, 589-598, doi:10.1002/ana.410380407 (1995).

35 Mozley, P. D. *et al.* Effects of age on dopamine transporters in healthy humans. *Journal of nuclear medicine : official publication, Society of Nuclear Medicine* **40**, 1812-1817 (1999).

36 van Dyck, C. H. *et al.* Age-related decline in dopamine transporters: analysis of striatal subregions, nonlinear effects, and hemispheric asymmetries. *The American journal of geriatric psychiatry : official journal of the American Association for Geriatric Psychiatry* **10**, 36-43 (2002).

37 Volkow, N. D. *et al.* Decreased dopamine transporters with age in health human subjects. *Annals of neurology* **36**, 237-239, doi:10.1002/ana.410360218 (1994).

38 Brooks, D. J. *et al.* Assessment of neuroimaging techniques as biomarkers of the progression of Parkinson's disease. *Experimental neurology* **184 Suppl 1**, S68-79 (2003).

39 Marek, K. *et al.* [123I]beta-CIT SPECT imaging assessment of the rate of Parkinson's disease progression. *Neurology* **57**, 2089-2094 (2001).

40 Morrish, P. K., Rakshi, J. S., Bailey, D. L., Sawle, G. V. & Brooks, D. J. Measuring the rate of progression and estimating the preclinical period of Parkinson's disease with [18F]dopa PET. *Journal of neurology, neurosurgery, and psychiatry* **64**, 314-319 (1998).

41 Nurmi, E. *et al.* Rate of progression in Parkinson's disease: a 6-[18F]fluoro-L-dopa PET study. *Movement disorders : official journal of the Movement Disorder Society* **16**, 608-615 (2001).

42 Nurmi, E. *et al.* Progression in Parkinson's disease: a positron emission tomography study with a dopamine transporter ligand [18F]CFT. *Annals of neurology* **47**, 804-808 (2000).

43 Pirker, W. *et al.* Progression of dopaminergic degeneration in Parkinson's disease and atypical parkinsonism: a longitudinal beta-CIT SPECT study. *Movement disorders : official journal of the Movement Disorder Society* **17**, 45-53 (2002).

44 Guttman, M. *et al.* [11C]RTI-32 PET studies of the dopamine transporter in early dopa-naive Parkinson's disease: implications for the symptomatic threshold. *Neurology* **48**, 1578-1583 (1997).

45 Marek, K. L. *et al.* [123I] beta-CIT/SPECT imaging demonstrates bilateral loss of dopamine transporters in hemi-Parkinson's disease. *Neurology* **46**, 231-237 (1996).

46 Ravina, B. *et al.* The role of radiotracer imaging in Parkinson disease. *Neurology* **64**, 208-215, doi:10.1212/01.WNL.0000149403.14458.7F (2005).

47 Wooten, G. F. Agonists vs levodopa in PD: the thrilla of whitha. *Neurology* **60**, 360-362 (2003).

48 Marek, K., Jennings, D., Tamagnan, G. & Seibyl, J. Biomarkers for Parkinson's [corrected] disease: tools to assess Parkinson's disease onset and progression. *Annals of neurology* **64 Suppl 2**, S111-121, doi:10.1002/ana.21602 (2008).

49 DeKosky, S. T. & Marek, K. Looking backward to move forward: early detection of neurodegenerative disorders. *Science* **302**, 830-834, doi:10.1126/science.1090349 (2003).

50 Fearnley, J. M. & Lees, A. J. Ageing and Parkinson's disease: substantia nigra regional selectivity. *Brain : a journal of neurology* **114 (Pt 5)**, 2283-2301 (1991).

51 Marek, K., Jennings, D., Tamagnan, G. & Seibyl, J. Biomarkers for Parkison's disease: tools to assess Parkinson's disease onset and progression. *Ann Neurol* **64 Suppl 2**, S111-121 (2008).

52 Parkinson Study Group. Levodopa and the progression of Parkinson disease. *NEMJ* **351**, 18-28 (2004).

53 Parkinson Study Group. Mixed lineage kinase inhibitor CEP-1347 fails to delay disability in early Parkinson disease. *Neurology* **69**, 1480-1490 (2007).

54 Fearnley, J. M. & Lees, A. J. Ageing and Parkinson's disease: substantia nigra regional selectivity. *Brain* **114 (Pt 5)**, 2283-2301 (1991).

55 Halliday, G., Lees, A. & Stern, M. Milestones in Parkinson's disease--clinical and pathologic features. *Movement disorders : official journal of the Movement Disorder Society* **26**, 1015-1021, doi:10.1002/mds.23669 (2011).

56 Lang, A. E. & Obeso, J. A. Challenges in Parkinson's disease: restoration of the nigrostriatal dopamine system is not enough. *Lancet neurology* **3**, 309-316, doi:10.1016/S1474-4422(04)00740-9 (2004).

57 Przuntek, H., Muller, T. & Riederer, P. Diagnostic staging of Parkinson's disease: conceptual aspects. *J Neural Transm* **111**, 201-216, doi:10.1007/s00702-003-0102-y (2004).
58 Halliday, G., Lees, A. & Stern, M. Milestones in Parkinson's disease--clinical and pathologic features. *Movement disorders : official journal of the Movement Disorder Society* **26**, 1015-1021, doi:10.1002/mds.23669 (2011).
59 Gasser, T. Genomic and proteomic biomarkers for Parkinson disease. *Neurology* **72**, S27-31, doi:10.1212/WNL.0b013e318198e054 (2009).
60 Pankratz, N. & Foroud, T. Genetics of Parkinson disease. *Genetics in medicine : official journal of the American College of Medical Genetics* **9**, 801-811, doi:10.1097GIM.0b013e31815bf97c (2007).
61 Sidransky, E. *et al.* Multicenter analysis of glucocerebrosidase mutations in Parkinson's disease. *The New England journal of medicine* **361**, 1651-1661, doi:10.1056/NEJMoa0901281 (2009).
62 Simon-Sanchez, J. *et al.* Genome-wide association study reveals genetic risk underlying Parkinson's disease. *Nature genetics* **41**, 1308-1312, doi:10.1038/ng.487 (2009).
63 Emre, M. *et al.* Clinical diagnostic criteria for dementia associated with Parkinson's disease. *Movement disorders : official journal of the Movement Disorder Society* **22**, 1689-1707; quiz 1837, doi:10.1002/mds.21507 (2007).
64 Johns, M. W. A new method for measuring daytime sleepiness: the Epworth sleepiness scale. *Sleep* **14**, 540-545 (1991).
65 Meara, J., Mitchelmore, E. & Hobson, P. Use of the GDS-15 geriatric depression scale as a screening instrument for depressive symptomatology in patients with Parkinson's disease and their carers in the community. *Age and ageing* **28**, 35-38 (1999).
66 Wechsler, D. *WMS-II Administration and Scoring Manual*. (The Psychological Corporation, 1997).
67 Benedict, R., Schretlen, D., Groninger, L. & Brandt, J. *The Hopkins Verbal Learning Test-Revised: Normative date and analysis of interform and test-retest reliability*. Vol. 12 43-55 (Clinical Neuropsychologist, 1998).
68 Spielberger, C. *Manual for the State-Trait Anxiety Inventory*. (Consulting Psychololgists Press, 1970).
69 Stiasny-Kolster, K. *et al.* The REM sleep behavior disorder screening questionnaire--a new diagnostic instrument. *Movement disorders : official journal of the Movement Disorder Society* **22**, 2386-2393, doi:10.1002/mds.21740 (2007).
70 Weintraub, D. *et al.* Validation of the questionnaire for impulsive-compulsive disorders in Parkinson's disease. *Movement disorders : official journal of the Movement Disorder Society* **24**, 1461-1467, doi:10.1002/mds.22571 (2009).
71 Visser, M., Marinus, J., Stiggelbout, A. M. & Van Hilten, J. J. Assessment of autonomic dysfunction in Parkinson's disease: the SCOPA-AUT. *Movement disorders : official journal of the Movement Disorder Society* **19**, 1306-1312, doi:10.1002/mds.20153 (2004).
72 Dubois, B. *et al.* Diagnostic procedures for Parkinson's disease dementia: recommendations from the movement disorder society task force. *Movement disorders : official journal of the Movement Disorder Society* **22**, 2314-2324, doi:10.1002/mds.21844 (2007).
73 Litvan, I. *et al.* MDS Task Force on mild cognitive impairment in Parkinson's disease: critical review of PD-MCI. *Movement disorders : official journal of the Movement Disorder Society* **26**, 1814-1824, doi:10.1002/mds.23823 (2011).
74 Spreen, O., & Benton, A. L. (1977). Neurosensory center comprehensive examination for aphasia: Manual of directions. revised edition. Vicotria, BC, Canada: Neuropsychology Laboratory, University of Victoria.
