# Supplementary material for: Autonomic dysfunction and white matter microstructural changes in drug-naïve patients with Parkinson’s disease
Source: PeerJ. 2018 Sep 13;6:e5539. doi: 10.7717/peerj.5539 (PMC6139241; doi:10.7717/peerj.5539)
Supplement: Supplemental Information 3 [file peerj-06-5539-s003.pdf]

PARKINSON'S PROGRESSION MARKERS INITIATIVE

Play a Part in Parkinson's Research

PPMI

OPERATIONS MANUAL

PARKINSON'S PROGRESSION MARKERS INITIATIVE

Play a Part in Parkinson's Research

TABLE OF CONTENTS

SECTION 1 **GENERAL INFORMATION**

Study Contacts 2
Study Personnel 4
CTCC Office Closings 5

SECTION 2 **PROTOCOL**

SECTION 3 **ENROLLMENT**

General Information 2
Subject ID Assignment 2
CTCC Unique ID 2
Activities Prior to Enrollment...3
Protocol Deviation Codes...4
Electronic Enrollment Process 4
Subject Identification Code List 8

SECTION 4 **IMAGING**

Ordering DaTSCAN™ (US Sites) 2
Shipment to Sites (US Sites)..5
Ordering DaTScan™ (EU Sites)...5
Storage of DaTSCAN™ 6
Administration or DaTSCAN™ 6
Accountability of DaTSCAN™ 6
DaTSCAN™ Interpretation Report 7

SECTION 4 APPENDIX

DaTSCAN Drug Order Form...9
DaTSCAN Drug Acknowledgement of Receipt.......................10
Investigational Medicinal Product Accountability Log 11
IND Visual Interpretation Report..12
Imaging Technical Operations Manual (TOM)

SECTION 5 **SOURCE DOCUMENTS WORKSHEETS AND eCRFs**

eClinical Training Manual...2
Access to EDC and Portal 2
Electronic Signatures 2
Source Document Worksheets 3
General Directions for eCRF Completion..............................5
Directions for PPMI eCRF Pages..7

PARKINSON'S PROGRESSION MARKERS INITIATIVE
Play a Part in Parkinson's Research

SECTION 6 **ASSESSMENTS**

Order of Assessments 2
Schedule of Alternate Forms 2
General Testing Guidelines 3
Equipment and Supplies 5
MDS-UPDRS with Hoehn & Yahr 6
Modified Schwab & England Activities of Daily Living 7
Physical Activity Scale for the Elderly (PASE) 8
Hopkins Verbal Learning Test – Revised (HVLT-R) 8
Benton Judgment of Line Orientation 12
Semantic Fluency 15
Letter Number Sequencing (LNS) 17
Symbol Digit Modalities Test 19
Montreal Cognitive Assessment (MoCA) 21
Epworth Sleepiness Scale (ESS) 26
REM Sleep Behavior Disorder Questionnaire 28
Geriatric Depression Scale (GDS-15) 30
State-Trait Anxiety Inventory for Adults 32
Questionnaire for Impulsive-Compulsive Disorders (QUIP) 34
Scales for Outcome in Parkinson's Disease (SCOPA-AUT) 36
Cognitive Categorization 38
Smell Identification Test (UPSIT) 38

SECTION 6 APPENDIX

Assessment Order Form 41
P-PPMI Phenoconversion Guide 42

SECTION 7 **REPORTABLE EVENTS**

Incident Reporting 2
Notification Reporting 3
Adverse and SAE Reporting 4

SECTION 8 **LABORATORY SPECIMEN MANAGEMENT**

Covance Lab Manual
Research Biomarkers Lab Manual

SECTION 9 **SOURCE DOCUMENTATION**

Instructions for Source Documentation 2

SECTION 9 APPENDIX

Source Documentation Worksheets

PARKINSON'S PROGRESSION MARKERS INITIATIVE

Play a Part in Parkinson's Research

SECTION 10 **MONITORING**

Study Initiation 2
Site Visits 2
Interim Monitoring Visit(s) 2
Clinical Site Close Out Visit 3
Contents of Regulatory Binder 3

SECTION 11 **REPORTS**

Enrollment Projections Report 2
Enrollment By Diagnosis, Gender, Age 2
Incident Report 2
Notification Report 3
Pending Queries Report 3
Missing Pages Report 3
Missing Investigator Signature Report 3
Site Performance Report 3

SECTION 12 **SITE TRANSFER**

Subject Site-to-Site Transfer Process 2

SECTION 12 APPENDIX

Sample Medical Records Release Form 5

PARKINSON'S PROGRESSION MARKERS INITIATIVE
Play a Part in Parkinson's Research

SECTION 1

GENERAL INFORMATION

Study Contacts

Study Personnel

Clinical Trials Coordination Center (CTCC) Office Closings (2015)

PARKINSON'S PROGRESSION MARKERS INITIATIVE
Play a Part in Parkinson's Research

CLINICAL TRIALS COORDINATION CENTER (CTCC) STUDY CONTACTS

NOTE: International callers should add (00 +1) to numbers listed below.

Whom to Call	Reason to Call
**Lead Project Manager**<br>Renee Wilson<br>Phone: 585-273-4242<br>Fax: 585-461-3554<br>Email: renee.wilson@chet.rochester.edu	Protocol or day-to-day study operational questions<br>Reportable events (Incidents and Notifications)<br>SAEs
**Project Manager**<br>Liana Baker<br>Phone : 585-276-4831<br>Fax : 585-461-3554<br>Email : liana.baker@chet.rochester.edu	Protocol or day-to-day study operational questions<br>Reportable events (Incidents and Notifications)<br>SAEs
**Project Manager**<br>Sugi Mahes<br>Phone : 585-273-2529<br>Fax : 585-461-3554<br>Email : sugi.mahes@chet.rochester.edu	Protocol or day-to-day study operational questions<br>Regulatory Questions
**Project Manager**<br>Jose Perez-Ramos<br>Phone : 585-273-4239<br>Fax : 585-461-3554<br>Email : jose.perez-ramos@chet.rochester.edu	Protocol or day-to-day study operational questions<br>Study Assessments
**Information Analyst**<br>Lisa Rumfola<br>Phone : 585-273-4126<br>Fax : 585-461-4594<br>Email : lisa.rumfola@chet.rochester.edu	CRF questions;<br>Data entry questions;<br>Data queries

Whom to Call	Reason to Call
**Clinical Monitor**<br>Saloni Sharma<br>Phone: 585-275-1574<br>Email: saloni.sharma@chet.rochester.edu	Urgent medical concerns
**eClinical System Support**<br>Phone: 585-275-3893<br>(Monday-Friday 8:30 a.m. – 9:00 p.m. ET)<br><br>Website: http://support.ctcc.rochester.edu/	Issues related to the eClinical system
**Clinical Imaging Director**<br>Nichole Daegele<br>Phone: 203-401-4367<br>Fax: 203-508-1515<br>Email: ndaegele@mnimaging.com	Questions regarding the MRI or DaTSCAN processes, procedures, transfer of scans. Questions or issues regarding ordering, shipping or receipt of DaTSCAN study drug.
**Associate Director of Research Programs**<br>Vanessa Arnedo (Michael J. Fox Foundation)<br>Phone: 212-509-0995 EXT: 287<br>Email: varnedo@michaeljfox.org	Questions regarding contracts or recruitment & retention strategies.
**Research Partnerships Officer**<br>Katie Forsberg<br>Phone : 212-509-0995 EXT: 280<br>Fax : 212-509-2390<br>Email : kforsberg@michaeljfox.org	Questions regarding site payments, document translations, Greenphire, or recruitment & retention materials.

PARKINSON'S PROGRESSION MARKERS INITIATIVE

Play a Part in Parkinson's Research

STUDY PERSONNEL

Please note the importance of consistency of study personnel. Each site has identified **one primary investigator** and **one primary coordinator** on record; however, due to the number of subjects and variety of activities involved as part of the PPMI study, we are allowing more than one investigator and coordinator to be designated. We are also allowing sites to designate additional study personnel who will be conducting neuropsychological assessments, blood draw and processing, imaging and CSF collection and processing. Therefore, it is essential to maintain consistency as much as possible throughout the study for the same investigator or sub-investigator and coordinator/co-coordinator to follow the same subject from screening through all subsequent follow up visits.

All key site personnel (e.g., investigator, sub-investigator, coordinator, co-coordinator and data entry staff) involved in the conduct of the study, including conducting assessments and/or entering data in EDC, will require a staff code assigned by the CTCC, and must be listed on the Delegation Log provided by the CTCC. Additional staff who are involved in the study (e.g., lead nuclear medicine PI, imaging tech, physician performing LP only, individual responsible for regulatory documents, etc.) do not require a staff code, however, they must be listed on the Delegation Log. The Delegation Log must be updated whenever there is a change to the study team staff. A copy of this log will be submitted to the CTCC prior to study start up and at study completion.

Notify the CTCC Project Manager in advance of the need to change investigator or coordinator. Change in Investigator or Sub-Investigator must be approved by the Steering Committee and changes in coordinator will be reviewed by the project team. Any new staff that does not currently have a staff code should complete the *New/Change Staff Form* (see Portal Administrative folder for a copy) and fax (fax number and instructions are provided on the form) or email (PPMI_CTCC_ProjectManagers@chet.rochester.edu) to the CTCC.

A PPMI *Address List* is located in the eClinical Portal Administrative folder and will be updated as changes in staffing are made. Please notify the PPMI Project Manager if there are any changes in address, email, fax, telephone numbers, or staffing.

PARKINSON'S PROGRESSION MARKERS INITIATIVE
Play a Part in Parkinson's Research

CLINICAL TRIALS COORDINATION CENTER (CTCC)

CTCC OFFICE WILL BE CLOSED ON THE FOLLOWING DAYS

Office Closings from January 1, 2015 through January 1, 2016

The CTCC will be closed on the days listed below. If you have an emergency on a day we are closed, please call 585-275-7311 and you will be instructed who to contact. Please distribute this information to the appropriate people in your office.

All day on Thursday, January 1, 2015

All day on Monday, May 25, 2015

All day on Friday, July 3, 2015

All day on Monday, September 7, 2015

12 PM EST on Wednesday, November 25, 2015

All day on Thursday and Friday, November 26-27, 2015

All day on Thursday and Friday, December 24-25, 2015

All day on Thursday and Friday, Dec 31, 2015 and Jan 1, 2016

PARKINSON'S PROGRESSION MARKERS INITIATIVE

Play a Part in Parkinson's Research

SECTION 2

PROTOCOL

(Insert a copy of the protocol and any amendments)

PARKINSON'S PROGRESSION MARKERS INITIATIVE
Play a Part in Parkinson's Research

SECTION 3

ENROLLMENT

General Information
Subject ID Assignment
CTCC Unique ID
Activities Prior to Enrollment
Protocol Deviation Codes
Electronic Enrollment Process
Subject Identification Code List

GENERAL INFORMATION

A subject will be considered to have enrolled into the study if the subject consented to participation, all eligibility criteria are met and the RANDOM page is completed in EDC. Subject withdrawals will be monitored by the study team and the need to replace subjects who withdraw will be evaluated as the study progresses. Once the projected number of subjects have enrolled into the study/cohort (see protocol for enrollment goals), sites will be notified that enrollment is closed.

SUBJECT ID ASSIGNMENT

- A series of consecutive 4 or 5 digit subject IDs will be provided to each site by the CTCC at the time a site is activated (approved to screen and enroll).
- Each potentially eligible person who signs consent will be assigned a subject ID from the list in sequential order by the site.
- The subject ID will be used throughout the study and will be included on all data forms and labs for all visits.

CTCC Unique ID

The subject will also be identified with a CTCC Unique Identification number. The CTCC Unique ID system was designed and implemented in response to the desired ability to track individual subjects across multiple studies conducted at the Clinical Trials Coordination Center without storing any personally identifiable information. The information captured is the subject's information, so make sure to document prior to completion of the subject's study visit. This number is only captured once and is not used throughout the study. It is a separate number from the assigned Subject ID described above.

NOTE: To avoid duplicate Unique ID's assigned to a subject; only collect the subject's information. Do not use information about other individuals to generate the number. If a subject at your site already has a Unique ID, there is no need to re-enter the information. Use the same number as previously generated.

Once the subject has signed the consent form, collect the following data elements about the subject that will be entered into the secure website **https://www.ctcc.rochester.edu/uniqueid/** to generate the Unique ID number:

- Last Name at birth
- First Name at birth
- Gender at birth
- Day, Month, Year of birth
- City and Country of birth

- Mother's Maiden Name

Record the number on the CTCC Unique ID page and keep a copy of the subject's CTCC Unique ID number in the site records. If at any point the subject forgets his or her CTCC Unique ID number, you or the subject can go back to the website and enter the 9 data elements again. These 9 data elements **must be identical to those entered before** (i.e., if no middle initial was used the first time data was entered, there should be no middle initial used when re-entering the data) to obtain the same unique ID number. The computer program will create the same fingerprint and return the same unique ID number each time the subject's data elements are entered.

ACTIVITIES PRIOR TO ENROLLMENT (COMPLETION OF RANDOM PAGE)

HC/PD/SWEDD/Prodromal Subjects: Before a subject is considered enrolled into the study (i.e., an eligible subject who counts towards the total enrollment goal), the site investigator and coordinator should review the Inclusion/Exclusion forms to assess that the subject meets all eligibility criteria and complete the Inclusion/Exclusion page in EDC. Any uncertainty regarding the subject's eligibility should be referred to the CTCC Project Manager prior to the subject's Baseline Visit or completing the RANDOM page in EDC.

Genetic Subjects (LRRK2/SNCA/GBA): Please refer to the Genetics Coordination Core (GCC) Operations Manual for instructions regarding genetic testing to determine subject eligibility for enrollment.

For all subjects screened by sites, the genetic mutation testing form (GMU) must be entered by the site into eClinical. The GCC will enter genetic testing results into eClinical, which will trigger arm assignment if the subject is eligible for enrollment. The site will be notified of a subject's arm assignment via email.

For subjects recruited through the Wide Spread Recruitment Initiative (WRI), please refer to the GCC Operations Manual section regarding the prescreening process through the WRI.

- **GBA Only: unaffected, LRRK2-, GBA+, ≥50 years** will be asked to complete additional testing to determine arm assignment (UPSIT and a self report questionnaire). Please refer to the GCC Operations manual for instructions regarding this specific population of subjects and requirements for enrollment.

What Information Must Be Available to Complete the RANDOM Page and Enroll a Subject?

- Required data to be entered
  - Screening/Demographics Page
  - MoCA Page
  - Inclusion/Exclusion Page

Note: If all Baseline Visit pages are not completed at the time the RANDOM page is completed, any unopened pages will appear on a site's Missing Pages report after 5 days of the Baseline Visit have passed.

- Staff Code (investigator/coordinator reporting enrollment)
- Enrollment Date (date of Baseline Visit)
- Subject's Initials
- Subject's Date of Birth
- Subject's Gender
- Date Consent Form was Signed
- Protocol Deviation Code (if applicable)
- Genetic Mutation Testing Form and Genetic Results (for Genetic subjects only)

PROTOCOL DEVIATION CODES

In the event a subject does not meet specific protocol eligibility criteria, yet the site investigator believes the subject should be allowed to enroll, the CTCC Project Manager must be contacted immediately. The Project Manager, in consultation (if necessary) with the Principal Investigator and/or Clinical Monitor, will determine if a subject will be allowed to deviate from the specified protocol criteria to enroll in the study. In this case, the Project Manager will provide the site with a **Protocol Deviation Code** to use for the subject. This unique alphanumeric code will be entered into EDC on the Inclusion/Exclusion page identifying the subject as having been allowed to enroll. The relevant eligibility statement should be answered accurately and use of the code will enable completion of the RANDOM page.

NOTE: If a Protocol Deviation Code is not provided for a given subject not meeting eligibility criteria, the subject should not be enrolled into the study. Enrollment procedures on the RANDOM page will not be accepted.

ELECTRONIC ENROLLMENT PROCESS IN eCLINICAL

Subjects will be enrolled via the web-based electronic data entry server protected by 128-bit SSL server certificates. Authenticated accounts will be created and maintained by the CTCC for each of the sites participating in the study once appropriate training has been completed by assigned site staff.

Use of the EDC system will allow **24/7** access to the module, providing additional flexibility, which may be especially useful for sites outside the Eastern Time zone as well as European sites.

NOTE: The eClinical system is down for routine maintenance every Sunday from

8 am to 12 pm (noon) Eastern Time. The eClinical system is also unavailable for a daily restart at 12 am (midnight) Eastern Time.

Site staff with user access will be directed to a page where they will enter their assigned **User ID** and **Password** in order to access the system. Once these have been validated, there will be options for entering a new subject into the system.

NOTE: A MAC computer cannot be used for this process.

In the event of system difficulties with the enrollment process, the eClinical Help Desk should be contacted (585-275-3893). NOTE: International callers should call (00 +1) 585-275-3893. Hours of operation are Monday - Friday from 8:30 a.m. – 9:00 p.m. ET.

Sample eClinical Login Page:

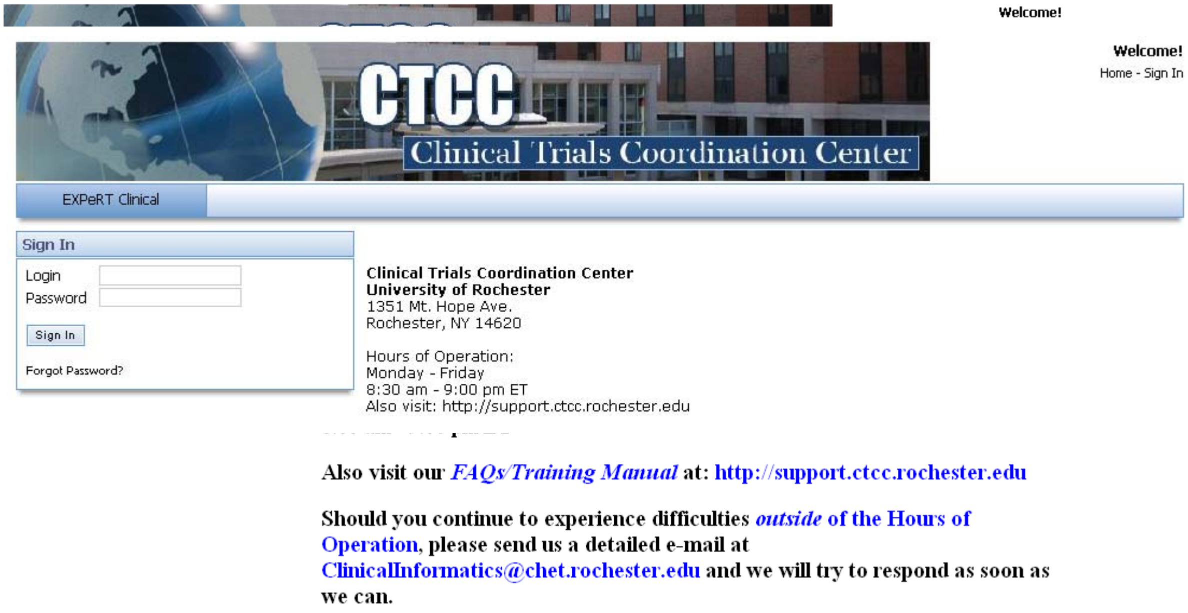

Sample EDC New Patient Page:

NOTE: 'Subject Number' is called 'Patient ID' in the system.

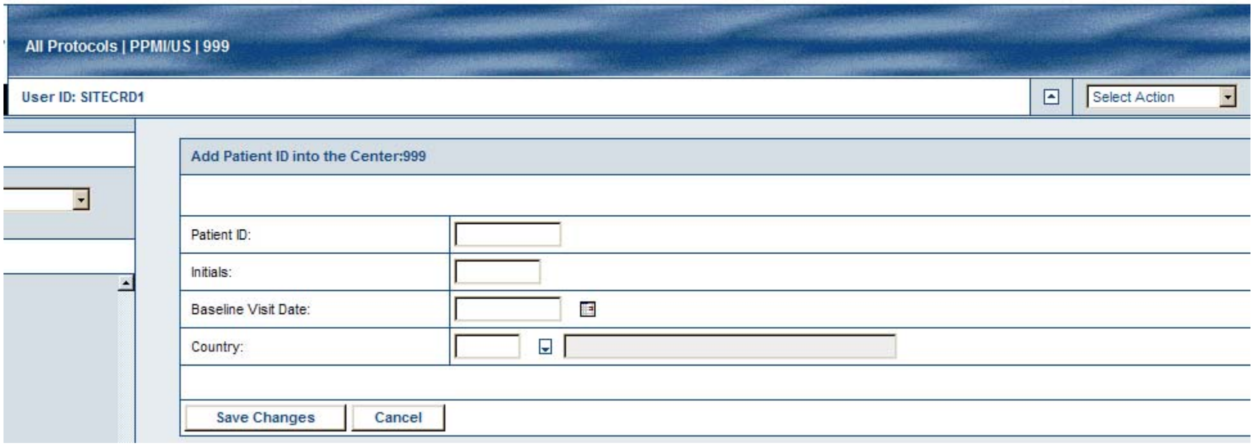

Patient ID = The 4 or 5 digit subject number assigned to the person who signed consent. This number is assigned consecutively from the block of numbers provided from the CTCC. **Please note: the block of numbers provided by the CTCC is NOT consecutive**

beyond the numbers assigned to your site. Once you reach the end of your assigned subject ID numbers, please contact the CTCC to be assigned a new block of numbers.

Initials = Insert the initials of the subject.

Baseline Visit Date = Leave this field blank. This will be auto-populated once the subject is enrolled (Random page is completed).

Country = Leave this field blank. We are not collecting Country at the present time.

Sample EDC Enrollment (Random) Page:

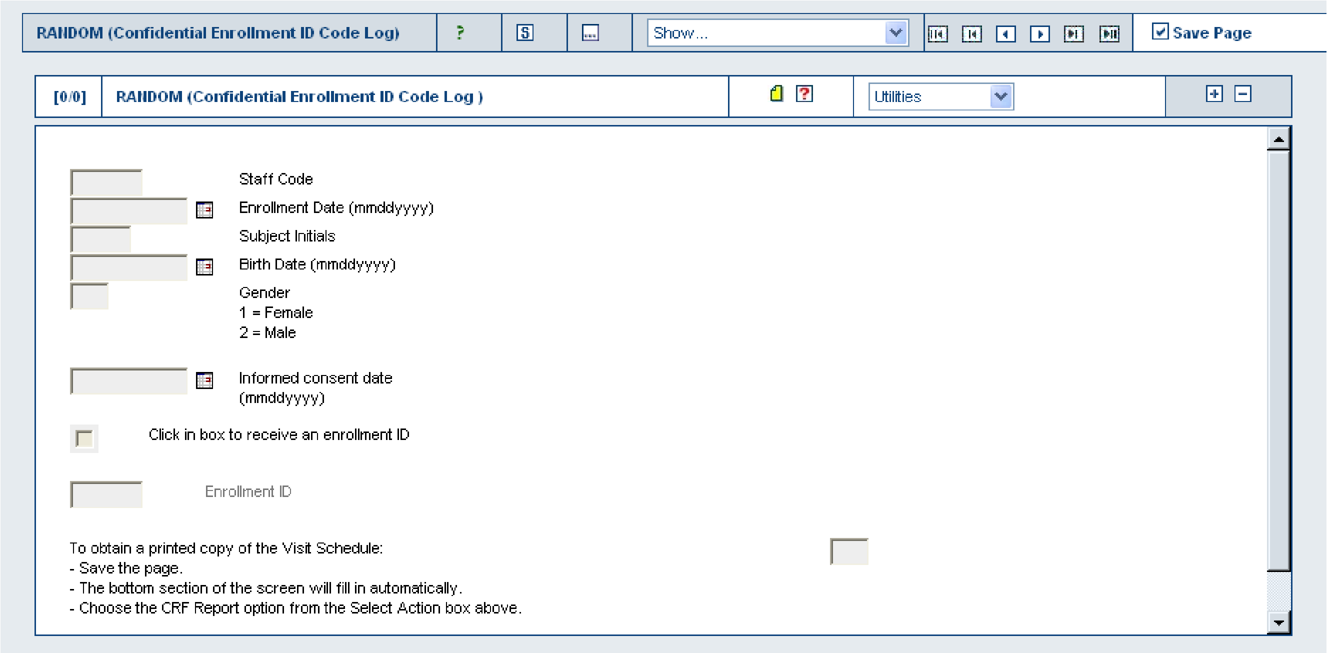

The EDC system uses the date of enrollment (Baseline Visit date) to calculate the subject's follow-up visit window schedule (the dates in between which the subject should be seen by the study staff for a given visit to be considered on time or "within window").

Enrollment Verification:

A CRF Report listing the subject's visit window schedule should be printed from the eClinical system after completing the RANDOM page (enrollment). The coordinator should file the report in the subject's study binder.

SUBJECT IDENTIFICATION CODE LIST

The Subject Identification Code List is a confidential record of all subjects enrolled into the study. This list containing individuals' names and addresses is maintained only at the site and should remain in a safe, secure location. <u>DO NOT DISTRIBUTE A COPY TO SPONSOR OR THE CTCC.</u> The original must be retained at the site and may not be destroyed until notified by sponsor or the CTCC that the document is no longer needed. A copy of this form may be obtained from the ePortal "Source Document Worksheets- 9k. PPMI Miscellaneous" folder.

SECTION 4

IMAGING

Ordering DaTSCAN™ (US Sites)
Shipment to Sites (For US Sites Only)
Ordering DaTSCAN™ (EU Sites)
Storage of DaTSCAN™
Administration of DaTSCAN™
Accountability of DaTSCAN™
DaTSCAN™ Interpretation Report

APPENDIX 4

DaTSCAN™ Drug Order Form
DaTSCAN™ Acknowledgement of Receipt Form
Investigational Medicinal Product Accountability Log
IND Visual Interpretation Report
Imaging Technical Operations Manual [insert copy]

ORDERING DaTSCAN™ (For US Sites)

DaTSCAN™ is a Schedule II controlled substance. The investigator responsible for ordering DaTSCAN™ must hold a Schedule II DEA license. ***Note: The address of the purchaser (the person holding the Schedule II license) must be the same address that the*** **DaTSCAN™** ***is shipped to.*** Also, a valid *DEA Certificate of Registration* (***DEA Form 223***) *must be maintained at the registered location in a readily retrievable manner and kept available for official inspection. If your center currently does not have a DEA Form 223, you will need to submit an Application for Registration* (***DEA Form 224***) *to the DEA. At that time, indicate that you will need 222 forms. Information about obtaining these forms can be obtained from the DEA at this website:*

http://www.deadiversion.usdoj.gov/drugreg/reg_apps/

DaTSCAN ™ can be ordered either electronically or using paper Form 222 through GE customer service. To obtain additional information about placing DaTSCAN™ ordering, go to http://us.datscan.com/imagers/ordering/ and follow the instructions provided.

Please note: To order electronically, a site must be a registered user of the DEA's CSOS (Controlled Substance Ordering System) system. To obtain a CSOS Digital Certificate, go to http://www.DEAecom.gov, click "Enroll in CSOS" and follow the instructions to enroll.

Once you have the digital certificate you will be able to access GE's online ordering system to place your order. For additional information, please contact GE customer service at DaTscanCS@ge.com .

In order to receive DaTSCAN™ using paper forms, your site will need to complete a Form 222, which can be ordered from the DEA website:

https://www.deadiversion.usdoj.gov/webforms/orderFormsRequest.jsp

The 222 forms will be mailed from the DEA within 10 working days.

DEA Form 222 Completion Instructions:

GE will requires a new 222 for each order that is sent so you will be required to send multiple completed 222s for GE to have on hand to fulfill your orders. It is suggested that the purchaser fill in a separate line for each week (9 lines should cover 60 days) even if all 9 will not be used (i.e., essentially this is a pre-order form). In the column "No. of Packages" it is suggested that the purchaser record 2 vials per week, even if only 1 vial is requested on the order form. As long as the number of vials shipped to your site is less than the number requested, it is in compliance with DEA regulations.

The purchaser must fill in the following fields on the DEA Form 222:

Field To Be Completed	Completion Instructions
TO: *(Name of Supplier)*	GE Healthcare Attn: Nuclear Licensing
STREET ADDRESS:	3350 North Ridge Avenue
CITY and STATE:	Arlington Heights, IL 60004
Date	Record Date that form is completed
No. of Packages	For Line Nos. 1-9 record "2" in each field
Size of Package	For Line Nos. 1-9 record "2.5 mL" in each field
Name of Item	For Line Nos. 1-9 record DaTSCAN
LAST LINE COMPLETED	Record "9"

Please note if a mistake is made you are not allowed to cross out and initial and date form. The DEA will accept forms with minor typographical errors. If you make any errors (other than minor typos) you must complete a new DEA Form 222.

Once the Form is completed, retain PURCHASER'S Copy 3 for your records and mail copies 1 and 2 directly to Cory Romzo at GE Healthcare (address is below). **It is important that when you tear copy 3 from the form you do not dislodge copies 1 or 2.** Your completed form will be kept on file at GE Healthcare for 60 days, after which time GE will submit a copy of the completed Form 222 to the DEA.

Attention: Cory Romzo
Nuclear Licensing
GE Healthcare
3350 North Ridge Avenue
Arlington Heights, IL 60004

Example of Completed Form 222:

See Reverse of PURCHASER'S Copy of Instructions | No order form may be issued for Schedule I and II substances unless a completed application form has been received, (21 CFR 1305.04). | OMB APPROVAL No. 1117-0010

TO: (Name of Supplier) GE HealThcare Attn: Nuclear Licensing | STREET ADDRESS 3350 North Ridge Avenue

CITY and STATE Arlington Heights, IL 60004 | DATE (current date) | TO BE FILLED IN BY SUPPLIER: SUPPLIERS DEA REGISTRATION No.

TO BE FILLED IN BY PURCHASER

Line No.	No. of Packages	Size of Package	Name of Item	National Drug Code	Packages Shipped	Date Shipped
1	2	2.5 mL	DaTSCAN			
2	2	2.5 mL	DaTSCAN			
3	2	2.5 mL	DaTSCAN			
4	2	2.5 mL	DaTSCAN			
5	2	2.5 mL	DaTSCAN			
6	2	2.5 mL	DaTSCAN			
7	2	2.5 mL	DaTSCAN			
8	2	2.5 mL	DaTSCAN			
9	2	2.5 mL	DaTSCAN			
10						

9 ◀ LAST LINE COMPLETED (MUST BE 10 OR LESS) | SIGNATURE OF PURCHASER OR ATTORNEY OR AGENT

Date Issued | DEA Registration No. | Name and Address of Registrant

Schedules

Registered as a | No. of this Order Form

DEA Form-222 (Oct. 1992)

U.S. OFFICIAL ORDER FORMS - SCHEDULES I & II
DRUG ENFORCEMENT ADMINISTRATION
SUPPLIER'S Copy 1

Requesting a Shipment of DaTSCAN™**:**
Currently DaTSCAN™ is being produced on Monday, Tuesday, Wednesday and Thursday for injection on the day following production.

Order Cut-offs:

- Orders for Tuesday injection must be submitted 5 PM eastern the previous Wednesday
- Orders for Wednesday injection must be submitted by 5 PM eastern the previous Thursday
- Orders for Thursday injection must be submitted by 5 PM eastern the previous Friday
- Orders for Friday injection must be submitted by 5 PM eastern the previous Monday

Order Cancellation Deadlines:

- Orders for Tuesday injection must be cancelled by 1 PM eastern Friday
- Orders for Wednesday injection must be cancelled by 1 PM eastern Monday
- Orders for Thursday injection must be cancelled by 1 PM eastern Tuesday

- Orders for Friday injection must be cancelled by 1 PM eastern Wednesday

To order the DaTSCAN™ you will need to complete the **[^{123}I] DaTSCAN – Ioflupane I 123 Injection Drug Order Form** (see appendix). On the order form, the "Principal Investigator" is the primary investigator listed in Box 1 of the FDA Form 1572. The phone and fax number should be indicated for the person placing the order. For each subject scheduled for imaging, you will need to request 1 vial.

Completed order forms are to be faxed or emailed as a PDF to:

Attn: Nichole Daegele
Fax: 203-508-1515
Email: datscan@indd.org

Upon receipt of the order form, IND will send an acknowledgement to your site. If you do not receive an acknowledgement, please contact Nichole Daegele at 203-401-4367 or datscan@indd.org.

Once DaTSCAN™ **is received at your site:**
For each shipment received, you will need to complete the **DaTSCAN Acknowledgment of Receipt Form** (see appendix) and record the number of packages received (vials in this case) and date received on the Copy 3 of Form 222 that you kept. Please file this completed document with your source documents.

SHIPMENT TO SITES (US sites only)

DaTSCAN™ will be delivered on the day of injection. If DaTSCAN™ is delivered to your site via FedEx, the shipment is expected to arrive by 10:30 AM. If DaTSCAN™ is delivered commercially to your site, the shipment is expected to arrive by 8:00 AM.

Please note: Each DaTSCAN™ vial will contain 5 mCi at 12:00 pm Eastern Time the day of delivery.

If a subject is scheduled for injection past 12:00 pm Eastern Time, additional vials may need to be ordered to ensure the appropriate amount of radioactivity is available at the time of the injection.

ORDERING DaTSCAN™ (For EU sites)

There is an internal system in place in the EU to accommodate a differential coding between imaging centers normal 'paid-for' vials and PPMI orders. The sites need to place their orders

with GE Healthcare using the documents provided by the Imaging Core in order to HIGHLIGHT that the vials are to be free of charge patient vials for the PPMI study. GE's customer service teams have been notified to expect and authorize free of charge vials to each of these centers.

STORAGE OF DaTSCAN™

DaTSCAN™ should be stored at room temperature and **is not to be frozen**. Appropriate radiation precautions should be observed during the preparation and storage of the agent. Aseptic technique using sterile syringes and needles should be used.

ADMINISTRATION OF DaTSCAN™

DaTSCAN™ should only be used by qualified personnel with the appropriate government authorization for the use and manipulation of radionuclides within a designated clinical setting.

DaTSCAN™ is a 5% (v/v) ethanolic solution for intravenous injection and should be used without dilution. Clinical efficacy has been demonstrated across the range 3 to 5 mCi (111 to 185 MBq). Do not exceed 5 mCi (185 MBq) and do not use when the activity is below 3 mCi (110 MBq).

To minimize the potential for pain at the injection site during administration, a slow intravenous injection (about 15 to 20 seconds) via an arm vein is recommended.

The dose should be assayed in a 10 ml syringe filled to a standard volume of 6 ml. In order to avoid geometry effects on determination of actual injected dose following injection of DaTSCAN™, the syringe should be reassayed. Subjects should be pretreated with saturated iodine solution (10 drops in water) or perchlorate (1000 mg,) prior to DaTSCAN™ injection. Subjects are to be imaged 4 +/- 0.5 hours after injection. Specifically-bound activity washes out from striatal binding sites slowly, but not negligibly, hence every effort should be made to maintain a consistent imaging time post injection of DaTSCAN™.

ACCOUNTABILITY OF DaTSCAN™

After each injection day complete the **DaTSCAN Acknowledgement of Receipt Form** and the **Investigational Medicinal Product Accountability Log** (see appendix) and file with your source documents.

Note: In the event there is a problem with the DaTSCAN™ during the study (e.g., issues with delivery or administration), the site should report the information directly to the Imaging Core. Once the issue is resolved, the Imaging Core will convey information to the CTCC as necessary. If applicable, a Notification will be generated and sent to the site for your records.

DaTSCAN™ VISUAL INTERPRETATION REPORT

The DaTSCAN completed during the screening visit is used to determine a subject's eligibility for participation. Therefore, **it is essential that screening DaTSCAN data is sent to IND within ONE day of acquisition**. IND will provide a **Visual Interpretation Report** (see appendix) within 5 business days of receipt of the data. This Visual Interpretation Report will indicate whether the subject had any evidence of a dopamine deficiency. A copy of the report should be filed with the subject's source information.

APPENDIX 4

DaTSCAN™ Drug Order Form
DaTSCAN™ Acknowledgement of Receipt Form
Investigational Medicinal Product Accountability Log
IND Visual Interpretation Report
Imaging Technical Operations Manual [insert copy]

PARKINSON'S PROGRESSION MARKERS INITIATIVE

[123I] DaTSCAN – Ioflupane I 123 Injection Drug Order Form

PPMI Study

Submit Drug Order To:	Nichole Daegele	Email Address/Fax Number:	datscan@indd.org<br>**203-508-1515**
**Note:**	**Once a screening and a date of DaTSCAN imaging is scheduled, please complete this form and fax or email a copy to the number or address listed above (a minimum of 2 days notice prior to the imaging date) and place the original on file in the Subject's CRF binder.**		

Clinical Site Name:		Site Number:	
Imaging Site Name:			
Phone Number:		Fax Number:	
Principal Investigator:			

Subject Information:

Date Subject Consented:	__ / ___ / ____ (DD MON YYYY)	Date of DaTSCAN	__ / ___ / ____ (DD MON YYYY)
Subject Number:		Subject Initials:	

DaTSCAN Delivery Contact Person(s)		Phone Numbers:	

Comments:

Site Contact Signature:		Date:	__ / ___ / ____ (DD MON YYYY)

CONFIDENTIAL
Institute for Neurodegenerative Disorders

©Copyright 2011. All Rights Reserved.

V 3.0
30/March/2015

PARKINSON'S PROGRESSION MARKERS INITIATIVE
Play a Part in Parkinson's Research

DaTSCAN Acknowledgement of Receipt

Site Number: [ ][ ][ ] Imaging Site Name: [ ]

Date DaTSCAN™ Received: [ ][ ] / [ ][ ][ ] / [ ][ ][ ][ ]
DD MON YYYY

Time DaTSCAN™ Received (24-hr format): [ ][ ] : [ ][ ] No. of vials received: [ ]

Volume/Dose(s) Received: [ ] Lot Number(s): [ ]

Any Damage to Vials? ☐ yes ☐ no

Shipment Receipt Confirmed by: [ ]

Signature of Confirmer: [ ]

Please complete form after each delivery and file with your source documents.

CONFIDENTIAL
The Institute for Neurodegenerative Disorders
©2011. All rights reserved.
V 3.0 ppmi 09/April/2015

PARKINSON'S PROGRESSION MARKERS INITIATIVE
Play a Part in Parkinson's Research

Medicinal Product Accountability Log (DaTSCAN™) PPMI

(Please retain original in Technical Binder)

DaTSCAN™ Administration/Destruction Accountability: Complete and file with your source documents after each injection day.

**Principal Investigator:**	**Pharmacist/Radiopharmacist:**

Destruction of IMP to be performed at: ☐ Radiopharmacy/Hospital ☐ Study Center ☐ Contract Vendor ☐ Returned to Clinical Supplies Unit

Note: May record up to 3 subjects per sheet

Subject Number	Date of Administration (dd/mon/yyyy)	Scheduled dose (Volume/ mL)	Batch No./ Vial Codes Used	Batch No./ Vial Codes Unused	Total Volume Administered (mL)	Estimated remaining volume in partly used vials (mL)	Signature of Person Responsible for IMP Administration	Date Vial(s) Returned if Destruction is Not at Site (dd/mon/yyyy)	Signature/Date of Person Responsible for Destruction/Decay

Comments: (Any discrepancy or deviation in drug accountability must be explained):

Adhere vial labels below:

CONFIDENTIAL **V 4.0 ppmi**

The Institute for Neurodegenerative Disorders 09/April/2015

©2011. All rights reserved.

SPECT Visual Interpretation Report
Reader 1

Study Name: ____ Protocol Number: ____

Site Number: ____ Subject Number: ____ Scan Date: ____

Imaging Center Name: ____

Clinical Site Name: ____

Scan Number: ____ ☐ N/A Radiopharmaceutical: ____ ☐ N/A

The DAT scan for the above subject was reviewed. Visual interpretation results are as follows:

☐ DAT SPECT scan ***shows*** evidence of Dopamine transporter deficit

☐ DAT SPECT scan ***does not*** show evidence of Dopamine transporter deficit

Interpretation Completed By: ____ **Date Completed:** ____

Please use this report in your assessment of the subject's eligibility for participation in the PPMI study and file this report in your study binder.

MNIID1042

CONFIDENTIAL

MNIID1042

Molecular NeuroImaging, L.L.C.

© Copyright 2011. All rights reserved.

V 4_SPECT Visual Interpretation Report_DAT 09/May/2011

PARKINSON'S PROGRESSION MARKERS INITIATIVE

Play a Part in Parkinson's Research

SECTION 5

SOURCE DOCUMENT WORKSHEETS AND eCRFs

eClinical Training Manual
Access to EDC and Portal
Electronic Signatures
Source Document Worksheets
General Directions for eCRF Completion
Directions for PPMI eCRF Pages

Version: May 2015

eClinical TRAINING MANUAL

The electronic data capture tool within the eClinical suite of applications is Data Review/Capture (EDC). A complete set of guidelines for the eClinical system including EDC basics and the query management process are detailed in the *eClinical Portal and Electronic Data Capture Training Guide.* Please refer to the *training guide* for expanded topic discussions as well as step-by-step procedures. The guide is located in the ePortal Training folder.

ACCESS TO EDC AND Portal

After successful completion of all training requirements, protocol (study) personnel will be assigned a permanent User ID and temporary password. Access to the PPMI protocol is based upon the user's role. Users may be limited to "read only" or may be given permits to enter and update data, provide resolutions to queries and apply electronic signatures.

Users are responsible for maintaining the security of their access to the applications. The eClinical application requires each user to change their password every 60 days.

NOTE: The EDC application will automatically time out after 30 minutes of inactivity. *Data must be saved as each eCRF page is entered.* You will be prompted to save the page if you attempt to move to the next page without saving data. However, if the system times out, unsaved data is lost.

ELECTRONIC SIGNATURES

The definition of an electronic signature is "a computer data compilation of any symbol or series of symbols executed, adopted or authorized by an individual's handwritten signature". The FDA and Clinical Trials Coordination Center consider electronic signatures to be the equivalent of handwritten signatures. Please refer to the eClinical Portal and Electronic Data Capture Training Guide for directions to apply electronic signatures.

An electronic signature (eSignature) is required by the Site Investigator or delegated Sub-Investigator on the following eCRFs:

- Signature Form
- Adverse Event Log

SOURCE DOCUMENT WORKSHEETS

At study start up, sites will be supplied with a supply of purchased assessments needed for the Screening and Baseline visits for a set number of participants. Sites are responsible for printing the source document worksheets (SDWs) from the ePortal "Source Document Worksheet" folder. The folders are organized by cohort and within each folder, by visit. Each visit includes a CRF checklist to assist with visit/CRF planning. Please be mindful of the reminders included in each CRF "bundle" as they contain important information regarding different scenarios in which a CRF would or would not be expected.

NOTE: Prior to the first study visit, print off a blank set of the source document pages from the ePortal "Source Document Worksheets" folder and keep the blank set in your study binder as back-up. Should you experience any issues with accessing the internet to enter data or download source worksheets, you will have a blank set to copy and use as needed.

GENERAL DIRECTIONS FOR eCRF COMPLETION

An eCRF is an auditable electronic record designed to record data required by the clinical trial protocol. eCRFs may have special display elements, electronic edit checks, and other properties or functions and are used for both capture and display of the data.

Sites will be provided source document worksheets to capture data onto the eCRF page, while other eCRF pages are completed from separate source notes. See Section 7 "Source Documentation" for details. Data should be entered within 2 days of a completed visit. Once the RANDOM event is completed and the subject is enrolled, any missing pages (from Screening and Baseline) will appear on the Missing Pages report for data not entered within 5 days of the baseline visit/enrollment data. For subsequent visits, missing pages will also be generated on pages not entered within 5 days of the target visit date (based on the subject's Visit Window Schedule). Once data is entered and saved, it is securely transmitted to the CTCC central database and stored on a secure server.

Range and edit checks run concurrently with data entry. Sites should develop a practice of immediately checking the Query Management Module to review and resolve any queries that may have been generated from these checks. The Information Analyst (IA) will also routinely run consistency checks, which review the data for consistency within a page and across multiple pages and/or multiple events. The IA will also run automated programs to code Current Medical Conditions, Concomitant Medications and Adverse Events.

REMEMBER: Queries are generated as soon as a page is opened and saved. If an assessment is not complete or information is pending, it is advised that you open the page and enter the data once the information is complete.

Sites are responsible for logging into EDC (at least weekly) to review and resolve additional queries that may have been generated or applied manually. Sites may also reference the Pending Queries Report to determine what requires resolution in the database.

When data for all pages in an event are "clean" (no queries pending resolution and no missing pages), the event can be *Secured*. Pages in a secured status can be viewed by all, but no updates or changes to data can be made by the site users. Should changes to data be required, the site staff must contact the CTCC Information Analyst and request that a page or pages be un-secured.

The EDC system audit trail tracks all the required elements:

- Initial entry of data: by whom and when;
- Updates to data: by whom, when, the old and new values, and why the change was made to the data.

Abbreviations

Sites are discouraged from abbreviating, but abbreviation is acceptable when the meaning is unambiguous.

Misspellings

Misspellings by site staff are corrected in the database by CTCC staff as long as there is no ambiguity in the meaning. If the meaning is unclear, the site is queried for clarification.

Numeric Data

All numbers will be auto justified and do not require any leading zero fills. If a response requires a decimal point, the decimal point must be entered into one of the answer boxes.

Dates

Dates are recorded in the MMDDYYYY format. If an exact date is not known, you will be prompted to identify which part of the date is estimated.

GENERAL DIRECTIONS FOR eCRF COMPLETION (Continued)

Time
Time is recorded in the hours:minutes (HH:MM) format using military time. For example: 13:31 (1:31 pm). The EDC application requires entry of the colon.

Data Entry Errors
Errors due to incorrect data entry ('keypunch error') are corrected in the database and assigned 'site entry error' as reason for change.

Corrections or Updates to Saved Data
If a change is made to an eCRF after it is saved in the database, the correction will require the Site Coordinator or Site Investigator to indicate the reason for change within the audit dialog box. If a change was made to a page that required an eSignature, the eSignature will need to be reapplied. If a correction is necessary after the data have been *secured*, the CTCC IA must be contacted. All changes are documented via the audit trail.

Missing Data
Unexpected blanks are queried unless accompanied by a note to explain the reason for leaving the item blank (see the training guide for instructions on entering notes).

Missing Assessments/Pages
If part of an assessment is not performed, an explanatory note should be added to the respective data field(s). If the entire assessment is missed, do not open and/or save the page (this could generate unnecessary queries). A comment should be added to the Signature Page to document why the assessment was missed or not done. However, until the Signature Page is completed, any missing pages will appear on the Missing Pages report after 5 days of the expected Target Visit date for that subject.

REMEMBER: Missing pages will appear on the Missing Pages report and will be included in site metrics regarding data entry compliance once the RANDOM event is completed.

Missed Visits
If a visit is not performed, it should be noted on the visit Signature page. **No other eCRFs from the visit should be opened.** If the final visit is missed, the site must complete the Signature page, Conclusion of Study Participation page and all logs.

eCRFs for Premature Withdrawal of Subjects

When a subject withdraws from the study, evaluation activities listed for the premature withdrawal visit should be completed. Sites should pull in the PW event (see the training guide for instructions on pulling in an unexpected event) and refer to the PPMI schedule of activities to determine which assessments and eCRFs should be completed.

Unscheduled Visits/Unscheduled Phone Visits

If a subject calls to report an adverse event, the site investigator must determine whether a clinical visit is required. If a visit is deemed necessary and it falls **within the window** for the next regular follow-up visit, proceed with the visit as a standard follow-up visit including the completion of all tests and case report forms corresponding to the visit number per the protocol schedule of activities. If it is determined the subject will be withdrawn from the study, ensure that activities specific to the final (premature withdrawal) visit are completed as well.

If the unplanned visit falls **outside of the window** for the next regular follow-up visit, the visit is considered an unscheduled visit. The site should pull in (add) the U0x event (see the training guide for instructions on pulling in an unexpected event) and refer to the schedule of activities to determine which assessments and eCRFs should be completed for an unscheduled visit.

Evaluation Dates

Visit date refers to the actual date that visit activities were conducted. Typically, the date is consistent on all eCRFs for the visit. If, for any reason, a visit stretches across several days, record the actual date the given task was performed (e.g., consent is signed and clinical assessments are done on Day 1 and DAT scan is done on Day 2 of the Screening visit) and indicate the latest date that activities were completed as the visit date on the Signature Page. The note function may also be used in the system to highlight different dates of evaluation.

Subject Identification Number (Subject ID)

All subjects who are potentially eligible and who have signed an informed consent should be assigned a 4-digit or 5-digit Subject Identification Number (provided by CTCC to sites). Subject ID Numbers should be assigned consecutively, beginning with the first number in the Subject ID Number list provided to each site.

NOTE: The EDC system refers to the Subject Number as Patient ID.

REMEMBER: Once data entry for a page (or visit) is complete, you must check the "Entry Complete" status box. This process has been implemented to identify that data is complete and ready for transfer to LONI, the core facility that combines all clinical, imaging and biologic data for the PPMI study.

DIRECTIONS FOR PPMI eCRF PAGES

For the frequency of administration, please refer to Study of Activities (SOA) relevant for given sub-group.

SCREENING/DEMOGRAPHICS FORM

- Data will be captured about any subject who has signed consent
- Reflects site predictions about the number and timing of future enrollments (see Projected Enrollment Date below)
- Information provided is also used to describe recruitment efforts in reports to the sponsor and IRB annual reports
- **Projected Enrollment Date** - Please be sure to indicate a projected enrollment date (i.e., date of Baseline Visit). If this is unknown at the time of the Screening visit or data entry, it is acceptable to estimate; however, please update the page once the visit is confirmed. This date is used for reporting on the Enrollment Projections Report (see Section 11 of this manual).

NOTE: The web-based *Screening/Demographics Form* must be updated regularly to document status of potentially eligible subjects. For example, when the subject signs a consent form complete the form. If a subject decides to decline participation in the study or is excluded prior to enrollment, the form must be updated in the system.

INCLUSION/EXCLUSION

- Ensure the correct form is used based on the subject group and arm assignment
- Question relating to imaging results are based on the information obtained from the Visual Interpretation Report forwarded from IND (Imaging Core)
- This page must be completed in order to enroll a subject (complete the RANDOM page).

ADVANCE DIRECTIVE/REVIEW OF CONTINUING ABILITY TO CONSENT

- Completed for all subjects in all cohorts to name a substitute decision maker to make clinical research decisions for PPMI if the subject becomes unable to make decisions for themselves during participation in the study.

- The Advance Directive for Clinical Research Participation form should be completed by the subject. This form allows the subject to name an individual as a substitute decision maker and indicate if they would like to continue in PPMI if they should lose their ability to make clinical research decisions.

- The Research Advance Directive will be completed to indicate the status of the document from visit-to-visit.

FOUND- CONSENT TO SHARE CONTACT INFORMATION

- Conduct once for all subjects who agree to share contact information with UCSF for the FOUND sub-study.
- Continue to ask at every 12 months for all subjects enrolled in PPMI who previously declined or delayed their decision when previously asked. Complete form and send to UCSF using a secure electronic or faxed contact form.

PD FEATURES

- Completed by PD subjects only (in any sub-groups) to capture symptom onset and date of diagnosis
- Question 3 – capture the symptoms that were present at the time of PD diagnosis.

FAMILY HISTORY OF PD (ORIGINAL PD, HC, SWEDD AND PRODROMAL)

- Completed only at Screening.
- If response is "None" then a zero (0) should be used.
- Note that Control participants should have no first degree relative with PD indicated (i.e., response to question 1, 2 or 3).

FAMILY HISTORY OF PD LOG (GENETIC COHORT AND REGISTRY)

- Completed at every in-person visits including Screening, Baseline, ST and Premature Withdrawal Visit.
- If response is "None" then a zero (0) should be used.
- If the information changes at subsequent visit, the new information will replace the previous data in eClinical.

PRIMARY DIAGNOSIS (ORIGINAL PD, HC AND SWEDD CONTINUING AFTER V06)

- Form is to be completed by the Investigator.
- Included for PD, Control and SWEDD subjects continuing after V06 to capture any change in diagnosis that may occur over the period of the study.

- In order to be eligible for participation, PD subject response to Q2 should be "01 = Idiopathic PD" and Control subject response to Q2 should be "17 = No PD nor other neurological disorder".

DIAGNOSTIC FEATURES OF PD

- Form is to be completed by the Investigator for all subjects enrolled.
- Used to determine phenoconversion based on the "Phenoconversion Guidelines Document". This document is included in Appendix B of Section 6 of the General Operations Manual (Assessments) and is also posted as a stand-alone document in the eClincial portal in the General Operations Manual folder.

DIAGNOSTIC QUESTIONNAIRE (PRODROMAL, GENETIC COHORT AND REGISTRY)

- Form is to be completed by the Investigator.
- For prodromal (hyposmic or RBD), response to Q1 should be "23 = Prodromal non-motor PD (at least one non-motor symptom and no motor symptoms)" until such time as PD or some other listed diagnosis is determined.

PREGNANCY FORM

- This form will only be populated (and completed) for a subject indicated with gender = 0 (Female of childbearing potential) on the Screening/Demographics page (except for Genetic Registry subjects).
- Urine pregnancy test must be completed and result confirmed prior to injection of DaTSCAN™ or ^{18}F-AV-133 or [^{18}F] florbetaben

USE OF PD MEDICATION

- Completed for PD, Prodromal, SWEDD and Genetic Cohort PD subjects at each visit after Baseline.
- Used to track whether a subject has started medication for treating symptoms of PD.
- Used to track whether a repeat motor exam is expected for a subject (i.e., if levodopa or dopamine agonist was started).

MDS-UPDRS (POST DOSE)

- Part III Motor Exam (Post Dose) is expected at an annual visit for any PD subject (except Genetic Registry PD) who has started levodopa or dopamine agonist (i.e., the Motor

Exam is conducted twice at the visit; once before PD medication dosing and again one hour after dosing of PD medication in clinic).

- Part III Motor Exam (Post Dose) should be completed for all subjects "ON" PD medication at the time of the study visit (e.g., PD medication was taken in the morning prior to the study visit).

SYMBOL DIGIT MODALITIES TEST

- The worksheet is considered source, so the total correct response can be entered directly into the database from the worksheet.
- The key to this is posted on the eClinical Portal Source Document Worksheets folder.

SIGNATURE FORM (ORIGINAL PD, HC, SWEDD AND PRODROMAL)

- A signature form is *required* for each expected study visit whether or not the visit was actually performed. If a subject misses a visit, the Signature Form for that visit still needs to be completed.
- Site payment for each visit is processed based on completed data entry and completed signature forms (eSignature is applied by the Investigator).
- If an expected visit (e.g., Visit 01) did NOT occur, complete ONLY the signature page for that missed visit as follows:
  - Enter the target date that visit should have occurred (refer to Enrollment Verification visit schedule for that subject).
  - Question 1.1 – answer as #3 (NOT DONE).
  - Question 1.2 – indicate reason why visit was missed.
  - Question 1.3 – since visit was not done, indicate response of 0 = No.
- This form certifies that the investigator (or designated sub-investigator) have reviewed all data points for the visit and determined that they are complete, accurate and compatible with source documents.
- ***This form must be signed AFTER all data for the visit have been reviewed and entered.***

INVESTIGATOR SIGNATURE (GENETIC COHORT AND GENETIC REGISTRY)

- A Investigator signature form is *required* for each expected study visit whether or not the visit was actually performed. If a subject misses a visit, the Signature Form for that visit still needs to be completed.
- Site payment for each visit is processed based on completed data entry and completed Investigator signature forms (eSignature is applied by the Investigator).

- This form certifies that the investigator (or designated sub-investigator) have reviewed all data points for the visit and determined that they are complete, accurate and compatible with source documents.
- ***<u>This form must be signed AFTER all data for the visit have been reviewed and entered.</u>***
- The visit status is updated in a separate form ("Visit Status" form) for Genetic Cohort and Genetic Registry participants.

VISIT STATUS FORM (GENETIC COHORT AND GENETIC REGISTRY)

- A Visit Status form is *<u>required</u>* for each expected study visit whether or not the visit was actually performed. If a subject misses a visit, the Visit Status Form for that visit still needs to be completed.
- If an expected visit (e.g., Visit 01) did NOT occur, complete ONLY the Visit Status form for that missed visit as follows:
  - Enter the target date that visit should have occurred (refer to Enrollment Verification visit schedule for that subject).
  - Question 1 – answer as #0 (NO).
  - Question 3 – indicate reason why visit was missed.
  - Question 4 – since visit was not done, indicate response of 0 = No.

CONCOMITANT MEDICATION LOG

- Record medications the subject is taking at the time of the Screening Visit.
- At subsequent visits record new meds, and changes/discontinuation of previously listed medications.
- <u>Medication</u>: Whenever possible, use generic name. If generic name cannot be determined, use brand name.
- <u>Dose</u>: The total dose the participant takes for one administration. Enter only the numerical portion. For example, 2 tablets of 300 mg each should be entered as TOTAL of 600 mg.
- <u>Units</u>: The unit of measurement by which the drug is being measured (e.g. mg, IU, tabs).
- <u>Frequency</u>: How often the drug is being given (e.g. QD, BID, QID).
- <u>Route</u>: Record route of administration using key options 1-9.
- <u>Start/Stop Dates</u>: The start date of medication should be the date the participant first began taking the medication. Enter in MMDDYYYY format; if the exact date is unknown, an **estimated date** should be used.

- Date estimation key: Record date estimation using key options 1-4 following start and stop dates.
- Ongoing: If medication is continuing, leave blank until the study is over or subject ends participation. If a medication has stopped, answer should be 0 (No) and a valid stop date must be provided.
- Indication: Purpose for which the medication is taken (e.g. hypercholesterolemia, general health). If taken for a medical condition, the indication should match the name of the event on the Current Medical Conditions Log.
- PD Medication: If medication is a PD med then response of 1=Yes should be used.

CURRENT MEDICAL CONDITIONS LOG

- Purpose of this form (different from the AE Log) is to capture conditions that have been diagnosed and are current at the time of a visit, essentially a "snapshot" of any active or chronic medical conditions that could potentially impact study analyses.
- Not necessary to capture previous ailments that are resolved.
- Capture the actual diagnosis, not the symptoms.
- For PD subjects, we do not expect to see Parkinson disease listed, this is understood and a requirement of eligibility.
- Symptoms: Do not enter symptoms, such as hip or back pain. These are not actual diagnoses so they would not be expected. If a diagnosis has not yet been made for one or more symptoms present at the visit, nothing is to be recorded regarding those symptoms.
- If the pain is related to arthritis then the term we would expect is "arthritis".
- **Procedures:** Do not enter procedures. For example, pacemaker implant or cataract removal.
- **Resolved Conditions:** Do not list conditions that started and resolved in between study visits. If a medical condition is listed, we would expect the subject to have a current/active diagnosis. For example, if "Appendicitis" is entered on this log we would expect the subject to have entered the clinic that day with appendicitis. Another example, if "cardiac arrest" is entered in the log this would be interpreted as the subject having experienced cardiac arrest while in the clinic for the visit. Please be sure to list only active diagnosed conditions.
- **Chronic Conditions:** There are times when active diagnosed conditions can have flare-ups. To capture these events we would expect the Current Medical Conditions Log to include "worsening of __________" entered on a new line. An example of this would be if a subject at baseline had been diagnosed with lupus. At visit 1 the subject still had lupus but also at that time had a flare-up of lupus. A new row on the log would read

"Worsening of Lupus." Then, record the end date for "Worsening of Lupus" at the next visit if the flare-up resolved prior to that visit.

ADVERSE EVENT LOG

- Used to record adverse events that may be reported within 7 days of a DAT scan or lumbar puncture or skin biopsy (selected sites) or Florbetaben imaging (selected sites).
- Used to record adverse events that may be reported within 48 hours (+/-24hrs) of a AV-133 PET Imaging (Australia and selected sites)
- Record these adverse events whether or not they are thought to be related to the DaTSCAN, LP, AV-133, Skin biopsy, Florbetaben or other.

GENETIC COUNSELING LOG

- Used to record date of counseling sessions for genetic cohort and registry subjects when counselling takes place.
- If counseling is provided by the GCC, site is responsible for documenting this in the Genetic Counseling Log (response 2=GCC should be noted in these cases). Date GCC provided counseling can be obtained from documents forwarded by the GCC for subjects recruited through the WRI.

CONCLUSION OF STUDY PARTICIPATION

- Used for subjects who withdraw participation before the study is completed, as well as for subjects who complete the study.

PARKINSON'S PROGRESSION MARKERS INITIATIVE

Play a Part in Parkinson's Research

SECTION 6

ASSESSMENTS

Order of Assessments
Schedule of Alternate Forms
General Testing Guidelines
Equipment and Supplies
Diagnostic Questionnaire
Diagnostic Features
MDS-UPDRS with Hoehn & Yahr
Modified Schwab & England Activities of Daily Living
Physical Activity Scale for the Elderly (PASE)
Hopkins Verbal Learning Test – Revised (HVLT-R)
Benton Judgment of Line Orientation
Semantic Fluency
Letter Number Sequencing (LNS)
Symbol Digit Modalities Test
Montreal Cognitive Assessment (MoCA)
Epworth Sleepiness Scale (ESS)
REM Sleep Behavior Disorder Questionnaire
Geriatric Depression Scale (GDS-15)
State-Trait Anxiety Inventory for Adults
Questionnaire for Impulsive-Compulsive Disorders (QUIP)
Scales for Outcome in Parkinson's Disease (SCOPA-AUT)
Cognitive Categorization
University of Pennsylvania Smell Identification Test (UPSIT)

Order of Assessments

The following recommended order of conducting the cognitive and behavioral assessments is listed below.

- HVLT Learning Trials (i.e., Trials 1-3)
- Benton Line Orientation
- Semantic Fluency
  - Animal Fluency
  - Vegetable Fluency
  - Fruits Fluency
- Letter Number Sequencing Memory Test
- Symbol-Digit
- HVLT Delayed Recall (i.e. Trial 4)
- HVLT Delayed Recognition
- MoCA

Schedule of Alternate Forms

There are alternate versions for several of the tasks (HVLT-R, Symbol Digit Modalities, and Benton Judgment of Line Orientation); forms will alternate between visits in order to minimize practice effects. Please refer to the following chart when preparing for your visit:

Assessment	BL	MTH 12/ V04	MTH 24/ V06	MTH 36/ V08	MTH 48/ V10	MTH 60/ V12
Symbol Digit Modalities Test	Form 1	Form 2	Form 1	Form 2	Form 1	Form 2
Hopkins Verbal Learning Test-Revised	Form 1	Form 2	Form 3	Form 4	Form 5	Form 6
Benton Judgment of Line Orientation	Form H (Odd)	Form H (Even)	Form H (Odd)	Form H (Even)	Form H (Odd)	Form H (Even)

For ST Visit replacing an annual visit, please use the form that was designated for that annual visit. If Premature Withdrawal Visit occurs at a visit that requires these assessments, please use the form that was designated for that visit. If ST or Premature Withdrawal Visit occurs outside of the visit window for an annual visit, please use the assessment that is available and not recently used.

General Testing Guidelines

1. Practice administering the tests (e.g., with co-workers) prior to testing a real subject. Become fully familiar with the test instructions (try to memorize them), and always have the test administration instructions with you and open at the relevant page during testing.

2. Conduct testing in a quiet room free from distractions or potential interruptions. The room should be physically comfortable (appropriate temperature and ventilation), and testing should be conducted at a table with examiner and examinee seated on opposite sides. Good lighting is essential.

3. Remind subjects to bring eyeglasses and hearing aids if needed. If the subject forgets to bring these aides, note this in the subject's source documentation. Depending on the severity of their sensory limitations, rescheduling may be necessary.

4. Have the necessary testing materials arranged in advance (e.g., stopwatches, pencils, test stimuli and forms, etc.). Consider keeping tests out of the subject's sight until you are ready to use them to avoid distraction. Whenever possible, score responses unobtrusively to lessen examinee anxiety about how they are doing.

5. Rapport is important, since subject good-will and effort are key to validity. Adherence to standardized procedures does not mean that you must test in an unnatural or mechanical manner. Use a natural conversational tone, encourage interest in the activity, and reinforce good effort.

   Providing a general idea of the reason for the testing, and how long it will take, often helps put subjects at ease. Stress the importance of good effort, and reassure examinees that all subjects find testing quite difficult at times – it is just part of the process. (Sometimes it helps to let subjects know that if the tests were easy they would not provide useful information; the important thing is that they just keep doing their best.)

6. Examinees should understand what a specific test requires them to do **before you start the actual test**. The standard administration instructions and practice items are designed to achieve this. However, you may occasionally need to supplement or reword these instructions to ensure the subject fully understands the test. If you have any doubt, re-instruct before beginning.

7. During testing, be attentive to potential sources of distraction (e.g., extraneous noises, interruptions by others, etc.). In memory tests you usually cannot repeat the material (e.g. words, numbers or letters to be remembered) during a trial. Do your best to ensure that extraneous distractions do not occur (e.g., turn off phones and put a "do not disturb"

notice on the door). When reading stimuli, make sure that you do so loudly and clearly from the outset.

If, in extreme circumstances, a specific trial is unambiguously undermined by an external event (e.g., your reading of a letter-number sequence is suddenly made inaudible by a jack-hammer), it *may* be permissible to repeat the sequence. **This must truly be the exception rather than the rule, and should rarely if ever happen. When it does you must note this on the CRF. Do not continue with the testing until you have made certain that the distraction will not reoccur.**

In contrast, you should never repeat a letter-number sequence, or words during the Hopkins memory test, simply because a subject asks you to. Do not begin a trial if it appears that the subject is momentarily not paying attention; however, once you begin, their loss of attention is part and parcel of what you are measuring.

When subjects ask for repetition of a letter-number sequence, or words on the Hopkins list, state that you cannot repeat them and ask them to "just do the best you can".

8. Feedback to subjects during testing is a somewhat complicated issue. The rule of thumb is that examiners should be "neutrally supportive", i.e., that you should reinforce good effort, reassure subjects ("you're doing fine"), and so forth, but that you should not provide specific feedback (such as whether an examinee got a particular item correct or not).

   In some circumstances additional clinical judgment may be called for. A short break is usually appropriate when an examinee exhibits real distress over their performance, with brief counseling or support as needed. As always, this should be reported on the CRF.

   Some examinees view the standard "neutral support" responses of the examiner as disingenuous or patronizing; in these more challenging situations, the examiner should adapt appropriately and maintain rapport as best they can.

   In yet other circumstances, the examiner may feel that the subject is not exerting reasonable effort. It is sometimes appropriate to address this issue in a direct but non-threatening manner, e.g. explaining your concern, reiterating the importance of the data to the research, and requesting a commitment to real effort. Any concerns about effort should be noted on the CRF. Be careful not to leap to any conclusions about effort with subjects, since what can appear to be insufficient effort may actually reflect inability. In contrast, the occasional healthy control may be "just going through the motions" during testing, and may need diplomatic cajoling regarding effort. Appropriate respect for the

testing process and for full engagement begins with the examiner, who should always convey a professional attitude towards the process and the data being generated.

9. In general, the examiner should display a friendly but business-like approach to testing. If a subject continually disrupts testing or is excessively digressive, the examiner should reorient the subject back to the task at hand to retain fidelity to time limits and the need for a reasonably standardized administration. No one approach will work across all challenges encountered by examiners; flexibility, and a professional commitment to the generation of valid data, are fundamental to successful testing. Learn as much as you can about the point of specific tests; understanding exactly what it is that the test is intended to measure will help you make good decisions when unexpected events occur.

10. Subjects should be encouraged to provide responses even when they are unsure. If they give more than one answer where just one is required, have them choose one (e.g., "which is the best answer"? or "choose one").

11. Verbatim recording and detailed notes are useful. Consider audio taping responses (with appropriate consent) for tests where you may not be able to fully capture the subject's responses and wish to check later (e.g., on the fluency tests or Hopkins word recall).

12. Deviations from the standard protocol by either examiner or examinee should be noted in detail on the CRF immediately upon completion of the testing.

Equipment and Supplies

You will need a stopwatch, pencils, and a quiet comfortable room with a table and chairs. Copies of all test answer forms, the HVLT-R booklets, and the Benton Judgment of Line Orientation booklets are provided by the CTCC. Re-supply will be provided as applicable.

If you are in need of additional PPMI assessments please complete the "PPMI Assessment Order Form" located in Appendix 6 and send via email to ppmi_ctcc_projectmanagers@chet.rochester.edu.

Diagnostic Features of PD

The Diagnostic Features assessment is used for all subjects and allows Investigators to provide a detailed assessment of clinical signs and symptoms. This assessment is used to determine phenoconversion. Please refer to the "Phenoconversion Guidelines Document" in Appendix 6. This document is also posted separately in the eClinical Portal in the General Operations manual folder.

MDS-UPDRS

The full 30 page MDS-UPDRS, including Hoehn and Yahr Stage, and Score Sheet has been provided to sites for use as source documentation for this assessment. Instructions for administration are contained within the MDS-UPDRS packet. In addition, Investigators and Sub-investigators must receive training and certification through the MDS website prior to administration. Coordinators are not permitted to administer the MDS-UPDRS.

Part Ia – Assessed by site Investigator or Sub-investigator (coordinator may conduct as approved by CTCC).

Part I (Patient Questionnaire) – Completed by the subject and/or caregiver.

Part II (Patient Questionnaire) – Completed by the subject and/or caregiver.

Part III – Assessed by site Investigator or Sub-investigator.

- Note: Questions 3a and 3c will not be captured in the database. See Use of PD Medication data form to capture and record information for subjects on medication for treating symptoms of PD.

Part IV – Assessed by site Investigator or Sub-investigator.

Hoehn and Yahr Stage – Assessed by site Investigator or Sub-investigator.

MDS-UPDRS (Post-Dose)
PD subjects who have started levodopa or dopamine agonist will have an annual assessment of MDS-UPDRS Part III and Hoehn and Yahr stage in a "practically defined off" state. **Note: the post dose assessment is only required at annual visits.** The repeat post-dose motor exam is NOT conducted in subjects who have started other types of PD medications (e.g., amantadine, rasagiline (Azilect®), selegiline (Eldepryl®, Zelapar®).

- Definition of practically defined off = Subject has not taken PD meds since last regularly scheduled dose the night before the visit (at least 12 hours before evaluation).

Steps for Assessing:
1. Subject should be reminded to hold medication for the in-person visit when this will be conducted.
2. Conduct full MDS-UPDRS.
3. Give subject appropriate PD medication dose.
4. <u>One hour</u> after the subject receives PD medication, repeat Part III (Motor Exam) and Hoehn and Yahr Stage. Complete the *MDS-UPDRS Post Dose* source worksheet.

Modified Schwab & England Activities of Daily Living

This 0-100 scale is used to rate a subject's current overall function. Scores should be in increments of 5 points (e.g., 100, 95, 90, 85, etc.) based on a consensus rating of the investigator, subject and/or caregiver.

100% Completely independent. Able to do all chores without slowness, difficulty or impairment. Essentially normal. Unaware of any difficulty.

90% Completely independent. Able to do all chores with some degree of slowness, difficulty and impairment. Might take twice as long. Beginning to be aware of difficulty.

80% Completely independent in most chores. Takes twice as long. Conscious of difficulty and slowness.

70% Not completely independent. More difficulty with some chores. Three to four times as long in some. Must spend a large part of the day with chores.

60% Some dependency. Can do most chores, but exceedingly slowly and with much effort. Errors; some impossible.

50% More dependent. Help with half, slower, etc. Difficulty with everything.

40% Very dependent. Can assist with all chores but few alone.

30% With effort, now and then does a few chores alone or begins alone. Much help needed.

20% Nothing alone. Can be a slight help with some chores. Severe invalid.

10% Totally dependent, helpless. Complete invalid.

0% Vegetative functions such as swallowing, bladder, and bowel functions are not functioning. Bedridden.

Consensus rating
(Investigator, patient, other sources) 1. [ ][ ][ ]

Physical Activity Scale for the Elderly (PASE)

The PASE assessment is a subject completed questionnaire to evaluate physical activity levels in the PD subjects as compared to the Controls, SWEDD, Prodromal and Genetic Cohort subjects. There are 10 parts to this questionnaire asking the subject questions related to leisure time activity; household activity; and work-related activity. Subjects are asked to complete the questionnaire by either circling the correct response or filling in the blank as applicable.

Hopkins Verbal Learning Test-Revised

There are 6 different forms used throughout the study.
Make sure to use the correct form for each subject at the visit.

Form 1 will be used for the Baseline visit and Forms 2-6 will be used for the annual follow up visits. Detailed instructions for administration are in the booklet. Page 2 contains instructions for Trials 1 – 4 and page 4 for trial 5. See summarized instructions below:

Immediate Recall Administration Instructions

Trial 1

Say the following:

I am going to read a list of words to you. Listen carefully, because when I'm through, I'd like you to tell me as many of the words as you can remember. You can tell them to me in any order. Are you ready?

Repeat or paraphrase the instructions if necessary.

Read the words at the rate of approximately one word every 2 seconds.

If the individual does not spontaneously begin reporting words after the last word is read, say the following:

OK. Now tell me as many of those words as you can remember.

Record the responses verbatim (including repetitions and intrusions) in the Trial 1 column of the HVLT-R test booklet. When the individual indicates no more words can be recalled, proceed to Trial 2.

Trial 2

Say the following:

Now we are going to try it again. I am going to read the same list of words to you. Listen carefully, and tell me as many of the words as you can remember, in any order, including all the words you told me the first time.

Use the same procedure as in Trial 1 to record the responses in the column for Trial 2. Then proceed to Trial 3.

Trial 3

Say the following:

I am going to read the list one more time. As before, I'd like you to tell me as many of the words as you can remember, in any order, including all the words you've already told me.

Record the responses in the column for Trial 3 using the same procedure as in the previous trials.

Record the Completion Time and make note of when the delay will end (20-25 minutes after the end of Trial 3.)

Remember: Do not inform the subject about the delay or
that they will be tested again on these words!

Delayed Recall Administration Instructions (Trial 4)

Trial 4 should be administered after Symbol Digit Modalities Test and it can be administered irrespective of how much time has passed since the completion of the intervening tests are constant and provide 'interference'.

After the 20-25 minute delay, say the following:

Do you remember that list of words you tried to learn before?

If the response is "No," remind the individual that you read the list three times and that he or she was asked to recall the words each time. Say the following:

Tell me as many of those words as you can remember.

Record the responses verbatim in the Trial 4 column of the answer form.

<u>Delayed Recognition Administration Instructions</u>

The Delayed Recognition (Forced Choice) trial is administered immediately after the Delayed Recall trial. Say the following:

Now I'm going to read a longer list of words to you. Some of them are words from the original list, and some are not. After I read each word, I'd like you to say "Yes" if it was on the original list or "No" if it was not.

Read the words of the Delayed Recognition trial list in numerical order. Allow the individual as much time as needed to respond. You may use the prompt, **"Was <u>[word]</u> on the list? Yes or no?"** The individual must give you a response for every word. If the individual is not sure, ask for a guess. If the individual gives an incorrect response, continue moving through the word list without pause.

<u>Scoring Instructions</u>

Immediate Recall:

- Enter the total number of correct responses for each Immediate Recall trial on the source document in the appropriate box.

- Score range: 0 - 12 for each Trial 1, 2, & 3.

Delayed Recall & Recognition:

- Enter the total number of correct responses for Trial 4, Delayed Recall, in the appropriate box.

- For Delayed Recognition, enter on page 4 of the booklet the total number of true-positive responses (words identified that were on the list that was presented), the number of semantically-related false-positive errors (words that were not on the list initially presented but are from the same category as a word on the original list; for example furniture, places, or family members), and the number of semantically-unrelated false-positive errors (words that were not on the list initially presented and are not in any way related to any word on that list).

- Score Ranges:
    - Delayed Recall: 0 - 12
    - Delayed Recognition Hits: 0 - 12
    - Delayed Recognition False Alarms: 0 – 12

Remaining scoring items on page 4 of the booklet do not need to be completed. Booklets should be kept as part of the source documentation for that subject.

Benton Judgment of Line Orientation

The Benton Judgment of Line Orientation Task (JLOT) assesses visuo-spatial processing. It examines the ability of a subject to estimate angular relationships between line segments by visually matching angled line pairs to 11 numbered radii forming a semi-circle. Only 15 of the 30 items will be administered at a visit, alternating between ODD and EVEN questions. Each item shows a different pair of angled lines to be matched to two of the numbered radii. A five-item practice set precedes the actual test.

The goal of this test is to assess spatial orientation, not intelligence or the ability to remember instructions. Helping the subject understand the test during the practice component is very important, and explicit instructions (below) are provided to help you do this.

Sometimes subjects continue to do poorly during the practice trials. In the original test, examiners were instructed to discontinue the test entirely if the subject failed to get both responses correct on at least 2 of the 5 practice items. We have modified this. If the subject appears to understand the test, but does poorly on the practice items because of limited spatial ability, the entire test is to be administered.

In contrast, if the subject does poorly on the practice items because of extreme confusion due to dementia, psychosis, or some other cause (i.e., does not understand what the test requires of them, despite instruction) the test should not be administered. This is explained further below.

Booklets are labeled ODD and EVEN.
Make sure to use the correct booklet at the visit.

Administration:

Place stimulus booklet open on table so that subject can see both top and bottom pages. The top page should be held at a 45° angle. Test administrator flips the pages.

Begin with Practice Item A:

See these two lines? Which two lines down here [point to response card] **are in exactly the same position and point in the same direction as the two lines up here? Tell me the numbers of the lines.**

- If subject supplies correct responses, say:
  That's right,
  and proceed to Practice Item B. Administer all Practice Items (A through E).

- If subject is aphasic or tends to misstate the numbers but points to the correct responses, say instead:
  Show me these lines down here. Point to them.

- If subject does not understand task or if he/she does not supply the correct responses for any of the five Practice Items, use the following "Extended Instructions":

Use your hand to cover the line in Position 6, pointing to the other line (in Position 1), say:
Let's just look at this line. Which line down here [point to the response–choice display] **points in the same direction as this one** [point to the stimulus line] **and is also in the same position? That is, it's on the same side of the page as this line up here.**

Record the response on the record sheet where it's labeled 'A'. Correct the subject if the wrong answer is given again. Then proceed, using these Extended Instructions, by covering the other line (in Position 1) and pointing to the line in Position 6. Again, say:
Let's just look at this line. Which line down here [point to the response–choice display] **points in the same direction as this one** [point to the stimulus line] **and is also in the same position? That is, it's on the same side of the page as this line up here.**

If the correct answer is not given for this second single line (A), provide it. Then continue to Practice Item B. If the subject gives the wrong answers for this Practice Item, follow up with the Extended Instructions using single lines (Trial B).

Continue with this cycle of instructions until the subject gives correct responses on two practice items on the first trial (e.g. they correctly answer "4 & 10" for C, so you do not need to go through the single line at a time procedure with C). A correct response means correctly identifying both response choices for the pair of stimulus lines.

After the five practice items have been presented, with the test booklet opened to the page labeled "Test Items", say to the subject:

Now we are going to do more of these, except now the lines which you see up here [point to the upper page] **will be shorter, because part of the line has been erased. Tell me [Show me] which two lines down here are pointing in the same direction as the lines up here.**

Do not supply the subject with knowledge of his/her results on any of the individual test items. However, general statements can be made to provide encouragement and sustain motivation.
If, after 30 seconds, a subject has not given his/her response choices, he/she should be encouraged to make his/her best guess regardless of how uncertain he is about it. There is no time limit for responding.

The subject's actual response choice should be entered on the Record Form (not just "right" or "wrong"). Spontaneous corrections by the subject are accepted. Data entry is item by item rather than by total correct.

Complete all 15 Test Items.

WHAT TO DO IF THE SUBJECT CONTINUES TO DO POORLY ON THE PRACTICE ITEMS

Failure to get both lines correct on any two practice items is considered a valid indication of spatial impairment in subjects who are alert and responsive to the examiner and whose answers, even if incorrect, are appropriate to the instruction.

For example, on Practice Item B where the correct answer is 4 and 8, if the subject gives an erroneous answer, such as 6 and 10, and accurately points to his/her verbal choices on the response-choice display (or, if nonverbal response choices are being used, the subject can reproduce his/her same two pointing responses on command), then the answer is considered to be appropriate and the performance is considered to reflect poor spatial ability.

In this case of poor performance on the practice items, **continue with the full test** once you have cycled through the practice items (A and A', B and B', etc.) regardless of how they have actually scored on the practice items.

YOU SHOULD DISCONTINUE THE TEST AFTER THE PRACTICE ITEMS ONLY IN THE FOLLOWING CIRCUMSTANCE: Very rarely in a study of this kind a subject will show evidence of simply not understanding what the test requires him/her to do, despite the instruction provided by the practice procedure.

Continued inappropriate responding such as selection of only one, or three or more, response choices, or other responses inappropriate to the instructions (e.g., irrationally responding "16 and 41"), the repetition of the same response choices on consecutive practice items, or failure to make any response choices, are considered to be invalid performances in that they probably reflect the presence of confusion, dementia, psychosis, failure to cooperate, impairment in language comprehension, or failure to acquire "test-set", independently of the specific ability to judge line orientation. In these circumstances, do not administer the test and document the reasons on the SDW and on the eCRF.

Scoring:
For each item, record the subject's two response choices. Both responses must be correct for the item to be scored as correct. The correct responses are printed on the work sheet. Place a check next to correct items for purposes of data entry.

Score range: 0 – 15

Semantic Fluency (Animal, Vegetables, Fruit)

Administration:

SAY: "I am going to give you a category and I want you to name, as fast as you can, all of the things that belong in that category.

For example, if I say 'articles of clothing,' you could say 'shirt,' 'tie,' or 'hat.' Can you think of other articles of clothing?"

Allow up to 20 seconds for the subject to produce two responses. If they cannot, clarify the task as best you can and then proceed with the actual testing (Animals, Vegetables, Fruits).

Time limit: 60 seconds per trial.

Response recording: Try to capture verbatim, but if you can't keep up with at least the first syllable of a response, use a check-mark until you can.

- Written recording helps you catch repetitions and incorrect responses. It also allows others to review your scoring. Consider audio-taping with appropriate consent.

Scoring:

The scoring is liberal. Productivity is favored over semantic exactness.

Animals = Total number of correct unique animal names produced within 60 seconds.

- CREDIT: breeds (e.g., terriers); male, female, and infant names of a species (e.g., bull, cow, calf); both superordinate and subordinate examples of a species (e.g., both dog and terrier are credited); birds; fish; reptiles, insects.

- DO NOT CREDIT: Repetitions, mythical animals.

Vegetable = Total number of correct unique names of vegetables produced within 60 seconds.

- CREDIT: Both superordinate and subordinate responses (e.g., peppers and jalapenos are credited); less specific names (e.g., greens); nuts (e.g., peanuts, acorns); and grains such as corn or rice.

- Names of vegetables found in other cultures but perhaps unfamiliar to you (e.g., Jicama) are acceptable only if they can be verified in the dictionary.
- After completion of the task, ask the subject to spell the word if you are unsure of the correct spelling & wish to check on the item.
- Grains (e.g. rice, wheat, oats, etc.), gourds, sugarcane, herbs and seaweed are counted as acceptable 'vegetable' responses.
- Tomato, avocado and pumpkin are acceptable responses.

➢ DO NOT CREDIT: Repetitions. Prepared vegetable products are not acceptable responses (e.g. pickles, tomato sauce, ketchup, etc.).

Fruit = Total number of correct unique names of fruits produced within 60 seconds. Some items (e.g. tomato, avocado) count as either a fruit or vegetable, but you should only give credit once, i.e., if the subject gives "tomato" as vegetable, and then again as a fruit, do not give credit the second time.

Count raisins & sultanas as well as grapes. If you are uncertain of a response, after the completion of the trial ask the subject to spell the item so that you can check it in the dictionary &/or obtain opinions from other examiners.

Letter Number Sequencing

Materials: Letter-Number Sequencing CRF source worksheet.

Definitions: An example of a trial is 1a. A trial can be scored as a 0 or 1.
An example of an item is the sum of 1a through 1c.
Each item is composed of 3 trials.

The highest score on an item is a total of 3. This occurs if each trial of an item, e.g. 1a through 1c, is scored as a 1.

Description:

For this test, the subject is read a combination of numbers and letters and is asked to recall the numbers first in ascending order and then the letters in alphabetical order. Each item consists of three trials, and each trial is a different combination of numbers and letter.

Start:

Conduct the practice session with the subject, see practice items below. Then conduct the assessment of Items 1a – 7c.

When to Discontinue Test:

Discontinue if a subject scores a **0** on ***all three trials*** of an item (e.g., if subject scored a "0" for item 3a, 3b and 3c, then the assessment is not continued). Leave remaining responses blank.

General Directions:

Administer all practice trials. For each Practice Item and item trial, say each combination at a rate of one number or letter per second. Allow the examinee ample time to respond (correct responses are in parentheses).

Item Instructions:

Practice Items.

Practice Example: B – 7 (7 – B)
B - 7 is read to the subject; the correct response is in parentheses.

Say to the subject: "**I am going to say a group of numbers and letters. After I say them, I want you to tell me the numbers first, in order, starting with the lowest number. Then tell me the letters in alphabetical order. For example, if I say 'B – 7', your answer should be '7 – B. The number goes first, then the letter. If I say '9 – C – 3, then your answer should be '3 – 9 – C', the numbers in order first, then the letters in alphabetical order. Let's practice:"**

**6 – F**	(6 – F)
**G – 4**	(4 – G)
**3 – W – 5**	(3 – 5 –W)
**T – 7 – L**	(7 – L – T)
**1 – J – A**	(1 – A – J)

If the subject makes an error on any Practice Item, correct him or her and repeat the instructions as necessary. Even if the subject fails all Practice Items, continue with the subtest.

Scoring:

The correct response appears in parentheses on the CRF. Record the subject's response to each trial ***verbatim*** next to or below the item on the CRF source worksheet.

For each trial of an item, score 1 point for each correct response, 0 points for each incorrect response. A response is incorrect if a number or letter is omitted or if the numbers and letters are not said in the specified sequence.

- The highest score on an item is a total of 3. This occurs if each trial of an item e.g. 1a through 1c is scored as a 1.
- ***As long as the numbers and letters are recalled in sequence, give credit if the examinee gives the letters in sequence before the numbers.***
- Allow the subject to make self-corrections if they recognize that they are making an error but do not prompt them to make self-corrections.

Symbol Digit Modalities Test

This test evaluates a person's speed of processing. Subjects are asked to write in the numbers that go with the symbols presented in a *Key* at the top of the page. The *Key* shows how the symbols and numbers are paired up correctly. They are given 90 seconds in which to complete as many pairings as quickly as they can. Form 1 will be used at Baseline visit and will alternate annually with Form 2.

Administration: Place the test form on the table in front of the subject and say,

Look at the boxes at the top of the page. Each box in the upper row has a symbol in it, and each box below it has a number.

Now look at the next line of boxes [point to the first line of boxes without numbers]**. Notice that the boxes on the top have symbols, but the boxes beneath are empty.**

You are to fill in each empty box with the number that goes with each symbol, according to the way they are paired at the top of the page. For example, if you look at the first symbol, [point to the first symbol in the row beneath the key]**, and then look up at the key, you see that this symbol is paired with the number "1"** [show the pairing]**. So, you would write a "1" in this box** [write a "1" in the first box]**.**

This next symbol [point to the next symbol] **is paired with "5", so you would put a "5" in this box** [write "5" in the second box]**. Now, what number goes in this box** [point to third box]**?** These instructions may differ, depending on the version of the form being used.

Subject should say "two." If not, correct the subject and explain the error.

When the subject appears to comprehend the task, say,

Good. Now, for practice, fill in the boxes up to this double line, and then stop.

Correct immediately any errors made during the practice period, explaining the subject's error. Repeat the instructions and review the correct coding of the practice boxes as necessary until the subject understands the task.

Continue with the test by saying,

When I say "Go," write in the numbers just like you have been doing as fast as you can until I say "Stop." Work as quickly as you can, moving from one line to the next, without skipping

any boxes. If you make a mistake, cross it out and write the correct answer below. Remember to work as quickly as you can. Ready? Go!

Start timing. Do not allow the subject to skip any boxes. Do not intervene if the subject records a number incorrectly.

At the end of <u>90 seconds</u>, say, "STOP!" Be sure that the subject does not continue working after the time limit is reached.

The score is the number of *correct* responses in 90 seconds. Do not include the practice items or incorrect responses in the total score.

Score range: 0 - 110

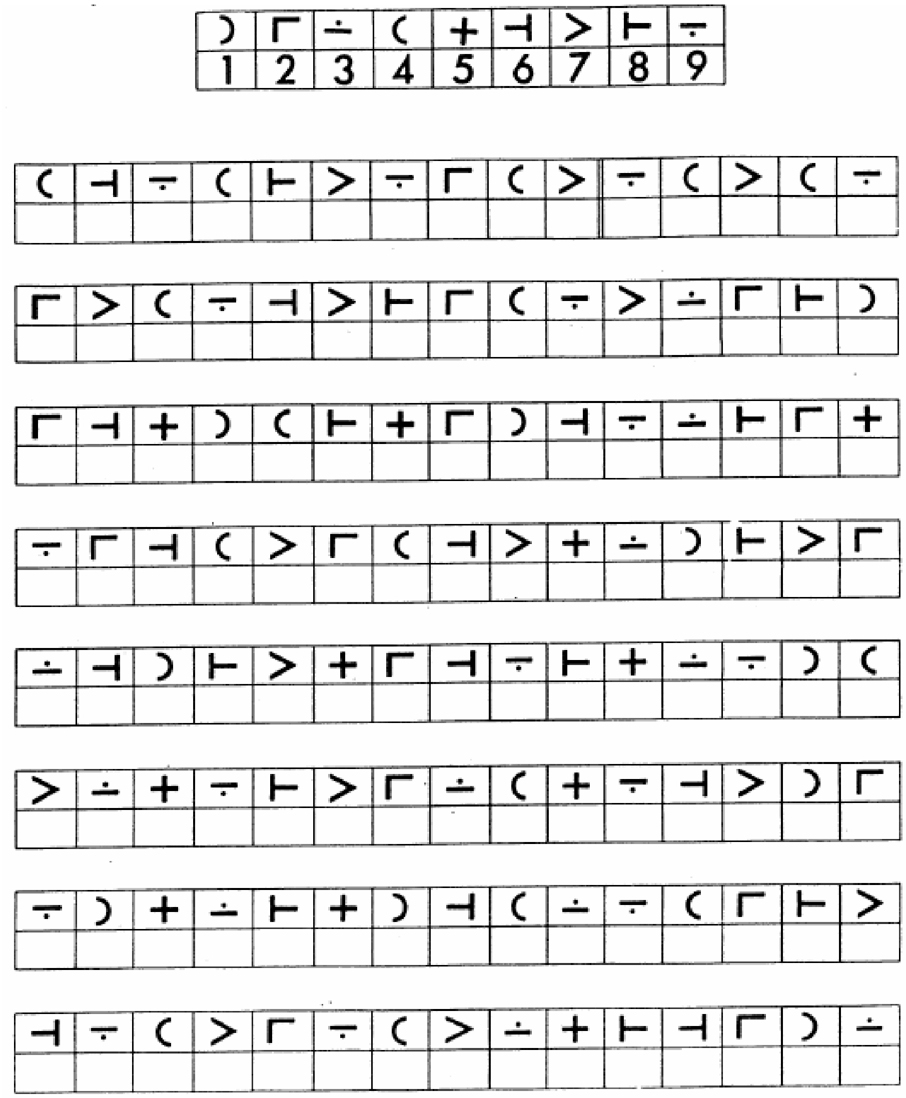

Montreal Cognitive Assessment (MoCA)

The Montreal Cognitive Assessment (MoCA) was designed as a rapid screening instrument for mild cognitive dysfunction. It assesses different cognitive domains: attention and concentration, executive functions, memory, language, visuoconstructional skills, conceptual thinking, calculations, and orientation. Time to administer the MoCA is approximately 10 minutes. The total possible score is 30 points.

1. Alternating Trail Making:

Administration: The examiner instructs the subject: "Please draw a line, going from a number to a letter in ascending order. Begin here [point to (1)] and draw a line from 1 then to A then to 2 and so on. End here [point to (E)]."

Scoring: Allocate one point if the subject successfully draws the following pattern: 1 –A- 2- B- 3- C- 4- D- 5- E, without drawing any lines that cross. Any error that is not immediately self-corrected earns a score of 0.

2. Visuoconstructional Skills (Cube):

Administration: The examiner gives the following instructions, pointing to the **cube**: "Copy this drawing as accurately as you can, in the space below".

Scoring: One point is allocated for a correctly executed drawing.

- Drawing must be three-dimensional
- All lines are drawn
- No line is added
- Lines are relatively parallel and their length is similar (rectangular prisms are accepted)

A point is not assigned if any of the above-criteria are not met.

3. Visuoconstructional Skills (Clock):

Administration: Indicate the right third of the space and give the following instructions: "Draw a **clock**. Put in all the numbers and set the time to 10 after 11".

Scoring: One point is allocated for each of the following three criteria:

- Contour (1 pt.): the clock face must be a circle with only minor distortion acceptable (e.g., slight imperfection on closing the circle);
- Numbers (1 pt.): all clock numbers must be present with no additional numbers; numbers must be in the correct order and placed in the approximate quadrants on the clock face; Roman numerals are acceptable; numbers can be placed outside the circle contour;

- Hands (1 pt.): there must be two hands jointly indicating the correct time; the hour hand must be clearly shorter than the minute hand; hands must be centered within the clock face with their junction close to the clock centre.

A point is not assigned for a given element if any of the above-criteria are not met.

4. Naming:

Administration: Beginning on the left, point to each figure and say: "Tell me the name of this animal".

Scoring: One point each is given for the following responses: (1) lion, (2) rhinoceros or rhino, (3) camel or dromedary.

5. Memory:

Administration: The examiner reads a list of 5 words at a rate of one per second, giving the following instructions: "This is a memory test. I am going to read a list of words that you will have to remember now and later on. Listen carefully. When I am through, tell me as many words as you can remember. It doesn't matter in what order you say them". Mark a check in the allocated space for each word the subject produces on this first trial. When the subject indicates that (s)he has finished (has recalled all words), or can recall no more words, read the list a second time with the following instructions: "I am going to read the same list for a second time. Try to remember and tell me as many words as you can, including words you said the first time." Put a check in the allocated space for each word the subject recalls after the second trial.
At the end of the second trial, inform the subject that (s)he will be asked to recall these words again by saying, "I will ask you to recall those words again at the end of the test."

Scoring: No points are given for Trials One and Two.

6. Attention:

Forward Digit Span: Administration: Give the following instruction: "I am going to say some numbers and when I am through, repeat them to me exactly as I said them". Read the five number sequence at a rate of one digit per second.

Backward Digit Span: Administration: Give the following instruction: "Now I am going to say some more numbers, but when I am through you must repeat them to me in the backwards order." Read the three number sequence at a rate of one digit per second.

Scoring: Allocate one point for each sequence correctly repeated, (N.B.: the correct response for the backwards trial is 2-4-7).

Vigilance: Administration: The examiner reads the list of letters at a rate of one per second, after giving the following instruction: "I am going to read a sequence of letters. Every time I say the letter A, tap your hand once. If I say a different letter, do not tap your hand".

Scoring: Give one point if there is zero to one errors (an error is a tap on a wrong letter or a failure to tap on letter A)

Serial 7s: Administration: The examiner gives the following instruction: "Now, I will ask you to count by subtracting seven from 100, and then, keep subtracting seven from your answer until I tell you to stop." Give this instruction twice if necessary.

Scoring: This item is scored out of 3 points. Give no (0) points for no correct subtractions, 1 point for one correction subtraction, 2 points for two-to-three correct subtractions, and 3 points if the participant successfully makes four or five correct subtractions. Count each correct subtraction of 7 beginning at 100. Each subtraction is evaluated independently; that is, if the participant responds with an incorrect number but continues to correctly subtract 7 from it, give a point for each correct subtraction. For example, a participant may respond "92 – 85 – 78 – 71 – 64" where the "92" is incorrect, but all subsequent numbers are subtracted correctly. This is one error and the item would be given a score of 3.

7. Sentence repetition:

Administration: The examiner gives the following instructions: "I am going to read you a sentence. Repeat it after me, exactly as I say it [pause]: **I only know that John is the one to help today."** Following the response, say: "Now I am going to read you another sentence. Repeat it after me, exactly as I say it [pause]: **The cat always hid under the couch when dogs were in the room.**"

Scoring: Allocate 1 point for each sentence correctly repeated. Repetition must be exact. Be alert for errors that are omissions (e.g., omitting "only", "always") and substitutions/additions (e.g., "John is the one who helped today;" substituting "hides" for "hid", altering plurals, etc.).

8. Verbal fluency:

Administration: Continuing within the sequence of this test, the examiner gives the following instruction: "Tell me as many words as you can think of that begin with a certain letter of the alphabet that I will tell you in a moment. You can say any kind of word you want, except for proper nouns (like Bob or Boston), numbers, or words that begin with the same sound but have a different suffix, for example, love, lover, loving. I will tell you to stop after one minute. Are you ready? [Pause] Now, tell me as many

words as you can think of that begin with the letter F. [time for 60 sec]. Stop." Record the subject's responses on the "MoCA Fluency: Letter F" response sheet.

Scoring: On the MoCA worksheet, 11 or more correct responses = 1; 10 or less = 0. On the MoCA page in EDC, also enter the total number of correct responses for "F" phonemic fluency as recorded on the response sheet.

9. Abstraction:

Administration: The examiner asks the subject to explain what each pair of words has in common, starting with the example: "Tell me how an orange and a banana are alike". If the subject answers in a concrete manner, then say only one additional time: "Tell me another way in which those items are alike". If the subject does not give the appropriate response (fruit), say, "Yes, and they are also both fruit." Do not give any additional instructions or clarification.

After the practice trial, say: "Now, tell me how a train and a bicycle are alike". Following the response, administer the second trial, saying: "Now tell me how a ruler and a watch are alike". Do not give any additional instructions or prompts.

Scoring: Only the last two item pairs are scored. Give 1 point to each item pair correctly answered. The following responses are acceptable:
Train-bicycle = means of transportation, means of traveling, you take trips in both;
Ruler-watch = measuring instruments, used to measure.
The following responses are **not** acceptable: Train-bicycle = they have wheels; Ruler-watch = they have numbers.

10. Delayed recall:

Administration: The examiner gives the following instruction: "I read some words to you earlier, which I asked you to remember. Tell me as many of those words as you can remember. Make a check mark for each of the words correctly recalled spontaneously without any cues, in the allocated space.

Scoring: **Allocate 1 point for each word recalled freely without any cues**.

Optional:
Following the delayed free recall trial, prompt the subject with the semantic category cue provided below for any word not recalled. Make a check mark in the allocated space if the subject remembered the word with the help of a category or multiple-choice cue. Prompt all non-recalled words in this manner. If the subject does not recall the word after the category cue, give him/her a multiple choice trial, using the following example instruction, "Which of the following words do you think it was, NOSE, FACE, or HAND?"
Use the following category and/or multiple-choice cues for each word, when appropriate:
FACE: category cue: part of the body multiple choice: nose, face, hand
VELVET: category cue: type of fabric multiple choice: denim, cotton, velvet

CHURCH: category cue: type of building multiple choice: church, school, hospital
DAISY: category cue: type of flower multiple choice: rose, daisy, tulip
RED: category cue: a color multiple choice: red, blue, green
Scoring: **No points are allocated for words recalled with a cue.** A cue is used for clinical information purposes only and can give the test interpreter additional information about the type of memory disorder. For memory deficits due to retrieval failures, performance can be improved with a cue. For memory deficits due to encoding failures, performance does not improve with a cue.

11. Orientation:

Administration: The examiner gives the following instructions: "Tell me the date today". If the subject does not give a complete answer, then prompt accordingly by saying: "Tell me the [year, month, exact date, and day of the week]." Then say: "Now, tell me the name of this place, and which city it is in."

Scoring: Give one point for each item correctly answered. The subject must tell the exact date and the exact place (name of hospital, clinic, office). No points are allocated if subject makes an error of one day for the day and date.

TOTAL SCORE: Sum all sub-scores listed on the right-hand side. Add one point for an individual who has 12 years or fewer of formal education, for a possible maximum of 30 points.

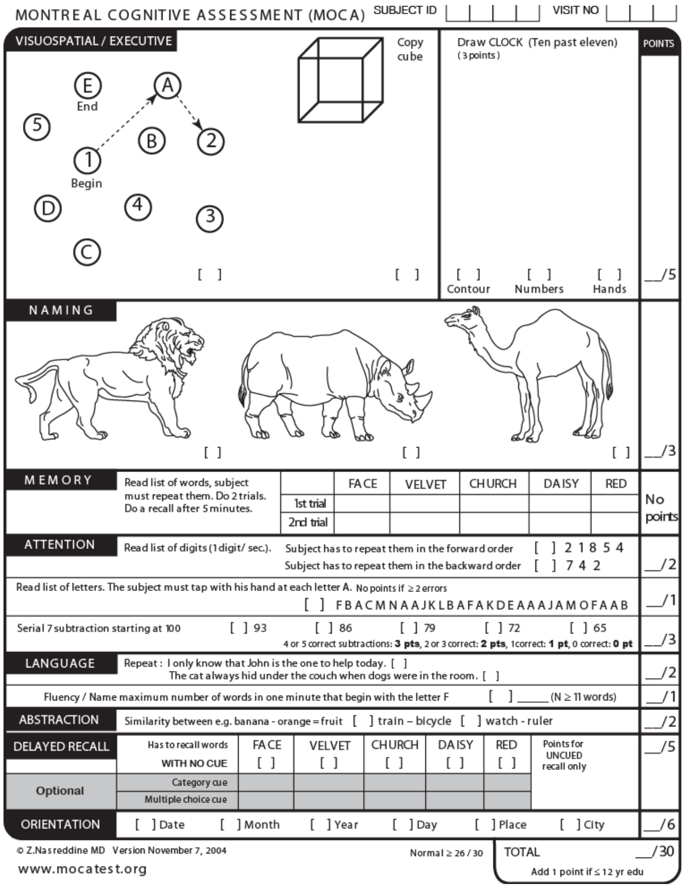
MONTREAL COGNITIVE ASSESSMENT (MOCA) SUBJECT ID VISIT NO

VISUOSPATIAL / EXECUTIVE — Copy cube — Draw CLOCK (Ten past eleven) (3 points) — POINTS
E End, A, 5, B, 2, 1 Begin, D, 4, 3, C
[ ] [ ] [ ] Contour [ ] Numbers [ ] Hands __/5

NAMING [ ] [ ] [ ] __/3

MEMORY	Read list of words, subject must repeat them. Do 2 trials. Do a recall after 5 minutes.		FACE	VELVET	CHURCH	DAISY	RED	No points
		1st trial						
		2nd trial						

ATTENTION — Read list of digits (1 digit/ sec.). Subject has to repeat them in the forward order [ ] 2 1 8 5 4; Subject has to repeat them in the backward order [ ] 7 4 2 __/2

Read list of letters. The subject must tap with his hand at each letter A. No points if ≥ 2 errors [ ] F B A C M N A A J K L B A F A K D E A A A J A M O F A A B __/1

Serial 7 subtraction starting at 100 [ ] 93 [ ] 86 [ ] 79 [ ] 72 [ ] 65 __/3
4 or 5 correct subtractions: **3 pts**, 2 or 3 correct: **2 pts**, 1 correct: **1 pt**, 0 correct: **0 pt**

LANGUAGE — Repeat: I only know that John is the one to help today. [ ] The cat always hid under the couch when dogs were in the room. [ ] __/2

Fluency / Name maximum number of words in one minute that begin with the letter F [ ] ____ (N ≥ 11 words) __/1

ABSTRACTION — Similarity between e.g. banana - orange = fruit [ ] train – bicycle [ ] watch - ruler __/2

DELAYED RECALL	Has to recall words WITH NO CUE	FACE [ ]	VELVET [ ]	CHURCH [ ]	DAISY [ ]	RED [ ]	Points for UNCUED recall only	__/5
Optional	Category cue							
	Multiple choice cue							

ORIENTATION [ ] Date [ ] Month [ ] Year [ ] Day [ ] Place [ ] City __/6

© Z.Nasreddine MD Version November 7, 2004 — Normal ≥ 26 / 30 — TOTAL __/30 — Add 1 point if ≤ 12 yr edu
www.mocatest.org

Epworth Sleepiness Scale

Administration:

The ESS asks people to rate, on a 4-point scale (0 – 3), their usual chances of dozing off or falling asleep in 8 different situations or activities that most people engage in as part of their daily lives, although not necessarily every day. It does not ask people how often they doze off in each situation. That would depend very much on how often they happened to be in those situations. Rather it asks what the chances are that they would doze off whenever they were in each situation. This requires a mental judgment which, it seems, most people are able to make in a meaningful way. The total ESS score is the sum of 8 item-scores and can range between 0 and 24.The higher the score, the higher the person's level of daytime sleepiness. Most people can answer the ESS, without assistance, in 2 or 3 minutes.

Ensure the following instructions (also provided on the assessment worksheet) are read to the subject prior to administering the scale:

> How likely are you to doze off or fall asleep in the following situations, in contrast to feeling just tired?
> This refers to your usual way of life in recent times.
> Even if you haven't done some of these things recently try to work out how they would have affected you.

What does it mean to ask about 'Recent Times'?

Respondents to the ESS rate their chances of dozing off in particular situations 'in recent times'. It was a deliberate decision not to specify this time scale more accurately. It was intended to mean long enough for the subject to have experienced each situation referred to and to have formed an estimate of his/her chances of dozing in each. This may be a few weeks to a few months. However, experience with the rapid changes in sleep propensity that occur when patients with obstructive sleep apnea are treated with nasal CPAP suggests that periods of recall as short as a week or two may be possible to use with the ESS.

How to Score the ESS?

Most people can answer the ESS without difficulty in a few minutes, but some cannot decide on one number (0-3), and instead write down ½ or 1½, etc. for some answers. It is recommended that these scores be taken at face value, adding up all 8 item-scores, including halves. If the total ESS score includes a half (e.g. 6½) that score should be rounded up to the next whole number. If one or more item-scores is missing, that ESS is invalid. It is not feasible to interpolate missing item-scores.

EPWORTH SLEEPINESS SCALE

A. Source of Information: 1 = Patient, 2 = Caregiver, 3 = Patient and caregiver A. ☐

How likely are you to doze off or fall asleep in situations described below, in contrast to feeling just tired?

This refers to your usual way of life in recent times.

Even if you haven't done some of these things recently try to work out how they would have affected you.

Use the following scale to choose the **most appropriate number** for each situation:

0 = **would never** doze
1 = **slight chance** of dozing
2 = **moderate chance** of dozing
3 = **high chance** of dozing

It is important that you answer each question as best you can.

1. Sitting and reading 1. ☐
2. Watching TV 2. ☐
3. Sitting, inactive in a public place (e.g., a theatre or a meeting) 3. ☐
4. As a passenger in a car for an hour without a break 4. ☐
5. Lying down to rest in the afternoon when circumstances permit 5. ☐
6. Sitting and talking to someone 6. ☐
7. Sitting quietly after a lunch without alcohol 7. ☐
8. In a car, while stopped for a few minutes in the traffic 8. ☐

© M. W. Johns 1990-1997

REM Sleep Behavior Disorder Questionnaire

The REM Sleep Behavior Disorder Screening Questionnaire (RBDSQ) is a 10 item, subject self-rating instrument assessing sleep behavior with short questions that have to be answered either "yes" or "no". Questions are framed to gather information about current behaviors. For purposes of PPMI, "current" is defined as within 6 months of the study visit.

Instructions and scoring:

1) Since subjects do not always have a long-time companion, the bed partner's input is encouraged, but not required.

2) Subjects should respond to each question. If they are not certain, they should choose the response (either yes or no) that is most likely.

3) For question 10, more than one nervous system disease may be recorded. All PD subjects should record "parkinsonism".

4) Questions 6.1 to 6.4 should be marked "yes" if any of the phenomena described in the appropriate line is present.

5) Scoring will be performed centrally.

REM SLEEP DISORDER QUESTIONNAIRE

A. Source of Information: 1 = Patient, 2 = Caregiver, 3 = Patient and caregiver — A. ☐

1. I sometimes have very vivid dreams. (0 = No, 1 = Yes) — 1. ☐

2. My dreams frequently have an aggressive or action-packed content. (0 = No, 1 = Yes) — 2. ☐

3. The dream contents mostly match my nocturnal behaviour. (0 = No, 1 = Yes) — 3. ☐

4. I know that my arms or legs move when I sleep. (0 = No, 1 = Yes) — 4. ☐

5. It thereby happened that I (almost) hurt my bed partner or myself. (0 = No, 1 = Yes) — 5. ☐

6. I have or had the following phenomena during my dreams:

   6.1 speaking, shouting, swearing, laughing loudly (0 = No, 1 = Yes) — 6.1 ☐

   6.2 sudden limb movements, "fights" (0 = No, 1 = Yes) — 6.2 ☐

   6.3 gestures, complex movements, that are useless during sleep, e.g., to wave, to salute, to frighten mosquitoes, falls off the bed (0 = No, 1 = Yes) — 6.3 ☐

   6.4 things that fell down around the bed, e.g., bedside lamp, book, glasses (0 = No, 1 = Yes) — 6.4 ☐

7. It happens that my movements awake me. (0 = No, 1 = Yes) — 7. ☐

8. After awakening I mostly remember the content of my dreams well. (0 = No, 1 = Yes) — 8. ☐

9. My sleep is frequently disturbed. (0 = No, 1 = Yes) — 9. ☐

10. I have/had a disease of the nervous system: (0 = No, 1 = Yes)

   10a. stroke — 10a. ☐

   10b. head trauma — 10b. ☐

   10c. parkinsonism — 10c. ☐

   10d. RLS — 10d. ☐

   10e. narcolepsy — 10e. ☐

   10f. depression — 10f. ☐

   10g. epilepsy — 10g. ☐

   10h. inflammatory disease of the brain — 10h. ☐

   10i. other, specify: ______________________________ — 10i. ☐

Geriatric Depression Scale (Short) – GDS-15

A self-report instrument to be completed by the subject. The subject should choose the best answer for how they have felt over the past week. A caregiver may provide input and assistance.

- An answer of 0= NO for questions 1, 5, 7, 11, and 13 indicate depression.
- A score of 1=YES on the rest of the questions (2,3,4,6,8,9,10,12,14,15) indicate depression.
- Note: Only raw scores are captured in the database.
- Sites may manually tally the total score to assess levels of depression. Count 1 point for each item answered in such a way as to indicate depression. If the GDS-15 score indicates at least moderate depression (score of 8 or higher), the PPMI site investigator should be notified, and clinical assessment for significant depression should be considered.

What to do if a subject misses a couple items?
Calculate the ratio of correct responses to the total number of items answered. Multiple this ratio by the number of missing items and add it to the number of correct responses.

For example: If 3 of 15 items are missed, the total score is calculated on 12 completed PLUS 3/15ths of the total score to make-up for omitted items, e.g. if they got a 4 on the 12 they completed or 1/3 positive, then add 1/3 of the 3 missing or 1 point for a total score of 5 of 15.

Note: If 12 or more items are missed, the test will be considered invalid.

Categories:

0 – 4 Normal depending on age, education, complaints
5 – 8 Mild depression
8 – 11 Moderate depression
12-15 Severe depression

GERIATRIC DEPRESSION SCALE (Short Version)

Choose the best answer for how you have felt over the **past week**. (0 = No, 1 = Yes)

1.	Are you basically satisfied with your life?	1. ☐
2.	Have you dropped many of your activities and interests?	2. ☐
3.	Do you feel that your life is empty?	3. ☐
4.	Do you often get bored?	4. ☐
5.	Are you in good spirits most of the time?	5. ☐
6.	Are you afraid that something bad is going to happen to you?	6. ☐
7.	Do you feel happy most of the time?	7. ☐
8.	Do you often feel helpless?	8. ☐
9.	Do you prefer to stay at home, rather than going out and doing new things?	9. ☐
10.	Do you feel you have more problems with memory than most?	10. ☐
11.	Do you think it is wonderful to be alive now?	11. ☐
12.	Do you feel pretty worthless the way you are now?	12. ☐
13.	Do you feel full of energy?	13. ☐
14.	Do you feel that your situation is hopeless?	14. ☐
15.	Do you think that most people are better off than you are?	15. ☐

Sheikh JI, Yesavage JA: Geriatric Depression Scale (GDS): Recent evidence and development of a shorter version. Clinical Gerontology: A Guide to Assessment and Intervention. 165-173, NY: The Haworth Press, 1986. (10/16/07) 5/6/10 Page 1 of 1

State-Trait Anxiety Inventory

Administration:

Note: It is suggested to administer this test after the Lumbar Puncture has been completed.

The STAI was designed to be self-administering and may be given either individually or to groups. The inventory has no time limits. College students generally require about six minutes to complete either the S-Anxiety or the T-Anxiety scale, and approximately ten minutes to complete both. Less educated or emotionally disturbed persons may require ten minutes to complete one of the scales and approximately twenty minutes to complete both. Repeated administrations of the S-Anxiety scale typically require five minutes or less.

Although many of the items have face validity as measures of "anxiety," the examiner should not use this term in administering the inventory. Rather, the STAI and its subscales should be consistently referred to as the *Self-Evaluation Questionnaire*, the title printed on the test form.

Examiners should establish rapport with respondents before administering the STAI. Approximately half of the items inquire about negative characteristics, (e.g., feeling "tense," "frightened," or "upset"), and some people are reluctant to admit having these characteristics because they regard them as signs of weakness. More over, persons who desire to look good in the eyes of the examiner may respond more positively to anxiety-absent items (e.g., "I feel calm") than they actually feel. To deal with such test taking attitudes in individual clinical applications, the examiner needs to establish a trusting relationship with examinees by sincerely communicating that their honest and candid responses will enable the therapist or agency to be more helpful and effective. Similarly, in research settings, subjects generally respond more objectively and accurately if they are informed that their responses will be kept confidential, and especially, if they are promised feedback about their test results. Clinical and research findings suggest that distorting effects of adverse test-taking attitudes are not a serious problem if sufficient care is taken to obtain the cooperation and trust of the respondent at the time the STAI is administered. However, in situations in which there are strong reasons to expect that respondents are motivated to "fake good," e.g., in screening applicants for employment, the STAI should only be used as part of a test battery that includes validity measures such as the MMPI Lie Scale. For applicants with high Lie scores, it may be assumed that STAI T-Anxiety scores underestimate the subject's anxiety proneness.

Complete instructions for the S-Anxiety and the T-Anxiety scales are printed on the test form. Critical to the validity of the inventory is the examinees' clear understanding of the "state" instructions, which require them to report how they feel "right now ... at this moment," and the "trait" instructions, which ask them to indicate how they "generally" feel. The examiner should emphasize that instructions are different for the two parts of the inventory and that examinees must read both sets of instructions carefully.

Exclusion Criteria for Prodromal and Genetic Cohort Unaffected :

The score of 54 or above for STAI Form Y-1 requires Investigator discretion to enter the study.

Scoring :

Please refer to the STAI answer key posted in ePortal in Source Documents Worksheets folder.

Questionnaire for Impulsive-Compulsive Disorders (QUIP-SHORT)

The QUIP-SHORT is a validated, brief scale for screening for impulse control disorder behaviors.

Instructions for Administration and Scoring:

1) The QUIP is a self-report instrument. It may be completed by a subject, informant or both. Record who completed the scale at the top of the page.

2) If appropriate, reinforce that the subject should answer all items, including those (like sexual function) that are potentially sensitive. Responses to these questions will not be disclosed without first informing the subject. However, if the QUIP responses indicate the presence of a significant ICD, the study staff should inform the PPMI site investigator and clinical follow-up should be considered. Contact PPMI staff if you are not sure how to proceed if the QUIP suggests an ICD.

3) The QUIP will be scored centrally. The scoring rule is provided, below, for your information.

Short Instrument	Number of endorsed items for positive screen
A. Compulsive gambling	any 1 of the 2 gambling items
B. Compulsive sexual behavior	any 1 of the 2 sexual behavior items
C. Compulsive buying	any 1 of the 2 buying items
D. Compulsive eating	any 1 of the 2 eating items
E. Other Behaviors	
Hobbyism	item #1
Punding	item #2
Walkabout	item #3
F. Medication Use	any 1 of the 2 items

Questionnaire for Impulsive-Compulsive Disorders in Parkinson's Disease (QUIP-Current-Short)

Reported : _____ Patient _____ Informant* _____ Patient and Informant

Patient name: ________________________________

Date: ________________________________

*If information reported by an informant, answer questions based on your understanding of the patient.

Answer ALL QUESTIONS based on CURRENT BEHAVIORS LASTING AT LEAST 4 WEEKS

A. GAMBLING

1. Do you or others think you have an issue with too much gambling behaviors (such as casinos, internet gambling, lotteries, scratch tickets, betting, or slot or poker machines)? __Yes __No

2. Do you have difficulty controlling your gambling behaviors (such as increasing them over time, or having trouble cutting down or stopping them)? __Yes __No

B. SEX

1. Do you or others think you have an issue with too much sex behaviors (such as making sexual demands on others, promiscuity, prostitution, change in sexual orientation, masturbation, internet or telephone sexual activities, or pornography)? __Yes __No

2. Do you think too much about sex behaviors (such as having trouble keeping thoughts out of your mind or feeling guilty)? __Yes __No

C. BUYING

1. Do you or others think you have an issue with too much buying behaviors (such as too much of the same thing or things that you don't need or use)? __Yes __No

2. Do you engage in activities specifically to continue the buying behaviors (such as hiding what you're doing, lying, hoarding things, borrowing from others, accumulating debt, stealing, or being involved in illegal acts)? __Yes __No

D. EATING

1. Do you or others think you have an issue with too much eating behaviors (such as eating larger amounts or different types of food than in the past, more rapidly than normal, until feeling uncomfortably full, or when not hungry)? __Yes __No

2. Do you have urges or desires for eating behaviors that you feel are excessive or cause you distress (including becoming restless or irritable when unable to participate in the behavior)? __Yes __No

E. OTHER BEHAVIORS

Do you or others think that you spend too much time….

1. On specific tasks, hobbies or other organized activities (such as writing, painting, gardening, repairing or dismantling things, collecting, computer use, working on projects, etc.)? __Yes __No

2. Repeating certain simple motor activities (such as cleaning, tidying, handling, examining, sorting, ordering, or arranging objects, etc.)? __Yes __No

3. Walking or driving with no intended goal or specific purpose? __Yes __No

F. MEDICATION USE

1. Do you or others (including your physicians) think that you consistently take too much of your Parkinson's medications? __Yes __No

2. Do you have difficulty controlling your use of Parkinson's medications (such as experiencing a strong desire for more medication, or having worse mood or feeling unmotivated at a lower dosage)? __Yes __No

SCOPA-AUT

The SCOPA-AUT is a 26 item self-report questionnaire of autonomic function. There are questions covering upper and lower gastro-intestinal function, urinary function, cardio-circulatory function, sexuality and other miscellaneous autonomic problems (e.g. sweating, light sensitivity).

Instructions for Administration and Scoring:

1) Ask the subject to answer the questions by placing a cross in the box that best reflects their situation. If they wish to change an answer, fill in the 'wrong' box and place a cross in the correct one.

2) If they have used medication in the past month in relation to one or more of the problems mentioned, then the question refers to how the subject was while taking this medication. They can note the use of medication on the last page.

3) It is permissible to have input from a spouse or other knowledgeable informant when completing the SCOPA-AUT.

4) If appropriate, reinforce that the subject should answer all items, including those (like sexual function) that are potentially sensitive. Responses to these questions will not be disclosed without permission from the subject.

5) Do not calculate a total score. Scoring will be done centrally.

A. Source of Information: 1 = Patient, 2 = Caregiver, 3 = Patient and caregiver A. ☐

SCOPA-AUT

By means of this questionnaire, we would like to find out to what extent in the past month you have had problems with various bodily functions, such as difficulty passing urine, or excessive sweating. Answer the questions by placing a cross in the box which best reflects your situation. If you wish to change an answer, fill in the 'wrong' box and place a cross in the correct one. If you have used medication in the past month in relation to one or more of the problems mentioned, then the question refers to how you were while taking this medication. You can note the use of medication on the last page.

1. In the past month have you had difficulty swallowing or have you choked?

☐ never ☐ sometimes ☐ regularly ☐ often

2. In the past month, has saliva dribbled out of your mouth?

☐ never ☐ sometimes ☐ regularly ☐ often

3. In the past month, has food ever become stuck in your throat?

☐ never ☐ sometimes ☐ regularly ☐ often

4. In the past month, did you ever have the feeling during a meal that you were full very quickly?

☐ never ☐ sometimes ☐ regularly ☐ often

5. *Constipation is a blockage of the bowel, a condition in which someone has a bowel movement twice a week or less.*
In the past month, have you had problems with constipation?

☐ never ☐ sometimes ☐ regularly ☐ often

6. In the past month, did you have to strain hard to pass stools?

☐ never ☐ sometimes ☐ regularly ☐ often

Cognitive Categorization

The Cognitive Categorization assessment is used to make a determination of Parkinson Disease Dementia (PDD) and PD with mild cognitive impairment (PD-MCI). Information for this assessment is provide through a combination of responses from the subject or other informant, the Investigator's judgment, and results from the cognitive testing covering four cognitive domains.

The determination of PDD will be made on the following factors:

1. History of cognitive decline determined by the investigator based on information from the patient, other informant (spouse, family member or friend) and the investigator's judgment.
2. Cognitive impairment defined as at least 1 test score (out of 6 scores) from at least 2 domains (out of 4 domains) >1.5 SD below the standardized mean.
3. Functional limitation as a result of cognitive impairment.

The determination of PD-MCI will be made based on the following factors:

1. Cognitive complaint by either the patient or informant (spouse, family member or friend).
2. Cognitive impairment defined as at least 2 test scores (out of 6 scores) from at least 1 domain (out of 4 domains) >1.0 SD below the standardized mean.
3. No functional impairment as a result of cognitive impairment.

Cognitive categorization will be performed annually. For subjects who have had visits prior to the implementation of this assessment, the site investigator may be asked to make the determination retrospectively based on his/her best clinical judgment. Retrospective determinations will be noted in the PPMI database.

University of Pennsylvania Smell Identification Test (UPSIT)

The University of Pennsylvania Smell Identification Test (UPSIT) is a forced-choice test (i.e., subject must choose one of the four choices, even if no smell is identified) in which subjects identify an odor among four response alternatives. The test has four booklets containing ten odorants each, one per page, for a maximum score of 40. The stimuli are embedded in scratch and sniff microcapsules fixed and positioned on strips at the bottom of each page. Lower scores indicate reduced odor identification.

Instructions for Administration and Scoring:

1. Instructions provided on the face page of Booklet 1 of 4 should be reviewed with the subject before beginning the test. DO NOT COMPLETE the address and other personal information requested on the back of Book 1.
2. Emphasize the importance of providing a response to every question, even if no odor is perceived. Subjects should understand that it is necessary to complete each item in order to make their test valid and reassure subjects that they may not smell each of the odors. Remind the subject not to seek help from other people to identify the odors.
3. The subject should understand the correct procedure for releasing the odors and should be encouraged to sniff the label immediately after it has been scratched. Be careful not to mark the test strip too thoroughly, for it is possible to eliminate the odor if this is done.
4. If a subject has difficulty scratching the label or reading the multiple choice responses, the coordinator may assist in releasing the odor and/or reading the responses aloud while the subject is sniffing the strip.
5. Responses should be recorded for each item on the columns provided on the last page of each booklet. Note that the number for each question corresponds with the number on the scoring cards.
6. If a subject has difficulty completing all 4 booklets at one time (e.g., short attention span or unwillingness to cooperate), it is permissible to spread the testing out over the period of the study visit.
7. Once all 40 items are completed, the total number of correct responses is established using the scoring key. Record the total number correct for each booklet in EDC on the UPSIT page.

The UPSIT booklets are considered source and should remain with the subject's source files.

NOTE: The smell test booklets may be used for up to 6 months after the stamped date that appears on the outside of the envelope.

APPENDIX 6

Assessment Order Form

Phenoconversion Guidelines Document

PARKINSON'S PROGRESSION MARKERS INITIATIVE
Play a Part in Parkinson's Research

PPMI Assessment Order Form

Site #:_________ Date____________

Please check your supplies well in advance and **allow 10 days** for CTCC to process and ship supplies.

Please be as accurate as possible when ordering the number of Baseline Visit Packets given that the UPSIT expires*.

_____ Baseline Visit Packets (UPSIT*, Benton, HVLT-1)

_____ V04/Month 12 Visit Packets (Benton, HVLT-2)

_____ V06/Month 24 Visit Packets (Benton, HVLT-3)

_____ V08/Month 36 Visit Packets (Benton, HVLT-4)

_____ V10/Month 48 Visit Packets (Benton, HVLT-5)

_____ V12/Month 60 Visit Packets (Benton, HVLT-6)

Individual assessment form (specify form name and amount needed below):

_____ ____________________________________

_____ ____________________________________

_____ ____________________________________

***UPSIT is good for 6 months past the stamped expiration date.**

Please email your request to PPMI_CTCC_ProjectManagers@chet.rochester.edu

P-PPMI Phenoconversion Guide

<u>Purpose:</u> The purpose of this document is to provide study investigators information regarding completion of the Diagnostic Features CRF and documentation to define phenoconversion to Parkinson's disease based on the UK Parkinson's Disease Society Brain Bank Criteria (Step 1 and 2) in unaffected individuals enrolled in prodromal cohorts for PPMI.

<u>Background:</u> Phenoconversion is a critical outcome for the prodromal cohorts for PPMI. There is no currently accepted definition for phenoconversion. It is acknowledged that phenoconversion to PD or a parkinsonian syndrome can vary among movement disorders specialists depending on experience and perspective. In PPMI, the approach described below was developed with the goal of attempting to standardize the definition of phenoconversion across the participating sites. The UK Parkinson's Disease Society Brain Bank Criteria will be used as a backbone, and the specific data obtained from the "Diagnostic Features" CRF will be used satisfy each of the criteria will be provided in italics.

Phenoconversion is a process that is fraught with uncertainty and in the research setting it is important to provide data that reflects our best answer at the time, understanding that it may change at future visits. It should be noted that phenoconversion based on the definitions provided below, may not be consistent with what you convey to the subject (if uncertain, you may not inform the subject your 'best guess' diagnosis) or influence your clinical management of the subject.

Importantly, although all the currently available forms and diagnostic criteria have been developed for PD specifically, in PPMI we are more focused on phenoconversion to a parkinsonian syndrome rather than PD specifically. Among phenoconverters we will then break out those phenoconverting to PD from those with an atypical parkinsonsian syndrome, though this will be a secondary anaylsis.

Note: If a subject presents with parkinsonism (2/3 cardinal signs) and it is unclear this is IPD versus an early atypical parkinsonian syndrome (mild cognitive change with parkinsonism, early DLB) it is recommended that the diagnosis Parkinson's disease on the 'Prodromal Diagnostic Questionnaire" be chosen, with the understanding that this can be updated at future visits as the diagnosis declares itself. It should be acknowledged that this is a cohort with prodromal or early signs of parkinsonism and it can be difficult, and in many cases impossible, to accurately differentiate PD from atypical parkinsonian syndromes (eg PSP, MSA, DLB) in this early stage based on clinical evaluation.

Below is a review of the UK Parkinson's Disease Society Brain Bank criteria and the related questions from the "Diagnostic Features" that will be used in PPMI to define phenoconversion.

<u>**UK Parkinson's Disease Society Brain Bank Criteria (UKPDSBBC)**</u>

The item from the PPMI 'Diagnostic Features" that addresses each of the criteria is provided in italics

UKPDSBBC Step 1. Diagnosis of a parkinsonian syndrome

Bradykinesia and at least one of the following:

- Muscular rigidity
- Rest tremor (4-6 Hz)
- Postural instability unrelated to primary visual, cerebellar, vestibular or proprioceptive dysfunction

"Diagnostic Questionnaire" used in PPMI *- a diagnosis of PD will be assigned if:*
Bradykinesia is present and typical for PD ("Diagnostic Features" CRF question 7.1 must be 1) and at least one of the following:

- *Rigidity is present and typical for PD ("Diagnostic Features" CRF question 6.1 = 1)*
- *Resting tremor present and typical for PD ("Diagnostic Features" CRF question 5.1 = 1)*
- *Postural and gait disturbances are completely typical of PD ("Diagnostic Features" CRF question 8.1 = 1)*

UKPDSBBC Step 2. Exclusion Criteria for PD (for reference)

The following excludes a diagnosis of PD

- History of repeated strokes with stepwise progression of parkinsonian features
- History of repeated head injury
- History of definite encephalitis
- Neuroleptic treatment at onset of symptoms
- MPTP exposure
- Oculogyric crisis
- More than one affected relative
- Sustained remission
- Strictly unilateral features after one year
- Supranuclear gaze palsy
- Cerebellar signs
- Babinski sign
- Early severe autonomic involvement
- Early severe dementia with disturbances of memory, language, and praxis

- Presence of cerebral tumor or communicating hydrocephalus on CT scan
- Negative response to large doses of levodopa (if malabsorption excluded)

"Diagnostic Questionnaire" used in PPMI – A diagnosis of PD/parkinsonian syndrome will be excluded if any of the following are met:

- Excessive stroke risk factors (e.g. diabetes, hypertension, cardiovascular disease) or past symptoms suggestive of cerebrovascular disease ("Diagnostic Features" CRF question 1 = "1" (yes)). **Note:** A diagnosis of hypertension, diabetes or cardiovascular disease would not warrant an answer of "1" (yes) on this question. The subject would need to have cerebrovascular disease that the investigator feels may be responsible for the subject's parkinsonian symptoms to warrant an answer of "1" (yes).

- Unusual or atypical risk factors, exposure, or past history (e.g. drug exposure, acute or chronic toxin exposure, acute infection preceding parkinsonism, repeated head trauma, boxer) ("Diagnostic Features" CRF question 2 = 1). **Note:** A diagnosis of remote drug exposure or head trauma would not necessarily warrant an answer of "1" (yes) on this question. The subject would need to have drug exposure or head trauma significant enough that the investigator feels it may be responsible for the subject's parkinsonian symptoms to warrant an answer of "1" (yes).

- Oculomotor disturbances disease ("Diagnostic Features" CRF question 13 = 1) or Eyelid disturbances (e.g. "apraxia of eyelid opening, blepharospasm) ("Diagnostic Features" CRF question 14 = 1).
  Note: Only answer "1" (yes) if the subject demonstrates oculomotor disturbances or eyelid disturbances that leads the investigator to suspect a diagnosis other than a parkinsonian syndrome.

- Wide based gait or ataxia ("Diagnostic Features" CRF question 8.2 = 1). **Note:** Only answer "1" (yes) if the subject demonstrates a wide-based gait or ataxia that s leads the investigator to suspect a diagnosis other than a parkinsonian syndrome.

- Mental changes: Cognitive ("Diagnostic Features" CRF question 9.2 = 1) **Note:** Only answer "1" (yes) if the subject demonstrates cognitive changes that leads the investigator to suspect a diagnosis other than a parkinsonian syndrome.

- Autonomic disturbances ("Diagnostic Features" CRF question 12):
  - Postural hypotension ("Diagnostic Features" CRF question 12.1 = 1)
  - Sexual dysfunction ("Diagnostic Features" CRF question 12.2 = 1)
  - Urinary dysfunction ("Diagnostic Features" CRF question 12.3 = 1)
  - Bowel dysfunction ("Diagnostic Features" CRF question 12.4 = 1)

Note: Only answer "1" (yes) to any of the above autonomic symptoms if the subject demonstrates significant autonomic signs or symptoms that lead the investigator to suspect a diagnosis other than a parkinsonian syndrome (ie primary autonomic failure).

- Other neurological abnormalities atypical of parkinsonism (e.g. hyperreflexia, Babinski sign, sensory deficit, amyotrophy, limb apraxia, sleep apnea, dsymetria or other cerebellar dysfunction) ("Diagnostic Features" CRF question 15 = 1.] **Note:** Only answer "1" (yes) if the subject demonstrates significant neurological signs or symptoms that lead the investigator to suspect a diagnosis other than a parkinsonian syndrome. Sleep apnea alone should not warrant an answer of "1" (yes) to this question given that it can commonly co-occur with parkinsonian syndrome.

- Little or no response to levodopa or a dopamine agonist ("Diagnostic Features" CRF question 16 = 1). **Note:** Only answer "1" (yes) if the subject has had an adequate trial of dopaminergics with no response and the investigator suspects a diagnosis other than a parkinsonian syndrome.

- Presence of very rapid speech (tachyphemia) ("Diagnostic Features" CRF question 17 = 1). **Note:** Only answer "1" (yes) if the subject demonstrates speech changes that leads the investigator to suspect a diagnosis other than a parkinsonian syndrome.

- Presence of dysphagia or other bulbar dysfunction ("Diagnostic Features" CRF question 18 =1). **Note:** Only answer "1" (yes) if the subject demonstrates swallowing changes that leads the investigator to suspect a diagnosis other than a parkinsonian syndrome.

- CT is suggestive of another cause of parkinsonism ("Diagnostic Features" CRF question 19 = 1) or MRI is suggestive of another cause of parkinsonism ("Diagnostic Features" CRF question 20 = 1). **Note:** Only answer "1" (yes) if there is a finding on CT or MRI that leads the investigator to suspect a diagnosis other than a parkinsonian syndrome.

- Is there anything unusual or atypical about this subject's disease (eg presentation, symptoms, signs, course, response to therapy, etc.), which could indicate an alternative diagnosis to Parkinson's disease (ie idiopathic parkinsonism with the presence of Lewy bodies in the substantia nigra), no matter how remote? ("Diagnostic Features" CRF question 21 = 1) **Note:** Only answer of "1" (yes) if the subject demonstrates signs that would be unusual or atypical for a parkinsonian syndrome or that leads the investigator to suspect a diagnosis other than a parkinsonian syndrome.

PARKINSON'S PROGRESSION MARKERS INITIATIVE

Play a Part in Parkinson's Research

SECTION 7

REPORTABLE EVENTS

Incident Reporting
Notification Reporting
Adverse Event and SAE Reporting

Version: May 2015

INCIDENT AND NOTIFICATION REPORTING

When notifying the CTCC of an event, please have the following information available:

- Caller's staff code
- Subject ID Number (if applicable)
- Date of notification/event
- Details surrounding the event

<u>INCIDENTS (REPORTABLE EVENTS)</u>
(See Protocol Section 10)

The objective of the incident reporting process is to alert key personnel overseeing the study regarding all experiences that may influence the safety of individual subjects or the subjects in the study as a whole. Incidents may be reported by the Site Investigator or Coordinator. The following events are considered incidents and must be reported to the CTCC Project Manager within 24 hours of the time they occur or become known to site staff:

- **Initiation of PD Medication**
  - PD medications may be initiated at any time after enrollment at the discretion of the patient or treating physician. It is important for sites to report this information to the CTCC as it may impact the type of visit that is conducted when the subject returns for the next protocol visit. The CTCC will be able to provide guidance to sites to ensure proper protocol procedures are followed.
- **Change of Diagnosis**
  - Although each subject is enrolled into the study with or without the clinical diagnosis of PD, it is understood that further evaluations or changes may occur to a subject over a period of 3-5 years that may result in a new or different diagnosis.
- **Participation in Another Clinical Trial (See protocol section 7.1.3)**
  - If it is known that a subject is participating in another clinical trial, we would like to track this information. Per protocol, this is allowed after the first year of participation in PPMI. If the subject is participating in a clinical trial, be sure to indicate the study drug information (if known) on the Concomitant Medication Log, recorded as "name of study medication/placebo".
- **Premature Withdrawal of Subject**
  - Premature Withdrawal visit assessments should be performed at time of withdrawal. If a subject discontinues early without scheduling a final follow-up visit, there should be documentation in the source notes as to the reason why a final visit was not conducted.
- **Premature Withdrawal for TAP**
- **Serious Adverse Event (occurring in the appropriate AE follow up window) – Must be reported by phone or email.**
- **Pregnancy (reported by female subject or female partner of a male subject)**

- **Death**
- **Change from Genetic Cohort to Genetic Registry – must receive approval, please contact CTCC PM for guidance.**
- **Change from Genetic Registry to Genetic Cohort- must receive approval, please contact CTCC PM for guidance.**

- **SWEDD continuing beyond 24 months**
  With IRB/EC approval of Amendment 8, eligible SWEDD subjects may choose to continue in PPMI beyond the initial 2 year follow up. Sites should contact the CTCC PM if they have identified an eligible SWEDD subject that would like to remain in PPMI. If the subject agrees to remain in the study, the site should contact the CTCC for specific instructions.

- **Permission for forego lumbar puncture**
  This report is used to document permission given to prodromal subjects to enroll in PPMI with permission from PPMI leadership to forego the baseline lumbar puncture. It is expected that sites will continue to assess a subjects willingness to have the lumbar puncture at each annual visit. Please contact the CTCC for specific information.

The incident will be entered into an on-line module and immediately distributed by email to the Principal Investigator, Steering Committee, and study team members.

The site will also receive an e-mailed report for review after the call. This report should be kept with the subject's source binder.

NOTIFICATIONS

The purpose of the notification process is to detail all noteworthy and relevant clinical or data management decisions that might influence the interpretation of the study data. The Coordinator, Investigator or Site Monitor may report notifications.

The following events are examples of issues requiring notification and should be reported to the CTCC Project Manager:

- Eligibility issues
  - Site must report any questions or issues regarding eligibility as soon as possible before completing the RANDOM page in EDC. If any eligibility criteria are not met, you will not be able to continue past the RANDOM page without obtaining a protocol deviation code from the CTCC.
- Issues with any protocol procedures
- Issues with DaTSCAN™ delivery

- Site specific issue(s)

The final Notification Report describing the event will be emailed to the site. The Site Coordinator should review the content of this report for accuracy. The CTCC should promptly be notified if any additional information or corrections are needed. This report should be kept with the subject's source binder. Protocol deviations with DAT imaging at sites will be documented by IND and forwarded to CTCC.

REPORTING OF ADVERSE EVENTS

Definitions and techniques for assessing Adverse Events (AEs) and Serious Adverse Events (SAEs) are described in Section 9 of the protocol.

- For the purposes of reporting AEs/SAEs, the adverse event monitoring timeline is from the date of an in-person study visit when LP, Dopamine transporter SPECT imaging, [^{18}F] Florbetaben imaging activity or skin biopsy is conducted until 7 to 10 days following such activity. For sites conducting [^{18}F] AV-133 PET imaging, the adverse event monitoring timeline is from the date of an in-person study visit when [^{18}F] AV-133 PET imaging activity is conducted until 48 (±24) hours following such activity.

The CTCC must be notified within 24 hours of site's awareness of any SAE, including death. The SAE reported by phone or email will be entered into an Incident Module and will be sent to the Clinical Monitor, Principal Investigator, Steering Committee, and other study staff via e-mail.

The following procedures must also be followed within the 24 hours:

- Complete MEDWATCH Form 3500A
  - See ePortal Operations folder to obtain a copy of the form to complete
  - General instructions for completion are below

- Email Form 3500A to the CTCC Project Manager
- The report will be reviewed by the Clinical Monitor and forwarded to IND
- Corrections or additional information may be requested; updates should be noted on a new form and emailed to the CTCC Project Manager
- AV-133: adverse events (and SAEs) are captured and assessed on the day of ^{18}F-AV-133 injection and PET imaging up through the time of the 48 hour reporting telephone call. If an SAE is noted, the Eli Lilly SAE reporting form should be completed. **Initial and follow up reports are forwarded to Eli Lilly by the site within 24 hours of site awareness of the event.** The report will be reviewed by study staff at Lilly to assess whether reporting to FDA or other regulatory agencies is necessary. Copies of the following documents should be provided:
  - Completed Eli Lilly SAE reporting form
  - Other information upon request

  Note: SAE follow-up updates need to be made on the original Eli Lilly SAE reporting form by crossing out and initialing revisions.

General Instructions for Completion of MEDWATCH Form 3500A

- All entries should be typed or printed in a font no smaller than 8 point.
- Complete all sections that apply. If information is unknown, not available, or does not apply, the section should be left blank.
- Dates should be entered as mm/dd/yyyy. If exact dates are unknown, provide an estimate.
- For narrative, if fields don't provide adequate space, attach additional page(s).
- Enter page number and total number of pages submitted (include attachments in total) where *Page_of_* are indicated.
- Complete Section A
  - A1 – indicate Subject ID number (do not use initials)
  - A2 – indicate subject's age at time of event
  - A3 and A4 – enter subject's gender and weight
- Complete Section B (if applicable)
  - B1 – indicate Adverse Event
  - B2 – check all outcomes that apply to the event
  - B3 – provide actual or best estimate of the date of the first onset of the AE; if day is unknown, month and year are acceptable
  - B4 - indicate date the report is filled out
  - B5 – describe the event in detail, including a description of what happened and summary of relevant clinical information
  - B6 – enter results of any relevant tests or lab data
  - B7 – ensure the Medical History eCRF is complete in EDC
- Complete Section C
  - C10 – ensure the Concomitant Medication Log is up to date in EDC
- Complete Section E
  - E4 – mark "No"; sites will not send report to FDA

Notifying the IRB/EC and Subjects

Institutional guidelines should be followed in notifying a site's IRB and Radiation Safety Committee of SAE's (safety reports) and in communicating relevant information to subjects. The site must maintain a copy of IRB/Ethics notification and of the IRB/Ethics acknowledgement of receipt of any serious adverse event or safety report that is submitted.

PARKINSON'S PROGRESSION MARKERS INITIATIVE
Play a Part in Parkinson's Research

SECTION 8

LABORATORY SPECIMEN MANAGEMENT

Covance Lab Manual
[Insert Copy]

Research Biomarkers Laboratory Manual
[Insert Copy]

Version: May 2015

PARKINSON'S PROGRESSION MARKERS INITIATIVE
Play a Part in Parkinson's Research

SECTION 9

SOURCE DOCUMENTATION
Instructions for Source Documentation

APPENDIX 9
Source Documentation Worksheets
[Insert blank set of unique forms]

SOURCE DOCUMENTATION

Per FDA and ICH guidelines, source documents include the first record of any subject data, regardless of the medium used to record the data. These documents may include subject's medical records, progress notes, lab reports, and so on.

The PPMI study uses electronic data capture (EDC). Sites are responsible for maintaining adequate source documents for this study. Sites must keep visit and subject correspondence notes in addition to use of the Source Document Worksheets (SDWs). Subject study charts must be made available to the site monitor for review. SDWs for all of the eCRFs that will be used in this study are located in the ePortal "Source Document Worksheets" folder. Sites are responsible for printing source documents worksheets from the ePortal "Source Document Worksheets" folder.As an alternative, sites can save the most current version of the PPMI SDW to a portable data storage device (i.e., thumb drive) and indicate in the study regulatory binder where the drive will be stored. In the event connectivity is lost with the eClinical system prior to a study visit, SDWs can be copied from the paper version or printed from electronic copy saved to the portable storage device.

The Investigator or Coordinator may record directly on the SDWs and supplement with additional information as needed. Any written or dictated note must, at a minimum, contain information specified in the worksheet.

You should carefully record the process used by your site when consenting an individual subject. Include in your source that the subject was given a copy of the signed consent.

The following SDWs will be considered source for data entered into EDC:

- Subject Identification Log (Note: Source only – not entered in the database)
- Delegation Log (Note: Source only – not entered in the database)
- Screening Demographics
- Socio-Economics
- Unique ID
- PD Features / Diagnostic features of PD
- Primary Diagnosis
- Medical History (General)
- Family History (PD) / Family History of PD log
- Inclusion/Exclusion
- General Physical Exam
- General Neurological Exam
- Diagnostic Questionnaire
- Vital Signs

- Genetic Mutation Testing FormCognitive Assessments (Time of Administration)
- Letter-Number Sequencing
- Epworth Sleepiness Scale
- REM Sleep Disorder Questionnaire
- GDS-15
- QUIP
- SCOPA-AUT
- Modified S&E Activities of Daily Living
- PASE
- Cognitive CategorizationPregnancy Form
- Laboratory Procedures
- DNA Sample
- Clinical Labs
- Whole blood sample
- Magnetic Resonance Imaging
- Lumbar Puncture
- DaTCAN Imaging
- Surgery for PD
- Florbetaben PET Imaging
- VMAT-2 Imaging (AV-133)
- Skin Biopsy
- Use of PD Medications
- Telephone Follow-up
- SWEDD Continuation Form
- Conclusion of Study Participation
- Visit Status
- Investigator Signature
- Signature Form
- Subject Site Transfer Form
- Change of Category Form
- Genetic Counseling Log
- Consent / Withdrawal of consent for future procedures
- Advance Directive/ Review Continuing Ability to Consent
- FOUND – consent to share contact information
- Current Medical Conditions Log
- Concomitant Medication Log
- Adverse Event Log

The following worksheets/booklets will be considered source for data entered into EDC:

- MDS-UPDRS & Hoehn & Yahr packet
- Montreal Cognitive Assessment (MoCA) worksheet

- Hopkins Verbal Learning Test booklet
- Benton Judgment of Line Orientation score sheet
- Semantic Fluency response sheets – Animals, Fruits, Vegetables
- Symbol Digit Modalities response sheet
- State-Trait worksheet
- UPSIT booklets

The progress notes/source documentation/medical records should contain information about whatever has happened to the subject during the course of the study, especially if it is not captured on the CRF worksheet or visit notes. Please keep your source documents either with your CRF worksheets or in a subject binder, available at the time of the site monitoring visit.

APPENDIX 9

Source Documentation Worksheets

[Insert blank set of unique forms or a note to file indicating the SDW will be stored electronically on a portable storage device and the location of the device]

PARKINSON'S PROGRESSION MARKERS INITIATIVE

Play a Part in Parkinson's Research

SECTION 10

MONITORING

Study Initiation
Site Visits
Interim Monitoring Visit(s)
Clinical Site Close Out Visit
Contents of Regulatory Binder

Version: May 2015

MONITORING

Study Initiation

Attendance at an Investigator/Coordinator orientation meeting is required. This meeting can occur in person or via phone. The objective of the orientation is to ensure that study site personnel understand the protocol and their individual responsibilities, as well as all of the regulatory requirements. During the site orientation meeting, the following items will be reviewed:

- Protocol and Data Forms
- AEs and SAEs
- Biological samples, storage and shipment
- DaTSCAN supplies
- Investigator obligations
- Record keeping
- Study files
- Source documentation
- Advertisement for enrollment

Site Visits

As per federal guidelines, periodic study monitoring visits will be conducted at the sites.

The Project Manager at the CTCC will work closely with the site monitors to provide consistent answers to investigators and coordinators regarding their protocol questions during monitoring visits.

After each monitoring visit, the study monitor will produce a site monitoring report that is reviewed at the CTCC. The study monitor will send a follow up letter to the site reiterating any discussions that occurred at the monitoring visit and listing any action items.

Interim Monitoring Visit(s)

The first monitoring visit will occur after a site has enrolled at least 2 subjects. Sites will then have in-person monitoring visits approximately once a year thereafter. More frequent visits may occur at high enrolling sites. At these visits, the Investigator, Coordinator, and any other staff members involved in the study should be available (or easily reachable). During this visit, the following will be reviewed:

- Adequacy of study facilities
- Receipt, storage, and accountability for DaTSCAN™ , F-AV-133 and Florbbetaben.
- Daily log of minimum/maximum temperatures reached for freezers
- Ensuring that the study staff has a good understanding of the protocol and proper procedures for CRF completion and data entry
- Regulatory binder, protocol, case report form worksheets, adverse event reporting, medical record (source) documentation, and informed consents (to assure that the inclusion/exclusion criteria have been properly met, that data forms are completed correctly, and the documentation adheres to the Good Clinical Practice (GCP) requirements)
- eCRFs of currently enrolled subjects will be reviewed for completeness and accuracy and compared to the source documentation
- Study correspondence, including correspondence between the Investigator and the site's IRB, and between the site staff and the CTCC

Clinical Site Close Out Visit

The monitor is responsible for a clinical site close out visit when all subjects have completed the study at that site. At the close out visit, final accountability for all DaTSCAN, AV-133 and Florbetaben will be conducted. Monitors will also conduct a review of eCRFs and logs for those subjects not previously monitored. The site's regulatory binder will be reviewed for all regulatory documents and correspondence pertaining to the study. Record retention requirements will also be discussed. These visits may be occur in-person or remotely, depending on the needs of the study at the time of close out.

Contents of the Regulatory Binder

The site must maintain a Regulatory Binder (also referred to as the Investigator's file or study file). This binder will be reviewed at every monitoring visit and should therefore be kept up-to-date.

The Investigator's Regulatory Binder should contain the following:

- Form 1572
- Current CV's and licenses (if applicable), including DEA license for individual ordering DaTSCAN™
- Protocol and Amendments (when applicable)
- Protocol Investigator Agreement Signature Page

- IRB Communication
  - Approval letter(s) for protocol, consent form, recruitment materials, amendments, sub-studies
  - Approved consent form
  - Approved recruitment and subject materials
  - IRB Membership Inquiry Form
  - Correspondence to/from IRB
  - Study closeout letter to IRB
- General Correspondence
- SAE's (if applicable)
  - Completed SAE forms
  - Correspondence regarding SAE forms
- Incident Reports (if applicable)
- Notifications (if applicable)
- Confidentiality Agreements
- Disclosure Agreements/Conflict of Interest Statements
- Monitoring Log
- Delegation Log
- Notes to File
- Blank paper or electronic copy of each unique CRF Worksheet (in Regulatory Binder or Operations Manual). If an electronic version is being stored, please write a note to file indicating the SDWs will be stored in this manner along with the location of the portable storage device.
- Investigator Drug Brochure
- Lab documentation and CLIA waiver certification (if applicable)
- Human Subject Education certifications
- MDS-UPDRS certification (for Investigators and Sub-investigators)
- Staff Training Documents
- DaTSCAN™ , AV-133 (if applicable), Florbetaben (if applicable) and MRI related documents and logs

PARKINSON'S PROGRESSION MARKERS INITIATIVE

Play a Part in Parkinson's Research

SECTION 11

REPORTS

Enrollment Projections Report

Enrollment By Diagnosis, Gender, Age

Incident Report

Notification Report

Pending Queries Report

Missing Pages Report

Missing Investigator Signature Report

Site Performance Report

PPMI STUDY REPORTING

Described below are some of the reports generated for the PPMI study to help the Steering Committee and study team manage the project and ensure sites are meeting expectations and goals of the study. In addition, some reports are generated to assist sites in keeping track of what may be missing as a result of all the data that needs to be entered.

ENROLLMENT PROJECTIONS REPORT

- Generated from information entered on Question 5 of the Screening/Demographics page, "Projected Enrollment Date".
- Tracks projected baseline visits at each site that are scheduled within the next 30 days, over 30 days, and indicates if a visit is beyond the projected date indicated in EDC.
    - If projected enrollment dates are confirmed or changed during the screening period, make sure to update that field in EDC.
- Distributed on a bi-weekly basis to site Investigator and Coordinator so that sites are able to see where they rank in enrollment status relative to all other sites.
- Steering Committee, sponsor and monitors also receive this report in order to monitor study progress as well as site recruitment and enrollment performance.

ENROLLMENT BY DIAGNOSIS, GENDER, AGE

- A summary of total enrollment at each site, as well as for the whole study, separated out by subject group, gender and age.
- A subject appears on this report once the RANDOM page is complete and the subject is enrolled into the study.
- This report will be used by the Steering Committee and study team to monitor the overall study population, particularly in regard to age and gender balance for subjects enrolled, as discussed in Section 4.3 of the protocol.

INCIDENT REPORT

- Generated when a site notifies CTCC of any protocol specified reportable event as outlined in Section 10 of the protocol. (Also see Section 7 of this manual.)
- This report is a summary of the event that occurred and is distributed to the site Investigator and Coordinator.
- Review the report to ensure accuracy and file a copy in the study binder.

NOTIFICATION REPORT

- Generated when a site informs the CTCC of any relevant clinical or data management issues that should be noted (see Section 7 of this manual).
- A summary of the event that occurred is distributed to the site Investigator and Coordinator.
- Review the information to ensure accuracy and file a copy in the study binder.

PENDING QUERIES REPORT

- Generated when a query is left unresolved 5 days beyond the creation date.
- Distributed to site Coordinator on a weekly basis or more frequently during close out.

MISSING PAGES REPORT

- Generated when an expected page is not entered within 5 days of the Target Visit Date for an individual subject.
- Missing pages start to be reported once a subject is enrolled (RANDOM page is completed and the Visit Window Schedule is generated).

MISSING INV eSIGNATURES

- For any completed visit that does not have the electronic signature of the Investigator applied to the Signature Form or Investigator Signature Form.
- The primary Investigator or designated Sub-investigator who conducted the visit should sign off on the visit.
- Report is sent only to the primary designated Investigator at each site.

SITE PERFORMANCE REPORT

- Tracks expected forms received (data entered) and the timeliness of data entry (i.e., within 5 days of the visit).
- Tracks how many queries are generated at each site and how timely the site addresses queries (i.e., timely is a comparison of origination date to the resolution date)
    - A combination of 100 missing pages and pending queries at any site will require phone follow-up from a CTCC Project Manager (PM). The PM will work with sites to help resolve any outstanding data entry or completeness issue. If the issues are not addressed and/or resolved during a 30 day time period, PPMI study leadership will be notified to help address/resolve identified issues.
- This report will be used by the study team to ensure timely entry of data, as well as accurate and clean data, which is essential to this project because data is transferred to LONI on a daily basis. Any significant issues will be addressed directly with a site.

PARKINSON'S PROGRESSION MARKERS INITIATIVE

Play a Part in Parkinson's Research

SECTION 12

SUBJECT SITE-TO-SITE TRANSFER PROCESS

Appendix 12

Sample Medical Records Release Form

GENERAL INFORMATION

When a subject is planning to relocate and would like to continue participation in the study, please follow the process below for transferring a subject from one site to another. For purposes of clarity, the current site will be referred to as the "transferring" site, and the site the subject is *transferring to* will be referred to as the "new site".

SUBJECT TRANSFER PROCEDURES

- The transferring site coordinator will contact the PPMI Project Manager if a subject transfer is to occur. The PPMI Project Manager will help identify a potential site (or sites) in or nearest to the subject's new location and will contact the new site coordinator to ensure that the site is willing to accept the subject.
- The transferring site coordinator must then contact the new site coordinator to provide information about the subject's course in the study.
- The transferring site investigator and coordinator will discuss with the subject the importance of establishing a clinical care provider in the subject's new location.
- The transferring site coordinator must work with the new site coordinator to facilitate the transfer and set up an appointment for the subject at the new site prior to the transfer. The PPMI Project Manager must be informed of when the appointment will occur.
- The transferring site coordinator will provide the subject's forwarding contact information to the new site.
- The subject should sign (at the transferring site) an institution-specific records release form in order for the subject's study information to be transferred to the new site (see sample form in Appendix 12).
- The transferring site must ensure all data for the subject have been entered into EDC and, importantly, **all queries must be resolved, missing data provided, and signatures completed.** The transferring site will work with the PPMI Information Analyst to complete this task. This step is critical, as once the subject is officially 'transferred', eDE access to the subject's data will no longer be available to the transferring site. Completing this step will also ensure site payment can be processed for any completed visits at the transferring site.
- The transferring site will print all eCRFs completed for the subject once the data have been confirmed by the PPMI Information Analyst as 'clean' (which must occur prior to the transfer completion).
- The transferring site will provide a copy of all study documentation (including a copy of the signed consent, incidents and notifications, DaTSCAN shipment and accountability forms, lab reports, subject-related correspondence, research visit notes and source document worksheets) to the new site once an appointment has been confirmed, and prior to the subject's first visit at the new site.

- The transferring site must also ensure the appointment has occurred. If the subject fails to report for his or her consent visit at the new site, the new site will contact the subject to determine continued interest. If agreeable, another appointment should be made. However, if the subject wishes to withdraw from the study, the transferring site is responsible for contacting the PPMI Project Manager to report the incident, as the subject remains the site's responsibility until a transfer visit has occurred at the new site.
- The subject must be re-consented at the new site using the new site's consent prior to any visit activities occurring at the new site. It is recommended that the new site conduct a transfer visit for this purpose. The transfer visit may occur in conjunction with the subject's routine protocol visit schedule, or independent of the subject's next expected protocol visit if not due to occur. (Note if both the transfer event and routine protocol visit occur on the same day, it will be necessary to complete a separate signature page for each event). At this point, the new site assumes the responsibilities for the subject and the transferring site is relinquished of these responsibilities.
- The *Subject Site Transfer Form* (eCRF) must be completed by the new site on the day consent is obtained at the new site.
- The new site coordinator must contact the PPMI Information Analyst to confirm completion of the transfer and to activate the subject in EDC at the new site.
- Once the subject's data is visible to the new site, the new site will add a transfer event to the subject's event schedule in EDC. For instruction on adding an event, see the posting to eClinical training folder entitled "adding an event". The event that you choose will have an event code of X01, X02, etc.
- The Subject ID will remain the same following the transfer; however, all data completed by the new site will contain the new site number.

CTCC Follow-Up Tasks

- The CTCC will inform Covance, study cores and study team of subject's new site.

APPENDIX 12

Sample Medical Record Release Form

PARKINSON'S PROGRESSION MARKERS INITIATIVE
Play a Part in Parkinson's Research

[STUDY ACRONYM]
STUDY FILE RELEASE AUTHORIZATION

TRANSFERRING SITE #: ☐☐☐ **SUBJECT ID:** ☐☐☐☐☐

NEW SITE #: ☐☐☐

This [STUDY ACRONYM] Study File Release Authorization allows [NAME OF TRANSFERRING INSTITUTION] to release to NAME OF NEW INSTITUTION] all study files belonging to you which have been collected during your participation in the [STUDY ACRONYM] study

Release records to: [NAME OF NEW SITE INVESTIGATOR]
[ADDRESS OF NEW INSTITUTION]

These[STUDY ACRONYM] study files are to be used by [NAME OF NEW INSTITUTION] solely for the purposes of the [STUDY ACRONYM] study and will only be released, if needed, to those stated in the consent form.

This authorization will not expire unless you cancel (revoke) it. You can always cancel this Authorization by writing to the Site Investigator at:

[NAME OF TRANSFERRING SITE INVESTIGATOR]
[ADDRESS OF TRANSFERRING INSTITUTION]

If you cancel your Authorization to release your study files, you will also be removed from the study. However, standard medical care and any other benefits to which you are otherwise entitled will not be affected. Canceling your Authorization only affects use and sharing of information after the Site Investigator gets your written request. You can also refuse to sign this Authorization and not be part of the study. By signing this Authorization you give us permission to use and/or share your health information as stated above with [NAME OF NEW INSTITUTION].

Version Date: 01/29/2009

SAMPLE

While we will make every effort to keep information we learn about you private this cannot be guaranteed. Other people may need to see this information. While these people normally protect the privacy of the information, they may not be required to do so by law.

I have read (or have had read to me) the contents of this Authorization and have been encouraged to ask questions. I have received answers to my questions. I agree to release my [STUDY ACRONYM] study files to [NAME OF NEW INSTITUTION]. I have received (or will receive) a signed copy of this form for my records and future reference.

Signature of Research Subject	Printed Name	Date

Print Name of Authorized Representative	Date

Signature of Authorized Representative	Relationship to Subject

For Study Staff:

Person Obtaining Consent
I have read this form to the subject/authorized representative and/or the subject/authorized representative has read this form. An explanation of the Authorization request was given and questions from the subject/authorized representative were solicited and answered to the subject/authorized representative's satisfaction. In my judgment, the subject/authorized representative has demonstrated comprehension of the information.

Signature of Person Obtaining Consent	Printed Name and Title	Date

SAMPLE

Version Date: 01/29/2009
